# Deciphering Haplotypic Variation and Gene Expression Dynamics Associated with Nutritional and Cooking Quality in Rice

**DOI:** 10.3390/cells11071144

**Published:** 2022-03-28

**Authors:** Nitika Rana, Surbhi Kumawat, Virender Kumar, Ruchi Bansal, Rushil Mandlik, Pallavi Dhiman, Gunvant B. Patil, Rupesh Deshmukh, Tilak Raj Sharma, Humira Sonah

**Affiliations:** 1 Department of Agriculture Biotechnology, National Agri-Food Biotechnology Institute (NABI), Mohali 140306, India; nitika@nabi.res.in (N.R.); surbhi.kumawat@nabi.res.in (S.K.); virender.ku@nabi.res.in (V.K.); ruchi@nabi.res.in (R.B.); mandlik.r@nabi.res.in (R.M.); pallavi.dh@nabi.res.in (P.D.); rupesh@nabi.res.in (R.D.); trsharma@nabi.res.in (T.R.S.); 2 Department of Biotechnology, Panjab University, Chandigarh 160014, India; 3 Department of Plant and Soil Sciences, Institute of Genomics for Crop Abiotic Stress Tolerance, Texas Tech University, Lubbock, TX 79409, USA; gunvant.patil@ttu.edu; 4 Department of Crop Science, Indian Council of Agriculture Research (ICAR), Krishi Bhavan, New Delhi 110001, India

**Keywords:** allelic effects, gene expression dynamics, genetic variation, haplotypic network, molecular evolution

## Abstract

Nutritional quality improvement of rice is the key to ensure global food security. Consequently, enormous efforts have been made to develop genomics and transcriptomics resources for rice. The available omics resources along with the molecular understanding of trait development can be utilized for efficient exploration of genetic resources for breeding programs. In the present study, 80 genes known to regulate the nutritional and cooking quality of rice were extensively studied to understand the haplotypic variability and gene expression dynamics. The haplotypic variability of selected genes were defined using whole-genome re-sequencing data of ~4700 diverse genotypes. The analytical workflow identified 133 deleterious single-nucleotide polymorphisms, which are predicted to affect the gene function. Furthermore, 788 haplotype groups were defined for 80 genes, and the distribution and evolution of these haplotype groups in rice were described. The nucleotide diversity for the selected genes was significantly reduced in cultivated rice as compared with that in wild rice. The utility of the approach was successfully demonstrated by revealing the haplotypic association of *chalk5* gene with the varying degree of grain chalkiness. The gene expression atlas was developed for these genes by analyzing RNA-Seq transcriptome profiling data from 102 independent sequence libraries. Subsequently, weighted gene co-expression meta-analyses of 11,726 publicly available RNAseq libraries identified 19 genes as the hub of interactions. The comprehensive analyses of genetic polymorphisms, allelic distribution, and gene expression profiling of key quality traits will help in exploring the most desired haplotype for grain quality improvement. Similarly, the information provided here will be helpful to understand the molecular mechanism involved in the development of nutritional and cooking quality traits in rice.

## 1. Introduction

Rice, a staple food in many countries, contributes around 21% to the total per capita calorie intake across the globe [1,2]. Rice is consumed in several forms such as processed and fermented products. Many rice preparation methods depend on the grain size and stickiness of the cooked grains. In addition to being rich in carbohydrates, rice grains contain a low amount of essential micronutrients like calcium, zinc, iron, and phosphorus [3]. Hence, tremendous efforts have been made to improve the palatability and nutrient content of rice. Several genes determining essential mineral and provitamin content in rice have been identified and subsequently well-characterized to reveal the molecular mechanism [4]. An in-depth understanding of genetic regulation, as well as information about allelic variations and effects, is important to explore their utilization for breeding programs. One of the most noteworthy examples is the *semi-dwarf 1* or *SD1* gene with a loss-of-function allele, which has been effectively used for rice improvement [5]. Among the quality-related traits, grain size is one of the most important factors determining the market price of rice more particularly for premium quality basmati rice. Recently, a quantitative trait loci (QTL), *qGS3*, regulating grain size has been characterized in rice [6]. Several other QTLs/genes, e.g., *OsSPL16*, *OsSPL13*, *GL-2*, and *GL-7*, have been identified and are known to regulate grain size in rice [7,8,9,10]. Even though rice is highly important for nutritional value, more intensified efforts are needed to improve nutritional quality related traits like minerals and protein content. Recent studies have identified genes, such as *OsAAP6*, *TGP12*, and *OsGluA2,* that have a role in governing protein content in rice [11,12,13]. Now, the challenge is to utilize these genes through approaches like marker assisted breeding or haplotype-based breeding in rice. 

Recent advancement in the next generation sequencing technology has generated plenty of genomics data, which will be helpful for basic as well as applied aspects of rice research. Various studies aimed at the generation and curation of genomic resources have been undertaken over the past decade. One such endeavor is ‘The 3000 Rice Genomes Project’, which resulted in re-sequencing data for ~3024 diverse rice accession [14]. A similar effort has been employed to generate whole genome resequencing information for the 108 diverse rice varieties, which have been subsequently used to reveal the sequence variations in *O. sativa* Purpleputtu colored rice landrace [15]. Additional sequence information was generated for 950 rice accessions in one study, and ~533 accessions from another study [16,17,18]. Molecular markers were developed for yield-related traits by using re-sequencing data for 50 rice accessions that belonged to both cultivated and wild rice varieties [19]. Similarly, Qin et al. [20] developed a pan-genome resource with a genetically diverse panel of 31 rice accessions, which revealed 171,072 structural variants and 25,549 gene copy number variants. A noteworthy study for quantitative trait nucleotides (QTNs) pyramiding and breeding have been conducted by Wei et al. [21]. The ‘RiceNavi’ navigation system developed by Wei et al. (2021) for QTN pyramiding will be helpful to explore haplotypic diversity existed in rice germplasm. The system contains a comprehensive map of 348 QTNs and 51 structural variants along with 207 Single nucleotide polymorphism (SNPs) and 90 InDels. Numerous such efforts have resulted in a huge source of information on allelic variations for important agricultural traits. 

In addition to genome sequence information, transcriptomic resources illustrate the interactions of gene expression and regulation under different conditions, e.g., developmental stages, tissues, and growth conditions. Moreover, the co-expression of genes plays a pivotal role in understanding complex biological phenomena. Generally, genes sharing a common molecular pathway tend to have similar expression patterns. Identification of such genes enables the prediction of pathways, events in molecular responses, and importantly, which genes have a key role in the expression network. Variation in the regulatory genes or regulatory sequences (promoter) determines differential gene expression. Therefore, genomics and transcriptomics will be invaluable resources for a better understanding of trait development. 

Allelic information from cultivated, wild, and traditional rice varieties help in the identification of accessions more conducive to well-established breeding strategies. In addition to single alleles, combinations of heritable sequence variations (haplotypes) that result in superior agronomic traits are being utilized. Several studies aimed towards the development of ideal varieties using haplotype-based breeding approach. One such study identified superior haplotype patterns for 120 genes governing desired yield and quality traits in rice [22]. The haplotypes, which were identified on a core dataset of 150 rice accessions panels were then compared to mega rice varieties. Such studies utilizing haplotype-based breeding pave the way for combining superior haplotypes with high-yielding rice accessions to meet consumer-centric nutritional demands. One of the important outcomes of allele mining and genome sequencing is the development and utilization of sequence-based markers for breeding programs. Several marker assays based on such SNPs have been developed which include KASP (Kompetitive Allele-Specific PCR), TaqMan, SNPlex, Illumina Infinium BeadChip, Affymetrix Axiom [23].

In the present study, we have selected functionally characterized genes that are important for the nutritional and cooking quality-related traits in rice. Utilizing the available re-sequencing data for approximately 4700 rice accessions, we have studied the allelic variations in the key genes and deciphered the haplotype combinations. The utility of this approach and the generated information were demonstrated by showing the association of various haplotypes of genes governing the grain length and chalkiness traits, with different patterns and degrees of grain phenotypes. In addition, we have developed a transcriptomic atlas to explore the gene expression and co-expression networks for the selected traits. We have identified several functionally important SNPs that can be further utilized for the development of marker assays associated with cooking and nutritional quality traits in rice. 

## 2. Materials and Methods

### 2.1. Selection of Genes Governing Cooking Quality and Nutritional Value Related Traits

A total of 80 functionally characterized genes governing cooking quality and nutrient content were selected from the literature search and by using tools viz. FunRiceGenes [24] and OGRO (Overview of Functionally Characterized Genes in Rice Online database) on Q-TARO [25] (Supplementary Table S1). The SNP data for selected genes was retrieved for the set of 4726 rice accessions including 595 Indica I (IndI), 465 Indica II (IndII), 913 IndicaIII (IndIII), 786 indica intermediate, 767 temperate japonica (TeJ), 504 tropical japonica (TrJ), 241 japonica intermediate, 269 Aus, 96 VI/aromatic, and 90 intermediate types from the SNP Seek database at IRRI (https://snp-seek.irri.org/) (accessed on 20 August 2021) and from RiceVarMap2.0 (http://ricevarmap.ncpgr.cn/) (accessed on 20 August 2021). Insertions and Deletions were obtained for these genes by querying the 3kRG filtered dataset. 

SNPs from wild Asian rice and cultivated rice accessions were used for diversity analysis. The nucleotide diversity (π), expected nucleotide diversity (θ), and Tajima’s D were estimated with a sliding window approach by using TASSEL v5.0 (http://www.maizegenetics.net) (accessed on 1 September 2021) [26]. Diversity analysis of the selected genes in case of wild and cultivated rice was studied using an R-based Pegas package (Population and Evolutionary Genetics Analysis System) (https://cran.r-project.org/web/packages/pegas/index.html) (accessed on 1 September 2021) and ‘APE’ (Analyses of Phylogenetics and Evolution) implemented in Ecogems online tool (http://150.109.59.144:3838/ECOGEMS) (accessed on 1 September 2021).

### 2.2. SNP Variation and Effect Prediction

Variants Effect Predictor tool [27] at Ensembl Plants was utilized to check the impact of variations (SNPs and InDels) on gene function. The distribution of variants was obtained in terms of intron, exon, upstream, downstream, splice region, 3 prime and 5 prime UTR variants. Protein Variation Effect Analyzer (PROVEAN) tool was used to gauge the deleterious effect of SNPs [28]. Prediction of the SNP impact on the biological function was obtained for the amino acid changes and PROVEAN score at threshold −2.5. If SNP scores ≤−2.5, it is predicted to be ‘deleterious’ to the protein function, whereas values > 2.5 predict ‘neutral’ effects of sequence variations. 

### 2.3. Haplotype Variation in Nutritional Quality-Related Genes 

Haplotypes were deduced for sequence variations in the selected genes using the RiceVarMap v2.0 tool [29]. Sequence variations were retrieved within the gene along with 1 kb upstream and downstream of the gene’s open reading frame (ORF). The SNPs that caused non-synonymous, missense, splice region, frameshift, start lost, stop gained, or stop gained variations were used for haplotype analysis using RiceVarMap2.0. The ‘haplotype’ module from RMBreeding, Rice Functional Genomics and Breeding v2.0 was employed for mining associations between SNPs and the phenotypic data [30]. The analysis was conducted against the coding sequence of genes and maximum allele frequency of ≥0.01.

### 2.4. Haplotype Network Analysis

Haplotype network analysis was performed using re-sequencing data available for ~4000 rice accessions. The haplotypic networks were developed using the online RiceVarMap v2.0 database (RiceVarMap2 (ncpgr.cn)) (accessed on 4 September 2021). The haplotypes found in more than 10 rice accessions were used to construct the haplotype network and the plots were build using the pegas functions in R package (https://cran.r-project.org/web/packages/pegas/index.html) (accessed on 4 September 2021). The classification system based on rice isozyme classification was used to group the accessions into nine categories including Indica I, Indica II, Indica III, Indica Intermediate, Aus, Temperate Japonica, Tropical Japonica, Japonica Intermediate, and Intermediate (including aromatic and rest of the accessions).

### 2.5. Gene Expression Dynamics and Co-Expression Network for Grain Quality-Related Traits in Rice

The raw sequencing reads were retrieved in the form of fastq format from the NCBI Sequence Read Archive (SRA) database (https://www.ncbi.nlm.nih.gov/sra) (accessed on 10 September 2021). The retrieved dataset possess RNAseq transcriptomic data from different studies representing 102 independent libraries. The dataset includes transcriptome of different genotypes, seed development stages and different tissues like leaves, inflorescence, seeds, embryo, endosperm [31,32]. The details of the SRA datasets used in this study are provided in Supplementary Table S2. The raw reads were processed based on quality and other parameters and then mapped to the reference genome of rice assembly build 4.0 using CLC genomics Workbench [33]. The expression data in the form of read count for all the rice genes were normalized in the form of Reads Per Kilobase of transcript per Million mapped reads (RPKM). The RPKM values for selected genes were further analyzed and visualized using the tool Multiple Experiment Viewer (MeV-4.9.0) [34]. The data was adjusted with log2 transformation, followed by hierarchical clustering analysis. Euclidean distance metric was further used to cluster genes. Gene-specific spatio-temporal expression across tissues such as leaf blade/sheath, root, stem, inflorescence, anther, pistil, lemma, palea, ovary, embryo, endosperm was obtained at Rice Expression Profile Database (RiceXPro v3.0) [35].

In addition to the 102 RNA-Seq libraries which were processed in-house, recently published 11,726 transcriptome libraries were queried for all the selected 80 genes, which are available at the Plant Public RNA-seq Database (PPRD, http://ipf.sustech.edu.cn/pub/plantrna/) (accessed on 15 February 2022) [36]. Subsequently, the gene co-expression network for data derived from these libraries was generated. The co-expression network was developed by CoExpNetViz plugin of Cytoscape [37] and visualized using Cytoscape software v3.7.2 [38]. The Pearson product-moment correlation coefficient was used as the correlation method at thresholds 5 and 95 for lower and upper percentile ranks, respectively. Furthermore, Weighted Correlation Network Analysis (WGCNA) v1.68 [39] was utilized to identify functional modules and groups of 80 query genes. RPKM values of these genes calculated from RNAseq experimental data were used as an input for the package. Module size varied for 3 genes, 4 genes, and 5 genes were considered to obtain an optimum number of modules. Module size 3 was selected for further analysis.

### 2.6. Quantitative Real-Time PCR Analysis

To study the expression profile of selected candidate genes, three different tissues including root, stem, and leaves were considered. Different tissues were harvested and immediately flash-frozen in liquid nitrogen and subsequently used for total RNA extraction. Spectrum™ Plant Total RNA Kit was used to extract RNA. The quantity and quality of the total RNA were evaluated using Nanodrop and agarose gel electrophoresis. Subsequently, high-quality RNA samples were used for cDNA synthesis using the RevertAid First Strand cDNA Synthesis Kit (Thermo Scientific™, Waltham, MA, USA). SYBR^®^ Green Master Mix (Bio-Rad, Hercules, CA, USA) 2× was used for the quantitative real-time PCR (qPCR) reaction. Each reaction comprises of 5 µL SYBR^®^ Green master mix, 1 µL template cDNA, 2 µL water, and 1 µL of each primer (10 mM). The qPCR thermal profile an initial denaturation at 95 °C for 1 min, followed by 40 cycles of denaturation at 95 °C for 10 s, and annealing at 55 °C for 20 s, and extension at 72 °C for 20 s. The qPCR was performed in 96-well plates. After 40 cycles, a melting curve analysis was performed with a stepwise increase in temperature over 65 °C to 95 °C by an increment of 0.5 °C every 5 s. The mean CT and standard deviations were calculated. 

### 2.7. Transcription Factors and Their Interaction with Nutritional Quality-Related Genes

Interactions between transcription factors and 80 genes governing grain quality and nutritional traits were predicted using PlantRegMap (Plant Transcriptional Regulatory Map) server [40]. Rice promoter sequences were first retrieved from EnsemblPlants through the Biomart tool [41]. The promoter sequences were used as an input for the ‘Regulation Prediction’ tool of PlantRegMap. A *p*-value threshold of 1.00 × 10^−5^ was set for the prediction of significant binding sites. 

### 2.8. Phenotyping of Rice Grains for Chalkiness Trait

Grain chalkiness and translucence were visualized for rice accessions representing different haplotypic groups. Whole rice grains were dried, de-husked, polished, and multiple transverse sections were obtained using a handheld microtome cutter with a changeable razor blade. The cross-sections were mounted on the specimen holder and coated with 5 NM Chromium Gold for visualization with a field emission scanning electron microscope (FESEM). The images were captured at 10,000× magnification and 10 µm resolution. 

## 3. Results

### 3.1. Haplotypic Diversity in Nutritional and Cooking Quality-Related Genes in Rice

Significant haplotypic diversity was observed in 80 grain quality related genes (Supplementary Table S1). A total of 6,572,189 SNPs were retained after filtering for missing calls per sample (<31%), missing calls per variant (<20%), and minor allele frequency per variant (>1%). The maximum numbers of SNPs were obtained for gene *Kala4|OsS1*, which is responsible for pericarp color in rice (Supplementary Table S1). A maximum of 56 missense mutations were observed in the *qCdT7|OsHMA3* gene, which is responsible for the grain zinc content in rice. No missense mutations were observed in nine genes (Supplementary Table S1). The functional amino acid change prediction revealed a total of 133 deleterious mutations in 39 genes (Table 1). A maximum of 30 deleterious mutations were observed in the *FLO2* gene, which is known to regulate grain size (Table 1 and Table S1).

**Figure 1:**
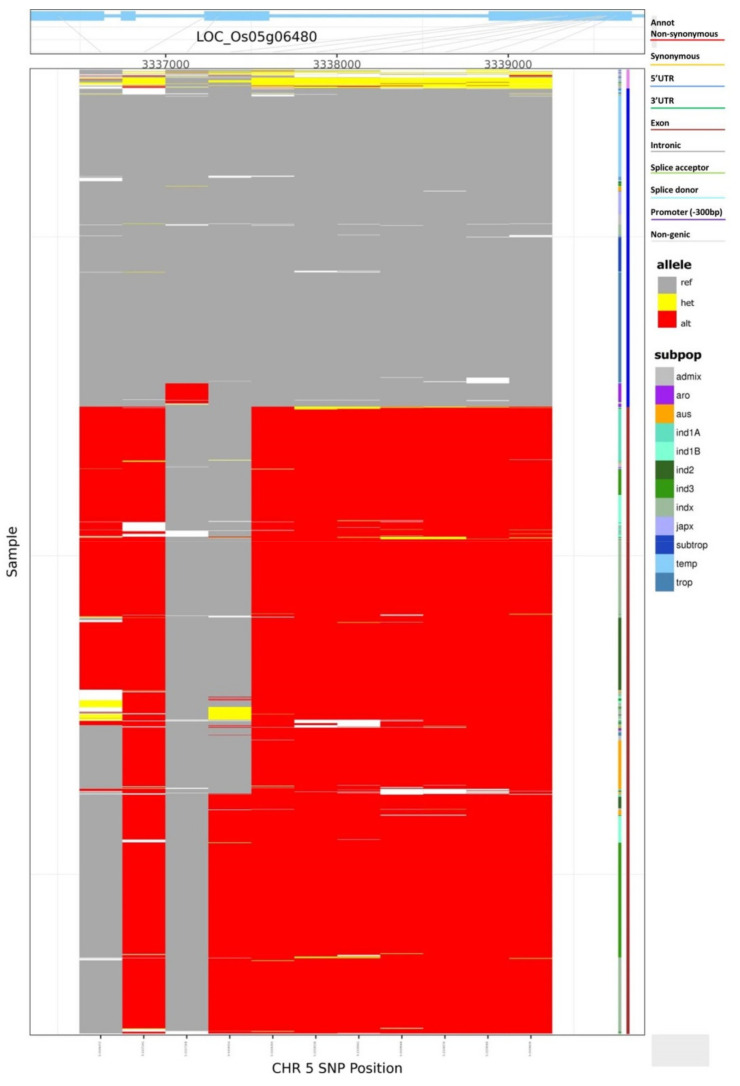
Haplotypic distribution of *chalk5 gene* across the ~3024 rice accessions depicted based on the sequence variation data retrieved from SNPseek database (https://snp-seek.irri.org/) (accessed on 15 September 2021). The topmost box represents the gene structure and sequence variations for gene *chalk5*. Bigger blue sections denote exons and the bars represent intronic regions. Red lines show 11 non-synonymous SNPs.

The highest numbers of InDel (607) were observed in gene *SUG1*, which is involved in the process of seed starch biosynthesis. Similarly, gene *Kala4*, which is responsible for grain pericarp color in rice, showed 448 InDels (Supplementary Table S1). In the case of the *chalk5 gene*, which regulates grain chalkiness, a total of 212 SNPs and 19 missense mutations were observed, and most of the missense mutations were present in exon 4. The representative haplotype groups among 3024 rice accessions for the *chalk5 gene* were specific to rice sub-populations. The indica types have reference type alleles and mostly belonged to the same haplotypic group (Figure 1). The tropical and temperate rice types mostly possessed alternate alleles and were grouped in distinct haplotypic groups when compared to the indica type. Similarly, the aromatic group showed mutations in the third exon specifically and were grouped separately compared to other rice sub-populations (Figure 1). In the case of the grain length *GL7* gene, a total of 10 missense mutations and ten haplotypes were observed with the maximum number of missense mutations in the third exon (Supplementary Figure S1A,B). Most of the accessions belonged to the haplotype Hap-III (1667) and only 13 rice accessions belonged to the Hap-X group (Supplementary Figure S1B,C). 

**Figure 2:**
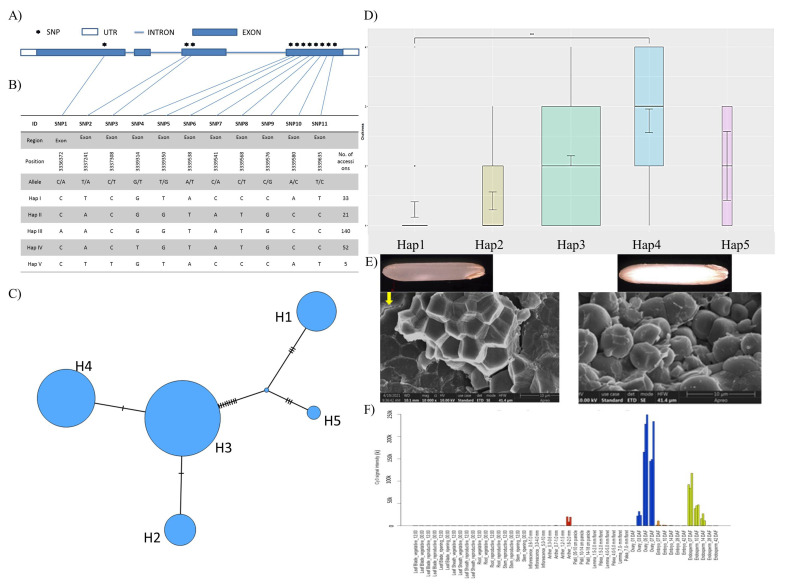
Representative illustration showing genetic variations observed in *chalk5 gene* defining haplotypic diversity, evolution, and expression profile in different tissues. (**A**) Intron–exon organization of *chalk5 gene* showing the non-synonymous single nucleotide polymorphism sites; (**B**) haplotypic grouping based on the SNPs present in the *chalk5 gene*; (**C**) haplotypic network showing relatedness and allelic evolution of *chalk5 gene*; (**D**) box plot showing the frequency distribution of haplotypes and their association with translucent and chalky phenotype, *** significance level at *p*-value < 0.001; (**E**) field emission scanning electron microscopy for chalky and translucent grains; and (**F**) expression profiling of *chalk5 gene* in different tissues.

The rice accessions belonging to different haplotypic groups showed varying gradations of grain chalkiness and translucence. A total of 19 missense mutations and 11 haplotypes were observed for the *chalk5 gene* (Table 1). After removing the missing data and heterozygous calls, five major haplotypes of the *chalk5 gene* were found most promising. These five haplotypes included distinctly differentiated chalkiness and translucent accessions (Figure 2A,B). The haplotypic network for the chalk 5 gene revealed five major haplotypes and large number of accessions belonged to haplotype group 3 (AACGGTATGCC) (Figure 2C). Among the five haplotypic groups, haplotype 1 (CTCGTACCCAT) and 2 (CACGGTATGCC) were most pronounced for translucent phenotype whereas haplotype 4 (CACTGTATGCC) was found to be associated with rice grains with varying degrees of chalkiness (Figure 2D). Most of the accessions in haplotype groups 1, 2, and 4 belonged to japonica, aromatic, and indica subpopulations, respectively. Field emission scanning electron microscopy (FESEM) for chalky and translucent grains revealed contrasting structures of starch granules (Figure 2E). The translucent rice had distinct polyhedral structures with no air pockets nearby and the chalky rice grains show round starch granules with neighboring air gaps (Figure 2E). It was also observed that starch granules in chalky grains had varying sizes. Gene expression dynamics for *chalk5* showed a higher gene expression in the ovary and endosperm tissues as compared to leaf, root, stem, and other tissues (Figure 2F). 

### 3.2. Haplotype Network Defining the Evolution of Important Rice Genes

Haplotypes found in at least ten accessions were considered for the development of haplotype networks (Supplementary Dataset S1). Based on the sequence variations prevalent in these genes, a total of 791 haplotyping groups were formed across the 80 genes (Supplementary Table S1). A maximum number of haplotypes (23) was observed in the *OsABCC1|MRP1* gene, which is an ABC transporter and reduces arsenic uptake (Supplementary Dataset S1). The lowest frequency was found in *OsHAC1;1* gene (4), which is known to regulate arsenic accumulation in rice (Supplementary Table S1). The *DU3* and *BADH2* genes showed 21 haplotypes, each distributed across nine rice isozyme categories (Supplementary Dataset S1). Both the *OsABCC1|MRP1*, and *OsHAC1;1* genes showed the highest number of accessions belonging to TeJ group. In the case of the *GL7* gene, ten haplotypes were observed and most of the accessions belonged to the indica rice types (IndII, IndIII, and Ind_admix), whereas accessions belonging to IndI group were mostly present in haplotype 4 (Supplementary Figure S1D). Most of the accessions belonging to TrJ, TeJ, and Jap_admix were more prevalent in the haplotypic group 6 in the case of the *GL7* gene (Supplementary Figure S1D). In the case of the *DU3* gene, haplotype 1 possessed a larger number of accessions, which were mostly grouped under the TeJ group (519) followed by TrJ (370) (Supplementary Dataset S1). Similarly, for the *BADH2* gene, haplotype 2 had the highest number of accessions with the TeJ group (535) followed by TrJ (245) (Supplementary Table S1). Similarly, for gene *OsCAO1|PGL* (chlorophyllide a oxygenase), haplotype 1 had the highest frequency with a larger number of accessions belonging to the IndIII subgroup followed by TeJ (Supplementary Dataset S1). The haplotypic networks for all the 80 genes are given in Supplementary Dataset S1.

#### Diversity Analysis 

Most of the genes showed positive Tajima D values indicating a decrease in population size and/or balancing selection; however, seven genes showed negative values that indicates population size expansion and/or purifying selection (Supplementary Table S3). The genes that show purifying selection include *OsPT2* (phosphate transporter), which is responsible for selenite transport, *RINO1* (low phytate), *OsIRT1* (Iron and Zinc accumulators), *SPDT* (phosphorous accumulation), *OsVIT2* (Iron transporter), *OsCAO1*|*PGL* (leaf senescence, grain yield), and *Rab5a* (storage protein transporter).

**Figure 3:**
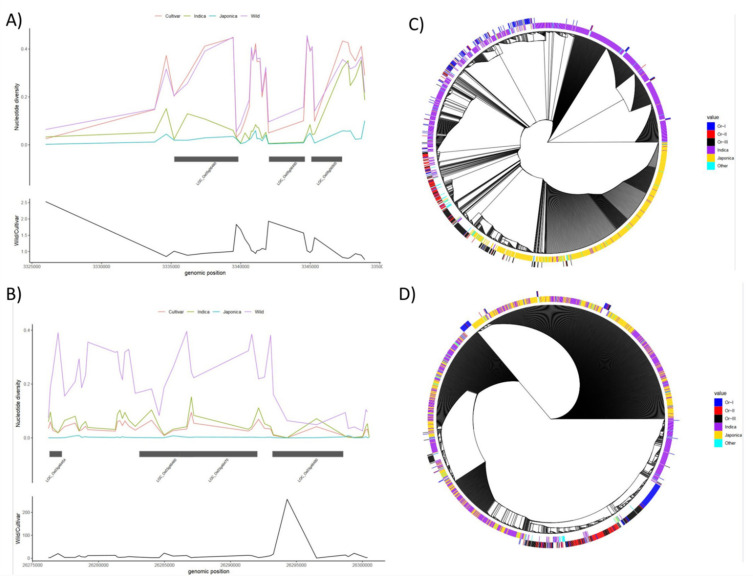
Nucleotide diversity and phylogenetic analysis for *chalk5* and *OsIRT1* genes. (**A**) Nucleotide diversity for the *chalk5* (*LOC_Os05g06480*) gene and (**B**) *OsIRT1* gene in cultivated (red), indica (green), japonica (blue), and wild (purple) rice varieties. The lower panel shows the ratio of nucleotide diversity between wild and cultivated rice accessions. Here, the *x*-axis represents the genomic positions and the *y*-axis denotes the nucleotide diversity values. (**C**,**D**) A neighbor-joining phylogenetic tree was implemented within the ECOGEMS resource for *chalk5* and *OsRT1* genes, respectively. Each edge of the circular tree represents a rice accession. The inner track represents the cultivated rice varieties whereas the outer track represents wild accessions. The *Oryza rufipogon* (Or) wild accessions are represented with blue (Or-I), red (Or-II), and black (Or-III) colors, whereas the cultivated Indica and Japonica accessions in purple and yellow colors, respectively.

The nucleotide diversity in the genomic region harboring nutritional and cooking quality-related genes was significantly reduced in cultivated rice compared to wild rice, particularly genes such as *OsCAO1|PGL*, *OsVIT2, and OsGZF1.* However, some genes like *OsABCC1|MRP1, OsMATE2, and SSIIIa* showed higher diversity in cultivated rice (Supplementary Dataset S2). The *chalk5 gene*, which governs the grain opaqueness/transparency showed similar nucleotide diversities for the cultivated and wild-type rice accessions (Figure 3A). However, significantly lower diversity values were observed for indica and japonica rice accessions. The comparative nucleotide diversity between for the *OsIRT1* gene, which governs the iron and zinc accumulation in rice shows a higher diversity in wild-type accessions when compared to the cultivated accessions (Figure 3B). It was noted that the diversity values for japonica sub-varieties were significantly decreased when compared to the cultivated and indica sub-types of accessions. The phylogenetic analysis for the *chalk5 gene* showed that the indica and japonica sub-groups grouped separately and most of the *Oryza rufipogon* OR-III accessions clustered with the japonica subtype and Or-I with the indica subtype (Figure 3C). The phylogenetic analysis for the *OsIRT1* gene shows a closer clustering of the indica and japonica sub-group and most of the *Oryza rufipogan* accessions were grouped separately and with more diversity than indica and japonica accessions (Figure 3D).

### 3.3. Gene Expression Dynamics for Grain Quality-Related Traits in Rice

**Figure 4:**
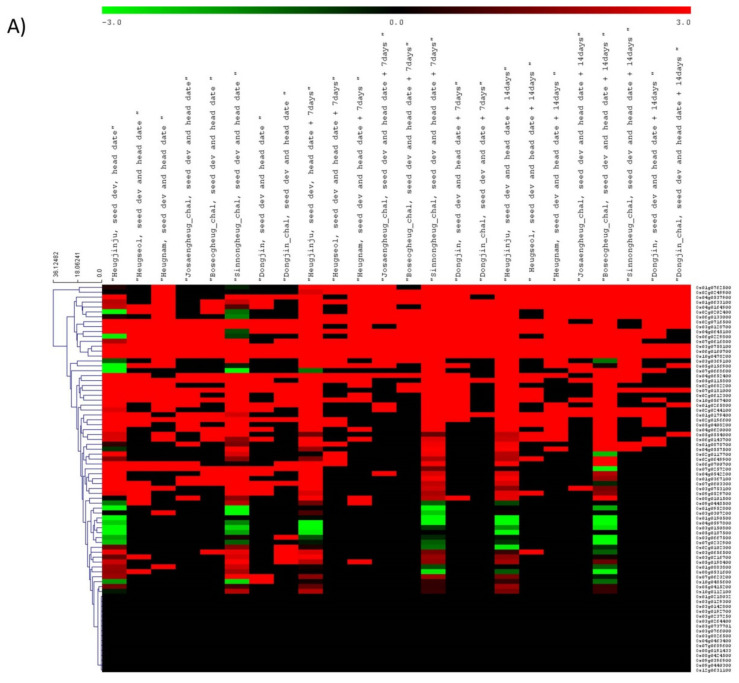

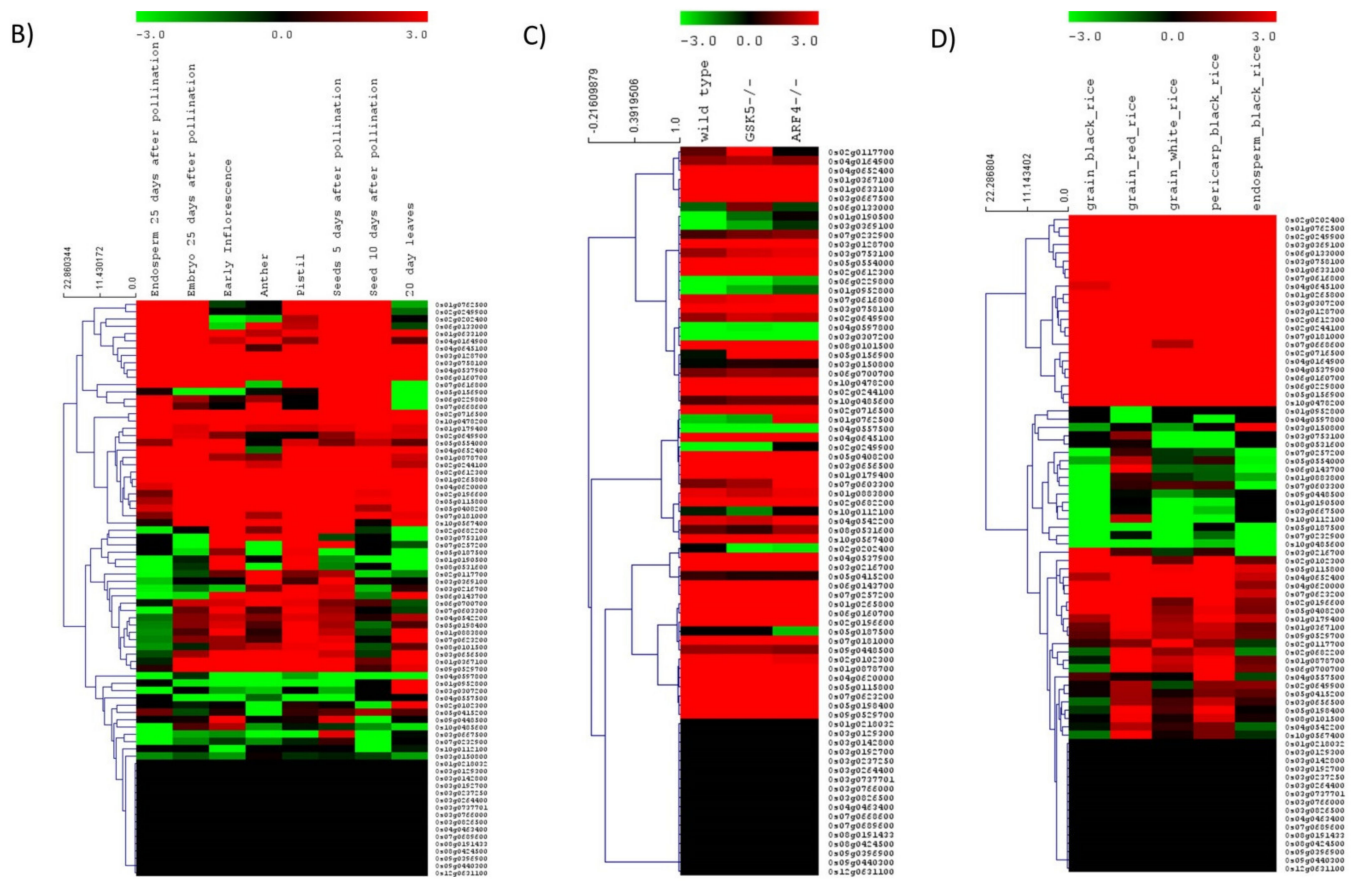
Heatmaps showing gene expression dynamics of 80 nutritional and cooking quality-related genes across different tissues and conditions. (**A**) Gene expression across seven rice varieties viz Heugjinju, Heuseol, Heugnam, Josengheug, Boseogheug, Sinnongheug, and Dongjin at different stages of seed development; (**B**) expression across different tissues such as endosperm, embryo, anther, pistil, seed, inflorescence, and leaves at different stages; (**C**) expression in wild type and loss of function mutants rice accessions for *GSK5* and *ARF4*; and (**D**) expression in grains, pericarp, and endosperm of white, black, and red rice. Red color corresponds to high expression whereas green for low gene expressions.

Expression analysis of grain quality-related genes was evaluated using publicly available transcriptomic data. A diverse expression profile across different tissues and developmental stages was observed for the quality-related genes in rice (Figure 4). Only 64 out of 80 genes showed expression in the selected tissues and developmental stages. Tissue-specific expression was observed in nutritional and cooking quality-related genes and about 29 genes showed higher expression in the endosperm (Supplementary Dataset S3). Clustering analysis showed tissue-specific and developmental stage-specific expression patterns for most of the genes. Some genes were constitutively expressed like *Rab5a* and *OsALDH7*, and a few genes were expressed only in the later stages of seed development such as *OsGZF1* (Figure 4A) Three genes, *GSE5*, *GW5L,* and *qGW8|OsSPL16|GW8*, were expressed only in early inflorescence and pistil tissue (Figure 4B). Three genes, *OsIRO2*, *OsNAS2,* and *OsHAC1;1* showed leaf-specific expression. Two genes, *OsUgp2|UGP2* and *WX* showed expression only in GSK5 mutants compared to wild type and ARF4 mutant. Two genes, *qCdT7|OsHMA3* and *chalk5*, were expressed in GSK5 and ARF4 mutants compared to wild type and *GLU4A* showed expression only in ARF4 mutant (Figure 4C). Gene *OsPht1;2* responsible for selenite uptake in rice showed higher gene expression in the endosperm tissue of black rice as compared to red and white rice. Similarly, the gene *SPDT* which governs phosphorus accumulation and *OsPCR1* showed significantly higher expression in grains of red rice as compared to white and black rice tissues (Figure 4D). Higher expression of the *GL7* gene was mostly observed in pistil followed by palea and lemma (Supplementary Figure S1E). The qPCR analysis showed higher expression of the *FLO16* gene in seedling and leaf as compared to the roots. In the case of *OsLTPL36* and *GLU4A* genes, comparatively higher expression was observed in roots (Supplementary Figure S2).

### 3.4. Hub Genes Identified through Gene Co-Expression Network Analysis in Rice

**Figure 5:**
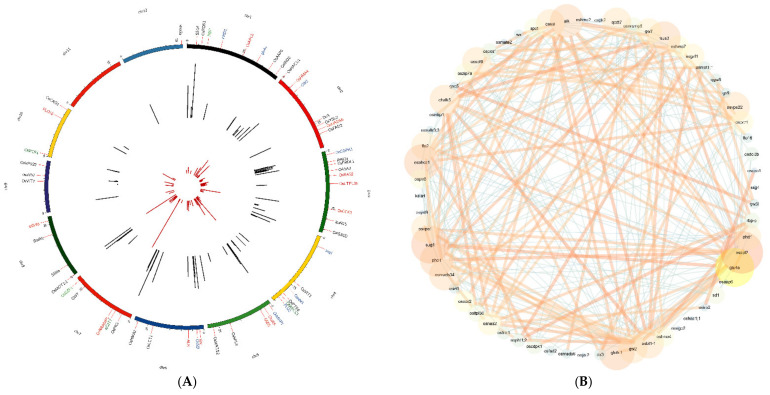
(**A**) Chromosomal distribution and cluster of genes as functional modules predicted by WGCNA. The outermost track number 1 shows the chromosomal position of 80 cooking and nutritional quality-related genes. Genes highlighted in red color belong to Module 1: Starch, Chalkiness, Zinc bioavailability, Sucrose; Module 2 (blue): Grain size, Starch, Seed storage protein, Glutelin; Module 3 (green): Cd, Zn, Glumes, Phosphorus, Arsenic, Heavy metals; Module 4 (black): Grain width, Size, Weight, Cadmium, Copper; Module 5 (orange): Iron, Phytate, Phosphorus; Module 6 (grey): Glutelin, Prolamine, Selenite. Track number 2 depicts the number of SNPs in various genes responsible for grain cooking and nutritional quality traits whereas the innermost track 3 represents the missense SNPs for these genes. (**B**) Co-expression network predicted among the 80 cooking and nutritional quality-related genes. The network was developed with Cytoscape v3.7.2.20 using RPKM values. Genes with the highest degree of co-expression have been displayed in bigger and brighter nodes and vice versa. Positive correlations have been depicted in orange, whereas negative correlations in blue edges.

A co-expression network for 80 genes was developed using gene expression data from 11,726 libraries from the ‘PPRD’ database. A total of 16 genes were removed after filtering the expression data, based on missing values using the WGCNA tool. The remaining 63 genes were used for co-expression network construction and module detection. Varying module sizes were selected to obtain an optimum number of modules. For network generation, the minimum number of genes to define a module was selected as 3. Among various modules, genes for grain protein content clustered separately, as did genes responsible for grain starch content and chalkiness (Figure 5A). Genes placed in module 0 were not grouped under any module.

A total of 19 hub genes showed a high degree of correlation with other genes (Table 2, Figure 5B). Among these, gene *OsCAO1*, which is involved in grain yield and quality, showed the highest degree of correlation with 20 other quality-related genes (Supplementary Table S4). The *OsSSI* gene involved in the starch biosynthesis pathway and *FLO2* which controls rice grain size and starch quality showed a high degree of correlation with 19 genes, respectively. The *OsSULTR3;3* gene showed 18 interactions with the rest of the genes. Among the hub genes, the *Ospho1* gene, which is responsible for starch structure within the endosperm, showed higher expression (1237.6 RPKM value) in seed (Table 2, Supplementary Table S4). The *Ospho1* gene was present in the WGCNA module 1, which consists of genes related to grain starch, sucrose and chalkiness (Figure 5A).

### 3.5. Interaction of Transcription Factors and Nutritional Quality-Related Genes 

A total of 1413 regulatory interactions were obtained between 211 transcription factors and 80 genes governing rice cooking and nutritional quality (Table 3). Among the total interactions, it was observed that 87 transcription factors possessed over-represented targets in the input gene set under the cutoff *p*-value ≤ 0.05. As a result, transcription factor LOC_Os05g03020 with target gene *OsGZF1* had the highest number of binding sites with 24. Similarly, transcription factor LOC_Os05g03020 belongs to the C2H2 family and interacts with gene OsHAC1;1. Likewise, transcription factor enrichment analysis revealed that LOC_Os03g60630 and LOC_Os07g13260 had the lowest *p*-values and most significant results in terms of over-represented targets in the input gene set under cutoff <= 0.05. Here, both the transcription factors belong to the Dof family. 

## 4. Discussion

The increasing human population has led to increased demands for high-calorie and nutrient-rich foods, which in turn have necessitated in-depth studies targeted at exploring the available genomic and transcriptomic resources for developing high-yielding crop-varieties. Several genes responsible for crop yield, abiotic and biotic stress resistance, grain quality, palatability, and cooking and nutritional quality-related traits have been characterized [42,43]. The development of novel varieties with improved traits is hindered by the lack of information about the sources of desirable alleles and the genetic background of the donor lines. The whole-genome re-sequencing data available for over 4500 diverse rice genotypes is an excellent resource for understanding the allelic diversity of well-characterized genes governing important traits in rice. In the present study, based on the analysis of 80 genes linked to nutritional and cooking quality traits, 133 deleterious mutations within 39 genes were identified. The rest of the genes either did not show any sequence variations or showed polymorphisms that were not functionally important for the selected traits. Based on these sequence variations, haplotype analysis was conducted to deduce the patterns of inheritance for the effective selection of informative SNPs. Thirty-nine genes showed more than or equal to 10 haplotypes. Among these, the highest number of haplotypes was observed in *OsABCC1|MRP1*, which is an ABC transporter followed by two genes, *DU3* and *BADH2*, which are involved in starch content regulation and aroma, respectively. *OsHAC1;1* showed the least number of haplotypes (4) and is involved in the regulation of arsenic accumulation. Similarly, four haplotypes were observed for *OsLCT,* which regulates cadmium accumulation in rice. In addition to this, haplotype networks were generated to study the evolution of important haplotypes across nine rice isozyme classification groups. To understand the expression of these genes across selected rice tissues and different developmental stages, transcriptome data from 102 experiments in four studies on rice have been analyzed. In terms of gene co-expression, we could finalize a set of 19 genes with the highest gene co-expressions and these were termed as ‘hotspot’ genes. The hotspot genes depict both a strong positive as well as a negative correlation with the rest of the genes. In addition, we have predicted the genetic regulation and a set of 87 transcription factors that were over-represented in the dataset. The transcription factors were predicted to regulate the expression of 78 genes related to nutritional and cooking quality-related traits. Gene sequence polymorphism, expression dynamics, and regulation provide a multi-tiered approach in the selection of SNPs for crop improvement studies. Recently, Angira et al. [44] identified two haplotypes of the *SD1* gene, which is commonly observed in the rice germplasm of the United States. Angira, Addison, Cerioli, Rebong, Wang, Pumplin, Ham, Oard, Linscombe and Famoso [44] first identified six SNPs that could differentiate all seven haplotypes present in the *SD1* gene. Subsequent haplotype-based marker development and screening of the rice germplasm of the US revealed that the first and the third haplotypes are predominant. In the present study, 66 SNPs, nine missense mutations, and 11 haplotypes including seven reported by Angira, Addison, Cerioli, Rebong, Wang, Pumplin, Ham, Oard, Linscombe and Famoso [44] for the *SD1* gene were identified (Supplementary Table S1). Previously, seven haplotypes were identified for the gene *chalk5* on chromosome 5, based on sequence variations across a panel of 191 rice accessions of indica and japonica type [45]. The haplotype groups were categorized into two sub-groups: class A (hap 1–4) and class B (hap 5–7), based on phylogenetic analysis. Class A haplotype groups represented accessions with white belly chalkiness trait. In our study, we have identified 11 haplotype groups based on 19 missense mutations within the *chalk5 gene* across indica, japonica, aus and intermediate subgroups. Our analysis revealed five promising haplotypes for chalkiness trait based on the 11 nonsynonymous SNPs after excluding heterozygous and missing SNPs in the haplotype groups. Among the five haplotypes, haplotypes 1 and 2 were associated with transparent rice grains, whereas haplotype 4 was associated with chalkiness of seeds. The present study demonstrated haplotype and phenotype association for the chalkiness traits, which can be explored further for haplotype-based breeding. Another example where haplotypic information was used to identify the desired allele for the *GS9* gene is reported by Zhao et al. (2018). Here, a set of 114 diverse rice genotypes were used to identify five SNPs in *GS9* which categorizes the set in five haplotypes. The null mutation in the *GS9* gene was found to improve grain shape to a slender form whereas its overexpression resulted in round grains. However, 75 SNPs, including the eight nonsynonymous SNPs identified herein for *GS9* provide an opportunity to identify novel alleles with the desired effects and consequently more genetic resources (donor lines) for breeding (Supplementary Table S1). Several studies similar to that of Zhao et al. [46] have explored of genetic variations to identify desired alleles and to understand haplotypic variation; however, those are mostly focused on a single gene (Supplementary Table S5). Notably, in the present study, haplotypic analysis of 80 genes regulating nutritional and cooking quality-related traits provides numerous advantages, including an understanding of allelic variability and evolution, the identification of desired alleles and their sources simultaneously for different genes, and providing an opportunity for haplotype-based precision breeding. 

The non-synonymous SNPs identified in the candidate genes were further evaluated with PROVEAN, which helped to associate the functional prediction to the haplotypes. The probable functional impact of non-synonymous SNPs is higher than that of synonymous SNPs. In this regard, PROVEAN scores were calculated based on the sequence level conservation and properties of the amino acids, which have helped to predict the effect of non-synonymous SNPs. An earlier study performed by Deshmukh et al. [47] has demonstrated the use of PROVEAN to accurately predict the effect of amino acid change. They have used site-directed mutagenesis to verify the deleterious and neutral effects predicted for the silicon transporter genes from tomato, rice, and poplar [47]. Similarly, desired alleles for negative regulators can be efficiently identified based on the PROVEAN score. 

In addition to genomic information, the present study has explored extensive transcriptomic data. The transcriptomic information provided here for the important genes related to nutritional and cooking quality in rice will be helpful to better understand gene regulation. Many of these genes might have pleiotropic effects which regulate other important traits. The *OsFAD2|OsFAD2-1* gene, which affects lipid accumulation, has high expression in 20-day-old leaves. Moreover, the gene *OsMADS34|PAP2*, which regulates grain yield and quality, shows high gene expression in anther. Similarly, a co-expression network developed here for the nutritional and cooking quality-related genes helped to identify hub genes. The exploration of haplotype diversity for such genes could provide a relatively high level of variation in the grain quality traits for future crop breeding and improvement programs. In addition, such a network will be helpful for understanding the interactions among the selected genes. Recently, a gene co-expression network has been successfully used to identify substructures of gene modules responding to salt stress in rice [48]. Similarly, a co-expression network developed for strawberry has been used to identify genes regulating flower and fruit-related traits [49]. Additionally, a co-expression network developed in rice identified modules associated with temperature-inducible and photoperiod sensitive genes, which are important for the sterility transition [50]. Similarly, 496 hub genes and four modules showed a significant correlation with photo-sensitive differentially expressed genes in rice [51]. Furthermore, weighted gene co-expression analysis has identified differentially expressed genes such as *OsHSPs, OsHSFC2A,* and *OsDJA5* upon cadmium treatment in stem nodes of different rice genotypes [52]. Many of such examples utilizing co-expression network information suggest the potential applications of the network developed in the present study for the rice quality-related genes. 

## 5. Conclusions

In this study, we aimed to understand the genetic variations, identify haplotypes, and analyze the expression patterns of genes known to have a significant role in rice nutritional and cooking quality-related traits. We have detailed the functionally important SNPs in 80 previously cloned genes known to regulate grain quality-related traits. The predicted functional impact of the sequence variants will help to understand gene regulation as well as the effect of haplotype on trait development. The generated resource will serve as a basis for haplotype-based breeding programs of rice. The detailed haplotypic information provided here will facilitate the identification of donor lines harboring the most desired haplotype. In addition, starting with 80 functionally characterized genes, we have narrowed down a list of 19 hotspot genes based on the co-expression network developed using extensive transcriptomic data. The information of hotspot genes can be further explored to understand the gene interaction and interdependency of grain quality-related traits. Subsequently, the efficacy of the approach was demonstrated by showing haplotypic association with key nutritional and cooking quality traits like grain length, grain weight, and chalkiness. The adopted approach and the information provided in the present study will be helpful for understanding genetic variation for the nutritional and cooking quality in rice and for accelerating haplotype-based breeding programs aimed at customizing high-quality rice with the desired nutritional value.

## Figures and Tables

**Table 1:** Details of single nucleotide polymorphisms (SNP) predicted to have a deleterious impact on the functionality of genes known to regulate the nutritional and cooking quality related traits in rice.

Gene Name	RAP ID	Feature	Total SNPs	Number of Haplotypes	Number of Missense Mutations	Functional Impact of Missense Mutations	Number of InDels
*GW5L*	*Os01g0190500*	Grain weight	82	12	3	V58G, A70S, H235R*, V58A	70
*PHD1*	*Os01g0367100*	Galactolipid biosynthesis	72	12	6	D63V, F335I*, S321C*, G305V*, D288N*, F273V*	186
*GLUa*	*Os01g0762500*	Glutelin content	29	8	8	Y5H*	7
*SD1| GA20ox2*	*Os01g0883800*	Grain protein content	66	11	9	A82N*, A82N*, E100G, H101D*, H101D*, C193S*, P240L*, L266F*, Q340R, D349H	105
*OsHMA4*	*Os02g0196600*	Copper accumulation	47	12	8	I55M, F303L, T316M, A553V, S660A, I704M, G818S, V914A*	64
*OsBT1-1|OsBT1*	*Os02g0202400*	Endosperm granule formation	65	9	17	V25A, C46R, R102K, M107I, S115A, H127Q, R130Q, R152C*, R170H, M173I, R183H*, G184D*, T205R*, Y209C, P217L, V224I, Y209F	70
*DU3*	*Os02g0612300*	Grain amylose content	107	21	7	S96F*, Y237F, R189K	116
*OsCDPK1|* *OsCDPK13|* *OsCDPK11|* *OsCPK11|* *OsCDPK12*	*Os03g0128700*	Grain starch structure	67	8	8	I143T*, E259Q*, A265S*, I268V*, Q284P, F288Y*, R355W*, V474I	54
*XS-LPA2-1*	*Os03g0142800*	Seed phytic acid	41	10	6	N6T, A19T, R32L, A350T, N645K*, L1469F*	28
*OsPht1;2|OsPT2*	*Os03g0150800*	Selenite uptake	20	9	5	H398R, N335D, P269S, S258C*, V185I	6
*OASA2*	*Os03g0264400*	Grain tryptophan content	78	17	6	E585D, G527R*, P446S, R303P, E79K, R68P	85
*OsCCX2*	*Os03g0656500*	Grain cadmium content	61	7	5	H90N, D292N, F412V*, V448L*, L532V	12
*OsIRT1*	*Os03g0667500*	Grain iron and zinc content	243	5	20	V369I, R307K, R304K, R281K, I227M, V213A, R189W, H180R, V174I, N122S, N121S, G93R, L82F, V71A, A58V*, D49E, I29L, I27F, L21V, P9A	217
*OsMADS34|PAP2*	*Os03g0753100*	Grain yield	90	8	5	Q89H*, Q106K*, Q71K*, T20A*, R10P*	140
*OsPho1*	*Os03g0758100*	Starch structure in endosperm	33	9	11	T268N*, V165I, E153K, R550H*, R501C*, P391S*, T268N*, I254F*, S203L*, L60F*, M1del*	37
*OsVIT1*	*Os04g0463400*	Iron translocation	72	10	5	A170T*, V136A*, Q105K	70
*OsVPE1*	*Os04g0537900*	Seed glutelin	33	5	3	E384G*, Q90R, Y86C*	27
*OsYSL9*	*Os04g0542200*	Iron distribution	48	8	4	S511N, T368R, L256F, R90L*	22
*Kala4|* *OsS1*	*Os04g0557500*	Grain pericarp colour	1140	14	6	E308D, D173N, L140V, D101H, P84L, A29V*	448
*OsABCC1|* *MRP1*	*Os04g0620000*	Arsenic accumulation	245	23	40	S1468T, R1398G*, R1300Q, E1231V*, R990Q, L933F, K892R, Q879L, V814L, R712H*, P642H*, A524S, R518C*, A449V, L285I, N283S, R276Q, T216S, P206L*, L176V, A156S, I150S, I134M, A107V, A92V, R90Q, T61A, G47S, T33S, N23Y*, V21L, S1468N, F708L*, A409T*, R383H*, S266I*, F252T*, A233V*, C228F*, C83F*, T29A*	135
*FLO2*	*Os04g0645100*	Grain size	117	9	30	A274T, L399P, I466T, R579K, P599L, G804D, S1203L, N1319D, A1608T, F195L*, H200N*, P204T*, S306P*, N323D*, M348R*, M348I*, L369F*, A378S*, G452S*, P515T*, W589C*, R725K*, A748V*, A789S*, N829Y*, R892Q*, G926C*, R987H*, A1060V*, L1107F*, Y1146F*	217
*chalk5*	*Os05g0156900*	Chalkiness	212	11	19	R525L, I497V, V412I, K401M, A379T, A364V, G237R*, A215V, A139G, R137S, T125P, V62D, G61V, G59V, D58G, S52N, E49D, V48G, M30V	143
*OsSPL9*	*Os05g0408200*	Grain copper accumulation	79	8	7	A132V, A200T, P309S, I576V, S751T, I789V, S248F*	114
*WX*	*Os06g0133000*	Grain characteristics	137	14	4	D166G*, Y224S*, P415S, D528Y*, D528N	205
*SPDT*	*Os06g0143700*	Phosphorus accumulation	281	7	6	Q385L*, I251V, L247F, V71G*, A47V, A21V	308
*OsSSI*	*Os06g0160700*	Grain starch content	197	10	12	S596L, K438E*, H420Y*, S319G, D214N*, L86F, A78S, T74A, L60M, R29L, R343S*, C251Y*	138
*ALK|SSIIa*	*Os06g0229800*	Grain starch quality	98	16	9	P56A, T117P, A148S, D161E, E208D, D283E, S604G, M737V, L781F*	65
*OsLCT1*	*LOC_Os06g38120*	Cadmium in grains	236	4	31	E15D, D26A, E35Q, P43L, P48S, A54S, I60T, L67H, L70Q, A71D, G73D, A77T, A80S, N84K, E87K, V95I, L101F, T147S, R152S, V183A, V211L, L215F, K223N, M241V, Q246H, E258L, M310V, L380F, T480S, V494M, L495F*	218
*OsHMA2|OsHMA2v*	*Os06g0700700*	Grain zinc and cadmium content	104	8	3	C19R*, R7W	261
*qCdT7|* *OsHMA3*	*Os07g0232900*	Grain zinc content	142	10	56	G990A, E975D, C960G, T953I, G930R, D926G, K912R, A908G, A908T, S873G, G787S, E775D, G770A, D768A, A759P, A758S, V752A, E733K, C678R, A638V, S614D, S614D, G595A, G594S*, S575T, D556H, V550I, T526I, S525T, R493Q, G490A, A381V*, S380R*, D338N*, T333M*, F299L, N298I*, Q269R, G268S*, V259I, E257K*, G256D*, G256S*, V250I, E238D, A234V, I233L, V229A, A184S, T134M, G130S*, A95V, E93A, P92S, A91T, R80H*, S614G, P92T, N725H*, L708F*, V697A*, N686K*, G642D*, E607A*, A341T*, V323G*, W293C*, P283L*, D267G*, D262N*, A252V*, R163C*, A99V*, D87N*, V82A*	50
*GW7|GL7|SLG7*	*Os07g0603300*	Grain quality and yield	60	10	10	I915M, S647A, S620G, N605K, P518S, A462S, R361H, R361C, L259F*	75
*RSUS3|* *SUS3*	*Os07g0616800*	Grain starch content	45	12	7	A26T, E541K*, L551S, S559N, N634D, E637K, S15G*	47
*OsHMA7*	*Os07g0623200*	Grain iron and zinc content	75	10	4	A32V, C37R, L147V*, R159C	5
*OsGZF1*	*Os07g0668600*	Seed storage protein	26	7	9	R255H, A219S, S179P, M174I, L169V, A111P, A102V, R100H, E47K*, A111T	12
*SSIIIa*	*Os08g0191433*	Endosperm appearance	186	14	35	A33T, M38K, T43N, A62S, R109H, K116N, E142D, A184T, A195V, A217T, G226E, E231K, A268V, S350L, F401S, D427G, V480L, T486I, A503T, R576K, E641V, S681N, G686E, R702Q, R748H, E790V, G817D, V843E, L957M, Y964C*, K1006N*, R1118K, R1240H, A1528S, T1755I	136
*BADH2*	*Os08g0424500*	Grain aroma	156	21	5	A190V, K244I*, A316E, P458S*, G468V*	123
*qGW8|* *OsSPL16|GW8*	*Os08g0531600*	Grain quality and shape	69	10	8	P79L, A110V, D172N*, T274N, Q285K, G315S, M364I, A397T	222
*OsDCL3b*	*Os10g0485600*	Seed quality	59	11	14	H89L*, A115V*, P129S*, N149D, S155A, T181A, F205L, I355M, G712S, R811S, V874L, R1063Q, F1101V, I1320L	135
*OsCAO1|* *PGL*	*Os10g0567400*	Grain yield and quality	28	5	4	L394F*, T181P, V35L	39

Gene names are italicized. * indicates deleterious mutations.

**Table 2:** Details of hub genes identified through weighted correlation network analysis (WGCNA) performed with transcriptome profiling of genes known to regulate nutritional and cooking quality related traits in rice.

Gene Name	Gene ID	Neighborhood Connectivity	Clustering Coefficient	Number of Directed Edges	WGCNA Module	WGCNA Module Description	Tissue Expression	Normalized Expression Value (RPKM)
*OsCAO1*	*Os10g0567400*	9.80	0.24	20	5	Iron, Phytate, Phosphorus	Seedling	249.5
*OsSSI*	*Os06g0160700*	11.84	0.39	19	2	Grain size, Starch, Seed storage protein, Glutelin	Endsoperm	397.9
*FLO2*	*Os04g0645100*	10.16	0.33	19	2	Grain size, Starch, Seed storage protein, Glutelin	Developing seed	491.1
*OsSULTR3;3*	*Os04g0652400*	10.67	0.39	18	5	Iron, Phytate, Phosphorus	Seedling	149.3
*OsGAPDHB*	*Os03g0129300*	8.88	0.13	17	0	NA	Seedling and shoot	672.4
*OsPTR6*	*Os04g0597800*	12.63	0.48	16	0	NA	Seedling	57.5
*OsFRDL1*	*Os03g0216700*	12.64	0.48	14	0	NA	Endosperm	86.7
*SSIIIa*	*Os08g0191433*	10.31	0.54	13	0	NA	Endosperm	363.9
*OsNRAMP5*	*Os07g0257200*	13.92	0.67	13	4	Grain width, size, weight, Cadmium, Copper,	Caryopsis	136.4
*GW2*	*Os02g0244100*	12.54	0.56	13	2	Grain size, Starch, Seed storage protein, Glutelin	Seed	492.1
*OsGZF1*	*Os07g0668600*	10.67	0.68	12	6	Glutelin, Prolamine, Selenite	Seed	264.4
*RSUS3*	*Os07g0616800*	10.67	0.68	12	1	Starch, Chalkiness, Zinc bioavailability, Sucrose	Endosperm	665.1
*qCdT7*	*Os07g0232900*	14.42	0.74	12	3	Cd, Zn, Glumes, Phosphorus, Arsenic, Heavy metals,	Seedling	47.9
*ALK*	*Os06g0229800*	10.67	0.68	12	1	Starch, Chalkiness, Zinc bioavailability, Sucrose	Seed	444.2
*Ospho1*	*Os03g0758100*	11.92	0.64	12	1	Starch, Chalkiness, Zinc bioavailability, Sucrose	Grain	1237.6
*OsHMA4*	*Os02g0196600*	14.25	0.61	12	4	Grain width, size, weight, Cadmium, Copper,	Seed	127.9
*Osvpe1*	*Os04g0537900*	11.45	0.64	11	2	Grain size, Starch, Seed Storage protein, Glutelin	Seed	370.6
*OASA1D*	*Os03g0826500*	12.80	0.58	10	0	NA	Seed	246.5
*OsAPL2*	*Os01g0633100*	11.60	0.80	10	1	Starch, Chalkiness, Zinc bioavailability, Sucrose	Developing seed	1138.6

**Table 3:** Details of transcription factors predicted to have interaction with cooking and nutritional quality-related genes. Plant Transcriptional Regulatory Map (PlantRegMap) server [40] was used to predict the interaction.

TF	Common Name	TF Family	Query_All ^#^	Query_Bind ^$^	*p*-Value ^¥^	q-Value
*LOC_Os03g60630*	*OJ1754_E06.26*	Dof	80	33	3.18 × 10^−6^	3.75 × 10^−4^
*LOC_Os07g13260*	*Os07g0236700*	Dof	80	37	3.56 × 10^−6^	3.75 × 10^−4^
*LOC_Os01g53220*	*Os01g0733200*	HSF	80	6	5.58 × 10^−5^	3.93 × 10^−3^
*LOC_Os02g41510*	*Os02g0624300*	MYB	80	11	2.28 × 10^−4^	8.59 × 10^−3^
*LOC_Os04g43680*	*Os04g0517100*	MYB	80	11	2.28 × 10^−4^	8.59 × 10^−3^
*LOC_Os12g39400*	*Os12g0583700*	C2H2	80	6	2.75 × 10^−4^	8.59 × 10^−3^
*LOC_Os02g47810*	*OsJ_35953*	Dof	80	26	2.85 × 10^−4^	8.59 × 10^−3^
*LOC_Os11g29870*	*Os11g0490900*	WRKY	80	6	4.24 × 10^−4^	1.12 × 10^−2^
*LOC_Os05g09020*	*Os05g0183100*	WRKY	80	8	4.79 × 10^−4^	1.12 × 10^−2^
*LOC_Os04g50770*	*Os04g0594100*	MYB	80	9	7.53 × 10^−4^	1.26 × 10^−2^

^#^ ‘Query_all’ stand for the number of gene promoters that were examined for the existence of transcription factor binding sites; ^$^ ‘Query_bind’ represents the number of genes with a binding site for a specific transcription factor in their promoter; ^¥^ *p*-value cutoffs of ≤0.05 was used to claim significant interaction.

## Data Availability

Not applicable.

## Supplementary Materials

The following supporting information can be downloaded at: https://www.mdpi.com/article/10.3390/cells11071144/s1, Table S1. Details of Single nucleotide polymorphism (SNPs), Insertions/deletions (InDels), and their haplotyping information for nutritional and cooking quality related genes. The deleterious effect mutations are represented with an asterisk; Table S2. Details of RNASeq datasets used in the present study. All the raw data was retrieved from NCBI SRA database and analyzed using CLC Genomics workbench; Table S3: Diversity analysis of 80 nutritional and cooking quality-related genes in terms of average pairwise divergence (π), estimated mutation rate (θ) and Tajima’s D values; Table S4. Details of hub genes and their interactions identified by co-expression network using RNAseq data; Table S5. Details of genetic variations previously reported for nutritional and cooking quality related genes; Figure S1: Representative illustration showing genetic variations observed in GL7 gene defining haplotypic diversity, evolution, and expression profile in different tissues. (A) Intron-exon organization of GL7 gene showing single nucleotide polymorphism sites; (B) haplotypic grouping based on the SNPs present in the GL7 gene; (C) Violin plot showing the frequency distribution of haplotypes; (D) haplotypic network showing relatedness and allelic evolution of GL7 gene; (E) expression profiling of GL7 gene in different tissues; Figure S2. The relative expression level of FLO16, OsLTPL36, and glu4a in root, leaf, and seedling, measured with real-time quantitative PCR (RT-qPCR). Bars show mean log(2) fold change ± SEM of three biological samples; Supplementary Dataset S1. Haplotypic network of 80 nutritional and cooking quality related genes depicting number of haplotypes and their interactions with different rice types; Supplementary Dataset S2. Diversity analysis between cultivated and wild rice accessions for 80 nutritional and cooking quality related genes; Supplementary Dataset S3. Expression in different rice tissues among three cultivars for 80 nutritional and cooking quality related genes. The genes highlighted have specific expression in rice endosperm.

## References

[1] Rana N., Rahim M.S., Kaur G., Bansal R., Kumawat S., Roy J., Deshmukh R., Sonah H., Sharma T.R. (2020). Applications and challenges for efficient exploration of omics interventions for the enhancement of nutritional quality in rice (*Oryza sativa* L.). Crit. Rev. Food Sci. Nutr..

[2] Awika J.M. (2011). Major cereal grains production and use around the world. Advances in Cereal Science: Implications to Food Processing and Health Promotion.

[3] Das P., Adak S., Majumder A.L. (2020). Genetic manipulation for improved nutritional quality in rice. Front. Genet..

[4] Kasote D., Sreenivasulu N., Acuin C., Regina A. (2021). Enhancing health benefits of milled rice: Current status and future perspectives. Crit. Rev. Food Sci. Nutr..

[5] Hedden P. (2003). The genes of the Green Revolution. Trends Genet..

[6] Li N., Xu R., Duan P., Li Y. (2018). Control of grain size in rice. Plant Reprod..

[7] Wang S., Wu K., Yuan Q., Liu X., Liu Z., Lin X., Zeng R., Zhu H., Dong G., Qian Q. (2012). Control of grain size, shape and quality by OsSPL16 in rice. Nat. Genet..

[8] Si L., Chen J., Huang X., Gong H., Luo J., Hou Q., Zhou T., Lu T., Zhu J., Shangguan Y. (2016). OsSPL13 controls grain size in cultivated rice. Nat. Genet..

[9] Che R., Tong H., Shi B., Liu Y., Fang S., Liu D., Xiao Y., Hu B., Liu L., Wang H. (2015). Control of grain size and rice yield by GL2-mediated brassinosteroid responses. Nat. Plants.

[10] Wang Y., Xiong G., Hu J., Jiang L., Yu H., Xu J., Fang Y., Zeng L., Xu E., Xu J. (2015). Copy number variation at the GL7 locus contributes to grain size diversity in rice. Nat. Genet..

[11] Kashiwagi T., Munakata J. (2018). Identification and characteristics of quantitative trait locus for grain protein content, TGP12, in rice (*Oryza sativa* L.). Euphytica.

[12] Peng B., Kong H., Li Y., Wang L., Zhong M., Sun L., Gao G., Zhang Q., Luo L., Wang G. (2014). OsAAP6 functions as an important regulator of grain protein content and nutritional quality in rice. Nat. Commun..

[13] Yang Y., Guo M., Sun S., Zou Y., Yin S., Liu Y., Tang S., Gu M., Yang Z., Yan C. (2019). Natural variation of OsGluA2 is involved in grain protein content regulation in rice. Nat. Commun..

[14] 3000 Rice Genomes Project (2014). The 3000 rice genomes project. GigaScience.

[15] Lachagari V., Gupta R., Lekkala S.P., Mahadevan L., Kuriakose B., Chakravartty N., Mohan Katta A., Santhosh S., Reddy A.R., Thomas G. (2019). Whole genome sequencing and comparative genomic analysis reveal allelic variations unique to a purple colored rice landrace (*Oryza sativa* ssp. indica cv. Purpleputtu). Front. Plant Sci..

[16] Zhao H., Yao W., Ouyang Y., Yang W., Wang G., Lian X., Xing Y., Chen L., Xie W. (2015). RiceVarMap: A comprehensive database of rice genomic variations. Nucleic Acids Res..

[17] Huang X., Zhao Y., Wei X., Li C., Wang A., Zhao Q., Li W., Guo Y., Deng L., Zhu C. (2012). Genome-wide association study of flowering time and grain yield traits in a worldwide collection of rice germplasm. Nat. Genet..

[18] Chen W., Gao Y., Xie W., Gong L., Lu K., Wang W., Li Y., Liu X., Zhang H., Dong H. (2014). Genome-wide association analyses provide genetic and biochemical insights into natural variation in rice metabolism. Nat. Genet..

[19] Xu X., Liu X., Ge S., Jensen J.D., Hu F., Dong Y., Gutenkunst R.N., Fang L., Huang L., Li J. (2012). Resequencing 50 accessions of cultivated and wild rice yields markers for identifying agronomically important genes. Nat. Biotechnol..

[20] Qin P., Lu H., Du H., Wang H., Chen W., Chen Z., He Q., Ou S., Zhang H., Li X. (2021). Pan-genome analysis of 33 genetically diverse rice accessions reveals hidden genomic variations. Cell.

[21] Wei X., Qiu J., Yong K., Fan J., Zhang Q., Hua H., Liu J., Wang Q., Olsen K.M., Han B. (2021). A quantitative genomics map of rice provides genetic insights and guides breeding. Nat. Genet..

[22] Abbai R., Singh V.K., Nachimuthu V.V., Sinha P., Selvaraj R., Vipparla A.K., Singh A.K., Singh U.M., Varshney R.K., Kumar A. (2019). Haplotype analysis of key genes governing grain yield and quality traits across 3K RG panel reveals scope for the development of tailor-made rice with enhanced genetic gains. Plant Biotechnol. J..

[23] Rasheed A., Hao Y., Xia X., Khan A., Xu Y., Varshney R.K., He Z. (2017). Crop breeding chips and genotyping platforms: Progress, challenges, and perspectives. Mol. Plant.

[24] Yao W., Li G., Yu Y., Ouyang Y. (2018). funRiceGenes dataset for comprehensive understanding and application of rice functional genes. Gigascience.

[25] Yamamoto E., Yonemaru J.-I., Yamamoto T., Yano M. (2012). OGRO: The Overview of functionally characterized Genes in Rice online database. Rice.

[26] Bradbury P.J., Zhang Z., Kroon D.E., Casstevens T.M., Ramdoss Y., Buckler E.S. (2007). TASSEL: Software for association mapping of complex traits in diverse samples. Bioinformatics.

[27] McLaren W., Gil L., Hunt S.E., Riat H.S., Ritchie G.R., Thormann A., Flicek P., Cunningham F. (2016). The ensembl variant effect predictor. Genome Biol..

[28] Choi Y., Chan A.P. (2015). PROVEAN web server: A tool to predict the functional effect of amino acid substitutions and indels. Bioinformatics.

[29] Zhao H., Li J., Yang L., Qin G., Xia C., Xu X., Su Y., Liu Y., Ming L., Chen L.-L. (2021). An inferred functional impact map of genetic variants in rice. Mol. Plant.

[30] Wang C., Yu H., Huang J., Wang W.-S., Faruquee M., Zhang F., Zhao X.-Q., Fu B.-Y., Chen K., Zhang H.-L. (2019). Towards a deeper haplotype mining of complex traits in rice with RFGB v2. 0. Plant Biotechnol. J..

[31] Hu Z., Lu S.-J., Wang M.-J., He H., Sun L., Wang H., Liu X.-H., Jiang L., Sun J.-L., Xin X. (2018). A novel QTL qTGW3 encodes the GSK3/SHAGGY-like kinase OsGSK5/OsSK41 that interacts with OsARF4 to negatively regulate grain size and weight in rice. Mol. Plant.

[32] Davidson R.M., Gowda M., Moghe G., Lin H., Vaillancourt B., Shiu S.H., Jiang N., Buell C.R. (2012). Comparative transcriptomics of three Poaceae species reveals patterns of gene expression evolution. Plant J..

[33] Matvienko M. (2015). CLC Genomics Workbench. Plant and Animal Genome. Sr. Field Application Scientist.

[34] Howe E., Holton K., Nair S., Schlauch D., Sinha R., Quackenbush J. (2010). Mev: Multiexperiment viewer. Biomedical Informatics for Cancer Research.

[35] Sato Y., Takehisa H., Kamatsuki K., Minami H., Namiki N., Ikawa H., Ohyanagi H., Sugimoto K., Antonio B.A., Nagamura Y. (2013). RiceXPro version 3.0: Expanding the informatics resource for rice transcriptome. Nucleic Acids Res..

[36] Yu Y., Zhang H., Long Y., Shu Y., Zhai J. (2022). PPRD: A comprehensive online database for expression analysis of ~45,000 plant public RNA-Seq libraries. bioRxiv.

[37] Tzfadia O., Diels T., De Meyer S., Vandepoele K., Aharoni A., Van de Peer Y. (2016). CoExpNetViz: Comparative co-expression networks construction and visualization tool. Front. Plant Sci..

[38] Shannon P., Markiel A., Ozier O., Baliga N.S., Wang J.T., Ramage D., Amin N., Schwikowski B., Ideker T. (2003). Cytoscape: A software environment for integrated models of biomolecular interaction networks. Genome Res..

[39] Langfelder P., Horvath S. (2008). WGCNA: An R package for weighted correlation network analysis. BMC Bioinform..

[40] Jin J., Tian F., Yang D.-C., Meng Y.-Q., Kong L., Luo J., Gao G. (2016). PlantTFDB 4.0: Toward a central hub for transcription factors and regulatory interactions in plants. Nucleic Acids Res..

[41] Smedley D., Haider S., Durinck S., Pandini L., Provero P., Allen J., Arnaiz O., Awedh M.H., Baldock R., Barbiera G. (2015). The BioMart community portal: An innovative alternative to large, centralized data repositories. Nucleic Acids Res..

[42] Guo L., Chen W., Tao L., Hu B., Qu G., Tu B., Yuan H., Ma B., Wang Y., Zhu X. (2020). GWC1 is essential for high grain quality in rice. Plant. Sci..

[43] Zhang Y., Lan H., Shao Q., Wang R., Chen H., Tang H., Zhang H., Huang J. (2016). An A20/AN1-type zinc finger protein modulates gibberellins and abscisic acid contents and increases sensitivity to abiotic stress in rice (*Oryza sativa*). J. Exp. Bot..

[44] Angira B., Addison C.K., Cerioli T., Rebong D.B., Wang D.R., Pumplin N., Ham J.H., Oard J.H., Linscombe S.D., Famoso A.N. (2019). Haplotype characterization of the sd1 Semidwarf gene in United States Rice. Plant. Genome.

[45] Li Y., Fan C., Xing Y., Yun P., Luo L., Yan B., Peng B., Xie W., Wang G., Li X. (2014). Chalk5 encodes a vacuolar H+-translocating pyrophosphatase influencing grain chalkiness in rice. Nat. Genet..

[46] Zhao D.-S., Li Q.-F., Zhang C.-Q., Zhang C., Yang Q.-Q., Pan L.-X., Ren X.-Y., Lu J., Gu M.-H., Liu Q.-Q. (2018). GS9 acts as a transcriptional activator to regulate rice grain shape and appearance quality. Nat. Commun..

[47] Deshmukh R.K., Vivancos J., Ramakrishnan G., Guérin V., Carpentier G., Sonah H., Labbé C., Isenring P., Belzile F.J., Bélanger R.R. (2015). A precise spacing between the NPA domains of aquaporins is essential for silicon permeability in plants. Plant. J..

[48] Du Q., Campbell M., Yu H., Liu K., Walia H., Zhang Q., Zhang C. (2019). Network-based feature selection reveals substructures of gene modules responding to salt stress in rice. Plant. Direct.

[49] Shahan R., Zawora C., Wight H., Sittmann J., Wang W., Mount S.M., Liu Z. (2018). Consensus coexpression network analysis identifies key regulators of flower and fruit development in wild strawberry. Plant. Physiol..

[50] Pan Y., Li Q., Wang Z., Wang Y., Ma R., Zhu L., He G., Chen R. (2014). Genes associated with thermosensitive genic male sterility in rice identified by comparative expression profiling. BMC Genom..

[51] Sun S., Wang D., Li J., Lei Y., Li G., Cai W., Zhao X., Liang W., Zhang D. (2021). Transcriptome Analysis Reveals Photoperiod-Associated Genes Expressed in Rice Anthers. Front. Plant. Sci..

[52] Wang Q., Zeng X., Song Q., Sun Y., Feng Y., Lai Y. (2020). Identification of key genes and modules in response to cadmium stress in different rice varieties and stem nodes by weighted gene co-expression network analysis. Sci. Rep..

