# Supplementary material for: Deciphering Haplotypic Variation and Gene Expression Dynamics Associated with Nutritional and Cooking Quality in Rice
Source: Cells. 2022 Mar 28;11(7):1144. doi: 10.3390/cells11071144 (PMC8998046; doi:10.3390/cells11071144)
Supplement: Supplementary file 1 [file cells-11-01144-s001.zip › cells-1589502-supplementary.pdf]

Supplementary Tables and Figures

Supplementary Table S1. Details of Single nucleotide polymorphism (SNPs), Insertions/ deletions (InDels), and their haplotyping information for nutritional and cooking quality related genes. The deleterious effect mutations are represented with an asterisk

Gene Name	RAP ID	Feature	Total SNPs (Rice Var Map 2.0)	Total SNPs (SNP-SEEK IRRI)	Number of Missense mutations	Functional Impact of Missense Mutations	Number of haplotypes	Haplotype with the highest frequency	Number of accessions under haplotype with the highest frequency	Number of InDels
SSG4	Os01g0179400	Starch size in endosperm	58	65	6	I17V,E69D,I114V,D135G,K406R,I638S	8	3	1931	64
GW5L	Os01g0190500	Grain weight	82	8	3	V58G,A70S,H235R*,V58A	12	1	1349	70
OsROS1|TA2	Os01g0218032	Aleurone thickness	0	31	0		0	9	0	64
RBP-P	Os01g0265800	Endosperm protein localization	24	10	0		5	1	2473	87
PHD1	Os01g0367100	Galactolipid biosynthesis	72	48	6	D63V,F335I*,S321C*,G305V*,D288N*,F273V*	12	1	2707	186
OsAPL2|osagpl2-3|OsAGPL2|GIF2	Os01g0633100	Grain filling	109	37	0		19	5	1247	43
glu4a	Os01g0762500	Glutelin content	29	15	8	Y5H*	8	2	1224	7
OsAAP6|qPC1	Os01g0878700	Grain protein content	55	12	2	R361S,A113T	10	3	1768	39
sd1|GA20	Os01g088	Grain	66	21	9	A82N*,A82N*,E100G,H101D*,H101D*,C	11	2	2051	105

Gene Name	RAP ID	Feature	Total SNPs (Rice Var Map 2.0)	Total SNPs (SNP-SEEK IRRI)	Number of Missense mutations	Functional Impact of Missense Mutations	Number of haplotypes	Haplotype with the highest frequency	Number of accessions under haplotype with the highest frequency	Number of InDels
ox2	3800	protein content				193S*,P240L*,L266F*,Q340R,D349H				
OsIRO2	Os01g0952800	Iron utilization	430	296	20	K240N,V184I,L151M,S149N,T136A,T135A,A55T	7	1	1596	404
OsHAC1;1	Os02g0102300	Arsenic accumulation	59	30	1	Y5H	4	1	2227	162
OsUgp2|Ugp2	Os02g0117700	Starch accumulation	45	22	2	G406R,A62E	11	1	1847	68
OsHMA4	Os02g0196600	Copper accumulation	47	20	8	I55M,F303L,T316M,A553V,S660A,I704M,G818S,V914A*	12	1	1521	64
OsBT1-1|OsBT1	Os02g0202400	Endosperm granule formation	65	18	17	V25A,C46R,R102K,M107I,S115A,H127Q,R130Q,R152C*,R170H,M173I,R183H*,G184D*,T205R*,Y209C,P217L,V224I,Y209F	9	2	2240	70
GW2	Os02g0244100	Grain width and weight	106	36	4	E189D,R262C,R394C,R260C	17	1	1295	84
GluB-1	Os02g0249900	Seed storage protein	0	1	0		0	0	0	0
Du3	Os02g0612300	Grain amylose content	107	35	7	S96F*,Y237F,R189K	21	1	1686	116
OsYSL2	Os02g0649900	Iron nutrition	87	6	3	R588H,R103W,D21V	15	3	1461	4
OsMADS6	Os02g0682200	Endosperm nutrient	107	22	3	M93L,M182I,P203S	7	1	1793	251

Gene Name	RAP ID	Feature	Total SNPs (Rice Var Map 2.0)	Total SNPs (SNP-SEEK IRRI)	Number of Missense mutations	Functional Impact of Missense Mutations	Number of haplotypes	Haplotype with the highest frequency	Number of accessions under haplotype with the highest frequency	Number of InDels
		accumulation								
OsFAD2|OsFAD2-1	Os02g0716500	Grain lipid accumulation	124	47	3	K443Q,A163T,T50A	15	2	1820	177
OsCDPK1|OsCDPK13|OsCDPK11|OsCPK11|OsCDPK12	Os03g0128700	Grain starch structure	67	6	8	I143T*,E259Q*,A265S*,I268V*,Q284P,F288Y*,R355W*,V474I	8	3	2768	54
OsGAPDHB	Os03g0129300	Grain aroma	34	9	0		10	2	2891	36
XS-lpa2-1	Os03g0142800	Seed phytic acid	41	9	6	N6T,A19T,R32L,A350T,N645K*,L1469F*	10	3	1946	28
OsPht1;2|OsPT2	Os03g0150800	Selenite uptake	20	2	5	H398R,N335D,P269S,S258C*,V185I	9	2	2819	6
RINO1	Os03g0192700	Phytic acid	74	10	5	L376P,I43V,Q25G,Q25G,D23E,Q25R,Q25K	12	2	2255	53
OsFRDL1	Os03g0216700	Grain iron distribution	132	69	4	A409T,V227L,P56L,V30A	14	2	2014	197
lpa1	Os03g0237250	Phytic acid content	68	25	4	G420S,A351S,G132S,G127A	9	3	1534	130
OASA2	Os03g0264400	Grain tryptophan content	78	31	6	E585D,G527R*,P446S,R303P,E79K,R68P	17	1	1315	85
OsNAS2	Os03g0307200	Grain zinc content	30	5	4	A87T,N91S,G216E,I252V	6	3	1956	14

Gene Name	RAP ID	Feature	Total SNPs (Rice Var Map 2.0)	Total SNPs (SNP-SEEK IRRI)	Number of Missense mutations	Functional Impact of Missense Mutations	Number of haplotypes	Haplotype with the highest frequency	Number of accessions under haplotype with the highest frequency	Number of InDels
OsLTPL36	Os03g0369100	Grain development	30	0	0		9	4	1576	31
OsCCX2	Os03g0656500	Grain cadmium content	61	9	5	H90N,D292N,F412V*,V448L*,L532V	7	1	3833	12
OsIRT1	Os03g0667500	Grain iron and zinc content	243	128	20	V369I,R307K,R304K,R281K,I227M,V213A,R189W,H180R,V174I,N122S,N121S,G93R,L82F,V71A,A58V*,D49E,I29L,I27F,L21V,P9A	5	1	3828	217
lpaN15-186	Os03g0737701	Phytic acid	50	11	2	M355I,A150T	9	2	1498	6
OsMADS34|PAP2	Os03g0753100	Grain yield	90	20	5	Q89H*,Q106K*,Q71K*,T20A*,R10P*	8	1	1992	140
pho1	Os03g0758100	Starch structure in endosperm	33	15	11	T268N*,V165I,E153K,R550H*,R501C*,P391S*,T268N*,I254F*,S203L*,L60F*,M1del*	9	1	2713	37
CysR10	Os03g0766000	Grain protein bodies	40	16	2	V40A	7	1	2341	64
OASA1D	Os03g0826500	Tryptophan accumulation	85	30	3	R498L,G395E,G22E	9	2	1830	85
sug1	Os04g0164900	Rice endosperm development	0	412	0		0	0	0	607
OsVIT1	Os04g0463400	Iron translocation	72	28	5	A170T*,V136A*,Q105K	10	1	3814	70

Gene Name	RAP ID	Feature	Total SNPs (Rice Var Map 2.0)	Total SNPs (SNP-SEEK IRRI)	Number of Missense mutations	Functional Impact of Missense Mutations	Number of haplotypes	Haplotype with the highest frequency	Number of accessions under haplotype with the highest frequency	Number of InDels
Osvpe1	Os04g0537900	Seed glutelin	33	12	3	E384G*,Q90R,Y86C*	5	1	3293	27
OsYSL9	Os04g0542200	Iron distribution	48	13	4	S511N,T368R,L256F,R90L*	8	3	1503	22
Kala4|OsS1	Os04g0557500	Grain pericarp colour	1140	118	6	E308D,D173N,L140V,D101H,P84L,A29V*	14	3	1506	448
OsPTR6|PTR6|OsNPF7.3	Os04g0597800	Grain yield	111	27	3	I31V,I427V,M561L	13	2	2265	72
OsABCC1|MRP1	Os04g0620000	Arsenic accumulation	245	19	40	S1468T,R1398G*,R1300Q,E1231V*,R990Q,L933F,K892R,Q879L,V814L,R712H*,P642H*,A524S,R518C*,A449V,L285I,N283S,R276Q,T216S,P206L*,L176V,A156S,I150S,I134M,A107V,A92V,R90Q,T61A,G47S,T33S,N23Y*,V21L,S1468N,F708L*,A409T*,R383H*,S266I*,F252T*,A233V*,C228F*,C83F*,T29A*	23	3	810	135
FLO2	Os04g0645100	Grain size	117	59	30	A274T,L399P,I466T,R579K,P599L,G804D,S1203L,N1319D,A1608T,F195L*,H200N*,P204T*,S306P*,N323D*,M348R*,M348I*,L369F*,A378S*,G452S*,P515T*,W589C*,R725K*,A748V*,A789S*,N829Y*,R892Q*,G926C*,R987H*,A1060V*,L1107F*,Y1146F*	9	3	2953	217
OsSULTR3;3	Os04g0652400	Metabolite content	49	30	3	F655I,I600V,A452S	9	3	2039	94
OsMKP1|GSN1|LARGE8|GLA1	Os05g0115800	Grain number and size	88	26	9	V731A,T707M,T618I,D406N,V380L,S378I,V191I,R123H,G40R	16	1	1151	28
Chalk5	Os05g0156900	Chalkiness	212	96	19	R525L,I497V,V412I,K401M,A379T,A364V,G237R*,A215V,A139G,R137S,T125P,V62D,G61V,G59V,D58G,S52N,E49D,V48G,M	11	2	1371	143

Gene Name	RAP ID	Feature	Total SNPs (Rice Var Map 2.0)	Total SNPs (SNP-SEEK IRRI)	Number of Missense mutations	Functional Impact of Missense Mutations	Number of haplotypes	Haplotype with the highest frequency	Number of accessions under haplotype with the highest frequency	Number of InDels
						30V				
GSE5	Os05g0187500	Grain size	25	4	2	D229N,A431G	5	1	2317	30
OsZIP7a|OsZIP7	Os05g0198400	Grain zinc and cadmium content	76	31	7	G11S,S18P,E32D,S77N,L210V,G217D,V263M	10	1	2047	119
OsSPL9	Os05g0408200	Grain copper accumulation	79	47	7	A132V,A200T,P309S,I576V,S751T,I789V,S248F*	8	2	2743	114
OsPCS1	Os05g0415200	Arsenic content	39	15	1	H108R	8	2	2561	23
OsMATE2	Os05g0554000	Plant growth and development	83	17	4	T133A,F439Y,A462V,V493A	10	3	2316	71
Wx	Os06g0133000	Grain characteristics	137	45	4	D166G*,Y224S*,P415S,D528Y*,D528N	14	2	1253	205
SPDT	Os06g0143700	Phosphorus accumulation	281	152	6	Q385L*,I251V,L247F,V71G*,A47V,A21V	7	3	2072	308
OsSSI	Os06g0160700	Grain starch content	197	66	12	S596L,K438E*,H420Y*,S319G,D214N*,L86F,A78S,T74A,L60M,R29L,R343S*,C251Y*	10	2	1589	138
ALK|SSIIa	Os06g0229800	Grain starch quality	98	65	9	P56A,T117P,A148S,D161E,E208D,D283E,S604G,M737V,L781F*	16	1	842	65
OsLCT1	LOC_Os06g38120	Cadmium in grains	236	0	31	E15D,D26A,E35Q,P43L,P48S,A54S,I60T,L67H,L70Q,A71D,G73D,A77T,A80S,N84K,E87K,V95I,L101F,T147S,R152S,V183A,V21	4	1	4215	218

Gene Name	RAP ID	Feature	Total SNPs (Rice Var Map 2.0)	Total SNPs (SNP-SEEK IRRI)	Number of Missense mutations	Functional Impact of Missense Mutations	Number of haplotypes	Haplotype with the highest frequency	Number of accessions under haplotype with the highest frequency	Number of InDels
						1L,L215F,K223N,M241V,Q246H,E258L,M310V,L380F,T480S,V494M,L495F*				
OsHMA2|OsHMA2v	Os06g0700700	Grain zinc and cadmium content	104	153	3	C19R*,R7W	8	3	1706	261
OsPK2|W59|OsPKpalpha1	Os07g0181000	Grain filling	110	42	6		5	2	2108	94
qCdT7|OsHMA3	Os07g0232900	Grain zinc content	142	19	56	G990A,E975D,C960G,T953I,G930R,D926G,K912R,A908G,A908T,S873G,G787S,E775D,G770A,D768A,A759P,A758S,V752A,E733K,C678R,A638V,S614D,S614D,G595A,G594S*,S575T,D556H,V550I,T526I,S525T,R493Q,G490A,A381V*,S380R*,D338N*,T333M*,F299L,N298I*,Q269R,G268S*,V259I,E257K*,G256D*,G256S*,V250I,E238D,A234V,I233L,V229A,A184S,T134M,G130S*,A95V,E93A,P92S,A91T,R80H*,S614G,P92T,N725H*,L708F*,V697A*,N686K*,G642D*,E607A*,A341T*,V323G*,W293C*,P283L*,D267G*,D262N*,A252V*,R163C*,A99V*,D87N*,V82A*	10	2	2306	50
OsNRAMP5	Os07g0257200	Grain cadmium	163	73	3	A375T,S183F,S182A	18	2	1404	430
GW7|GL7|SLG7	Os07g0603300	Grain quality and yield	60	22	10	I915M,S647A,S620G,N605K,P518S,A462S,R361H,R361C,L259F*	10	3	1667	75
RSUS3|SUS3	Os07g0616800	Grain starch content	45	12	7	A26T,E541K*,L551S,S559N,N634D,E637K,S15G*	12	3	1592	47
OsHMA7	Os07g062	Grain	75	13	4	A32V,C37R,L147V*,R159C	10	1	1361	5

Gene Name	RAP ID	Feature	Total SNPs (Rice Var Map 2.0)	Total SNPs (SNP-SEEK IRRI)	Number of Missense mutations	Functional Impact of Missense Mutations	Number of haplotypes	Haplotype with the highest frequency	Number of accessions under haplotype with the highest frequency	Number of InDels
	3200	iron and zinc content								
OsGZF1	Os07g0668600	Seed storage protein	26	7	9	R255H,A219S,S179P,M174I,L169V,A111P,A102V,R100H,E47K*,A111T	7	1	2276	12
OsNAS3	Os07g0689600		52	7	2	D87E,A327T	11	2	2434	34
OsMOT1;1	Os08g0101500	Grain molybdenum content	52	4	9	A125S,P183H,Q215E,M262I,I448T	16	1	1268	37
SSIIIa	Os08g0191433	Endosperm appearance	186	137	35	A33T,M38K,T43N,A62S,R109H,K116N,E142D,A184T,A195V,A217T,G226E,E231K,A268V,S350L,F401S,D427G,V480L,T486I,A503T,R576K,E641V,S681N,G686E,R702Q,R748H,E790V,G817D,V843E,L957M,Y964C*,K1006N*,R1118K,R1240H,A1528S,T1755I	14	4	1182	136
Badh2	Os08g0424500	Grain aroma	156	59	5	A190V,K244I*,A316E,P458S*,G468V*	21	2	1040	123
qGW8|OsSPL16|GW8	Os08g0531600	Grain quality and shape	69	36	8	P79L,A110V,D172N*,T274N,Q285K,G315S,M364I,A397T	10	2	1842	222
OsVIT2	Os09g0396900	Iron content in rice	38	12	0		8	1	4268	21
osaldh7	Os09g0440300	Endosperm colour	135	19	2	K242E,R226K	5	1	1531	107
GS9	Os09g0448500	Grain shape and aperanc	75	7	8	L15M,P202Q,A223S,P250L,A257P,A258P,A260P,R268C	8	2	1982	36

Gene Name	RAP ID	Feature	Total SNPs (Rice Var Map 2.0)	Total SNPs (SNP-SEEK IRRI)	Number of Missense mutations	Functional Impact of Missense Mutations	Number of haplotypes	Haplotype with the highest frequency	Number of accessions under haplotype with the highest frequency	Number of InDels
		e								
OsVPS22	Os09g0529700	Grain chalkiness	128	9	1	H77R	10	3	1548	35
OsPCR1	Os10g0112100	Grain weight/ zinc accumulation	153	48	9	P185R,H156L,F100L,A58S,A46G,P33S,A15P,S13A,E11G	6	2	3404	132
FLO16	Os10g0478200	Grain development	66	27	0		5	2	2501	72
OsDCL3b	Os10g0485600	Seed quality	59	37	14	H89L*,A115V*,P129S*,N149D,S155A,T181A,F205L,I355M,G712S,R811S,V874L,R1063Q,F1101V,I1320L	11	3	1585	135
OsCAO1|PGL	Os10g0567400	Grain yield and quality	28	8	4	L394F*,T181P,V35L	5	2	4422	39
Rab5a	Os12g0631100	Endosperm storage protein transport	170	63	7	V129I,Q174R,A178V,A178V,P184L,H197Q	7	3	1443	120

Supplementary Table S2. Details of RNASeq datasets used in the present study. All the raw data was retrieved from NCBI SRA database and analyzed using CLC Genomics workbench.

Illumina RNA Sequencing (RNA-Seq) of Oryza sativa, Nipponbare for the Conserved Poaceae Specific Genes project		
SRA Experiment	SRA Study:SRP008821	Samples
SRX100741	SRR352184	20 Day Leaves
SRX100743	SRR352187	Emerging Inflorescence
SRX100745	SRR352189	Early Inflorescence
SRX100746	SRR352190	Anther
SRX100747	SRR352192	Pistil
SRX100749	SRR352194	Seeds 5 days after pollination
SRX100753	SRR352204	Embryo 25 days after pollination
SRX100754	SRR352206	Endosperm 25 days after pollination
SRX100755	SRR352207	Seed 10 days after pollination
SRX100756	SRR352209	Endosperm 25 days after pollination
SRX100757	SRR352211	20 day leaves

**The novel quantitative trait locus qTGW3 encodes a GSK3-like kinase and negatively regulates grain size and weight in rice**			
BioProject: PRJNA386888	SRA Experiment	SRA Study: SRP107231	Samples
	SRX2823832	SRR5560727	wild type
	SRX2823833	SRR5560728	wild type
	SRX2823834	SRR5560729	wild type
	SRX2823835	SRR5560730	GSK5-/-
	SRX2823836	SRR5560731	GSK5-/-
	SRX2823837	SRR5560732	GSK5-/-
	SRX2823838	SRR5560733	ARF4-/-
	SRX2823839	SRR5560734	ARF4-/-
	SRX2823840	SRR5560735	ARF4-/-

Transcriptome of different rice cultivars research the flavonoid biosynthetic pathway			
BioProject:PRJNA475636	SRA Experiment	SRA Study:SRP150289	Samples
	SRX4195869	SRR7293214	grain

	SRX4195870	SRR7293213	grain
	SRX4195868	SRR7293215	endosperm
	SRX4195872	SRR7293211	pericarp
	SRX4195871	SRR7293212	pericarp
	SRX4195875	SRR7293208	grain
	SRX4195874	SRR7293209	grain
	SRX4195873	SRR7293210	endosperm
	SRX4195877	SRR7293206	grain
	SRX4195876	SRR7293207	grain
Transcriptome analysis of Heugjinju, seed development and heading date using Illumina HiSeq 2000			
Accession: ERP009858	Experiment: ERX779709		
Transcriptome analysis of Heugjinju, seed development and heading date + 7days using Illumina HiSeq 2000			
Accession: ERP009859	Experiment:ERX779710		
Transcriptome analysis of Heugjinju, seed development and heading date + 14days using Illumina HiSeq 2000			
Accession: ERP009860	Experiment:ERX779711		
Transcriptome analysis of Heugseol, seed development and heading date using Illumina HiSeq 2000			
Accession: ERP009861	Experiment:ERX779712		
Transcriptome analysis of Heugseol, seed development and heading date + 7days using Illumina HiSeq 2000			
Accession: ERP009862	Experiment:ERX779713		
Transcriptome analysis of Heugseol, seed development and heading date + 14days using Illumina HiSeq 2000			
Accession: ERP009863	Experiment:ERX779715		
Transcriptome analysis of Heugnam, seed development and heading date using Illumina HiSeq 2000			
Accession: ERP009864	Experiment:ERX779716		
Transcriptome analysis of Heugnam, seed development and heading date + 7days using Illumina HiSeq 2000			
Accession: ERP009866	Experiment:ERX779717		
Transcriptome analysis of Heugnam, seed development and heading date + 14days using Illumina HiSeq 2000			
Accession: ERP009867	Experiment:ERX779718		
Transcriptome analysis of Josaengheug_chal, seed development and heading date using Illumina HiSeq 2000			
Accession: ERP009879	Experiment:ERX780238		
Transcriptome analysis of Josaengheug_chal, seed development and heading date + 7days using Illumina HiSeq 2000			

Accession: ERP009880	Experiment:ERX782739
Transcriptome analysis of Josaengheug_chal, seed development and heading date + 14days using Illumina HiSeq 2000	
Accession: ERP009881	Experiment:ERX784240
Transcriptome analysis of Boseogheug_chal, seed development and heading date using Illumina HiSeq 2000	
Accession: ERP009892	Experiment:ERX808969
Transcriptome analysis of Boseogheug_chal, seed development and heading date + 7days using Illumina HiSeq 2000	
Accession: ERP009893	Experiment:ERX808970
Transcriptome analysis of Boseogheug_chal, seed development and heading date + 14days using Illumina HiSeq 2000	
Accession: ERP009894	Experiment:ERX808971
Accession: Transcriptome analysis of Sinnongheug_chal, seed development and heading date using Illumina HiSeq 2000	
Accession: ERP009895	Experiment:ERX808972
Transcriptome analysis of Sinnongheug_chal, seed development and heading date + 7days using Illumina HiSeq 2000	
Accession: ERP009896	Experiment:ERX808973
Transcriptome analysis of Sinnongheug_chal, seed development and heading date + 14days using Illumina HiSeq 2000	
Accession: ERP009897	Experiment:ERX808974
Transcriptome analysis of Dongjin, seed development and heading date using Illumina HiSeq 2000	
Accession: ERP009898	Experiment:ERX808975
Transcriptome analysis of Dongjin, seed development and heading date + 7days using Illumina HiSeq 2000	
Accession: ERP009899	Experiment:ERX808976
Transcriptome analysis of Dongjin, seed development and heading date + 14days using Illumina HiSeq 2000	
Accession: ERP009900	Experiment:ERX808977
Transcriptome analysis of Dongjin_chal, seed development and heading date using Illumina HiSeq 2000	
Accession: ERP009901	Experiment:ERX808978
Transcriptome analysis of Dongjin_chal, seed development and heading date + 7days using Illumina HiSeq 2000	
Accession: ERP009902	Experiment:ERX808979
Transcriptome analysis of Dongjin_chal, seed development and heading date + 14days using Illumina HiSeq 2000	
Accession: ERP009904	Experiment:ERX808980

Supplementary Table S3: Diversity analysis of 80 nutritional and cooking quality-related genes in terms of average pairwise divergence (π), estimated mutation rate (θ) and Tajima's D values

GeneID	Chromosome	StartChrPosition	SiteCount	AvgSiteCount	SegSites	PiPerBP	ThetaPerBP	TajimaD
Os01g0179400	1	41,27,288	65	63.54892	65	0.30854	0.1167	4.21853
Os01g0190500	1	48,26,604	8	6.96345	8	0.36419	0.1183	3.58516
Os01g0218032	1	64,53,022	31	29.88297	31	0.20937	0.11689	1.86053
Os01g0265800	1	90,64,023	10	9.6397	10	0.33072	0.11689	3.35153
Os01g0367100	1	1,49,93,220	48	45.98122	48	0.19151	0.11698	1.58801
Os01g0633100	1	2,53,54,168	37	36.12483	37	0.25503	0.11672	2.85729
Os01g0762500	1	3,20,76,774	15	14.87794	15	0.23852	0.1165	2.13264
Os01g0878700	1	3,81,33,892	12	11.86881	12	0.3329	0.11654	3.5725
Os01g0883800	1	3,83,83,221	21	19.47555	21	0.25489	0.11742	2.57653
Os01g0952800	1	4,19,71,749	296	281.5608	296	0.20628	0.11707	2.10697
Os02g0102300	2	1,24,668	30	28.90787	30	0.34151	0.1169	4.49553
Os02g0117700	2	9,26,294	22	21.80287	22	0.20007	0.11652	1.58654
Os02g0196600	2	54,04,813	20	19.79248	20	0.20332	0.11654	1.61676
Os02g0202400	2	57,36,616	18	17.55343	18	0.27491	0.11674	2.87973
Os02g0244100	2	81,15,283	36	35.04904	36	0.29831	0.11676	3.73573
Os02g0249900	2	84,65,269	1	0.64408	1	0.40658	0.12268	1.76462
Os02g0612300	2	2,41,00,241	35	34.47124	35	0.15387	0.1166	0.76441
Os02g0649900	2	2,61,71,875	6	5.67171	6	0.21622	0.11716	1.31731
Os02g0682200	2	2,78,76,185	22	21.47139	22	0.24847	0.11672	2.49947
Os02g0716500	2	2,97,25,380	47	46.40889	47	0.25344	0.11657	2.9154
Os03g0128700	3	16,27,188	6	5.88418	6	0.44617	0.11666	4.3887
Os03g0129300	3	16,62,783	9	8.91235	9	0.22767	0.11653	1.69058
Os03g0142800	3	23,68,355	9	8.85542	9	0.19769	0.11661	1.23281
Os03g0150800	3	28,15,656	2	1.99405	2	0.07489	0.11643	-0.35763
Os03g0192700	3	48,25,797	10	9.94124	10	0.09982	0.11647	-0.26146
Os03g0216700	3	61,31,883	69	67.94186	69	0.18969	0.1166	1.61629

GeneID	Chromosome	StartChrPosition	SiteCount	AvgSiteCount	SegSites	PiPerBP	ThetaPerBP	TajimaD
Os03g0237250	3	72,37,375	25	23.87344	25	0.31927	0.11702	3.92384
Os03g0264400	3	87,02,596	31	30.46896	31	0.34251	0.11663	4.54996
Os03g0307200	3	1,09,26,483	5	4.49415	5	0.25522	0.11785	1.70803
Os03g0656500	3	2,56,13,922	9	8.15331	9	0.13527	0.11775	0.26548
Os03g0667500	3	2,62,86,278	128	122.533	128	0.10003	0.11699	-0.38952
Os03g0737701	3	3,02,44,613	11	10.25179	11	0.38413	0.11735	4.28906
Os03g0753100	3	3,10,48,492	20	19.34461	20	0.34321	0.11685	4.21168
Os03g0758100	3	3,13,32,056	15	14.71875	15	0.32238	0.11665	3.59371
Os03g0766000	3	3,17,26,165	16	15.8537	16	0.21357	0.11652	1.72223
Os03g0826500	3	3,47,15,941	30	29.22926	30	0.27022	0.11675	3.07397
Os04g0164900	4	44,08,409	412	396.4897	412	0.27339	0.11691	3.72288
Os04g0463400	4	2,31,34,671	28	27.59705	28	0.12753	0.11659	0.21679
Os04g0537900	4	2,69,04,203	12	11.91487	12	0.11668	0.11649	0.00312
Os04g0542200	4	2,71,54,447	13	12.37409	13	0.3775	0.11707	4.38333
Os04g0557500	4	2,79,15,619	118	101.702	118	0.29913	0.11844	4.1006
Os04g0597800	4	3,01,61,862	27	24.29714	27	0.18638	0.11784	1.34277
Os04g0620000	4	3,15,02,980	19	18.84306	19	0.14903	0.1165	0.59969
Os04g0645100	4	3,28,35,576	59	57.50056	59	0.25349	0.11674	2.9799
Os04g0652400	4	3,32,15,661	30	29.51568	30	0.317	0.11661	4.01606
Os05g0115800	5	8,58,506	26	25.76784	26	0.19447	0.11652	1.52627
Os05g0156900	5	33,35,388	96	88.19535	96	0.4029	0.11755	6.43074
Os05g0187500	5	53,65,256	4	3.31698	4	0.30845	0.11899	2.15946
Os05g0198400	5	60,90,807	31	30.5758	31	0.23536	0.11658	2.39305
Os05g0408200	5	1,99,23,998	47	46.17985	47	0.26167	0.11663	3.08827
Os05g0415200	5	2,03,02,417	15	14.79456	15	0.32518	0.11658	3.64483
Os05g0554000	5	2,75,42,160	17	16.37206	17	0.33183	0.1169	3.86115
Os06g0133000	6	17,65,761	45	43.34151	45	0.2732	0.1169	3.30681
Os06g0143700	6	22,92,676	152	147.4325	152	0.07764	0.11681	-0.90746
Os06g0160700	6	30,79,977	66	63.76309	66	0.37409	0.11686	5.6591
Os06g0229800	6	67,48,443	65	61.69255	65	0.19583	0.1171	1.72753

GeneID	Chromosome	StartChrPosition	SiteCount	AvgSiteCount	SegSites	PiPerBP	ThetaPerBP	TajimaD
Os06g0700700	6	2,94,73,748	153	148.4848	153	0.35977	0.1168	5.63172
Os07g0181000	7	42,66,889	42	39.97037	42	0.15931	0.11707	0.88566
Os07g0232900	7	74,05,972	19	18.39385	19	0.2357	0.11683	2.18823
Os07g0257200	7	88,71,767	73	70.65909	73	0.22392	0.11683	2.37606
Os07g0603300	7	2,46,64,520	22	21.68465	22	0.19962	0.11659	1.57623
Os07g0616800	7	2,54,29,921	12	11.90566	12	0.16957	0.1165	0.87654
Os07g0623200	7	2,57,91,175	13	12.89911	13	0.16963	0.1165	0.89606
Os07g0668600	7	2,82,33,372	7	6.90671	7	0.12424	0.11658	0.10758
Os07g0689600	7	2,93,23,445	7	6.86686	7	0.16426	0.11665	0.66818
Os08g0101500	8	86,771	4	3.9624	4	0.23901	0.11652	1.40525
Os08g0191433	8	53,52,201	137	134.2537	137	0.19903	0.11667	1.90138
Os08g0424500	8	2,03,79,846	59	57.8951	59	0.1761	0.11665	1.29607
Os08g0531600	8	2,65,01,258	36	35.3934	36	0.25308	0.11662	2.80962
Os09g0396900	9	1,38,31,895	12	11.8905	12	0.08845	0.11652	-0.46351
Os09g0440300	9	1,63,30,652	19	18.45389	19	0.2426	0.11679	2.3167
Os09g0448500	9	1,67,66,211	7	6.34739	7	0.20727	0.11773	1.25255
Os09g0529700	9	2,07,45,597	9	8.68011	9	0.36865	0.11689	3.8247
Os10g0112100	10	8,24,973	48	44.50129	48	0.1741	0.11743	1.20475
Os10g0478200	10	1,79,13,836	27	26.54252	27	0.25749	0.11663	2.77459
Os10g0485600	10	1,83,60,613	37	36.3006	37	0.34851	0.11665	4.79117
Os10g0567400	10	2,24,83,116	8	7.95773	8	0.10478	0.11646	-0.17132
Os12g0631100	12	2,70,22,270	63	62.51149	63	0.08756	0.1165	-0.63516

Supplementary Table S4. Details of hub genes and their interactions identified by co-expression network using RNAseq data

Hotspot gene	SUID	Average Shortest Path Length	bait_gene	Betweenness Centrality	Clustering Coefficient	Degree	Neighborhood Connectivity	Number Of Directed Edges	Stress
1	64	1.866667	os10g0567400	0.127522	0.242105	20	9.8	20	3216
2	86	1.983333	os06g0160700	0.137596	0.391813	19	11.84211	19	2066
3	97	1.9	os04g0645100	0.112704	0.327485	19	10.15789	19	2250
4	96	2.016667	os04g0652400	0.070166	0.385621	18	10.66667	18	1554
5	118	1.95	os03g0129300	0.14301	0.125	17	8.882353	17	3646
6	99	2.083333	os04g0597800	0.036403	0.483333	16	12.625	16	1124
7	114	2.15	os03g0216700	0.027757	0.483516	14	12.64286	14	850
8	74	2.283333	os08g0191433	0.061271	0.538462	13	10.30769	13	934
9	81	2.15	os07g0257200	0.011252	0.666667	13	13.92308	13	454
10	125	2.083333	os02g0244100	0.060028	0.564103	13	12.53846	13	1598
11	77	2.416667	os07g0668600	0.024494	0.681818	12	10.66667	12	736
12	79	2.416667	os07g0616800	0.020373	0.681818	12	10.66667	12	668
13	82	2.2	os07g0232900	0.005035	0.742424	12	14.41667	12	232
14	85	2.416667	os06g0229800	0.024494	0.681818	12	10.66667	12	736
15	106	2.316667	os03g0758100	0.022093	0.636364	12	11.91667	12	426
16	127	2.15	os02g0196600	0.017487	0.606061	12	14.25	12	656
17	102	2.25	os04g0537900	0.053353	0.636364	11	11.45455	11	704
18	104	2.216667	os03g0826500	0.031869	0.577778	10	12.8	10	1060
19	134	2.45	os01g0633100	0.008528	0.8	10	11.6	10	242
	94	2.716667	os05g0156900	0.007576	0.722222	9	10.11111	9	258
	103	2.383333	os04g0463400	0.062503	0.277778	9	10.22222	9	1112
	116	2.333333	os03g0150800	0.003624	0.75	9	14.66667	9	122
	138	2.4	os01g0190500	0.061841	0.416667	9	11.11111	9	1818
	84	2.4	os06g0700700	0.016197	0.214286	8	12.625	8	682
	105	2.416667	os03g0766000	0.063427	0.285714	8	9.875	8	1412
	109	2.383333	os03g0667500	0.001049	0.892857	8	14.25	8	62
	129	2.566667	os02g0102300	0.0181	0.678571	8	12.125	8	228

Hotspot gene	SUID	Average Shortest Path Length	bait_gene	Betweenness Centrality	Clustering Coefficient	Degree	Neighborhood Connectivity	Number Of Directed Edges	Stress
	113	2.5	os03g0237250	0.028467	0.47619	7	13	7	1084
	135	2.6	os01g0367100	0.007796	0.333333	7	9.428571	7	176
	139	2.533333	os01g0179400	0.080958	0.142857	7	5	7	1028
	72	3.183333	os08g0531600	0.024035	0.266667	6	4.166667	6	480
	83	2.316667	os07g0181000	0.030286	0.4	6	12.16667	6	812
	117	2.383333	os03g0142800	0.047426	0.133333	6	9.333333	6	842
	120	2.9	os02g0716500	0.046637	0.266667	6	5	6	1022
	131	2.65	os01g0883800	0.019819	0.066667	6	7.833333	6	432
	68	2.566667	os09g0529700	0.022586	0.4	5	8.2	5	558
	75	2.433333	os08g0101500	0.03657	0.3	5	14.2	5	610
	78	2.733333	os07g0623200	0.005018	0.4	5	10.2	5	138
	89	2.516667	os05g0554000	4.75E-04	0.7	5	17.6	5	28
	91	2.5	os05g0408200	0.007101	0.6	5	14.4	5	134
	69	3.283333	os09g0448500	5.18E-04	0.833333	4	6.5	4	6
	92	2.616667	os05g0198400	0.0416	0.166667	4	14.5	4	832
	93	3.283333	os05g0187500	5.18E-04	0.833333	4	6.5	4	6
	111	3.333333	os03g0369100	0	1	4	11.25	4	0
	137	3	os01g0218032	0.028768	0.166667	4	4.5	4	444
	65	2.633333	os10g0485600	0.014287	0	3	14.33333	3	438
	73	2.966667	os08g0424500	0.004986	0.333333	3	6.333333	3	88
	98	3.333333	os04g0620000	0.002252	0.666667	3	5.666667	3	36
	121	3.183333	os02g0682200	0.019997	0.333333	3	5.666667	3	234
	123	3.266667	os02g0612300	9.23E-04	0.666667	3	5.333333	3	12
	124	3.216667	os02g0249900	0	1	3	11.66667	3	0
	126	3.366667	os02g0202400	0	1	3	11	3	0
	80	2.866667	os07g0603300	0	1	2	13.5	2	0
	107	3.8	os03g0753100	0	1	2	4.5	2	0
	108	3.466667	os03g0737701	0.0126	0	2	3	2	258
	110	3.833333	os03g0656500	0.003426	0	2	3	2	32
	119	3.016667	os03g0128700	0.002408	0	2	9.5	2	36

Hotspot gene	SUID	Average Shortest Path Length	bait_gene	Betweenness Centrality	Clustering Coefficient	Degree	Neighborhood Connectivity	Number Of Directed Edges	Stress
	122	1	os02g0649900	1	0	2	1	2	2
	66	3.516667	os10g0478200	0	0	1	7	1	0
	71	1.5	os09g0396900	0	0	1	2	1	0
	101	3.883333	os04g0542200	0	0	1	6	1	0
	115	2.966667	os03g0192700	0	0	1	19	1	0
	130	1.5	os01g0952800	0	0	1	2	1	0
	132	2.883333	os01g0878700	0	0	1	19	1	0
	62	0	loc_os06g38120	0	0	0	0	0	0
	63	0	os12g0631100	0	0	0	0	0	0
	67	0	os10g0112100	0	0	0	0	0	0
	70	0	os09g0440300	0	0	0	0	0	0
	76	0	os07g0689600	0	0	0	0	0	0
	87	0	os06g0143700	0	0	0	0	0	0
	88	0	os06g0133000	0	0	0	0	0	0
	90	0	os05g0415200	0	0	0	0	0	0
	95	0	os05g0115800	0	0	0	0	0	0
	100	0	os04g0557500	0	0	0	0	0	0
	112	0	os03g0264400	0	0	0	0	0	0
	128	0	os02g0117700	0	0	0	0	0	0
	133	0	os01g0762500	0	0	0	0	0	0
	136	0	os01g0265800	0	0	0	0	0	0

Supplementary Table S5. Details of genetic variations previously reported for nutritional and cooking quality related genes

Gene	RAPdb	Chromosome_location	MSU	Mutations/role
Rab5a	Os12g0631100	chr12:27021987-27025197	LOC_Os12g43550	G->A splice donor first intron, Gly->Asp(45),Gln->Stop(176)
Rab5a	Os12g0631100	chr12:27021987-27025197	LOC_Os12g43550	13 bp deletion in the third exon
OsCAO1|PGL	Os10g0567400	chr10:22482881-22486532 (complement)	LOC_Os10g41780	L253F,A106V
OsDCL3b	Os10g0485600	chr10:18360057-18372796	LOC_Os10g34430	Knockdown
FLO16	Os10g0478200	chr10:17913821-17917768	LOC_Os10g33800	4 bp deletion in the fifth exon
OsPCR1	Os10g0112100	chr10:824623-826321 (complement)	LOC_Os10g02300	Knockdown and Knockout
OsVPS22	Os09g0529700	chr9:20744818-20748508 (complement)	LOC_Os09g36020	Knockout
GS9	Os09g0448500	chr9:16765820-16767992	LOC_Os09g27590	Null allele
osaldh7	Os09g0440300	chr9:16330101-16338641 (complement)	LOC_Os09g26880	Insertion mutants
osaldh7	Os09g0440300	chr9:16330101-16338641 (complement)	LOC_Os09g26880	C to T (985) substitution in the tenth exon
OsVIT2	Os09g0396900	chr9:13831851-13833641	LOC_Os09g23300	Knockdown
OsVIT2	Os09g0396900	chr9:13831851-13833641	LOC_Os09g23300	Knockout
qGW8|OsSPL16|GW8	Os08g0531600	chr8:26500458-26506276	LOC_Os08g41940	Loss of function mutation results in slender grains
Badh2	Os08g0424500	chr8:20379779-20386042	LOC_Os08g32870	19 alleles
SSIIIa	Os08g0191433	chr8:5349928-5363361	LOC_Os08g09230	Knockout
OsMOT1;1	Os08g0101500	chr8:86166-87855	LOC_Os08g01120	Sequence variation in promoter
OsNAS3	Os07g0689600	chr7:29323078-29324696	LOC_Os07g48980	Activation of NAS Gene
OsGZF1	Os07g0668600	chr7:28233219-28235926 (complement)	LOC_Os07g47240	Downregulation
OsHMA7	Os07g0623200	chr7:25790365-25792702	LOC_Os07g43040	Downregulation
RSUS3|SUS3	Os07g0616800	chr7:25429550-25435142	LOC_Os07g42490	SNPs in promoter region

Gene	RAPdb	Chromosome_location	MSU	Mutations/role
GW7|GL 7|SLG7	Os07g0603300	chr7:24664276-24669347 (complement)	LOC_Os07g41200	Gene upregulation
OsNRAM P5	Os07g0257200	chr7:8871580-8878943 (complement)	LOC_Os07g15370	Knockout
OsNRAM P5	Os07g0257200	chr7:8871580-8878943 (complement)	LOC_Os07g15370	Functionally deficient OsNRAMP5 affects metal accumulation
qCdT7|O sHMA3	Os07g0232900	chr7:7405544-7409595 (complement)	LOC_Os07g12900	Ser380Arg Loss of function allele
qCdT7|O sHMA3	Os07g0232900	chr7:7405544-7409595 (complement)	LOC_Os07g12900	Promoter Sequence variations
OsPK2| W59|Os PKpalpha 1	Os07g0181000	chr7:4266867-4271499	LOC_Os07g08340	Ser296Leu
OsHMA2 |OsHMA 2v	Os06g0700700	chr6:29473272-29481042 (complement)	LOC_Os06g48720	Insertion mutations
ALK|SSIIa	Os06g0229800	chr6:6748306-6753338	LOC_Os06g12450	Leu781Phe,Val737Met
ALK|SSIIa	Os06g0229800	chr6:6748306-6753338	LOC_Os06g12450	GC4327, 4328TT,A4196G
OsSSI	Os06g0160700	chr6:3079058-3086803 (complement)	LOC_Os06g06560	Polymorphism at -916 bp in the SSSI promoter Indel, an A/G SNP in exon 6, a C/T SNP in exon 8, a C/A SNP
SPDT	Os06g0143700	chr6:2292641-2298815 (complement)	LOC_Os06g05160	Val330Ala
SPDT	Os06g0143700	chr6:2292641-2298815 (complement)	LOC_Os06g05160	Knockout
Wx	Os06g0133000	chr6:1765524-1770644	LOC_Os06g04200	GT to TT mutation at the 5’ splice site of the first intron
Wx	Os06g0133000	chr6:1765524-1770644	LOC_Os06g04200	G/T intron 1
Wx	Os06g0133000	chr6:1765524-1770644	LOC_Os06g04200	Splice donor site at intron 1, 224 and 415 positions in exon 6 and 10
OsMATE 2	Os05g0554000	chr5:27542040-27545926	LOC_Os05g48040	Expression

Gene	RAPdb	Chromosome_location	MSU	Mutations/role
OsPCS1	Os05g0415200	chr5:20301984-20305758	LOC_Os05g34290	Alternative splicing
OsSPL9	Os05g0408200	chr5:19923172-19932449	LOC_Os05g33810	Glu561Lys
OsZIP7a|OsZIP7	Os05g0198400	chr5:6090719-6094071	LOC_Os05g10940	Loss of function
GSE5	Os05g0187500	chr5:5364877-5367038	LOC_Os05g09520	950-bp deletion in indica and 1212-bp deletion in japonica varieties
Chalk5	Os05g0156900	chr5:3335334-3339834(complement)	LOC_Os05g06480	CATGCA at 721,CACT tetranucleotide at 485 and 2 SNPs in promoter
OsMKP1|GSN1|LARGE8|GLA1	Os05g0115800	chr5:858395-862791 (complement)	LOC_Os05g02500	Loss of function
OsMKP1|GSN1|LARGE8|GLA1	Os05g0115800	chr5:858395-862791 (complement)	LOC_Os05g02500	SNP (861,675; G to A) exon 1
OsSULTR3;3	Os04g0652400	chr4:33215039-33220693 (complement)	LOC_Os04g55800	(1-bp) deletion 12th exon-stop codon and 6-bp deletion first exon
FLO2	Os04g0645100	chr4:32835149-32848270	LOC_Os04g55230	3 SNPs
OsABCC1|MRP1	Os04g0620000	chr4:31502901-31515366 (complement)	LOC_Os04g52900	Knockout
OsPTR6|PTR6|OsNPF7.3	Os04g0597800	chr4:30161413-30165517	LOC_Os04g50950	Overexpression
Kala4|OsS1	Os04g0557500	chr4:27939824-27915598	LOC_Os04g47059	6 SNPs, 1 InDel
OsYSL9	Os04g0542200	chr4:27154293-27159796 (complement)	LOC_Os04g45860	knockdown
Osvpe1	Os04g0537900	chr4:26903954-26908730 (complement)	LOC_Os04g45470	Cys269Gly
OsVIT1	Os04g0463400	chr4:23134255-23137105 (complement)	LOC_Os04g38940	expression
sug1	Os04g0164900	chr4:4405753-4418895		80 SNP in the gene

Gene	RAPdb	Chromosome_location	MSU	Mutations/role
OASA1D	Os03g0826500	chr3:34715402-34721077 (complement)		
CysR10	Os03g0766000	chr3:31725962-31732275 (complement)		
pho1	Os03g0758100	chr3:31332021-31339259 (complement)	LOC_Os03g55090	Knockout
OsMADS 34|PAP2	Os03g0753100	chr3:31048350-31055281 (complement)	LOC_Os03g54170	null mutation
OsMADS 34|PAP2	Os03g0753100	chr3:31048350-31055281 (complement)	LOC_Os03g54170	1014-bp deletion
lpaN15-186	Os03g0737701	chr3:30244152-30247443 (complement)	Sequence analysis of the N15-186 allele of this orthologue (Os03g52760) revealed a single base pair change (C/G to T/A) in the first exon of the gene, which results in a nonsense mutation. Our results indicate that lpa N15-186 is a mutant allele of the rice myo-inositol kinase (OsMIK) gene.	nonsense mutation in first exon
OsIRT1	Os03g0667500	chr3:26286207-26292159 (complement)	LOC_Os03g46470	Overexpression
OsCCX2	Os03g0656500	chr3:25613813-25616205	LOC_Os03g45370	Knockout
OsLTPL36	Os03g0369100	chr3:14491725-14492433	LOC_Os03g25350	Knockout
OsNAS2	Os03g0307200	chr3:10926452-10927926	LOC_Os03g19420	Overexpression
OASA2	Os03g0264400	chr3:8702913-8707788 (complement)		site directed S126F/L530D mutations
lpa1	Os03g0237250	chr3:7237067-7241524 (complement)	LOC_Os03g13400	insersion in 3rd exon premature transcriptional termination

Gene	RAPdb	Chromosome_location	MSU	Mutations/role
OsFRDL1	Os03g0216700	chr3:6131845-6142900 (complement)	LOC_Os03g11734	Knockout
RINO1	Os03g0192700	chr3:4825696-4829742 (complement)		Expression
OsPht1;2 |OsPT2	Os03g0150800	chr3:2815495-2822792 (complement)	LOC_Os03g05640	Leu218Pro
OsPht1;2 |OsPT2	Os03g0150800	chr3:2815495-2822792 (complement)	LOC_Os03g05640	Overexpression
XS-lpa2-1	Os03g0142800	chr3:2367836-2374429		1 SNP (C/G–T/A transition) 6th exon of XS-lpa2-1 and a 5-bp deletion 1st exon of XS-lpa2-2
OsGAPDHB	Os03g0129300	chr3:1662177-1665491		P425S mutation
OsCDPK1	Os03g0128700	chr3:1624884-1629492	LOC_Os03g03660	expression and gene silencing
OsFAD2|OsFAD2-1	Os02g0716500	chr2:29725325-29730369(complement)	LOC_Os02g48560	gene silencing
OsMADS6	Os02g0682200	chr2:27875934-27884005	LOC_Os02g45770	Null allele
OsYSL2	Os02g0649900	chr2:26170543-26175198 (complement)	LOC_Os02g43370	Knockdown
Du3	Os02g0612300	chr2:24100127-24103567 (complement)		3 alleles
GluB-1	Os02g0249900	chr2:8465040-8467045	LOC_Os02g15169	Regulator
GW2	Os02g0244100	chr2:8114930-8121836	LOC_Os02g14720	A314 deletion
GW2	Os02g0244100	chr2:8114930-8121836	LOC_Os02g14720	Knockdown
OsBT1-1|OsBT1	Os02g0202400	chr2:5736568-5738793	LOC_Os02g10800	1 bp deletion GT->G exon 1
OsHMA4	Os02g0196600	chr2:5404709-5410739	LOC_Os02g10290	V914A
OsUgp2|Ugp2	Os02g0117700	chr2:926052-930040 (complement)	LOC_Os02g02560	5' deletion promoter and gain-of-function
OsHAC1;1	Os02g0102300	chr2:124618-127140	LOC_Os02g01220	Knockout
OsIRO2	Os01g0952800	chr1:41972004-41978403 (complement)	LOC_Os01g72370	Transcriptional activator

Gene	RAPdb	Chromosome_location	MSU	Mutations/role
sd1|GA20ox2	Os01g0883800	chr1:38382358-38385504	LOC_Os01g66100	Loss of function
OsAAP6|qPC1	Os01g0878700	chr1:38133432-38137790 (complement)	LOC_Os01g65670	(−7 to −12 bp, −32 bp) variations in the cis-regulatory elements of 5' UTR
glu4a	Os01g0762500	chr1:32076723-32078653		2 mutations L827S and G878E
OsAPL2|osagpl2-3|OsAGPL2|GIF2	Os01g0633100	chr1:25354534-25361907	LOC_Os01g44220	Single missense mutation
PHD1	Os01g0367100	chr1:14993203-14998127 9complement)	LOC_Os01g26920	(G-to-T) in exon 2,(A-to-T) in exon 7,3-bp Insertion between exon 3 and exon 4
RBP-P	Os01g0265800	chr1:9063927-9066803 (complement)	LOC_Os01g16090	3 mutations A252T, G373E, and G401S
OsROS1|TA2	Os01g0218032	chr1:6443035-6456074	LOC_Os01g11900	Knockout
GW5L	Os01g0190500	chr1:4826355-4828739	LOC_Os01g09470	Negative regulator
SSG4	Os01g0179400	chr1:4127233-4140749	LOC_Os01g08420	1 mutation G to A
OsLCT1	None	chr6:22566775-22571982	LOC_Os06g38120	Knockdown

Supplementary Figures

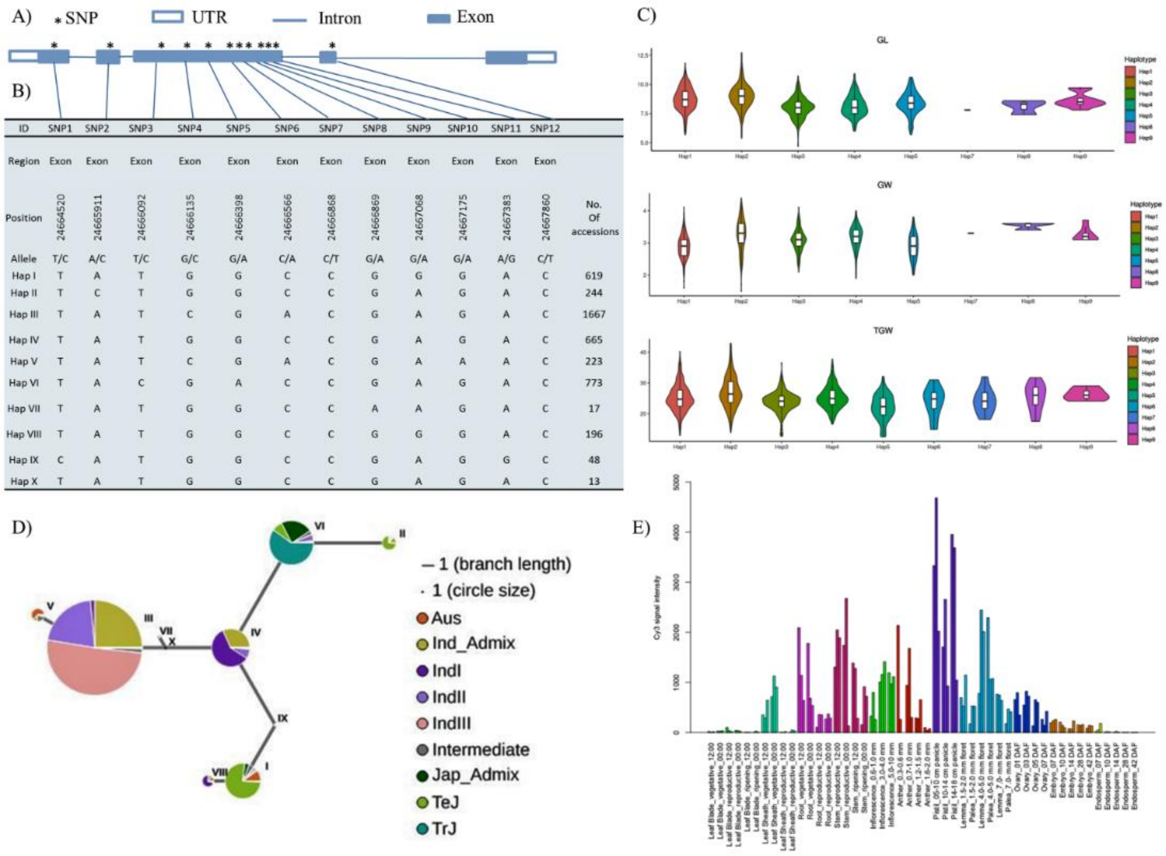

Supplementary Figure S1: Representative illustration showing genetic variations observed in *GL7* gene defining haplotypic diversity, evolution, and expression profile in different tissues. A) Intron-exon organization of *GL7* gene showing single nucleotide polymorphism sites; B) haplotypic grouping based on the SNPs present in the *GL7* gene; C) Violin plot showing the frequency

distribution of haplotypes; D) haplotypic network showing relatedness and allelic evolution of *GL7* gene; E) expression profiling of *GL7* gene in different tissues.

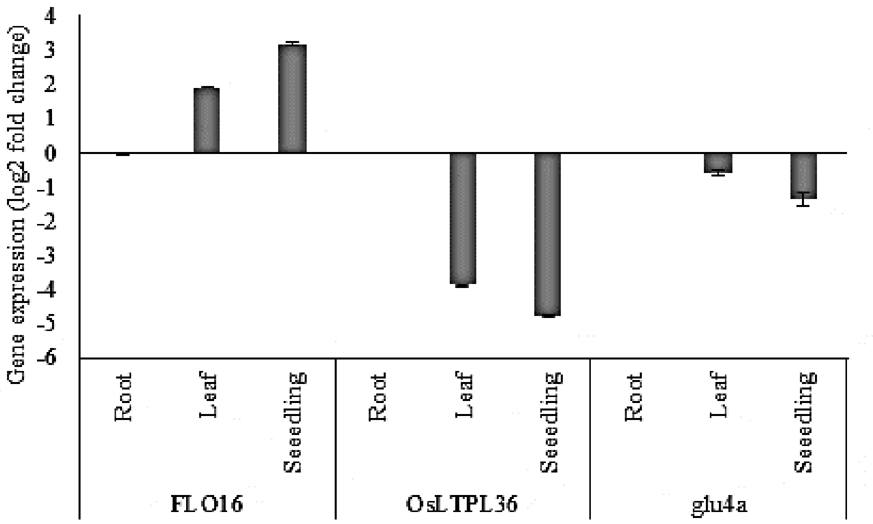

Supplementary Figure S2. The relative expression level of *FLO16*, *OsLTPL36*, and *glu4a* in root, leaf, and seedling, measured with real-time quantitative PCR (RT-qPCR). Bars show mean log(2) fold change ± SEM of three biological samples.

Supplementary Dataset 2. Diversity analysis between cultivated and wild rice accessions for 80 nutritional and cooking quality related genes.

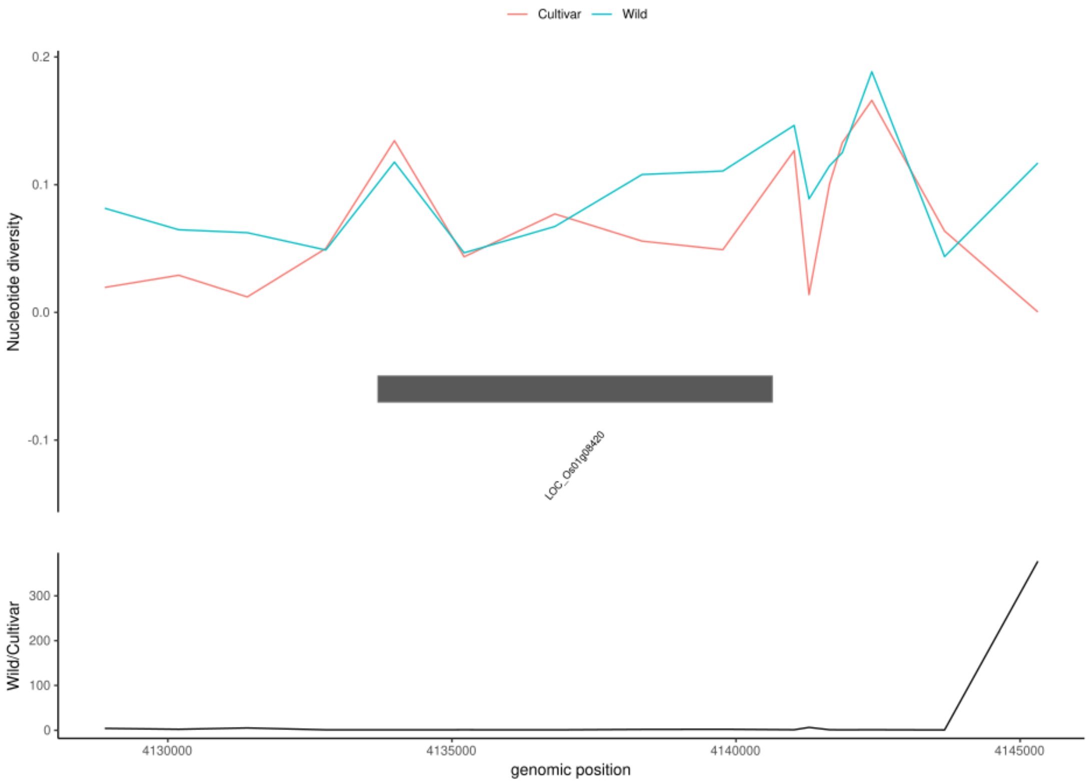

Os01g0179400

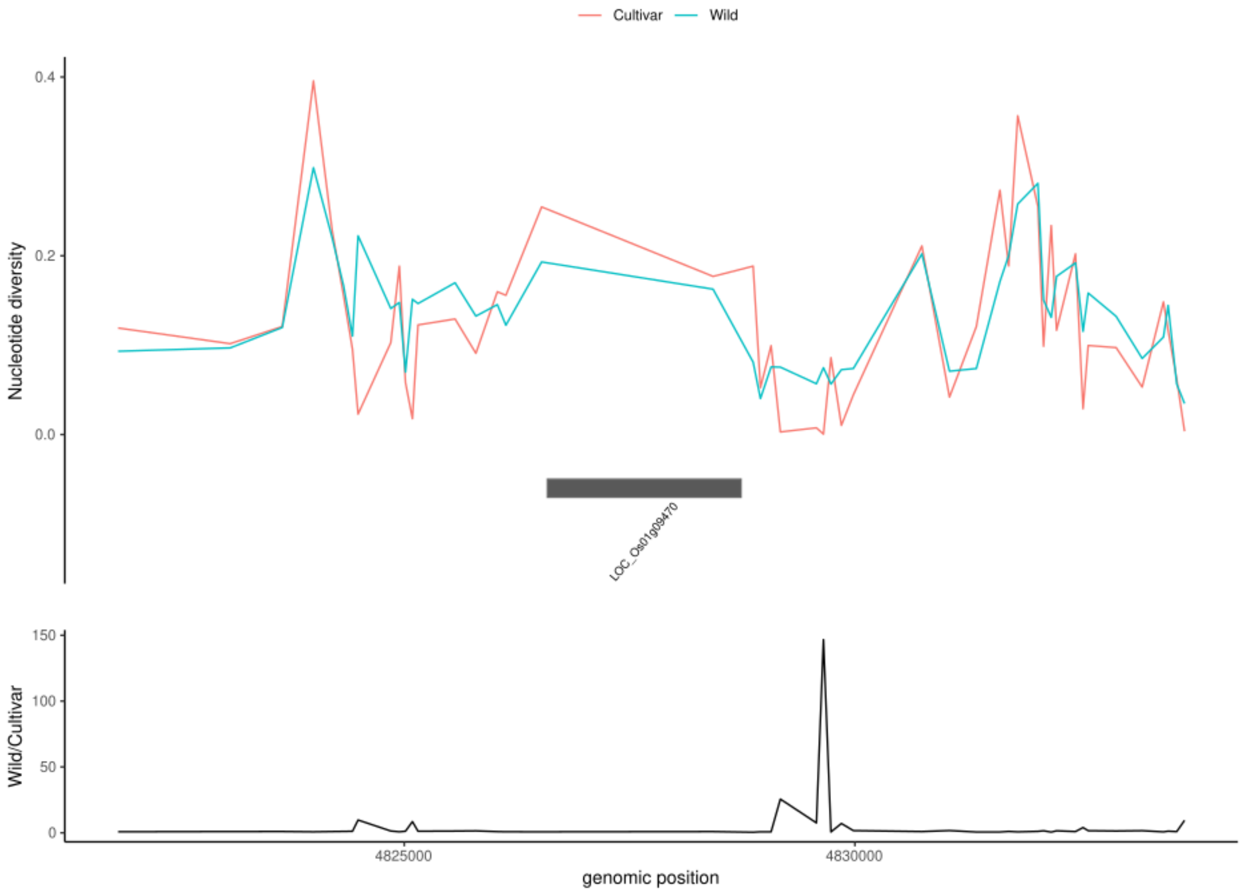

Os01g0190500

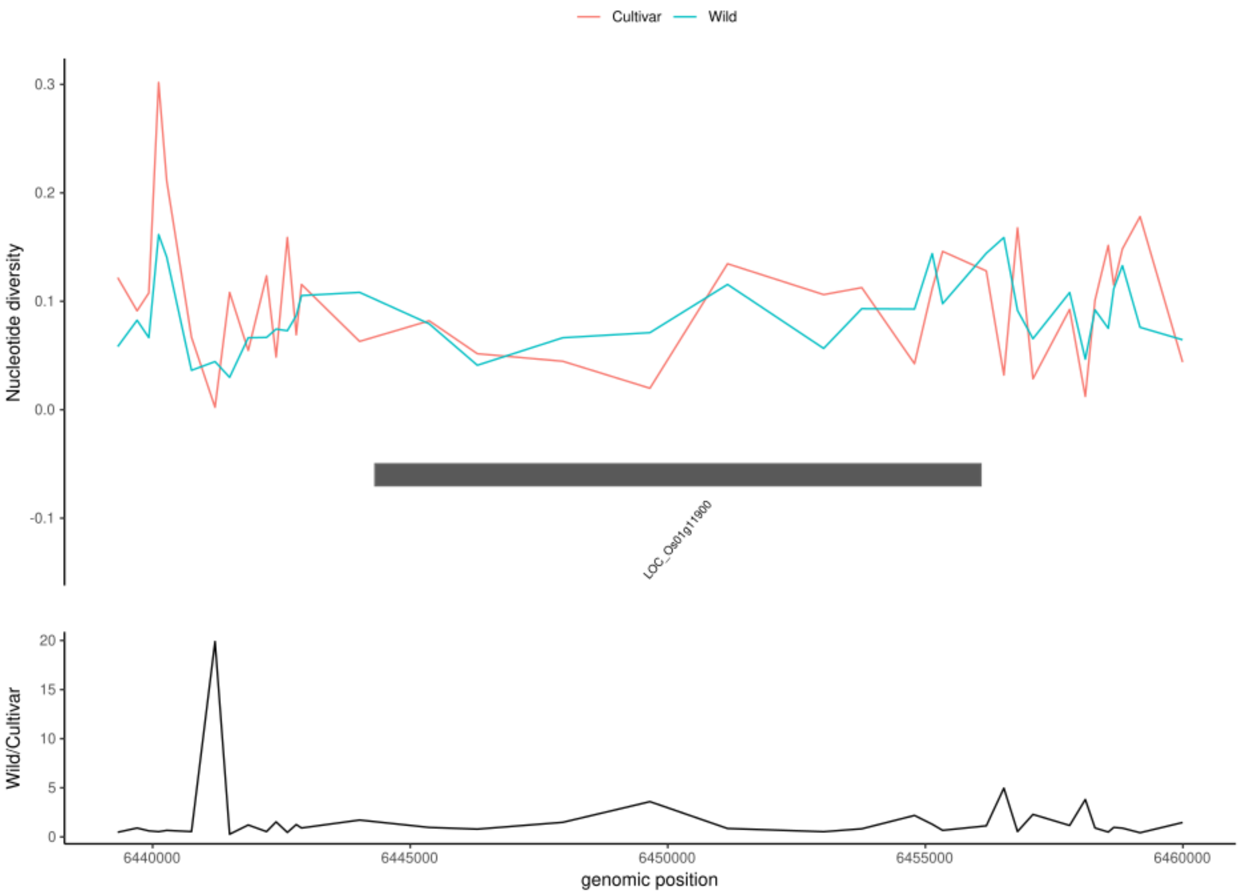

Os01g0218032

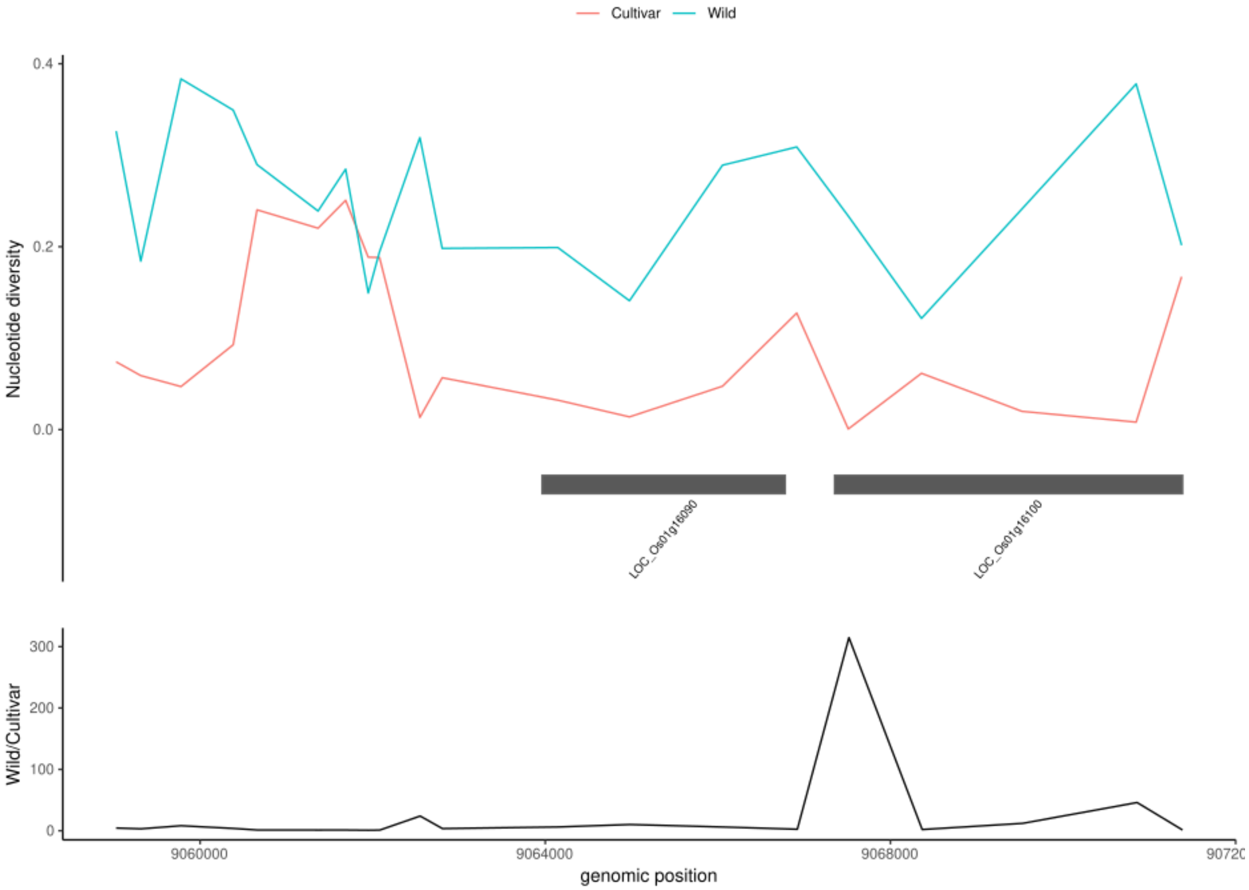

Os01g0265800

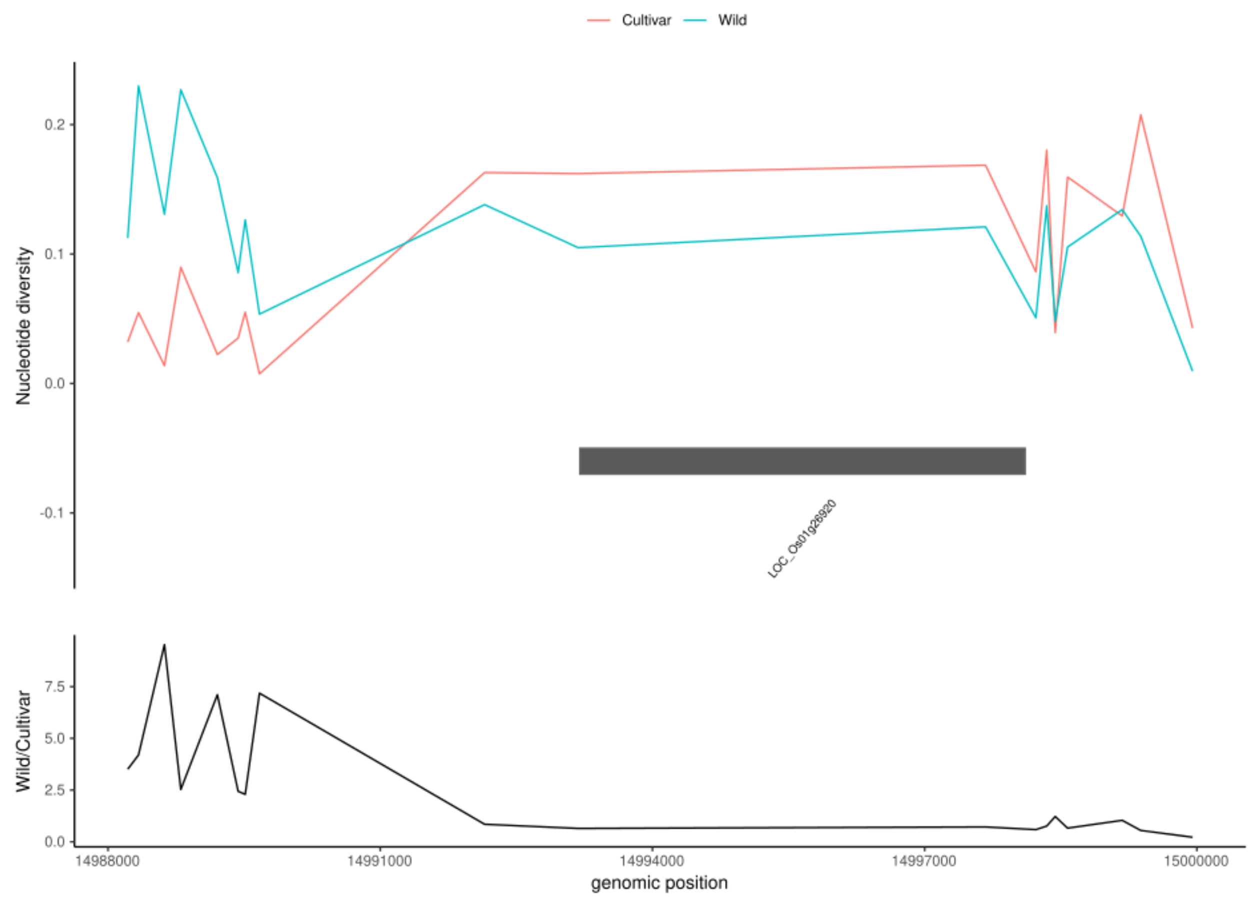

Os01g0367100

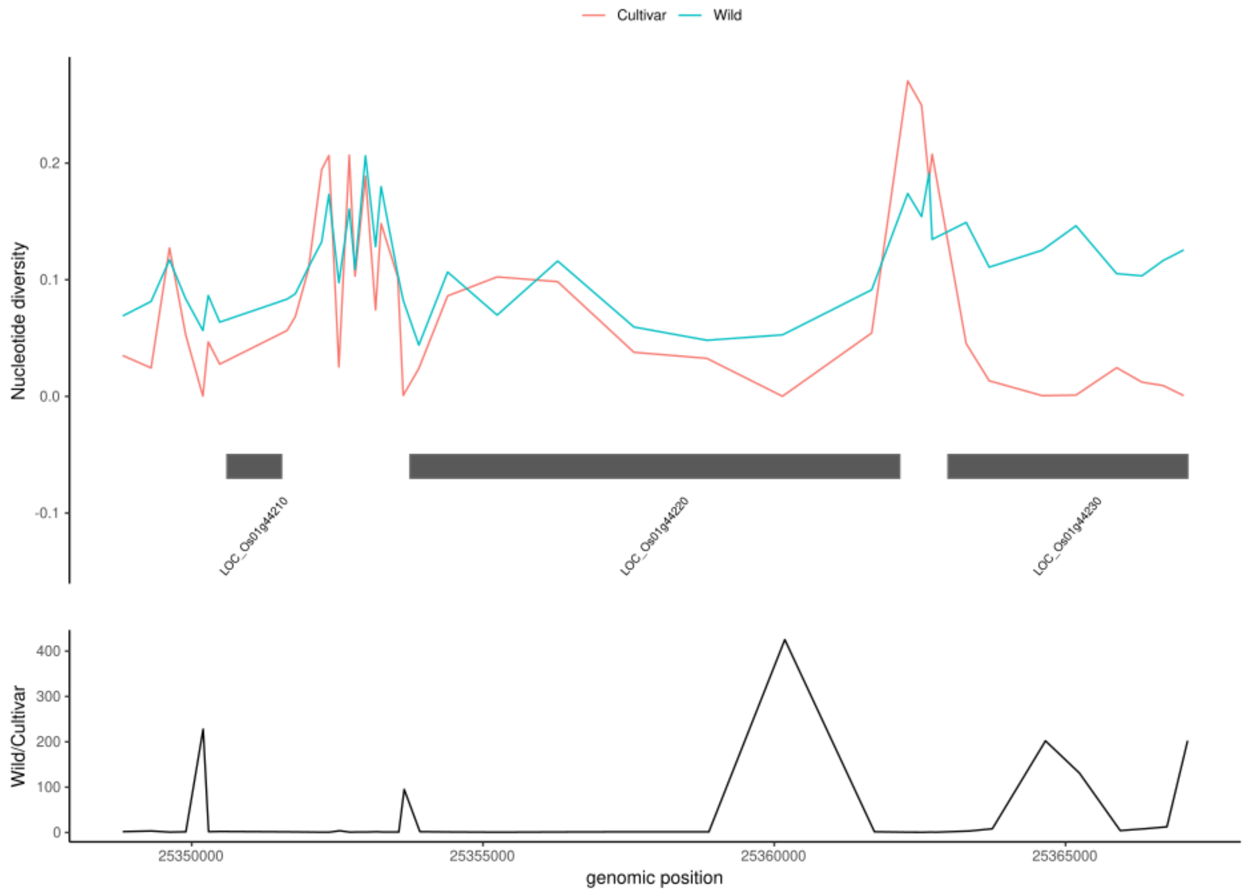

Os01g0633100

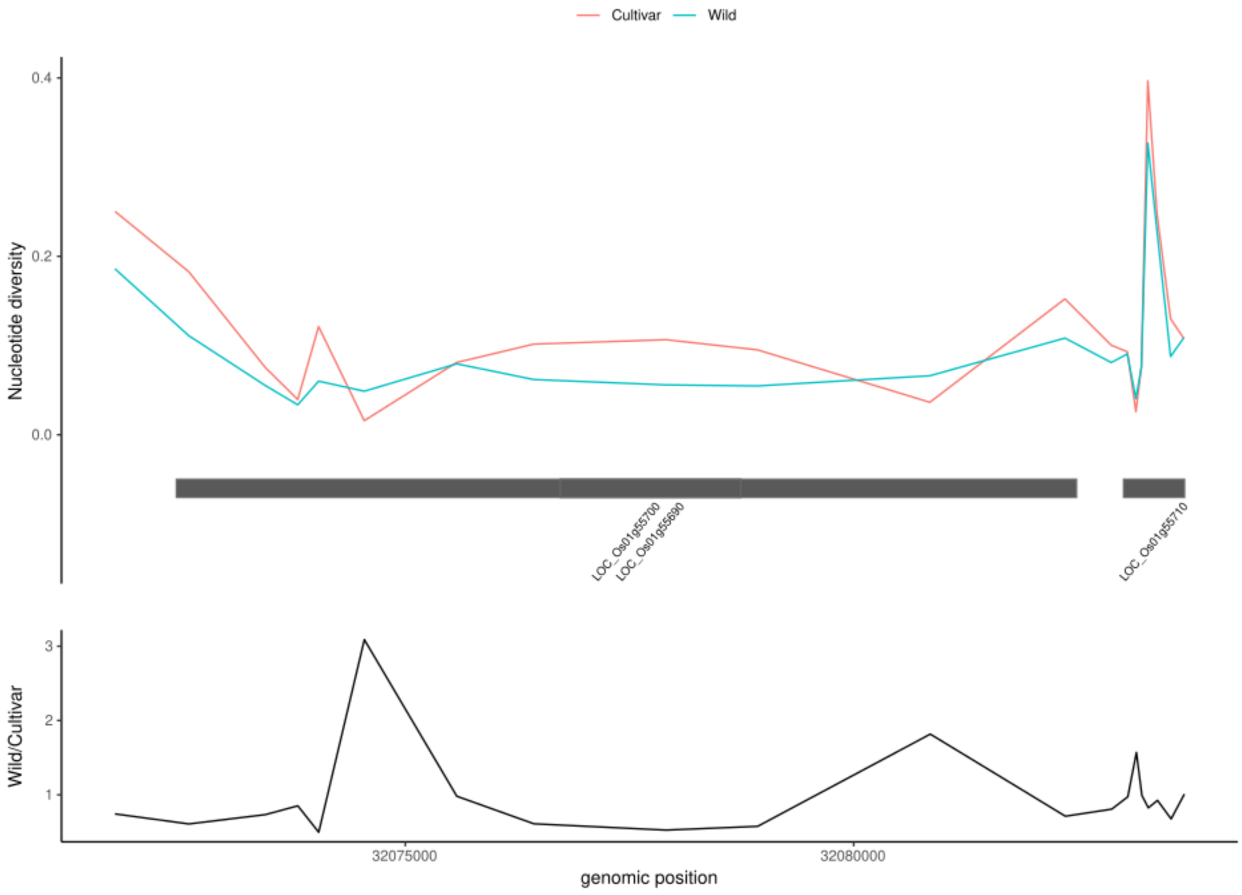

Os01g0762500

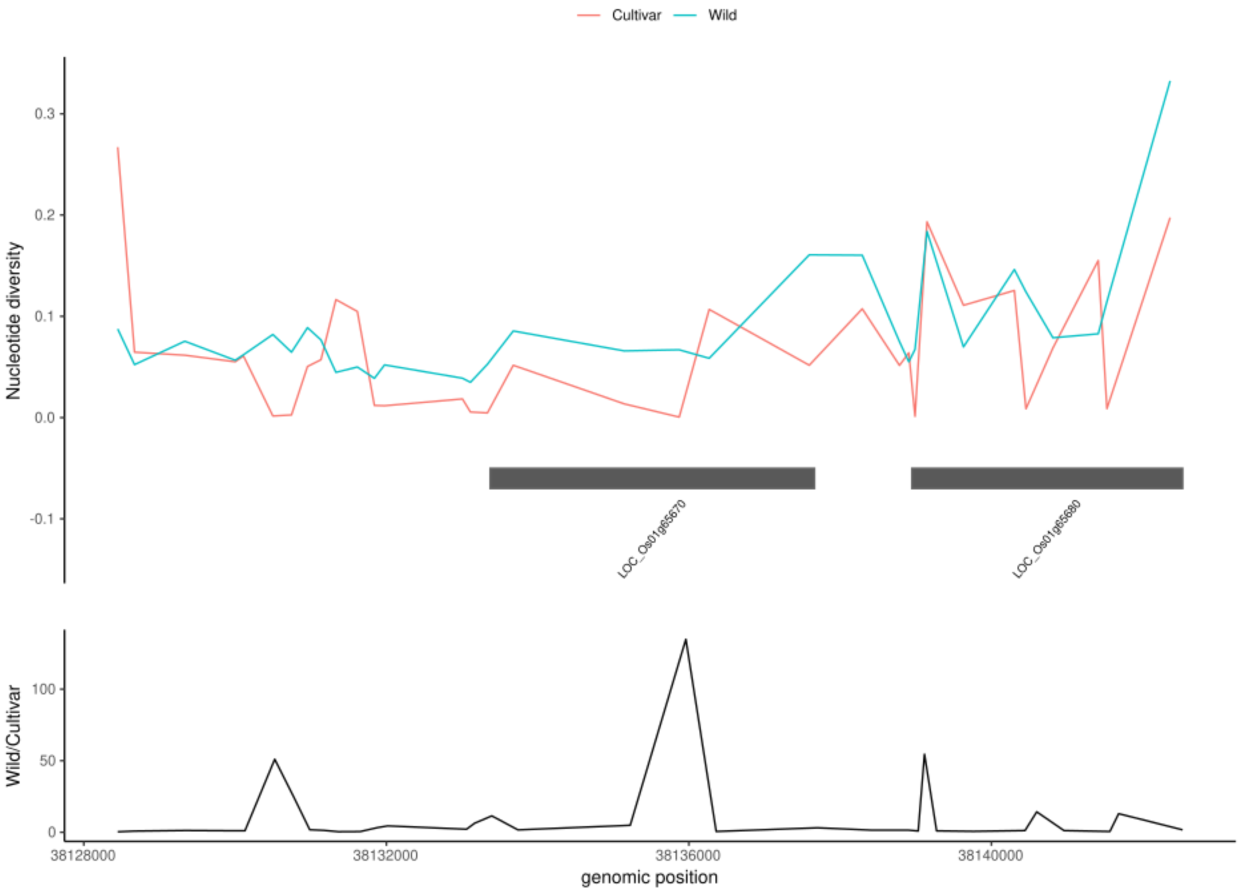

Os01g0878700

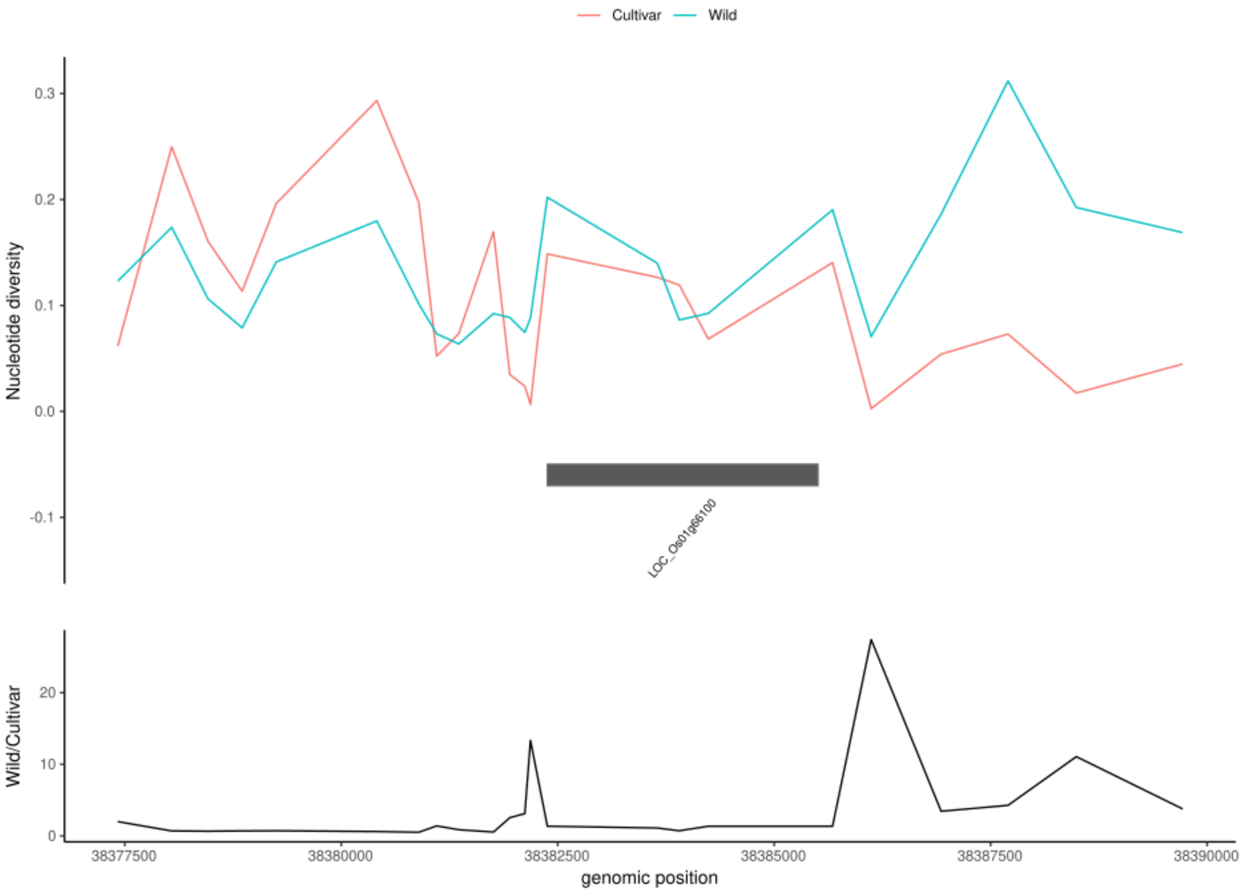

Os01g0883800

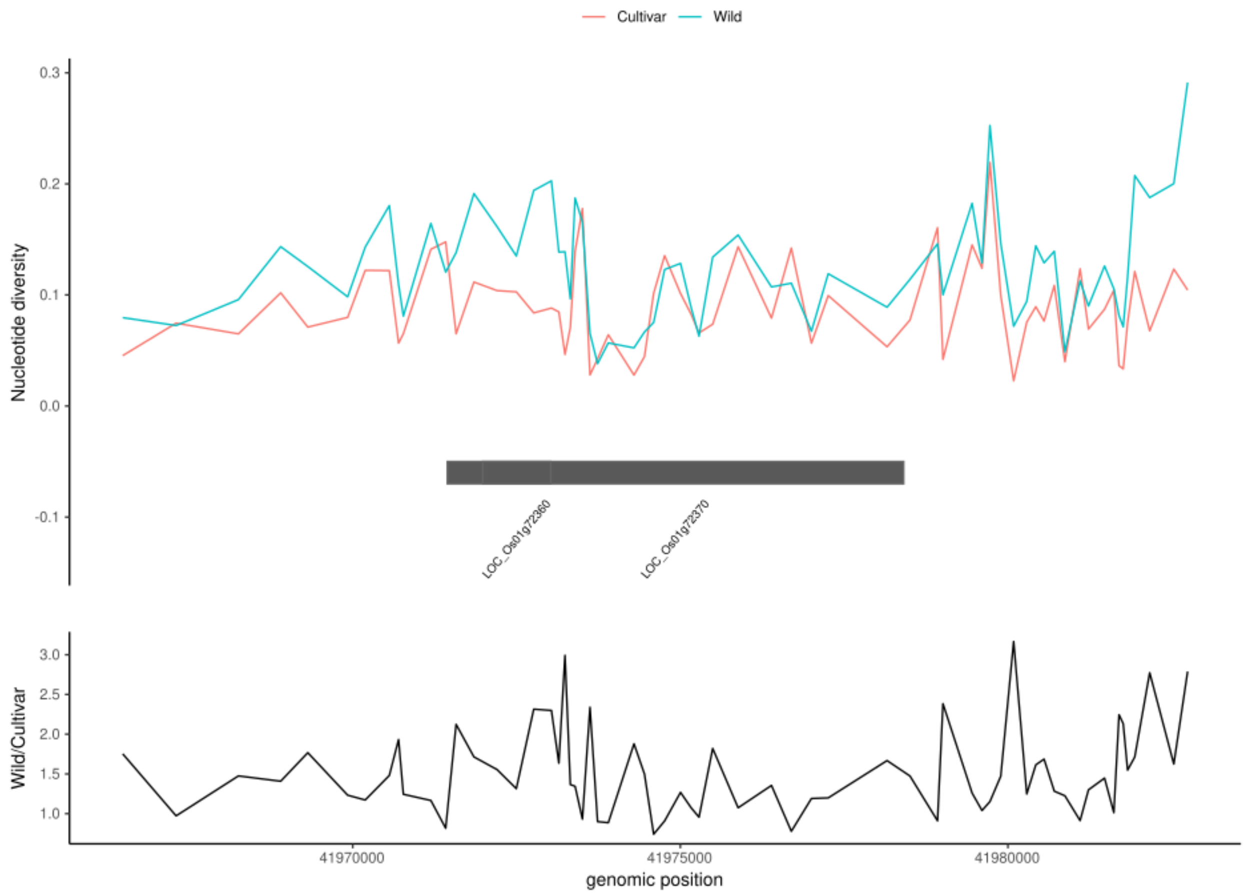

Os01g0952800

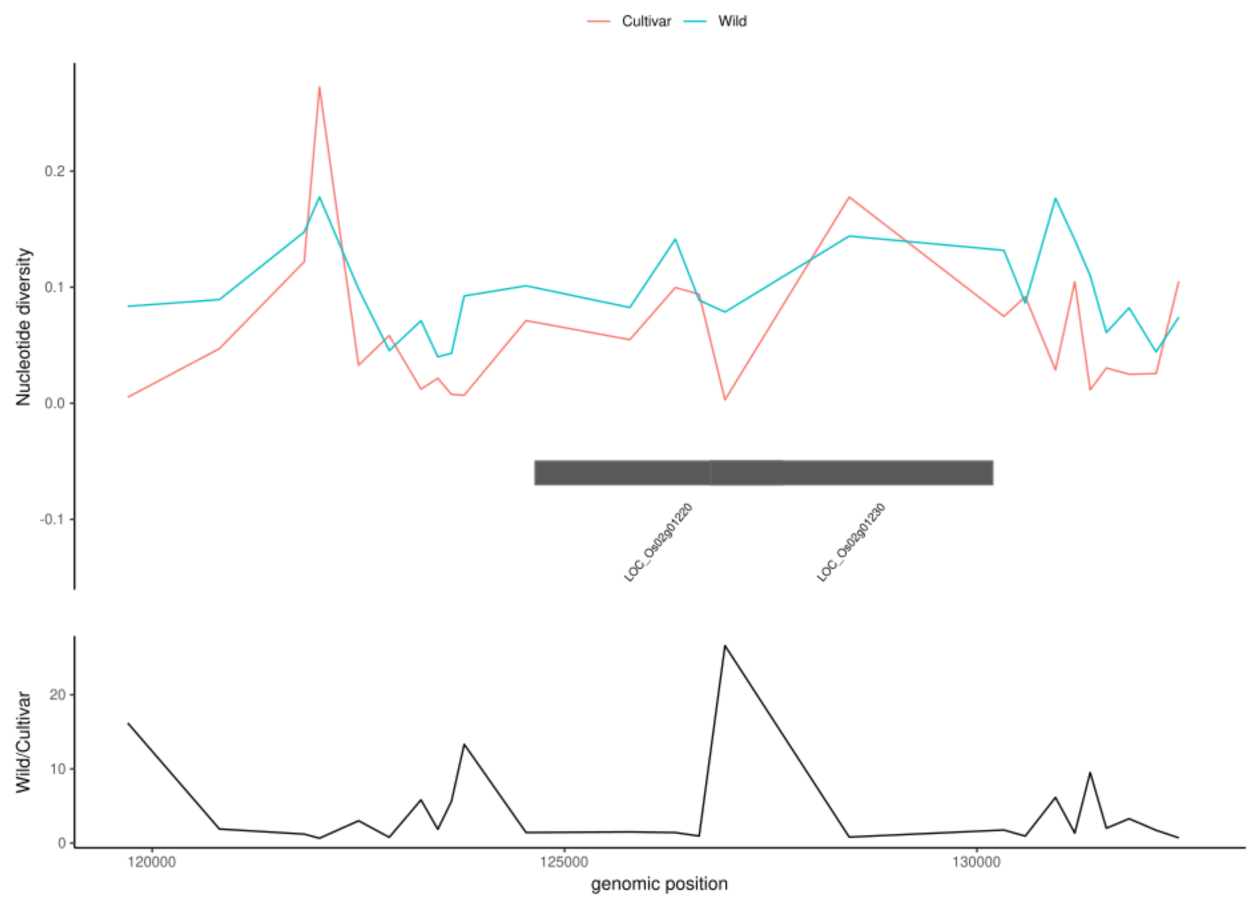

Os02g0102300

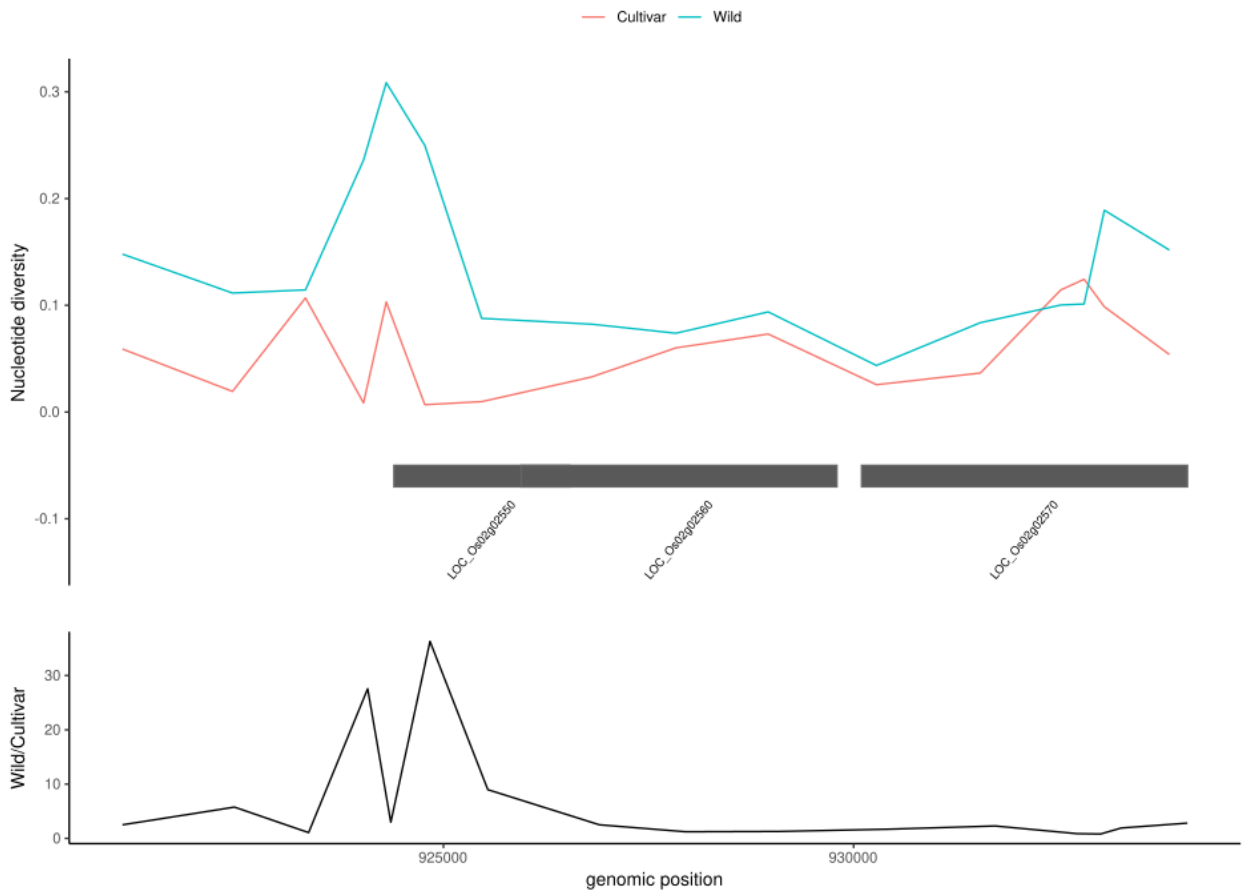

Os02g0117700

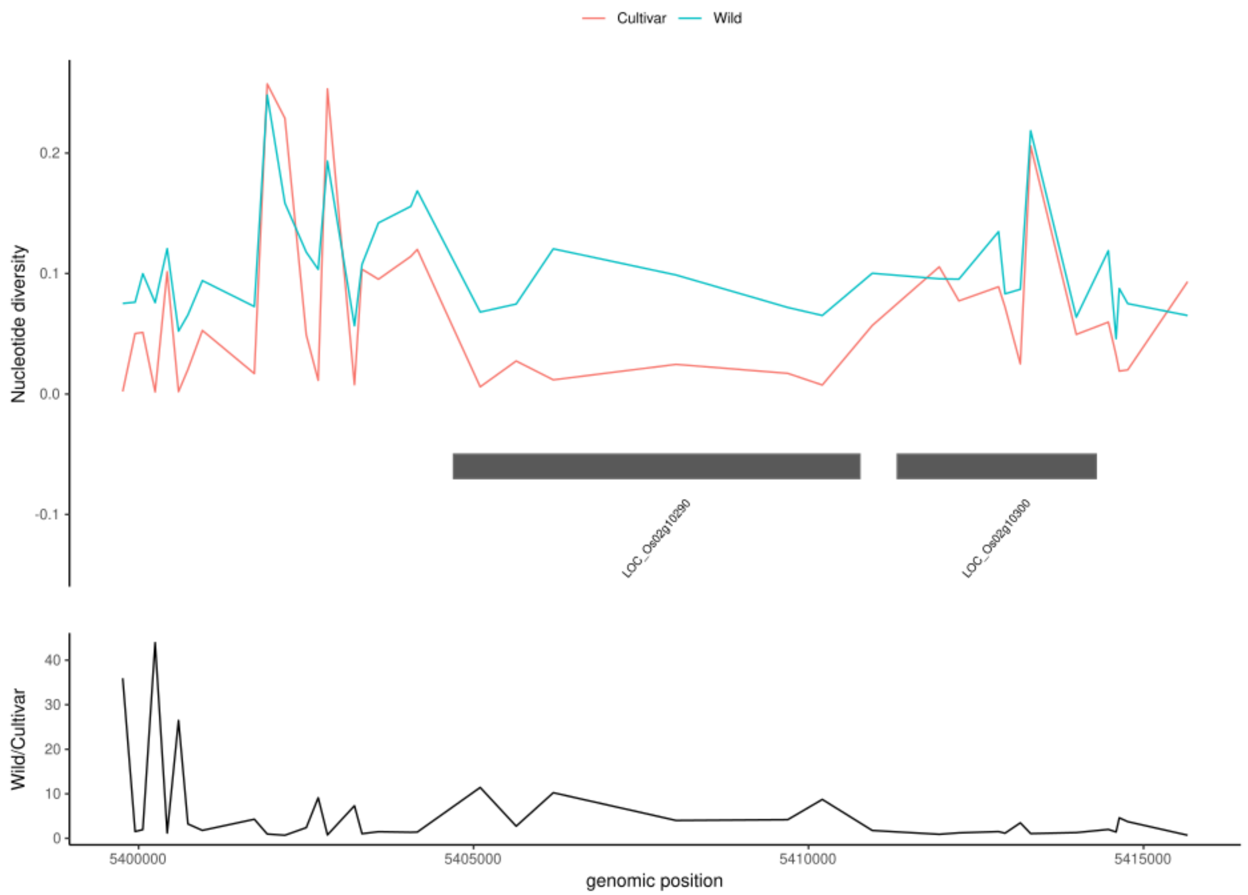

Os02g0196600

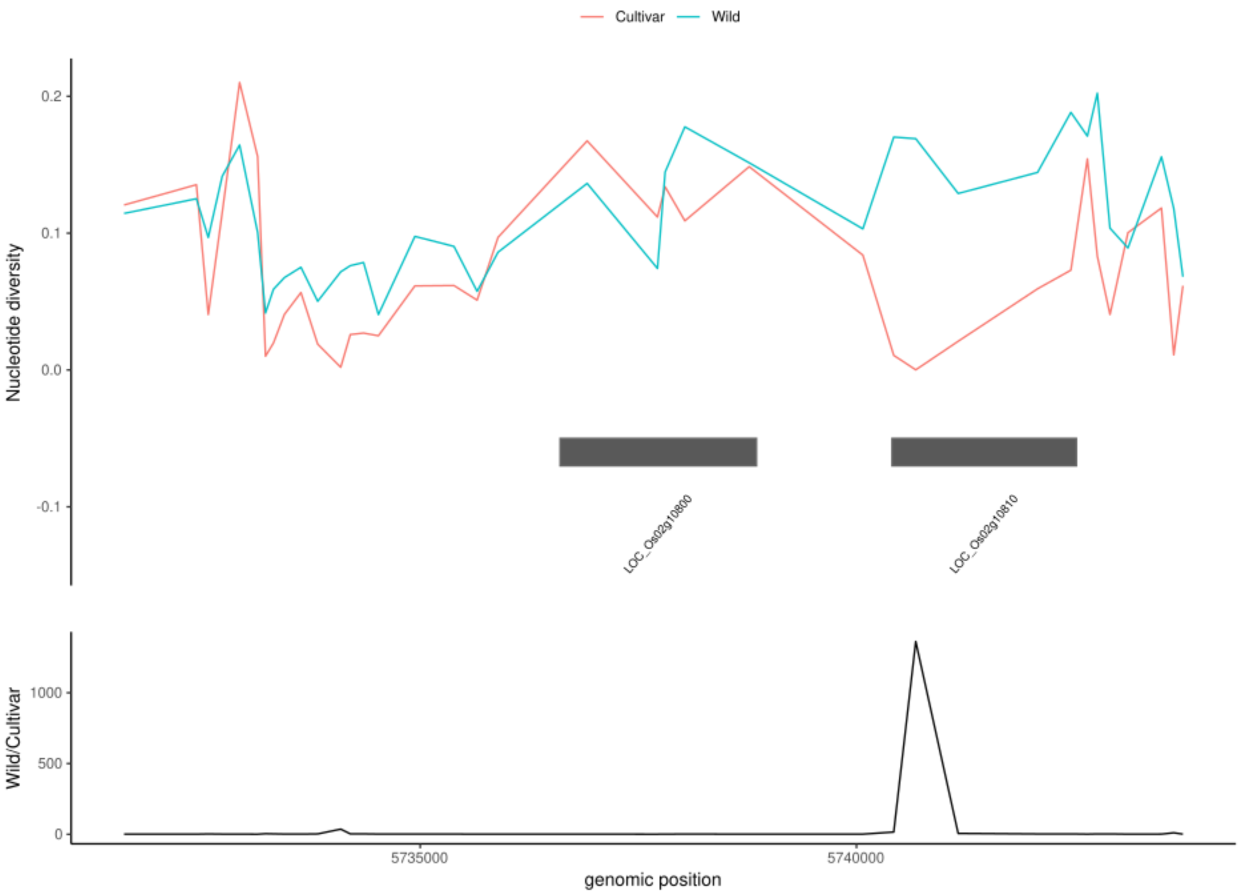

Os02g0202400

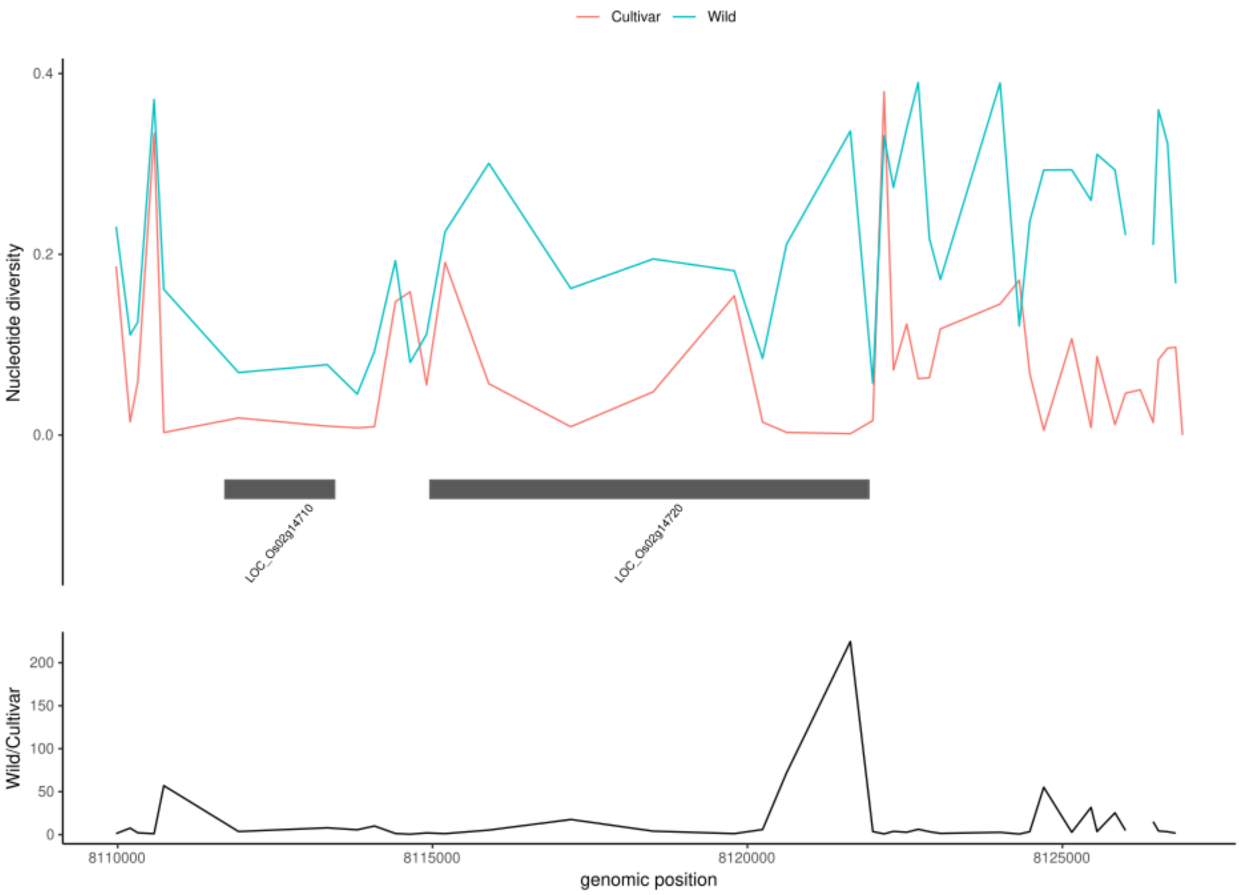

Os02g0244100

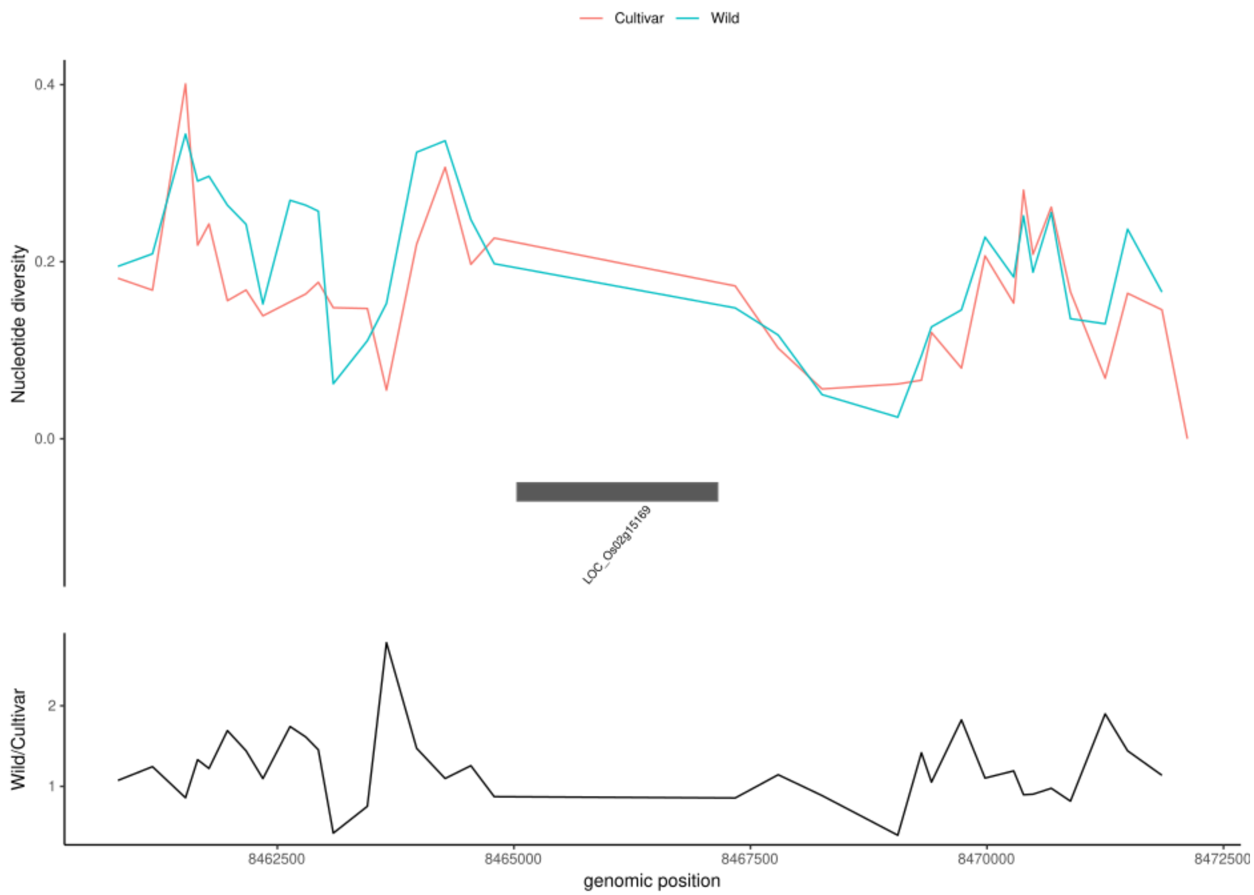

Os02g0249900

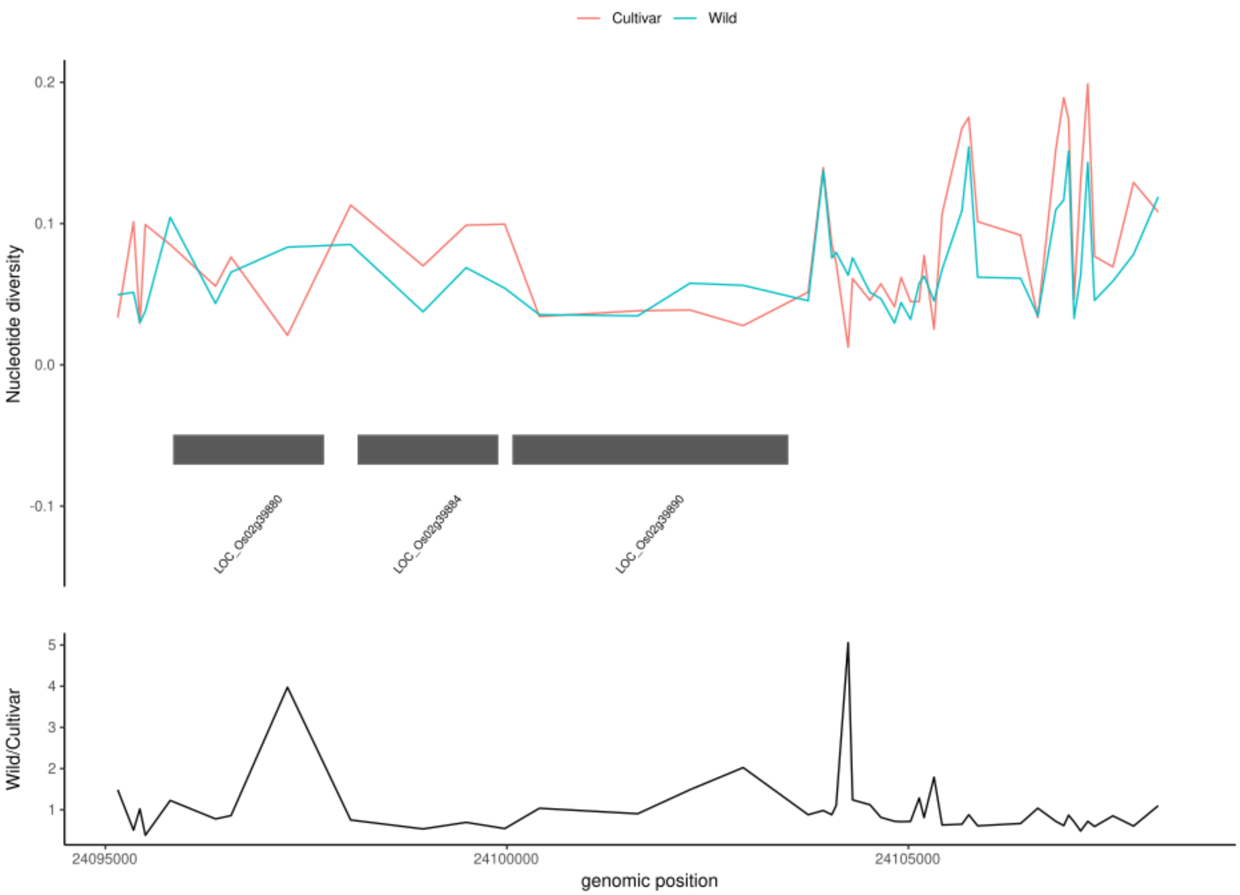

Os02g0612300

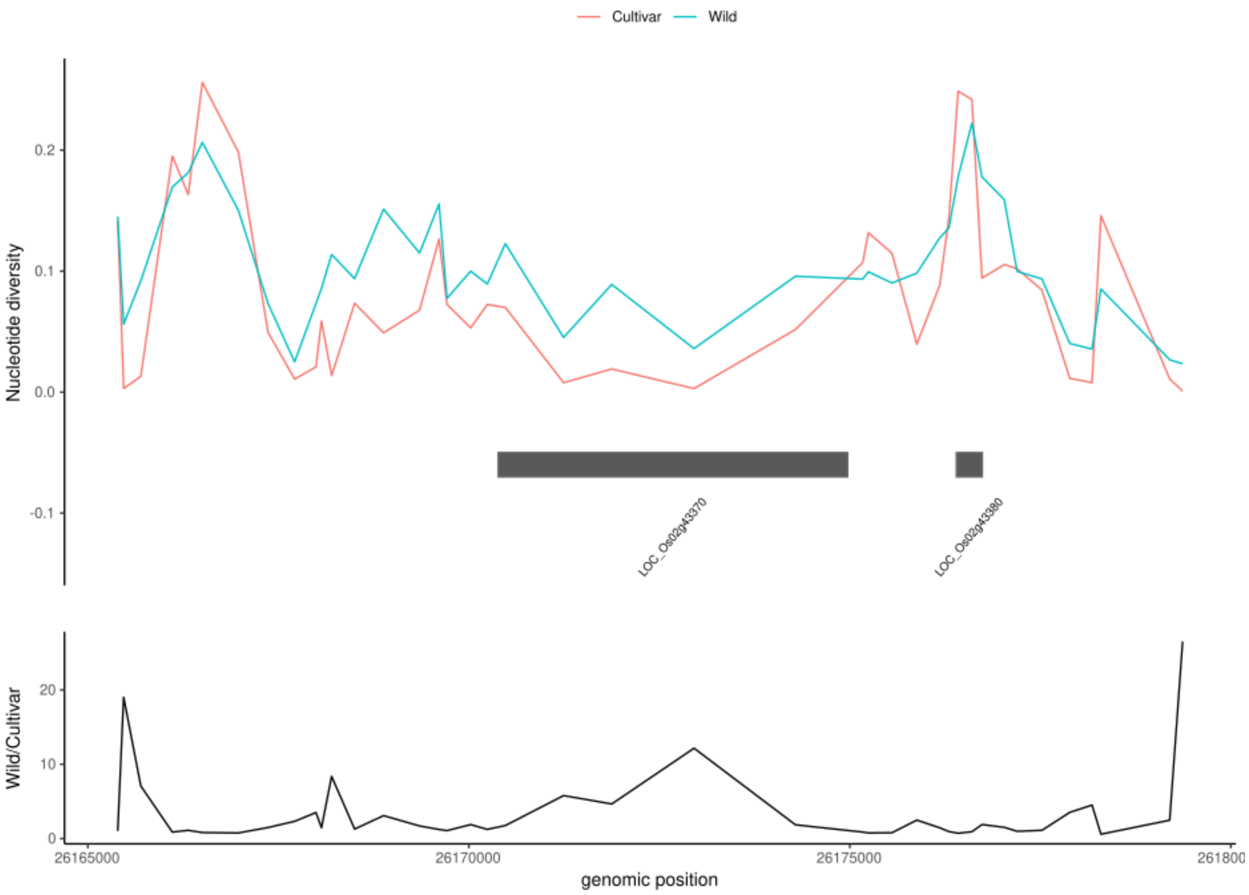

Os02g0649900

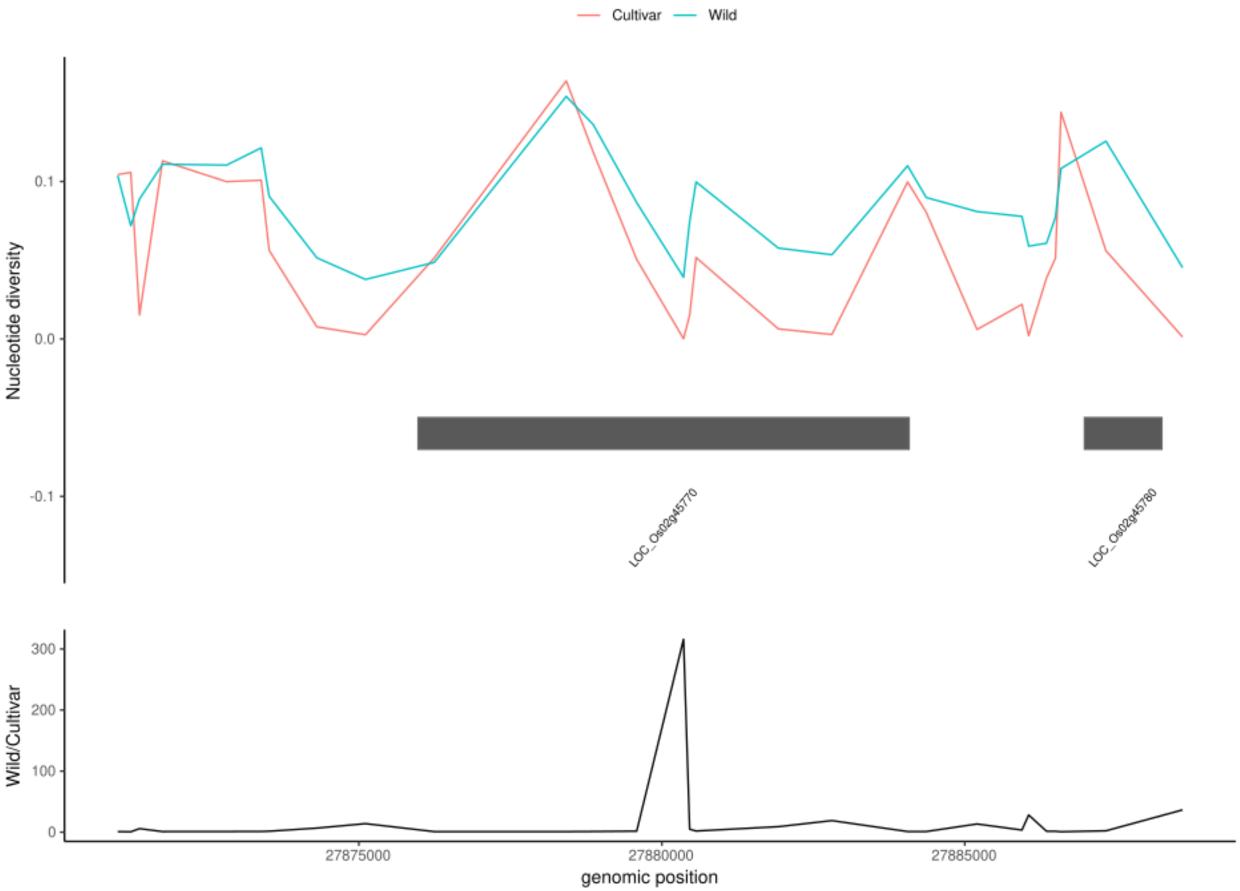

Os02g0682200

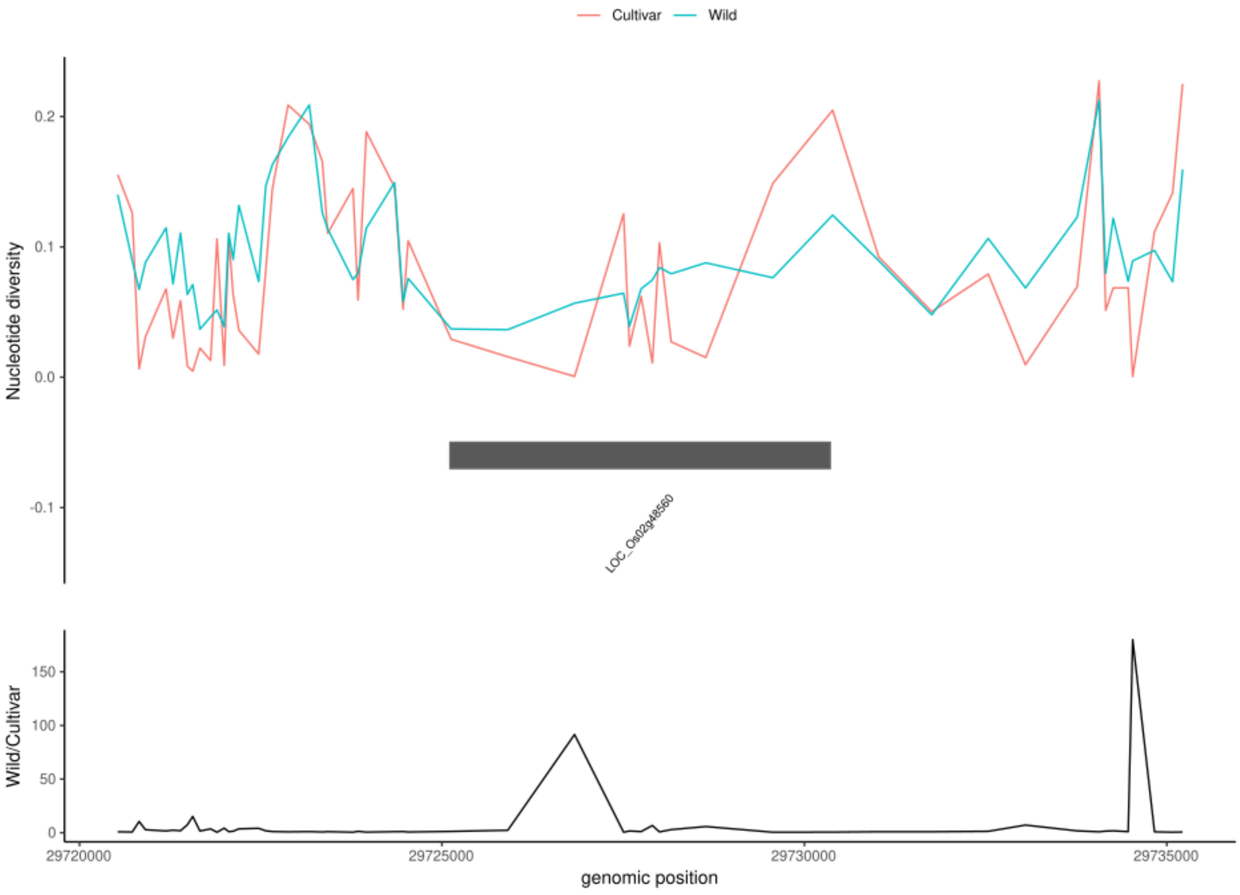

Os02g0716500

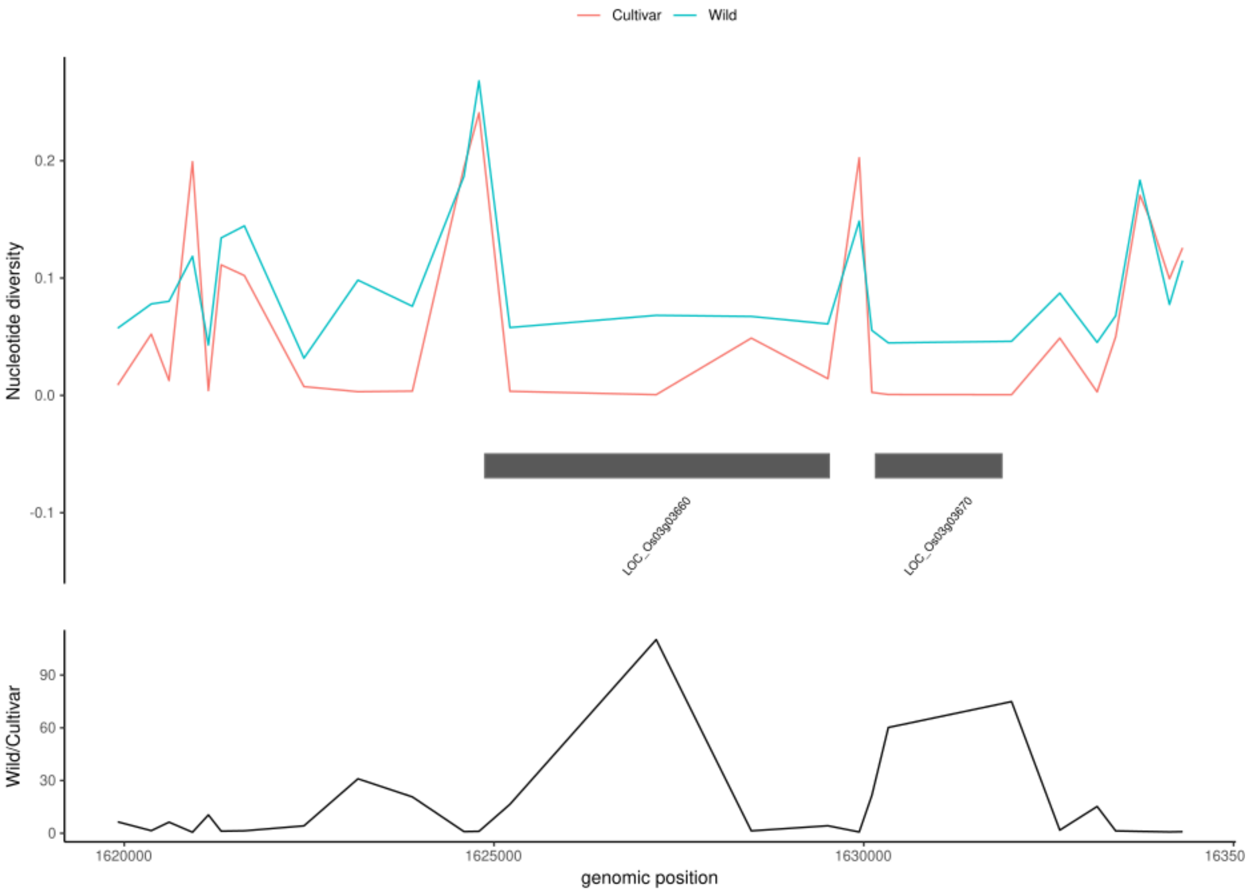

Os03g0128700

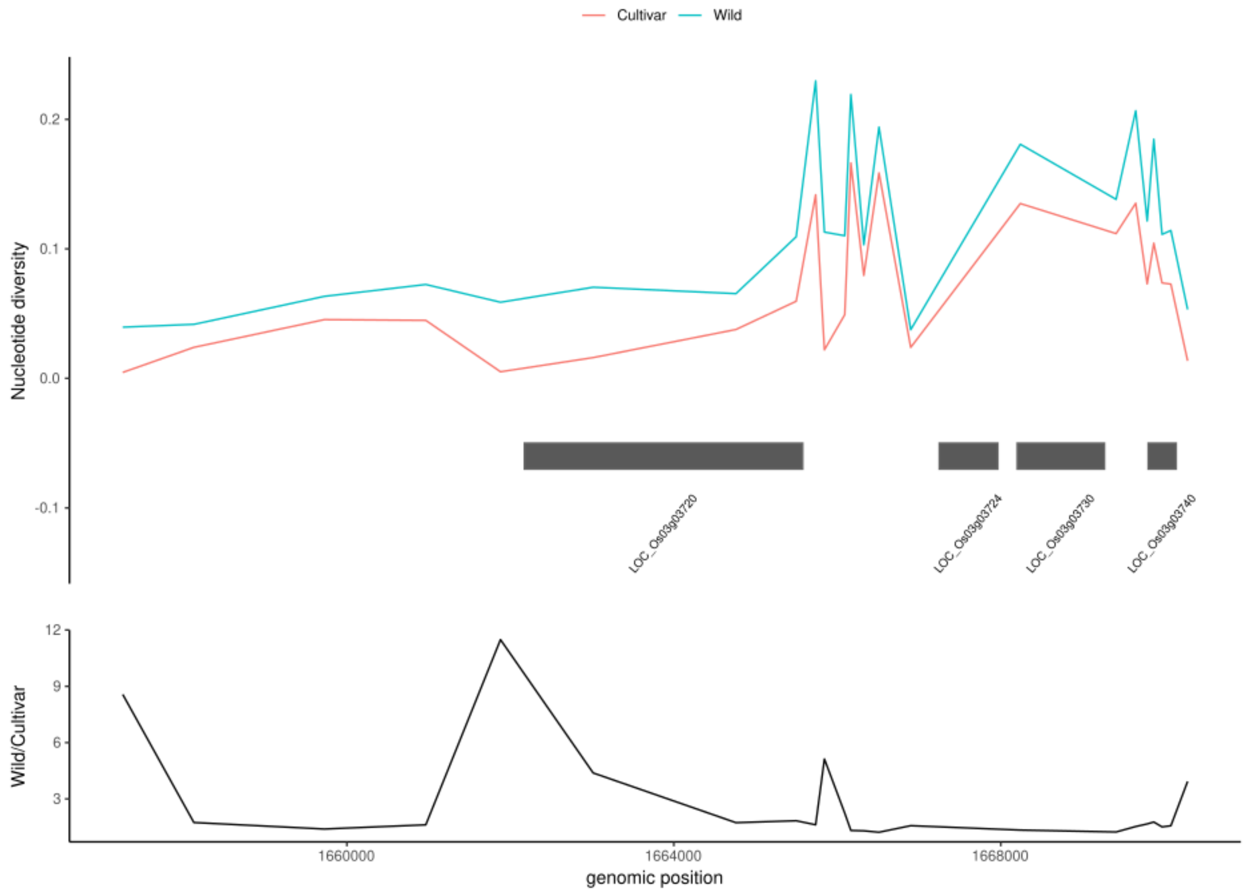

Os03g0129300

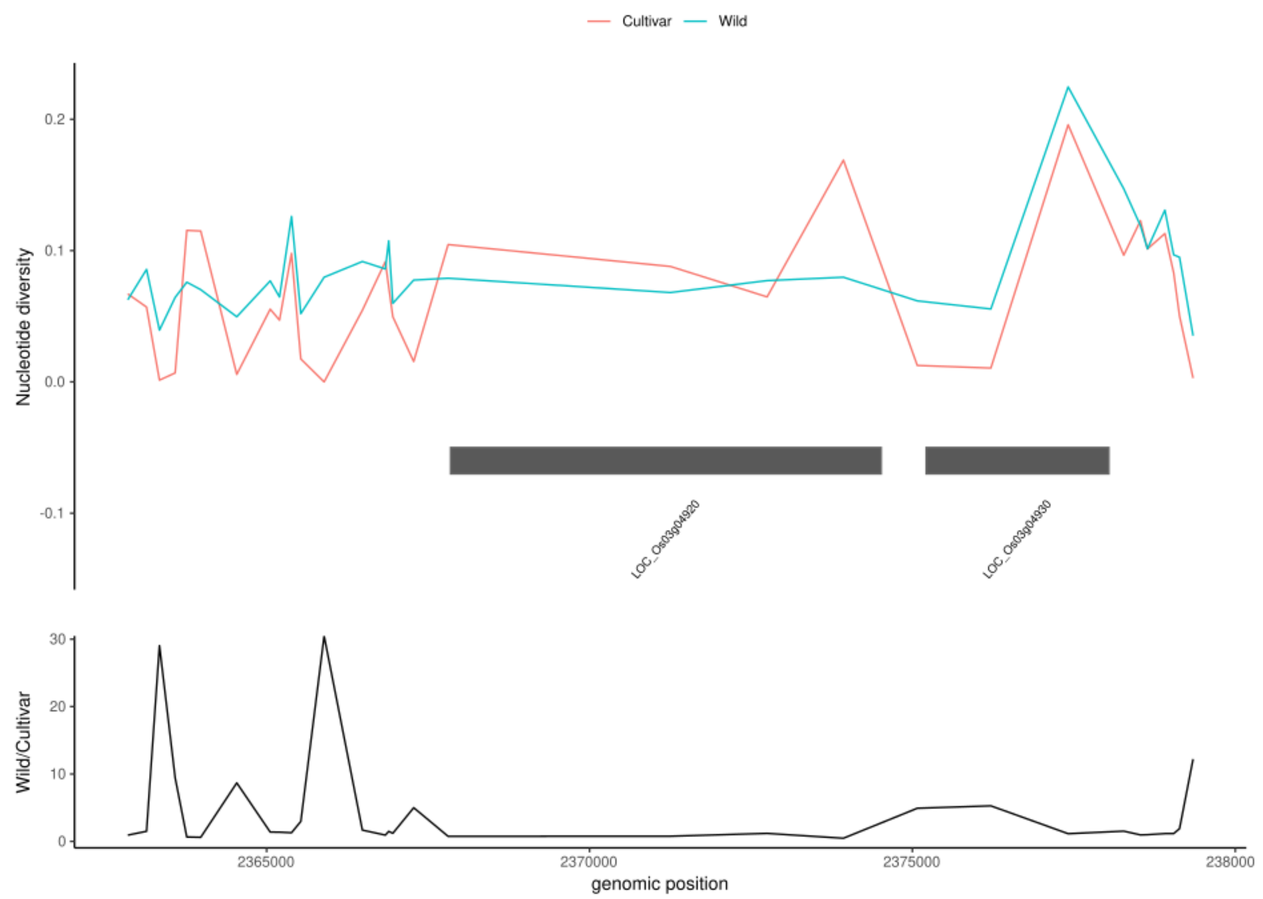

Os03g0142800

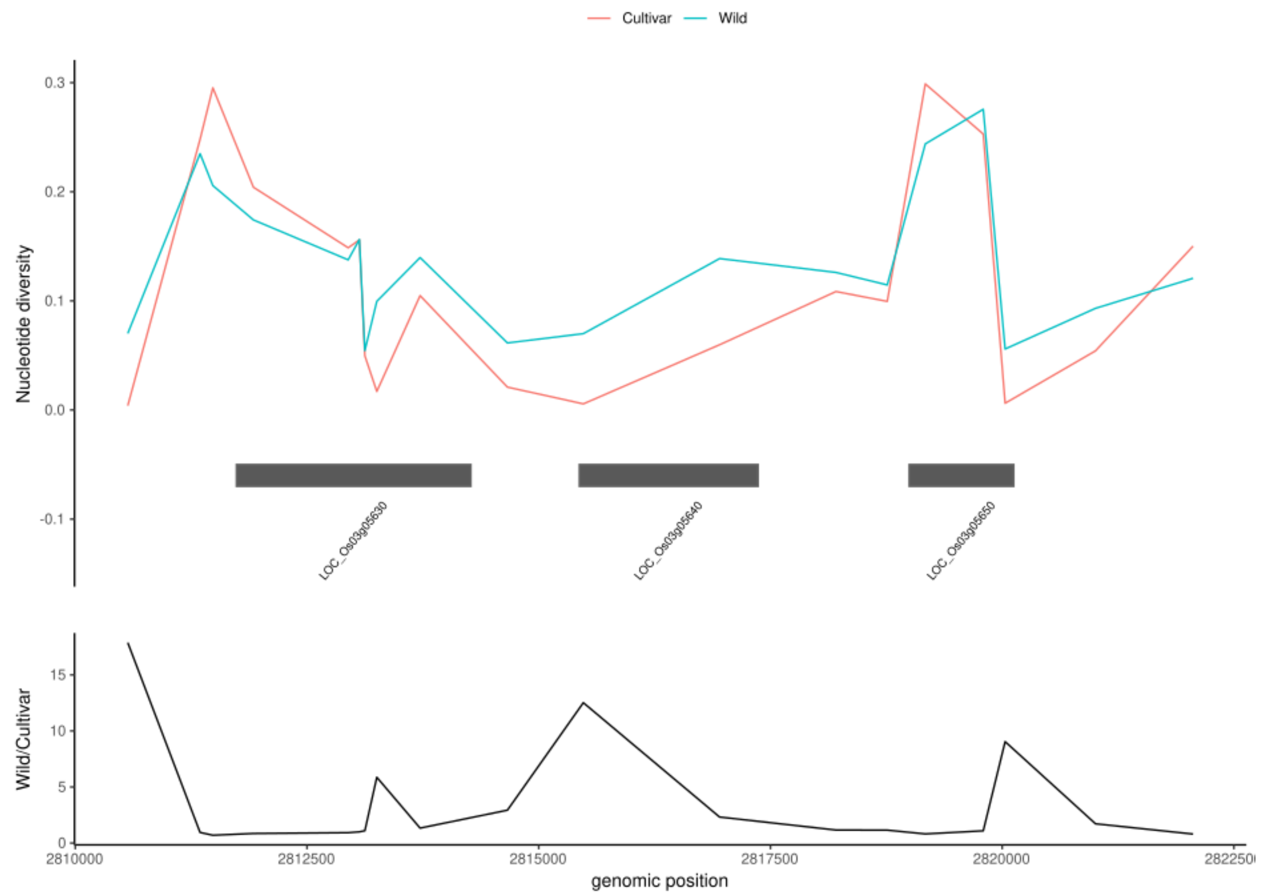

Os03g0150800

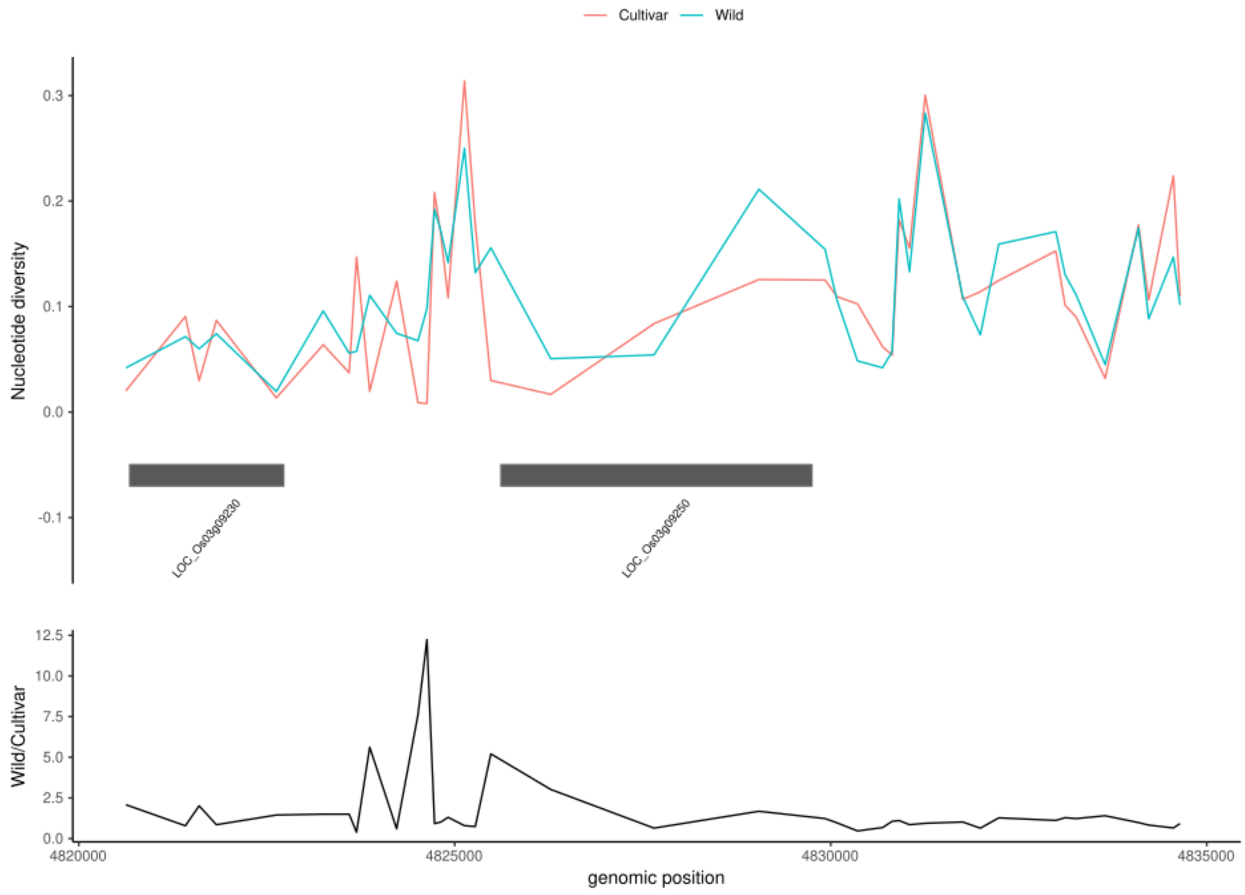

Os03g0192700

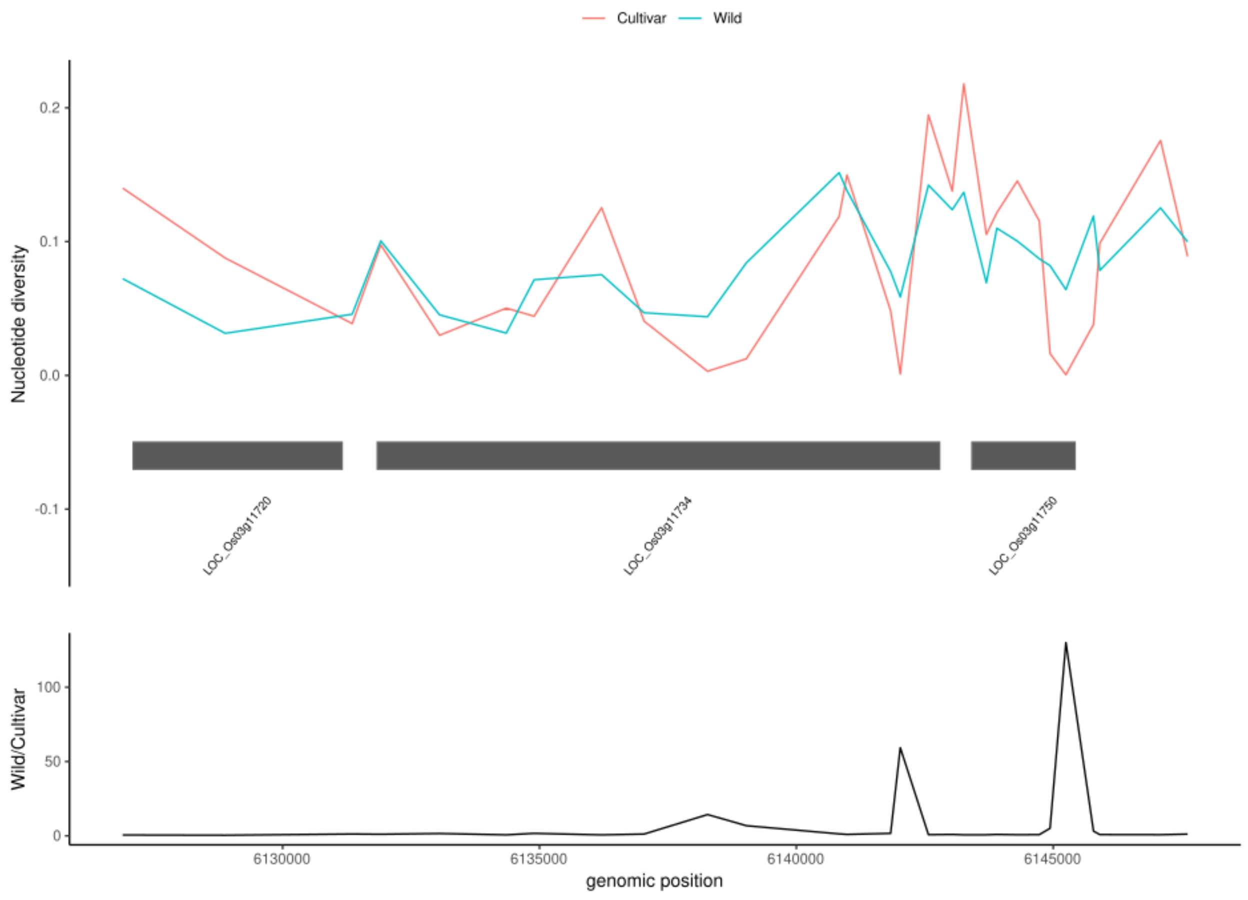

Os03g0216700

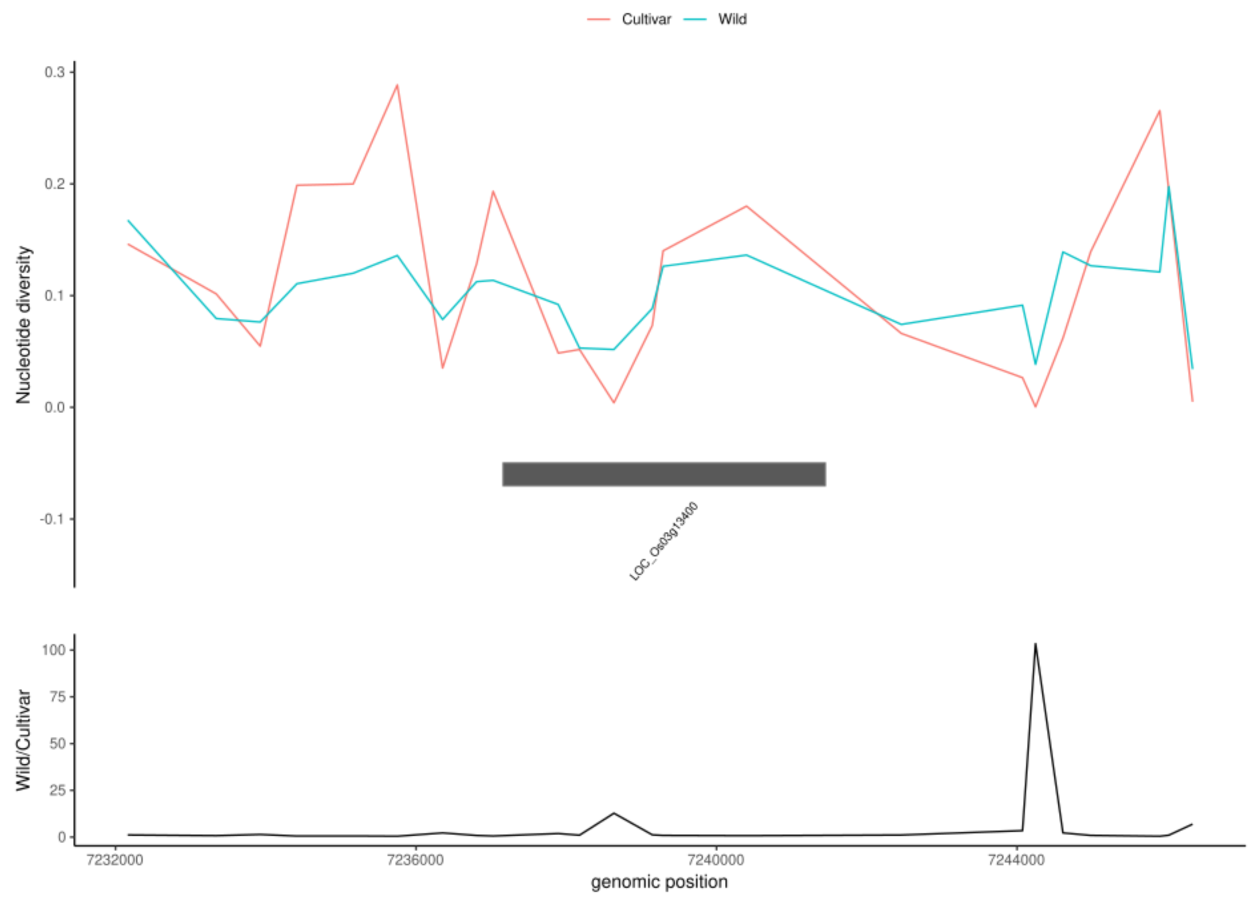

Os03g0237250

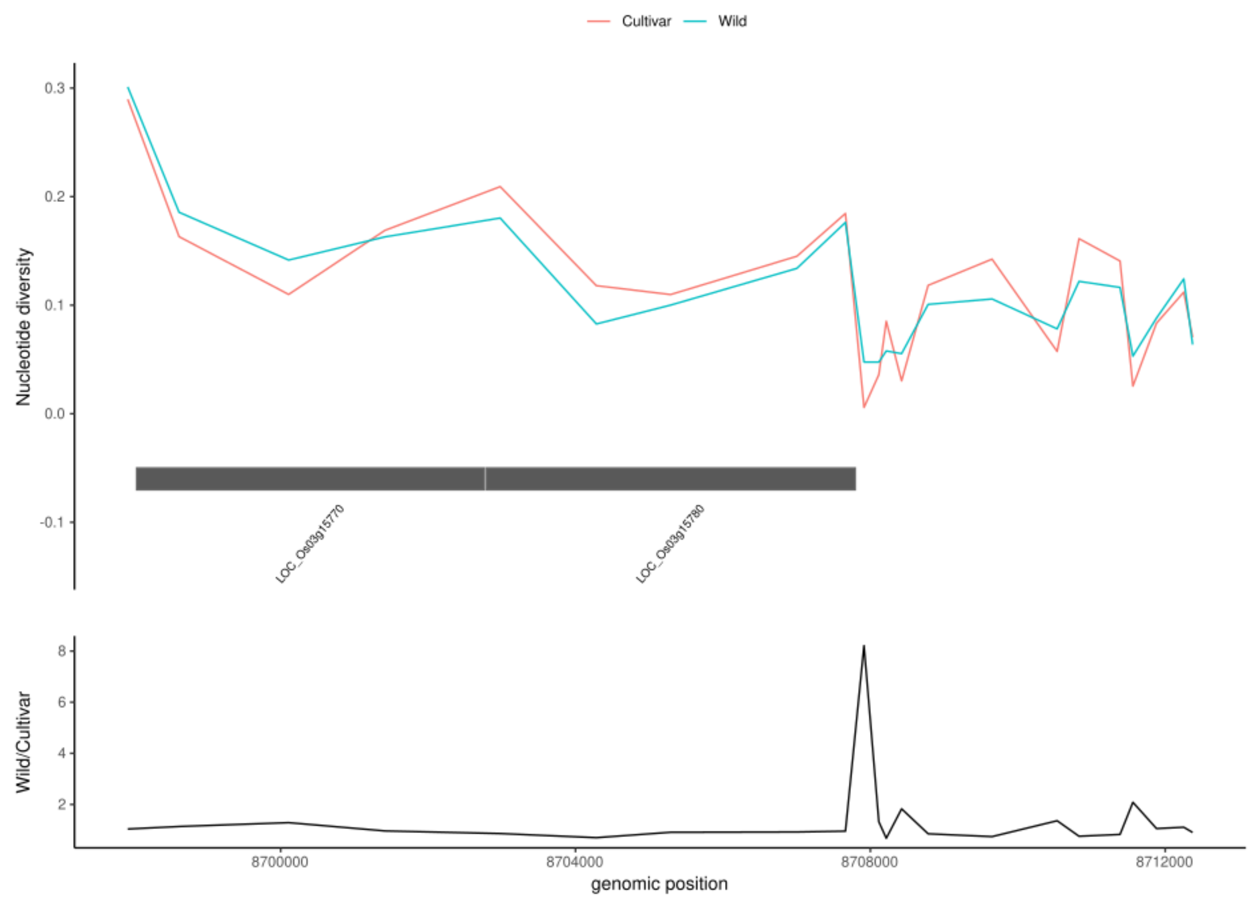

Os03g0264400

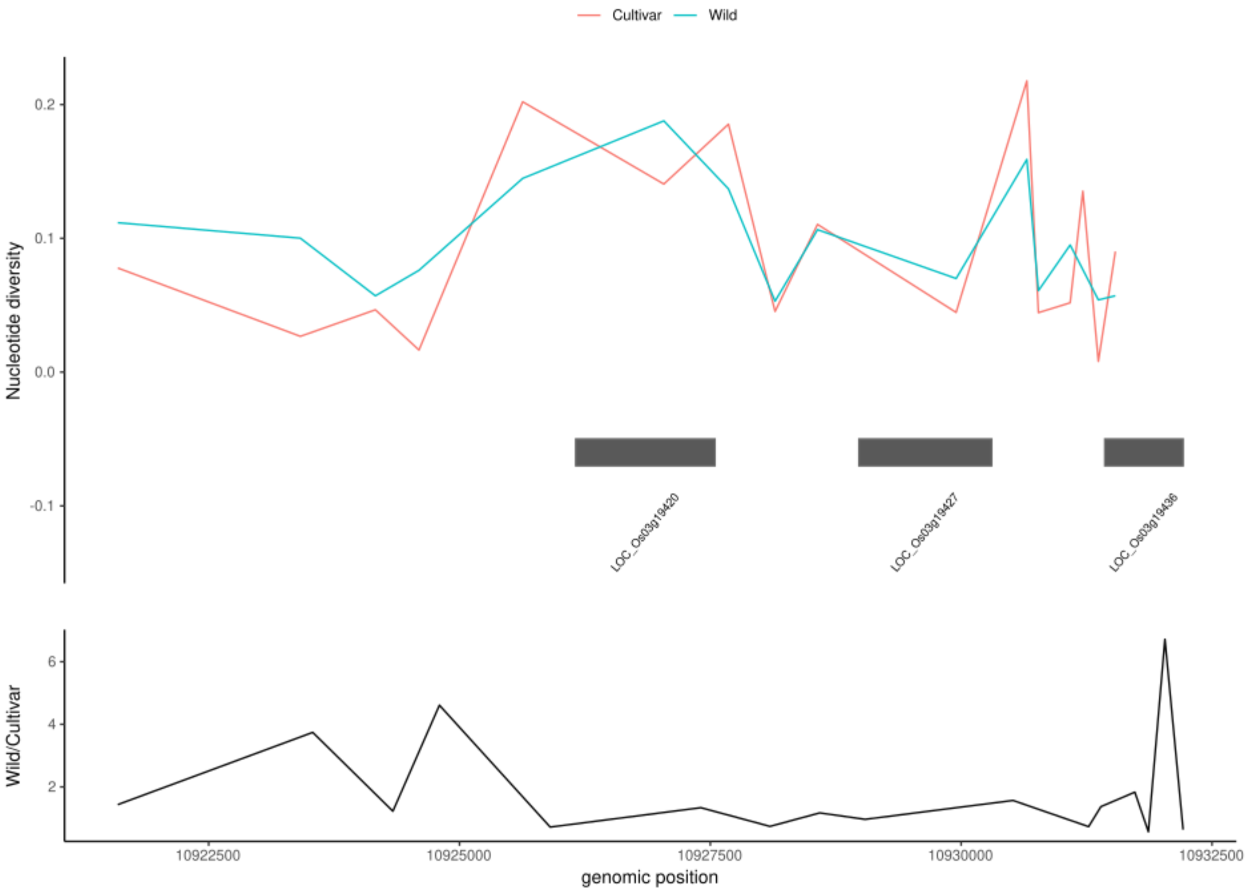

Os03g0307200

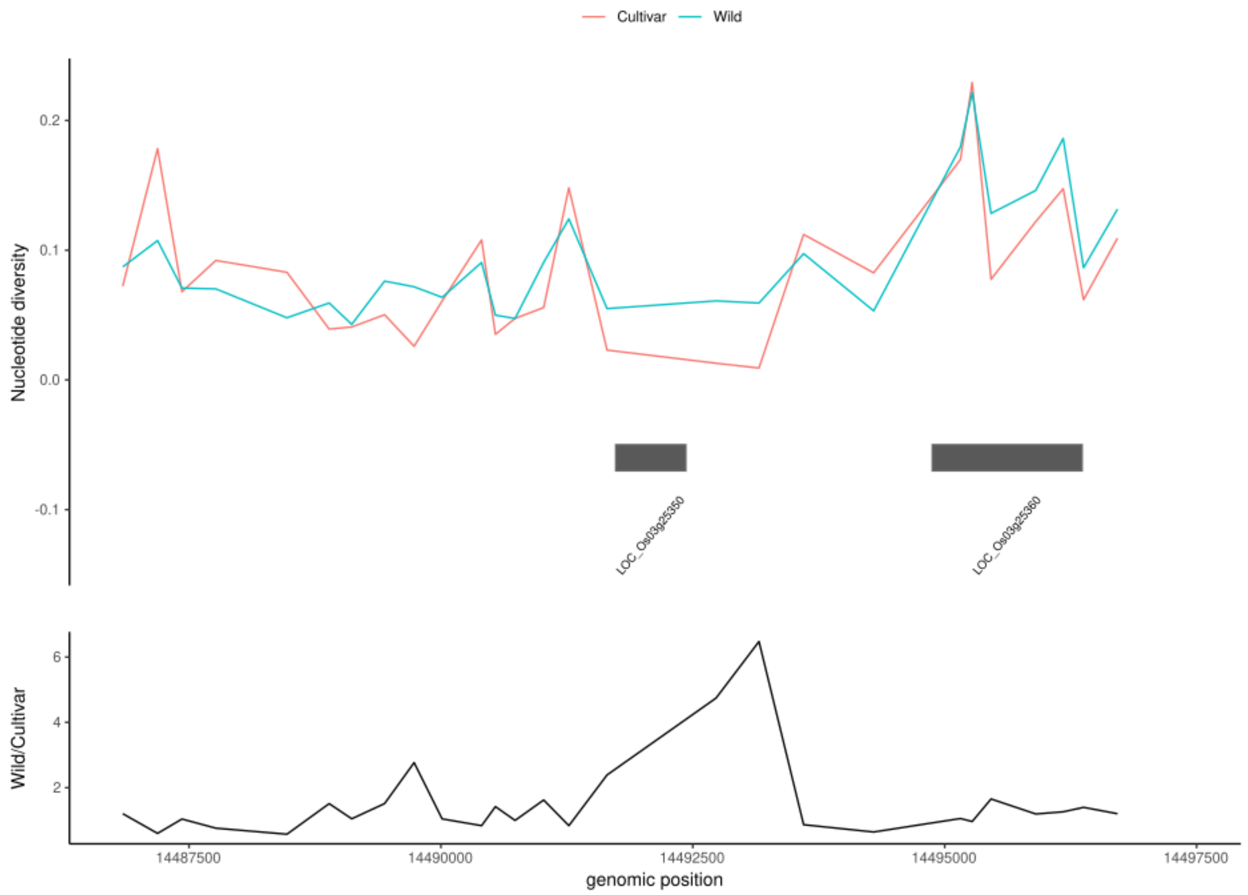

Os03g0369100

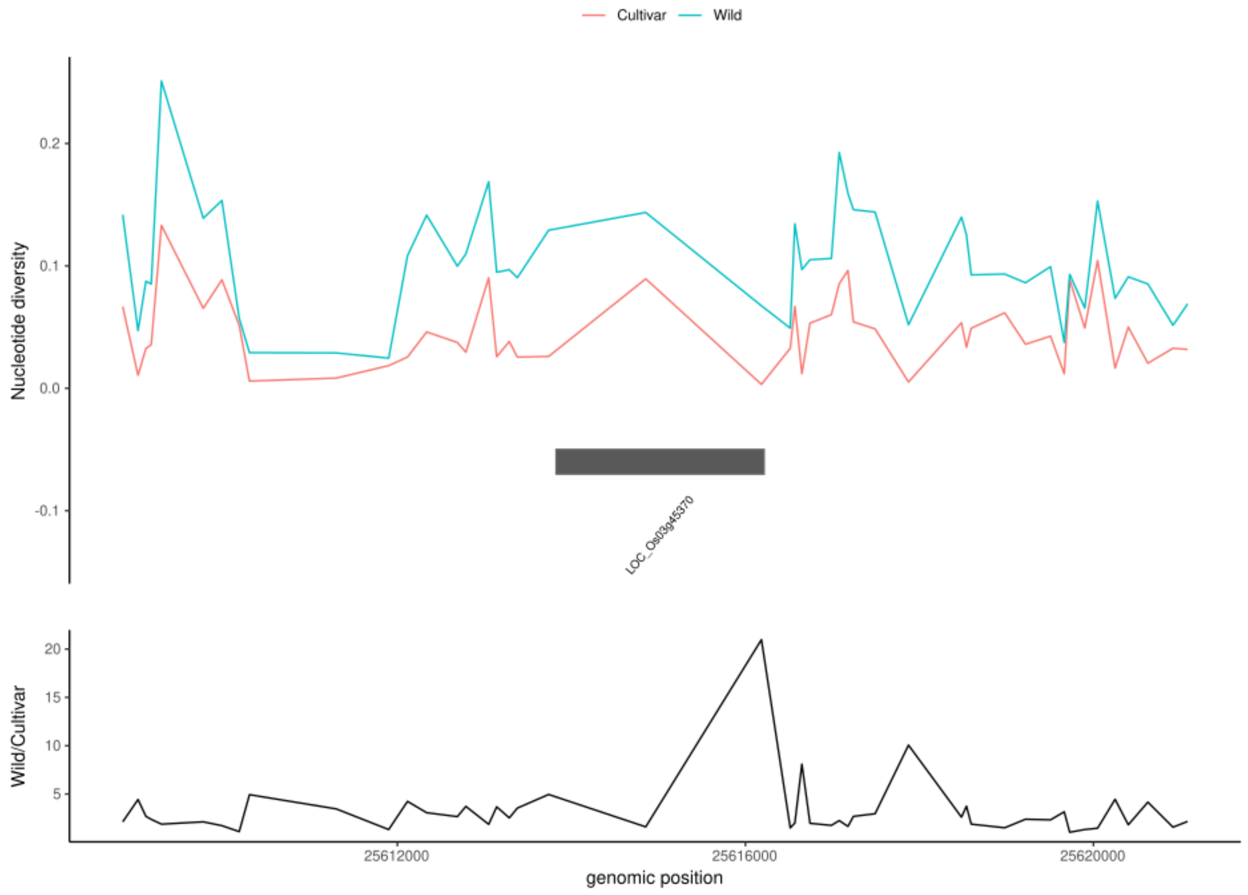

Os03g0656500

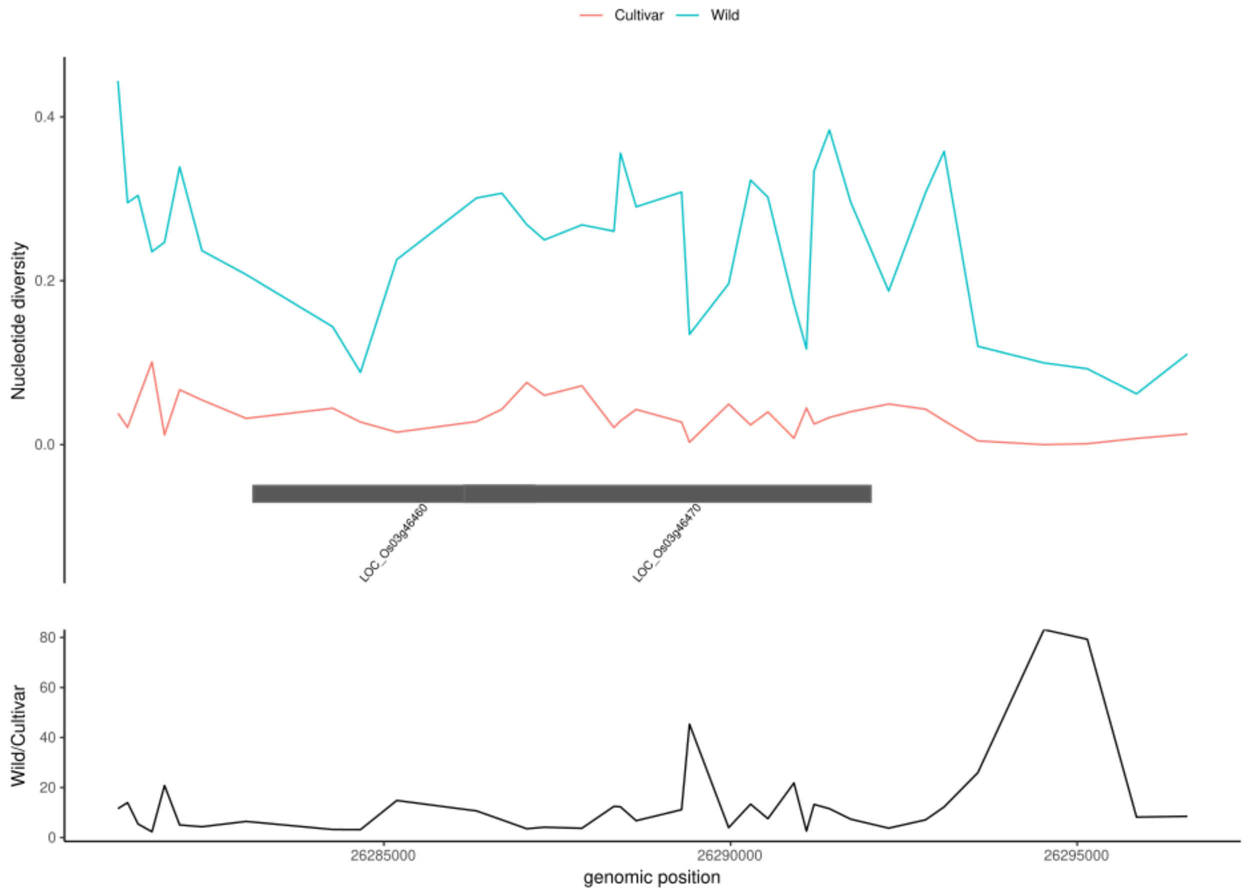

Os03g0667500

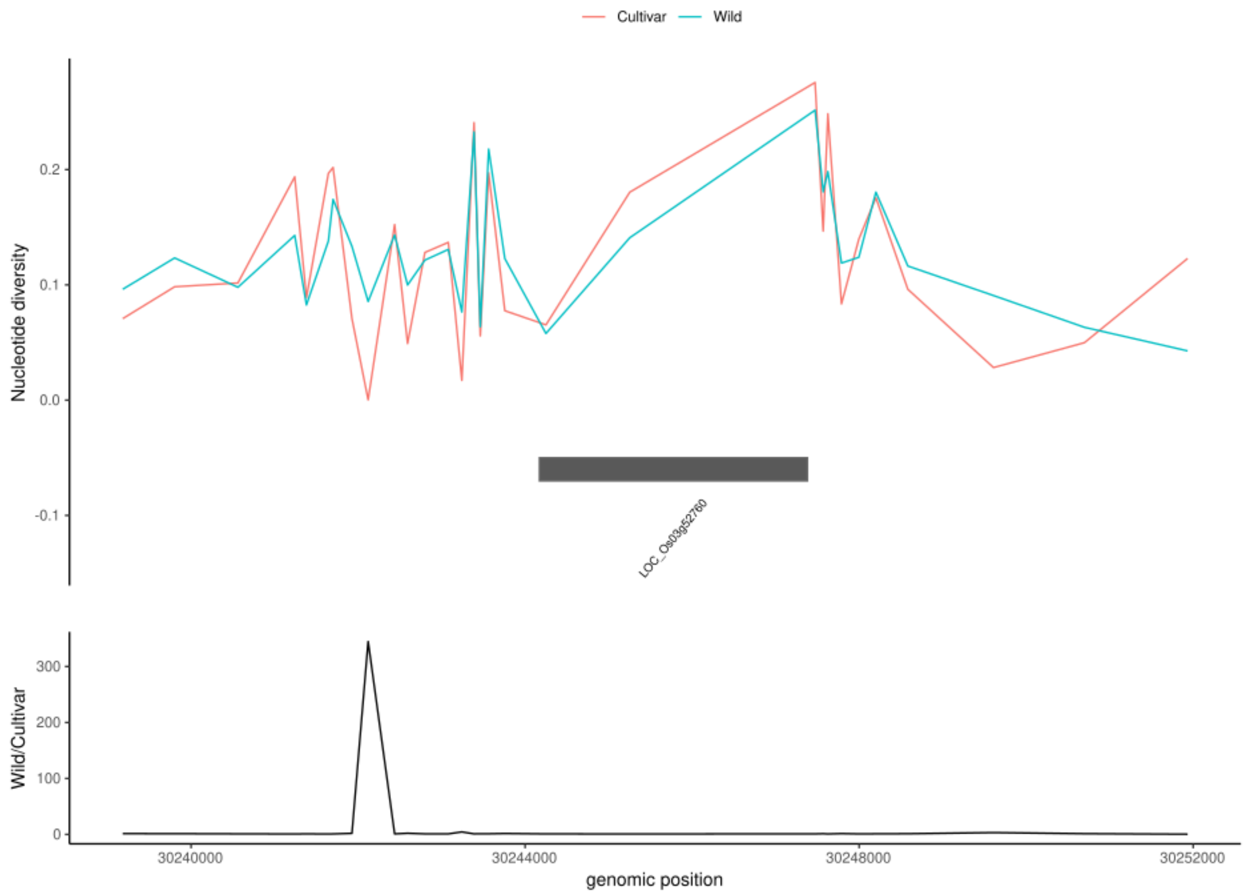

Os03g0737701

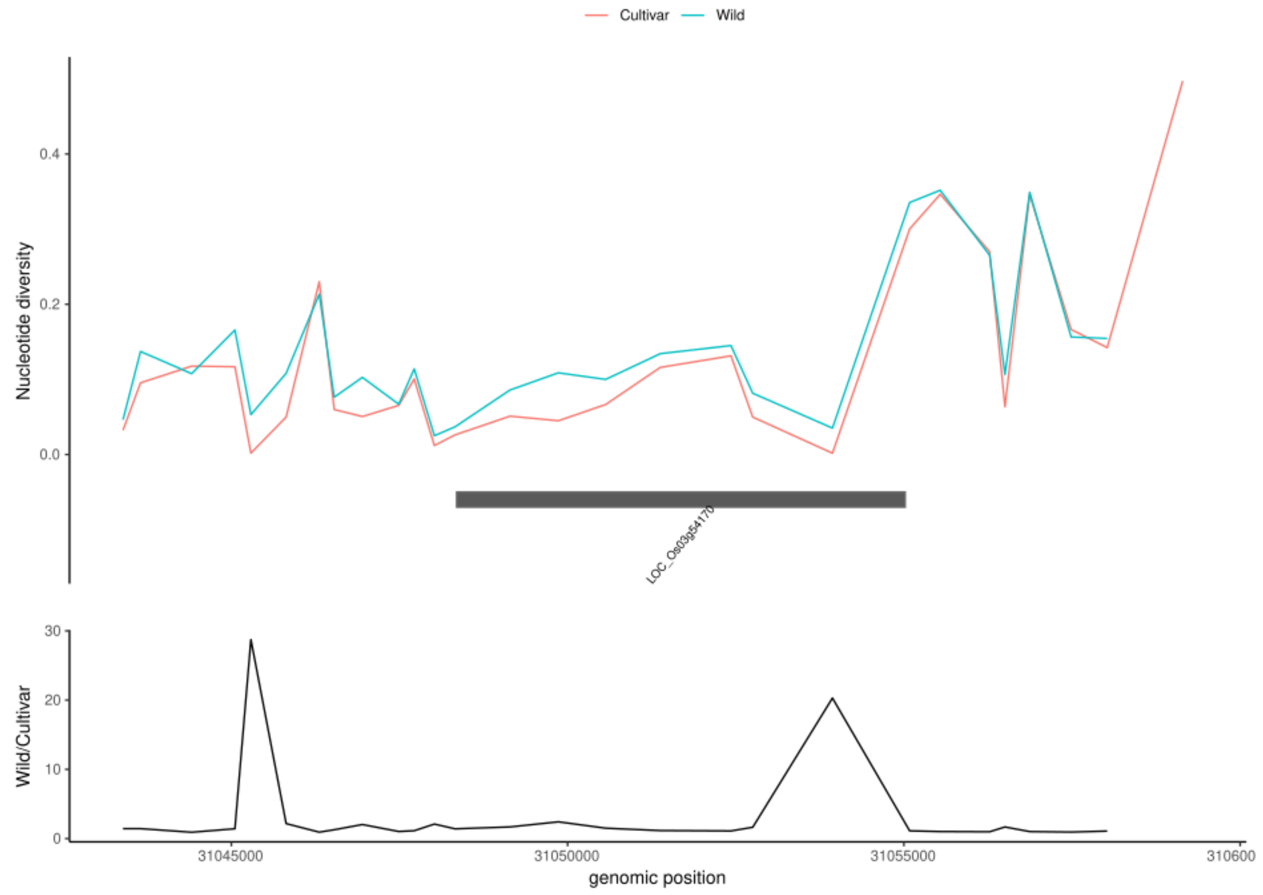

Os03g0753100

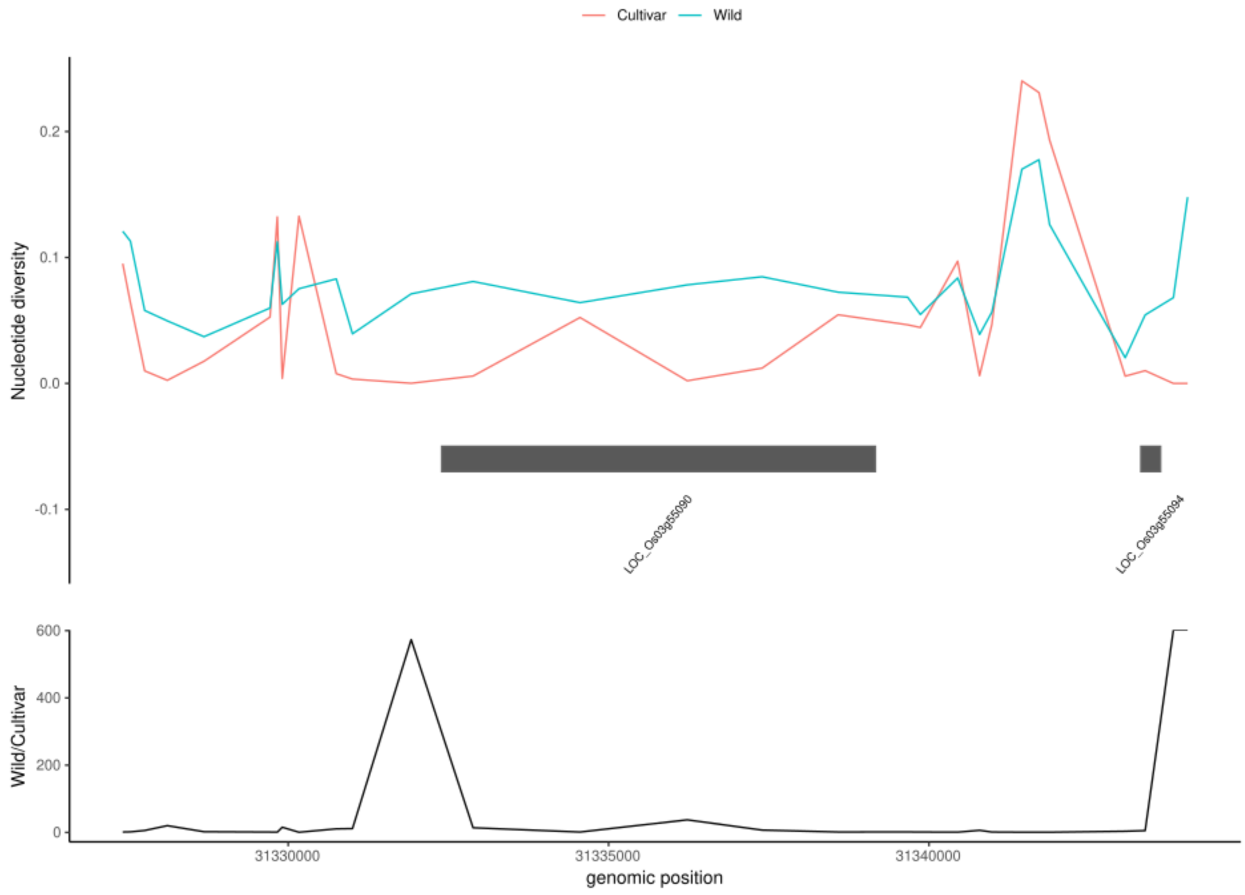

Os03g0758100

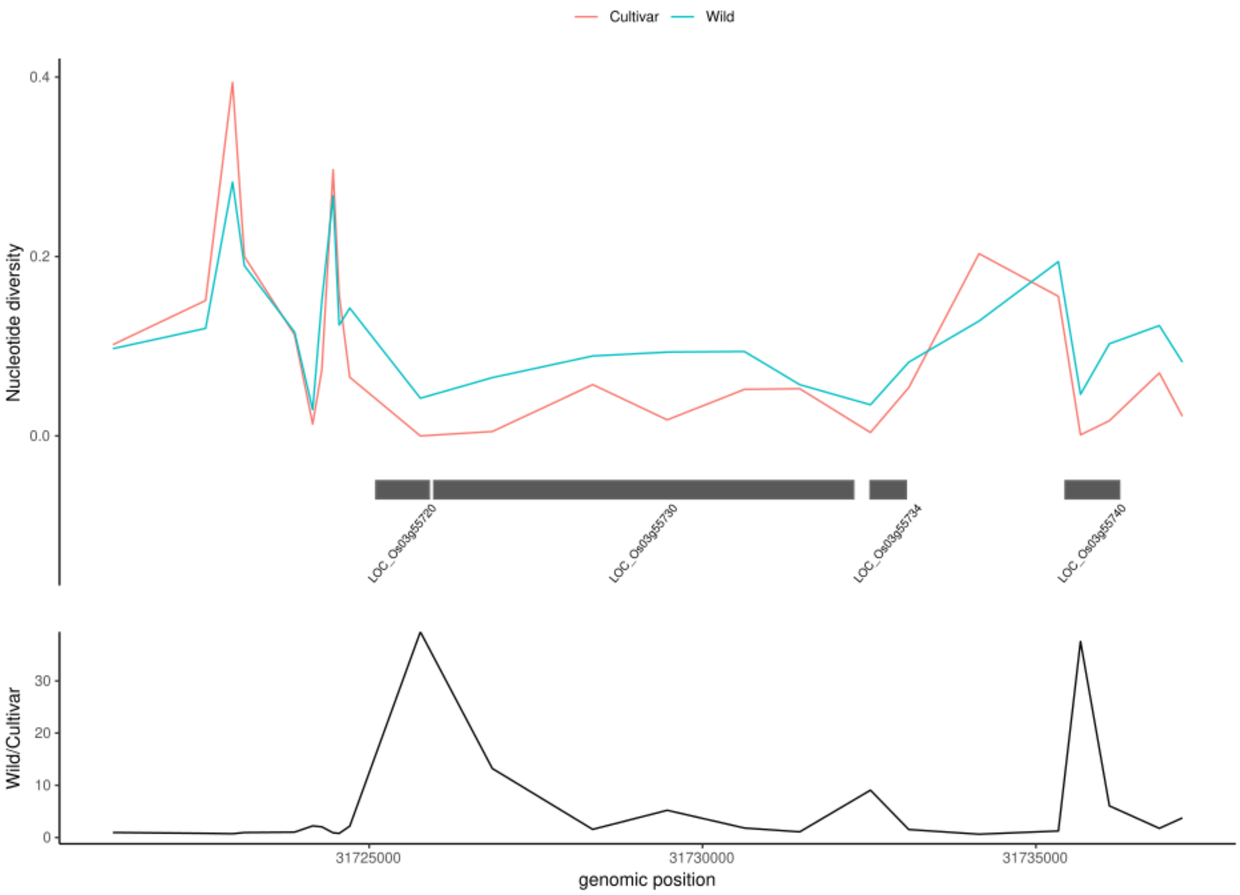

Os03g0766000

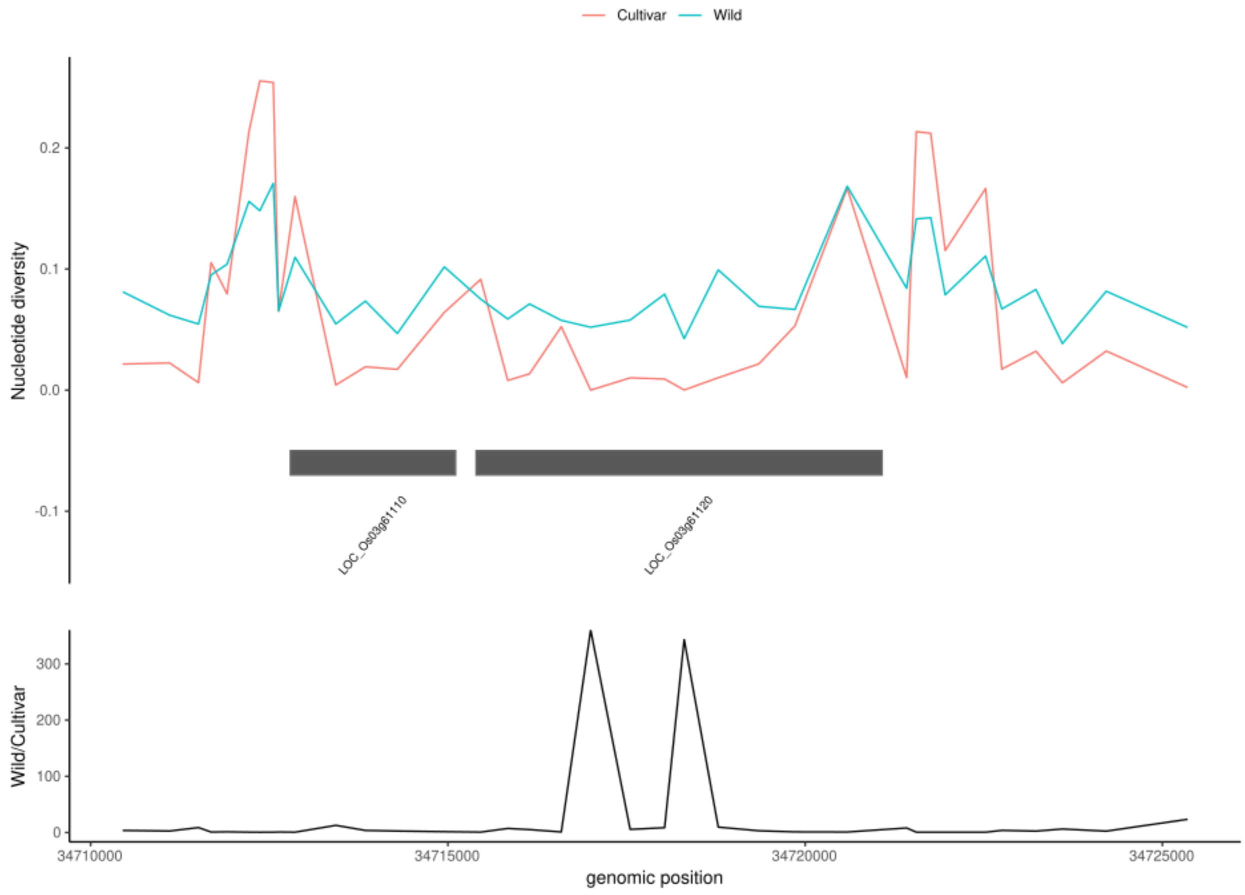

Os03g0826500

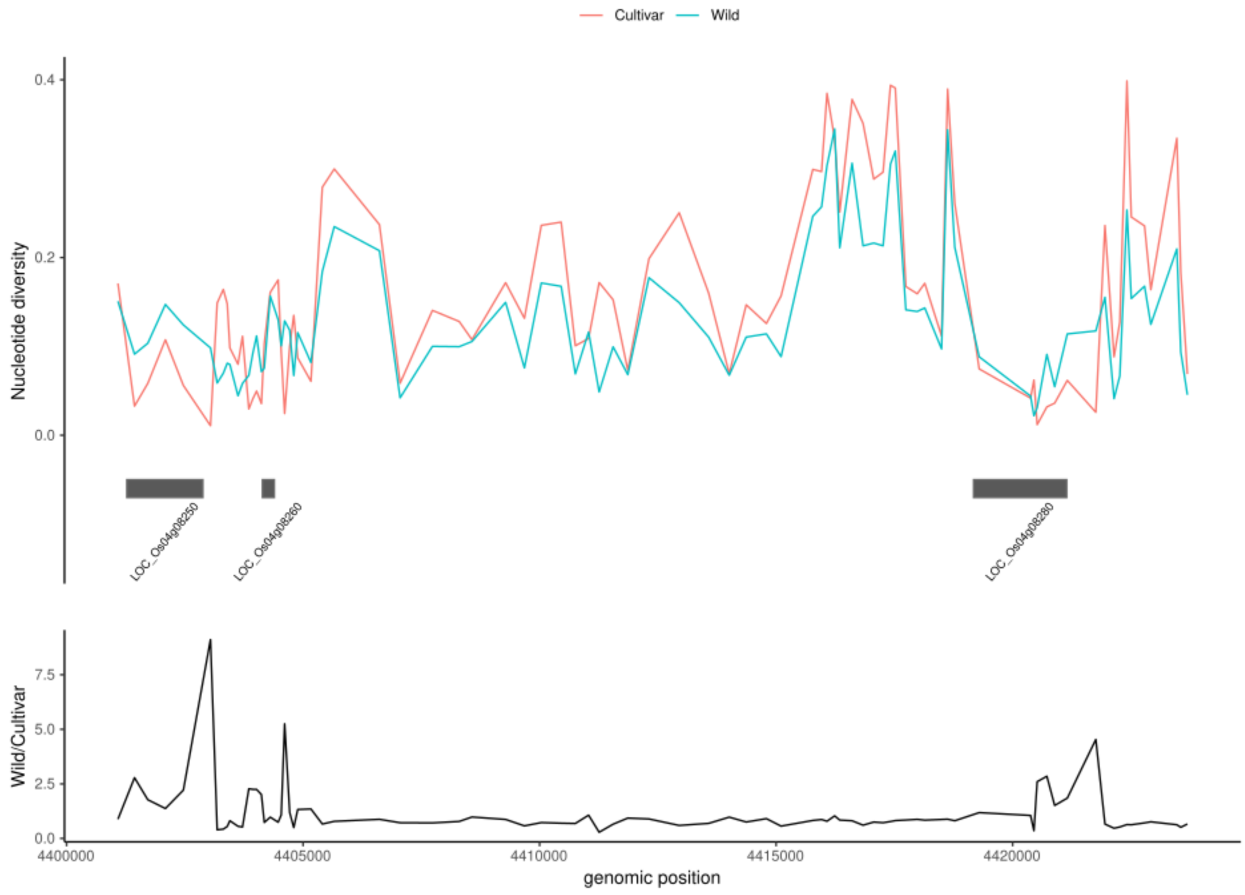

Os04g0164900

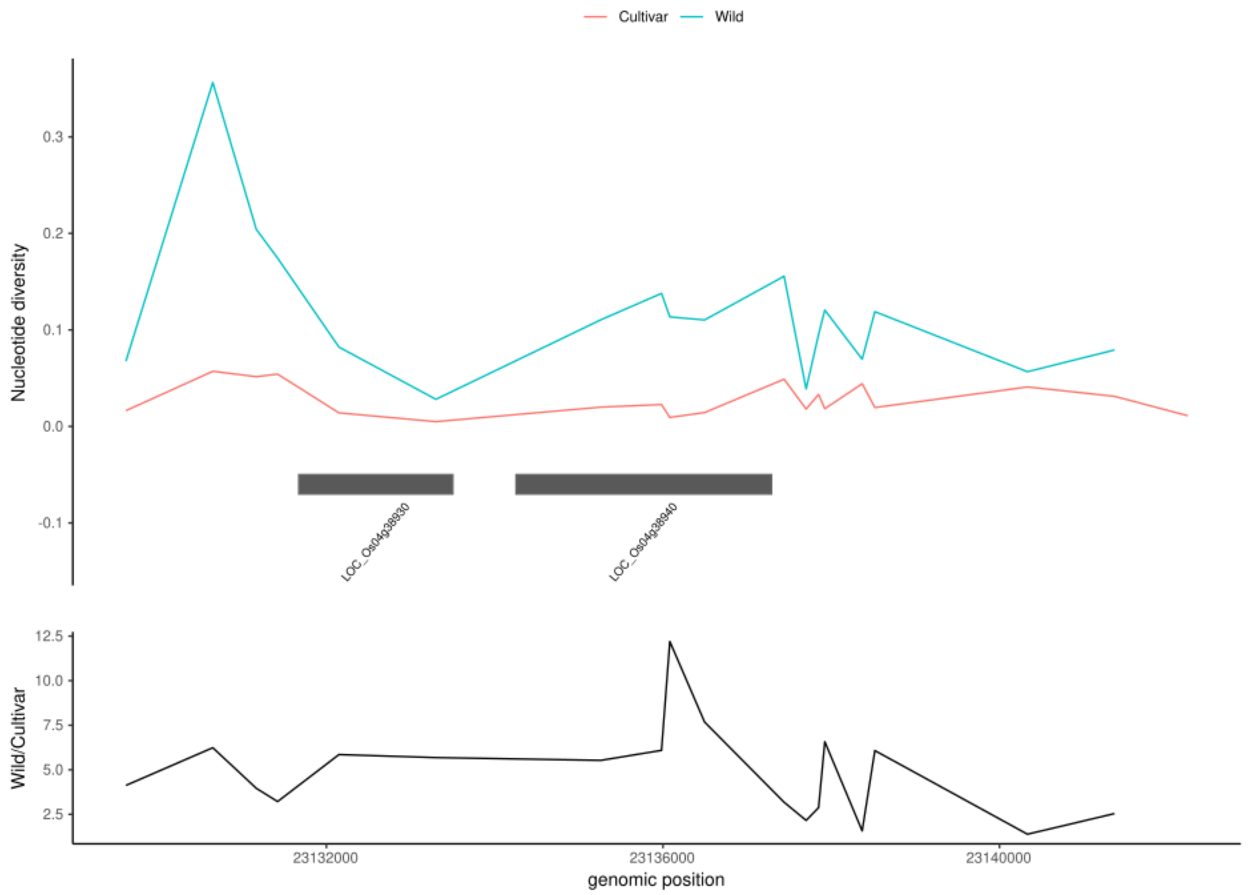

Os04g0463400

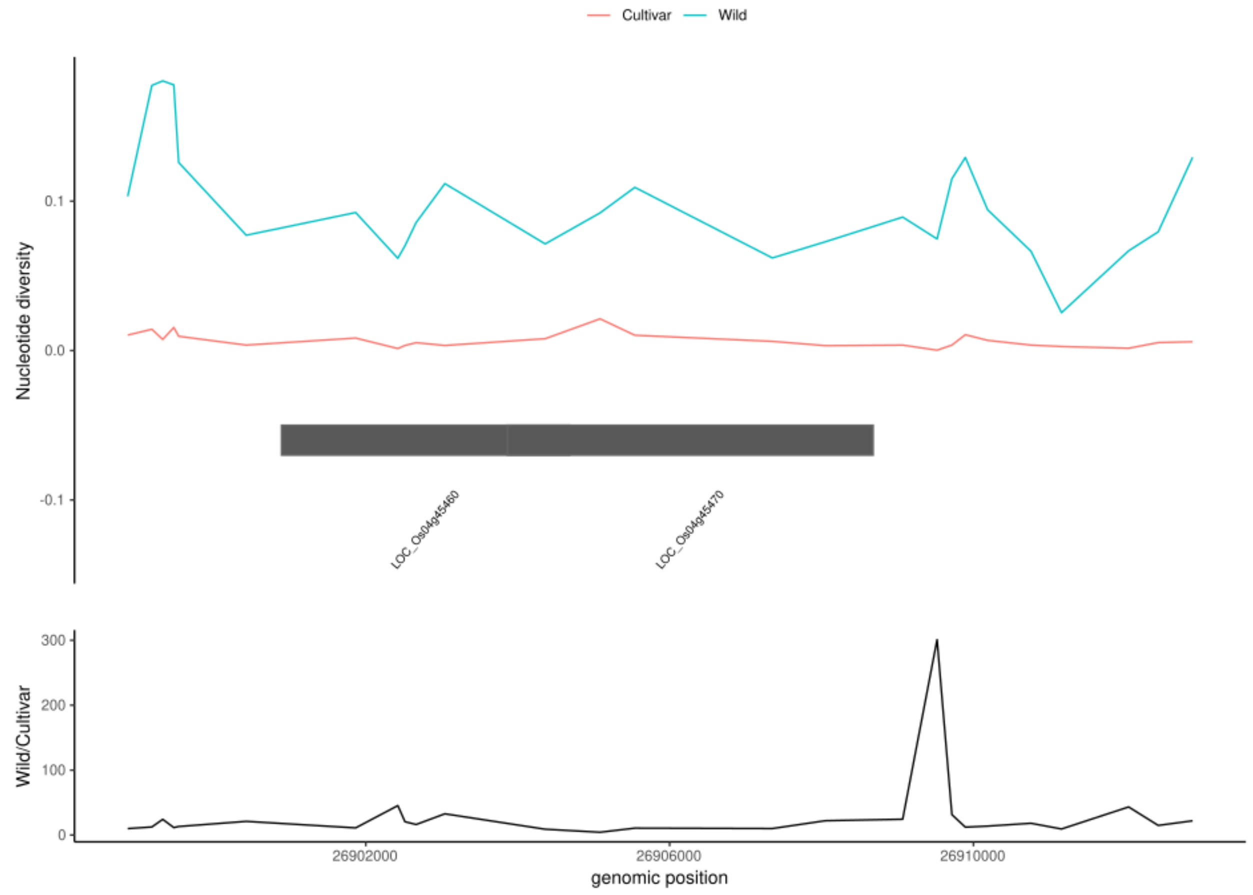

Os04g0537900

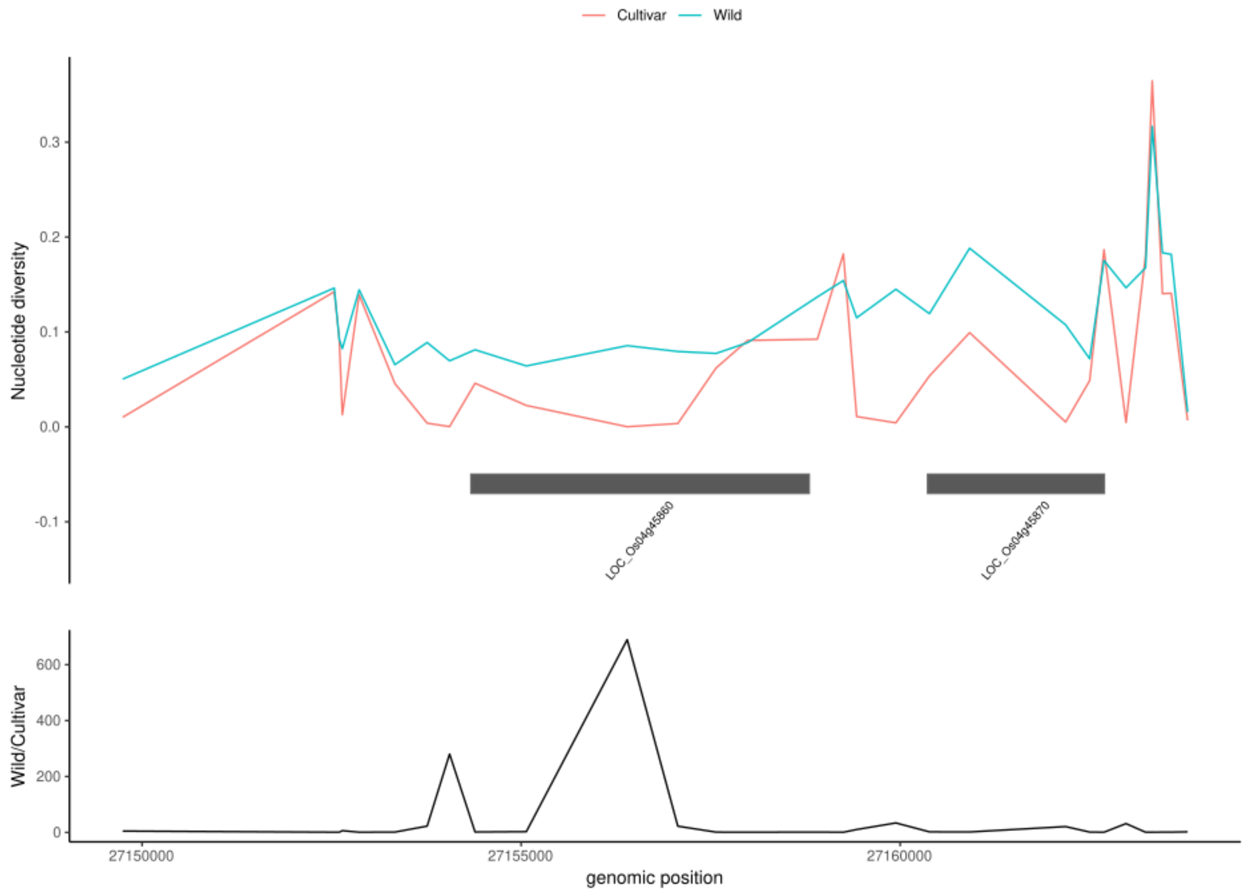

Os04g0542200

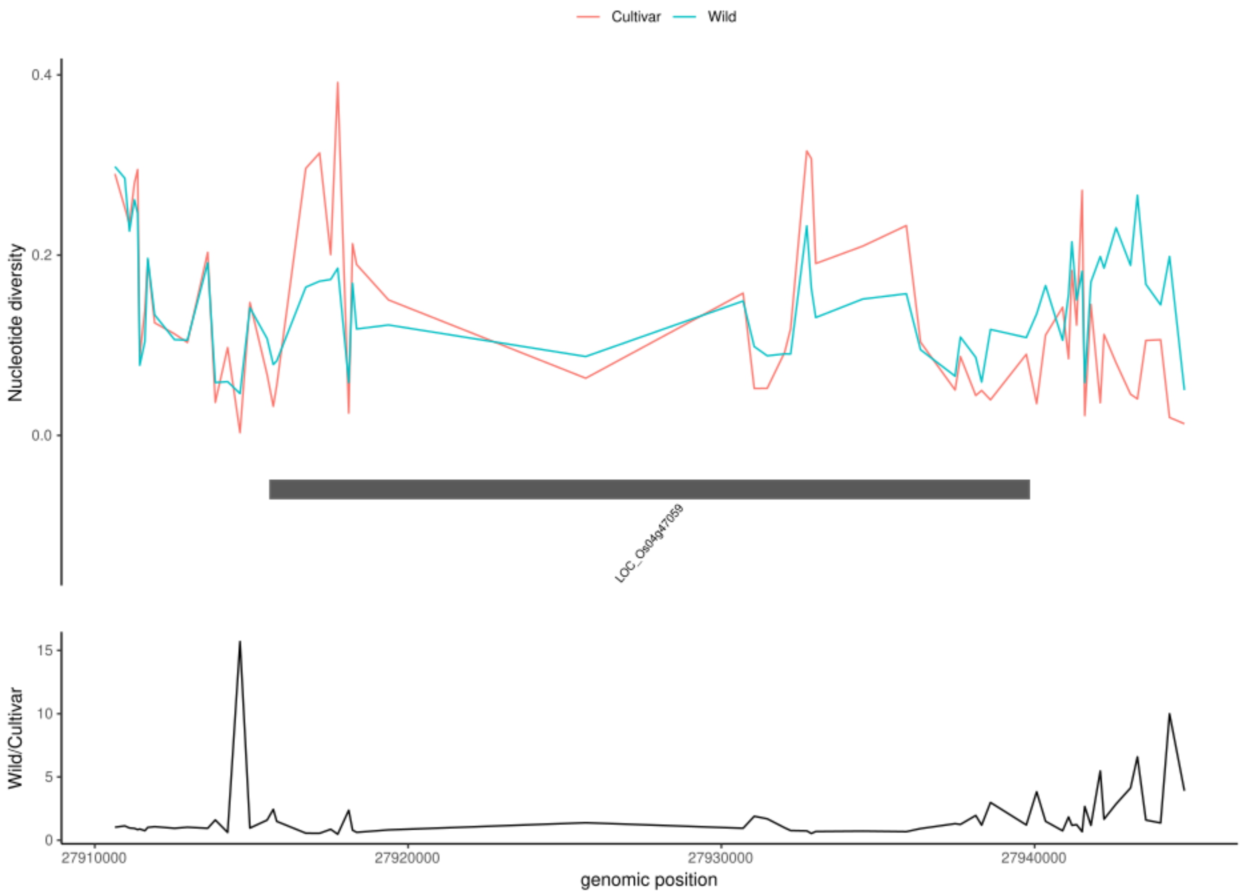

Os04g0557500

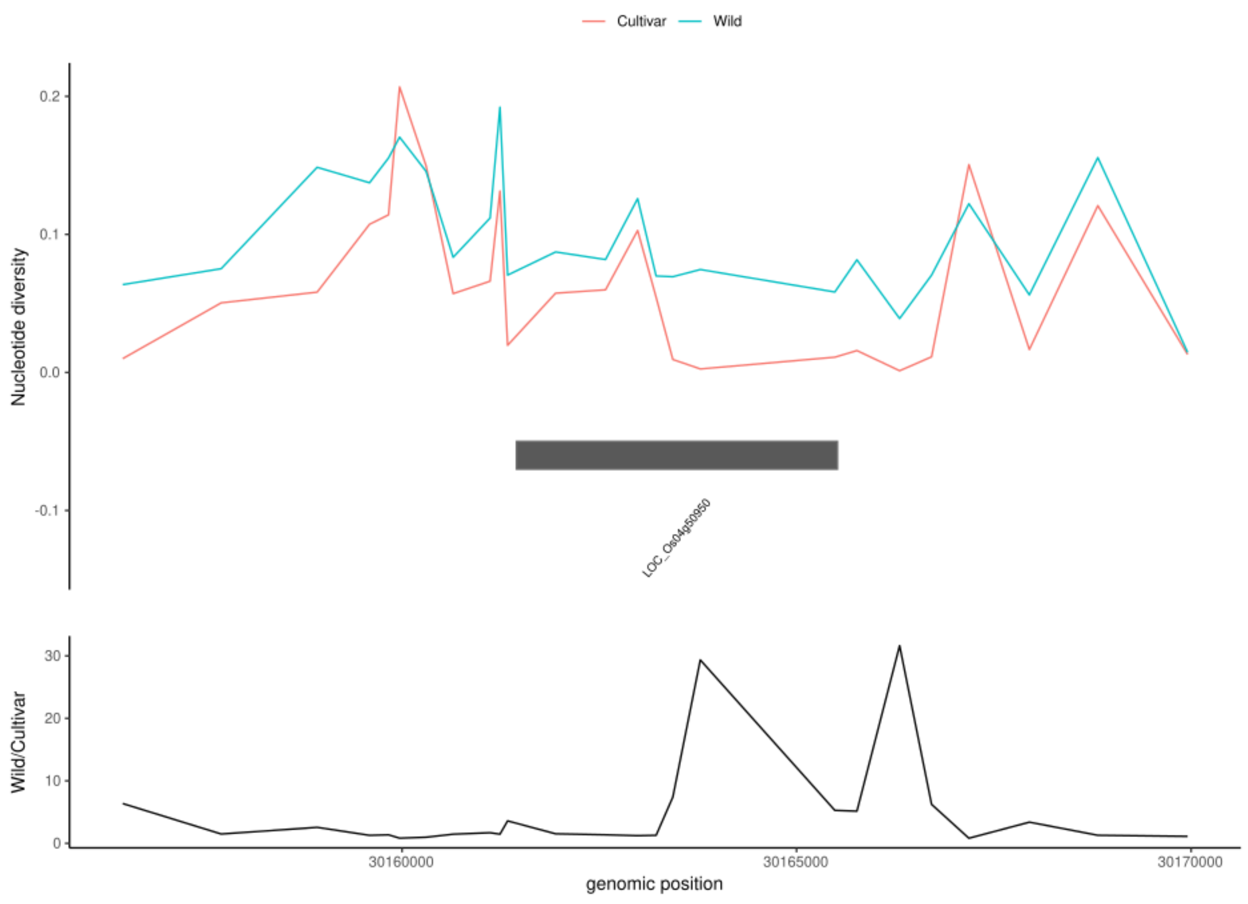

Os04g0597800

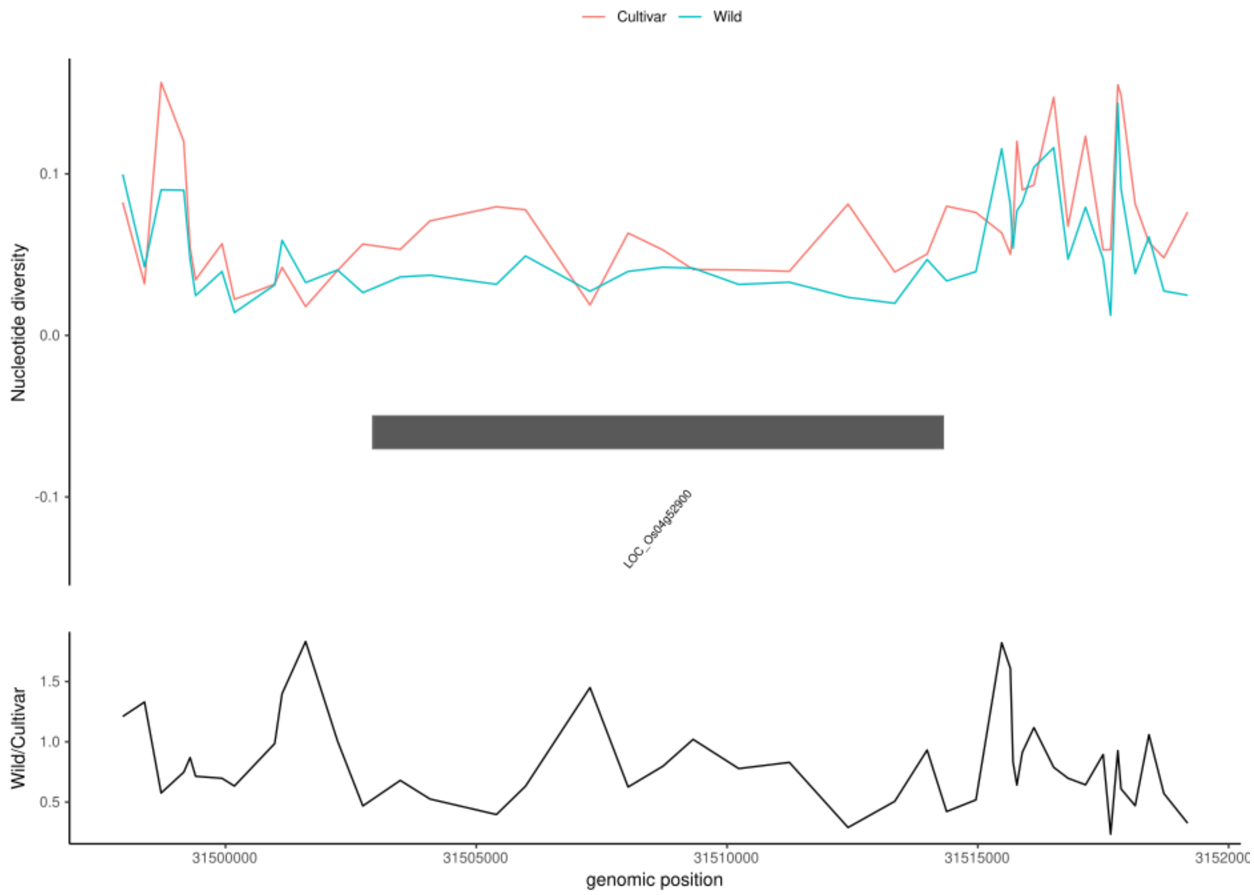

Os04g0620000

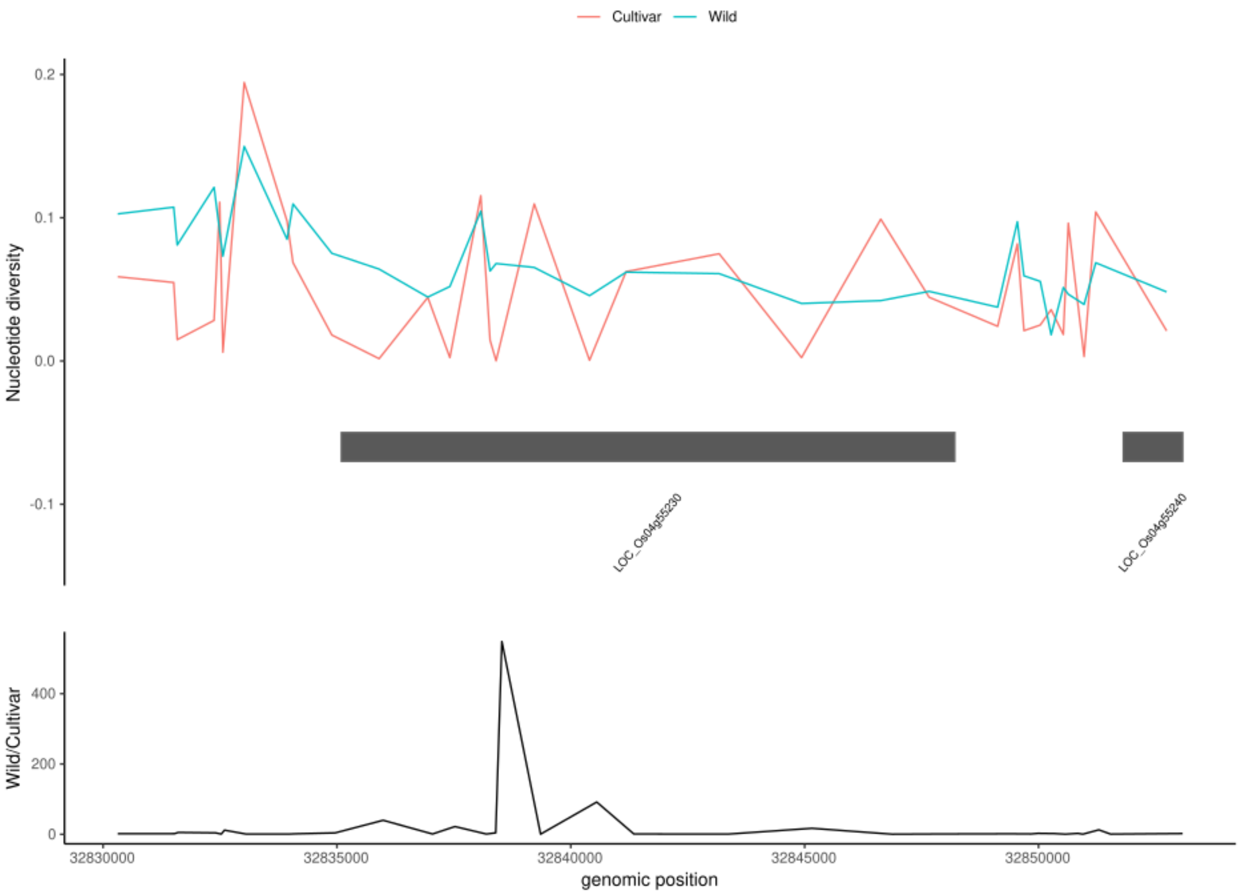

Os04g0645100

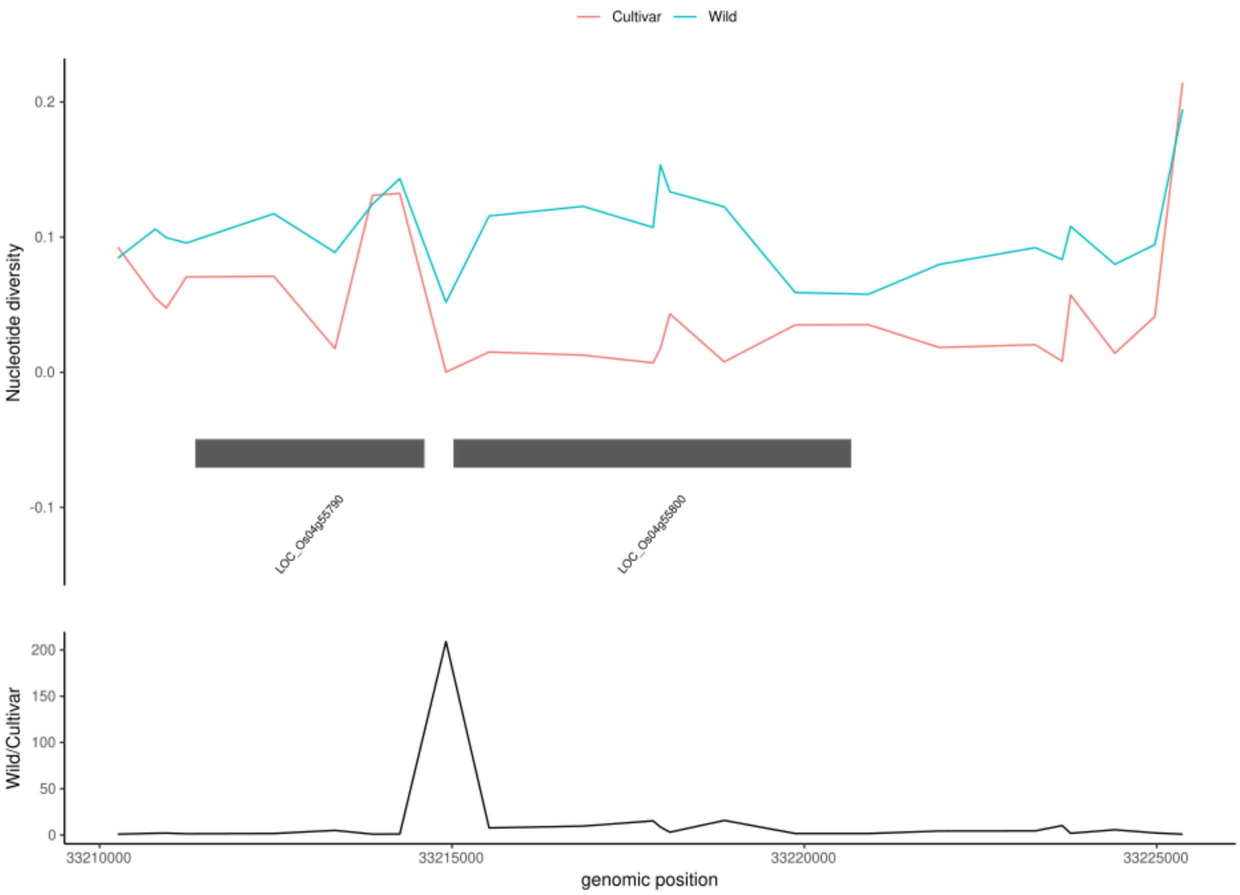

Os04g0652400

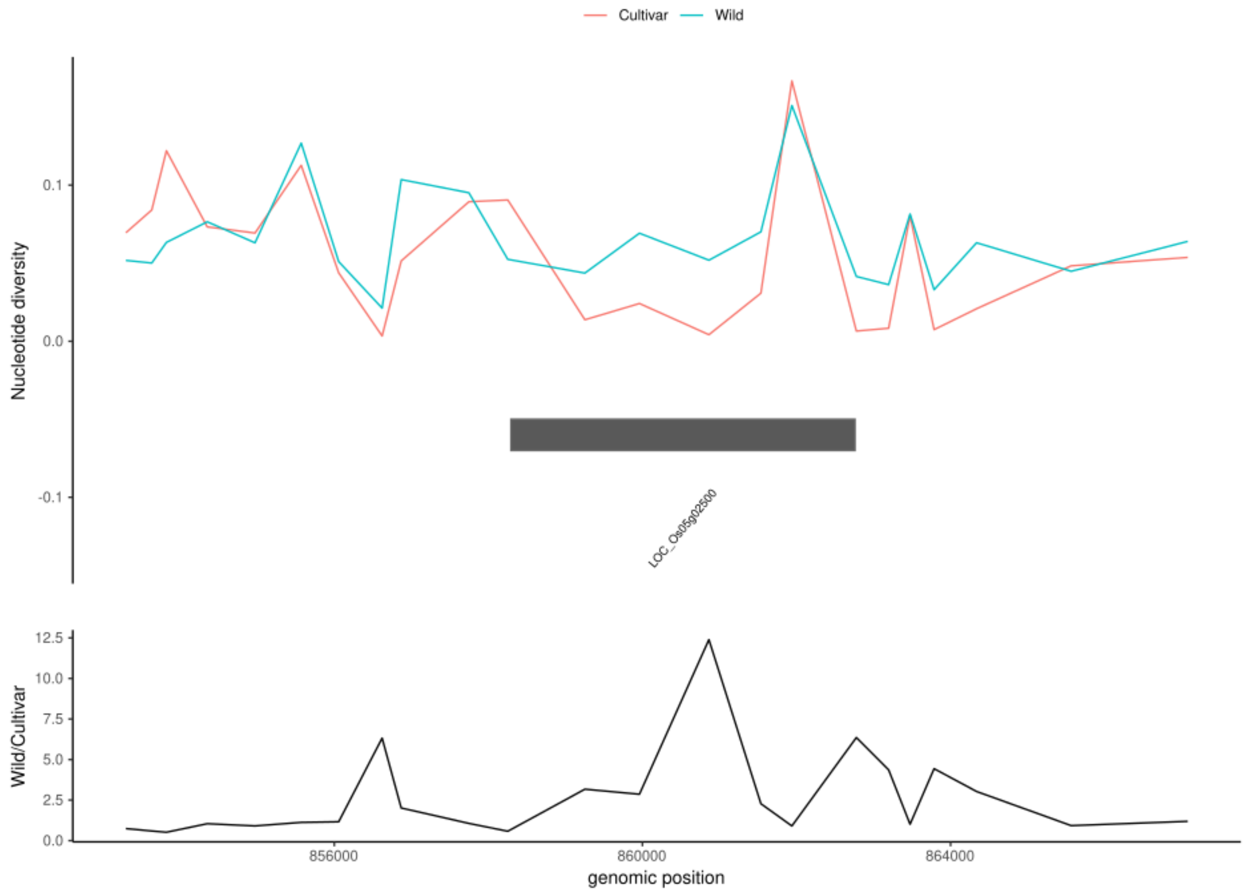

Os05g0115800

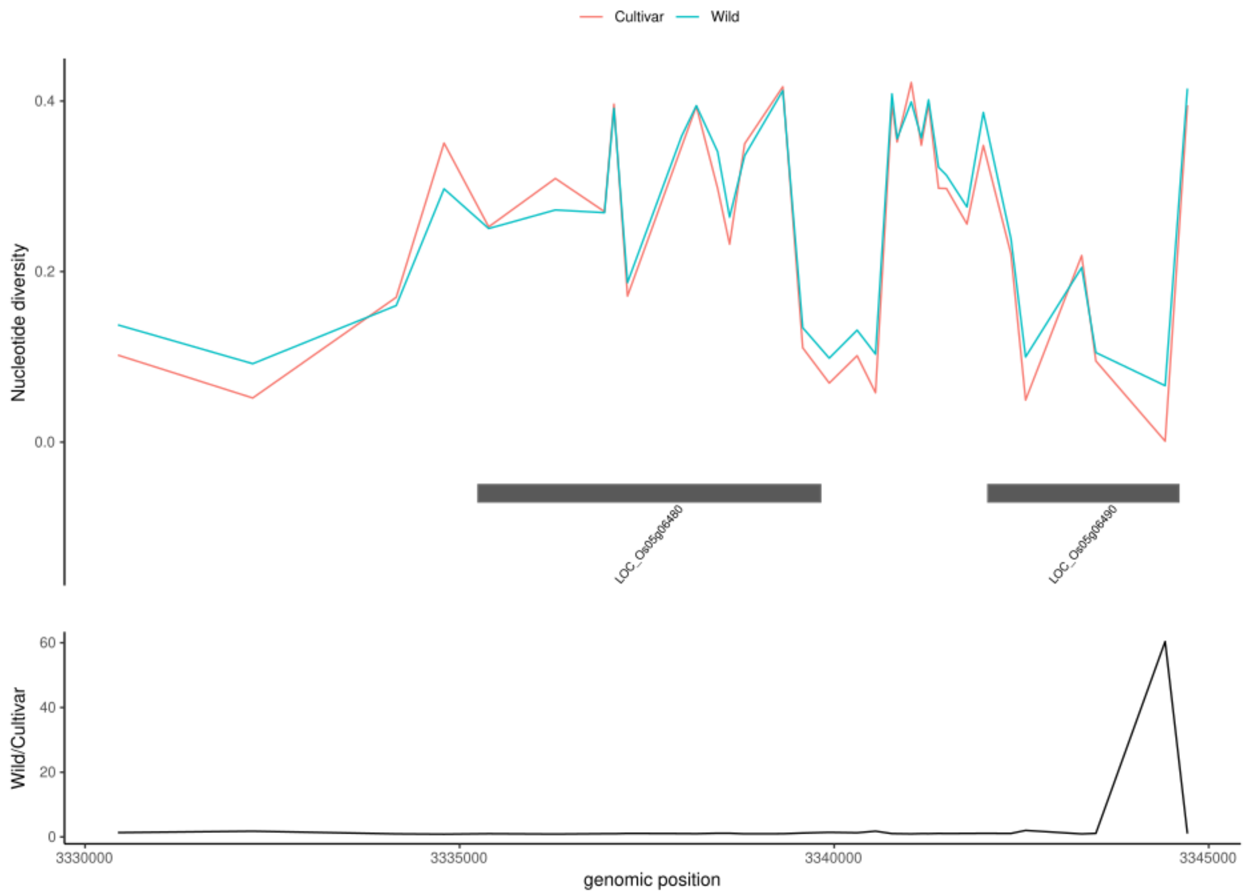

Os05g0156900

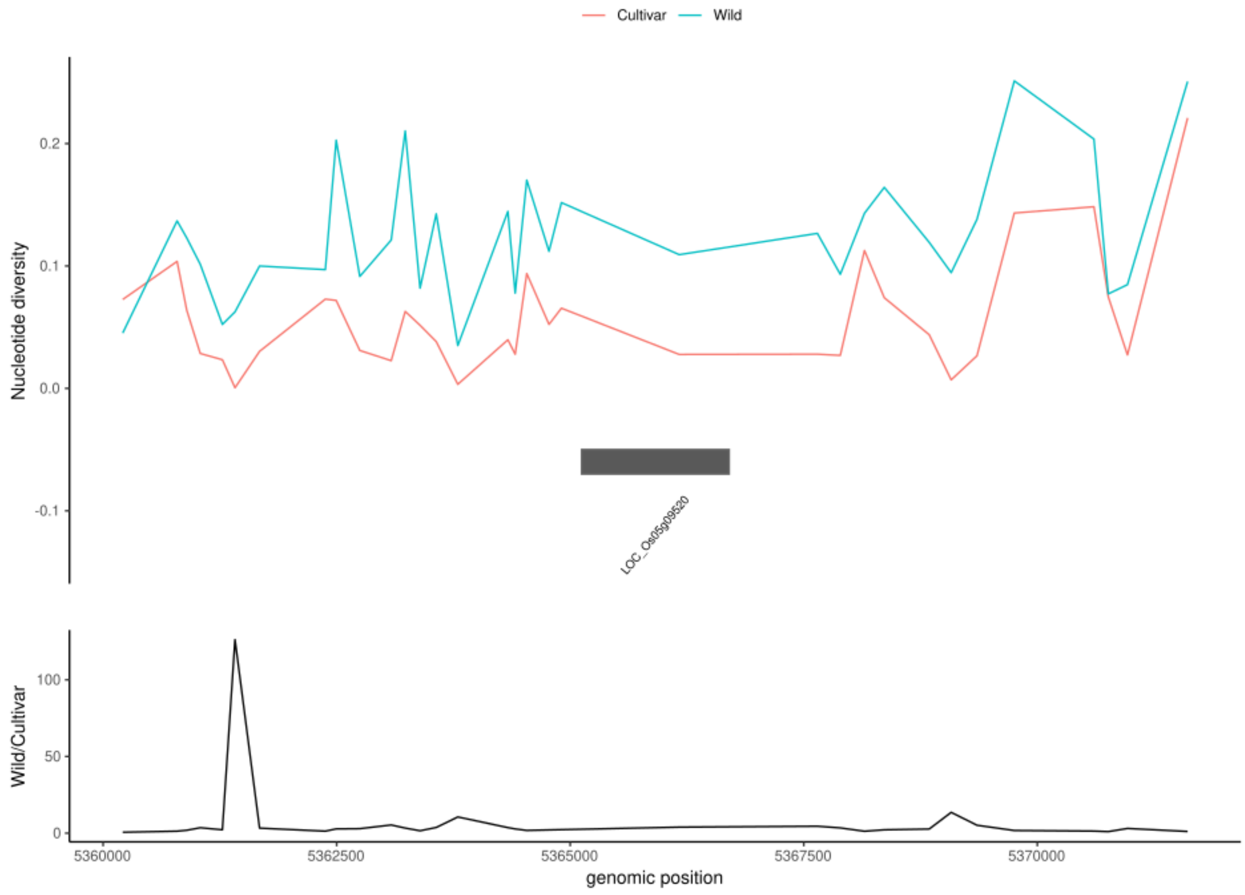

Os05g0187500

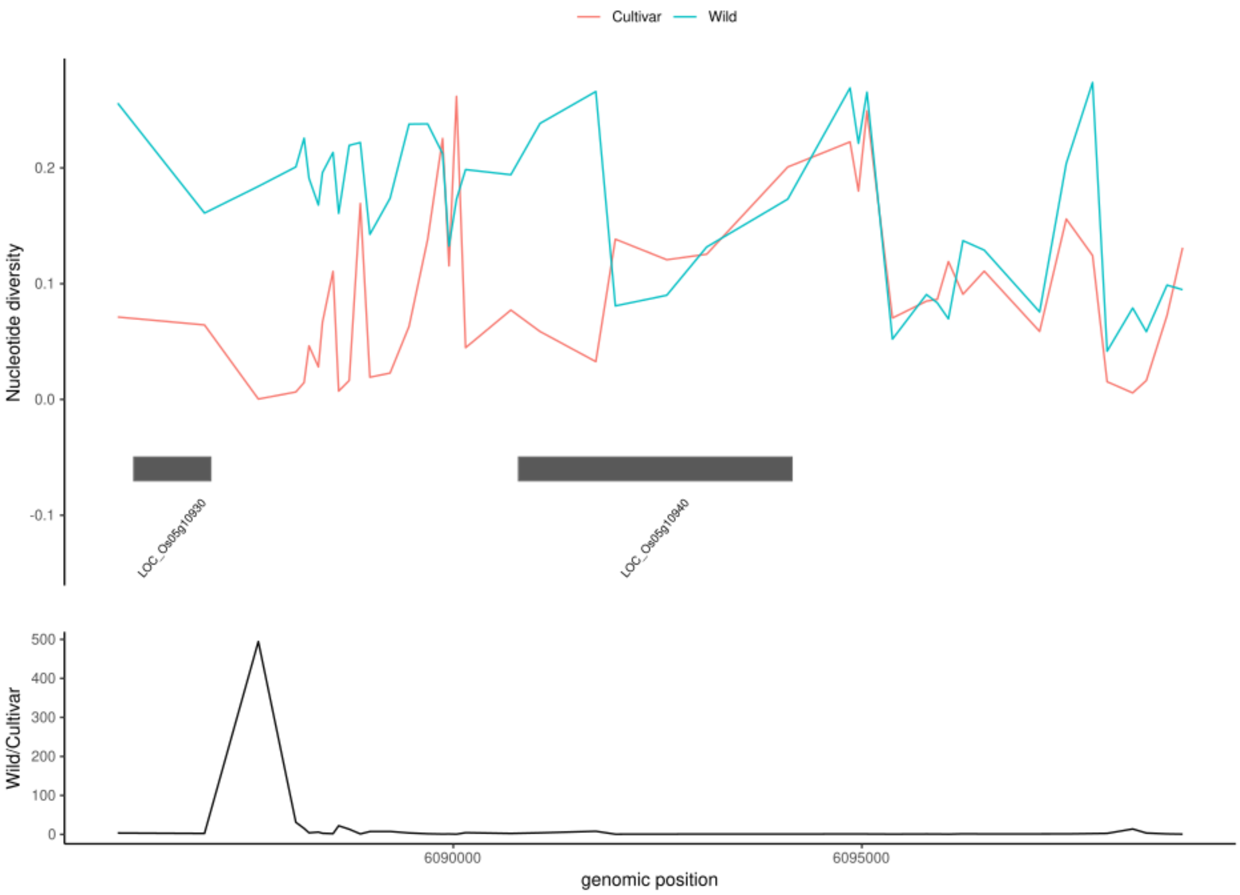

Os05g0198400

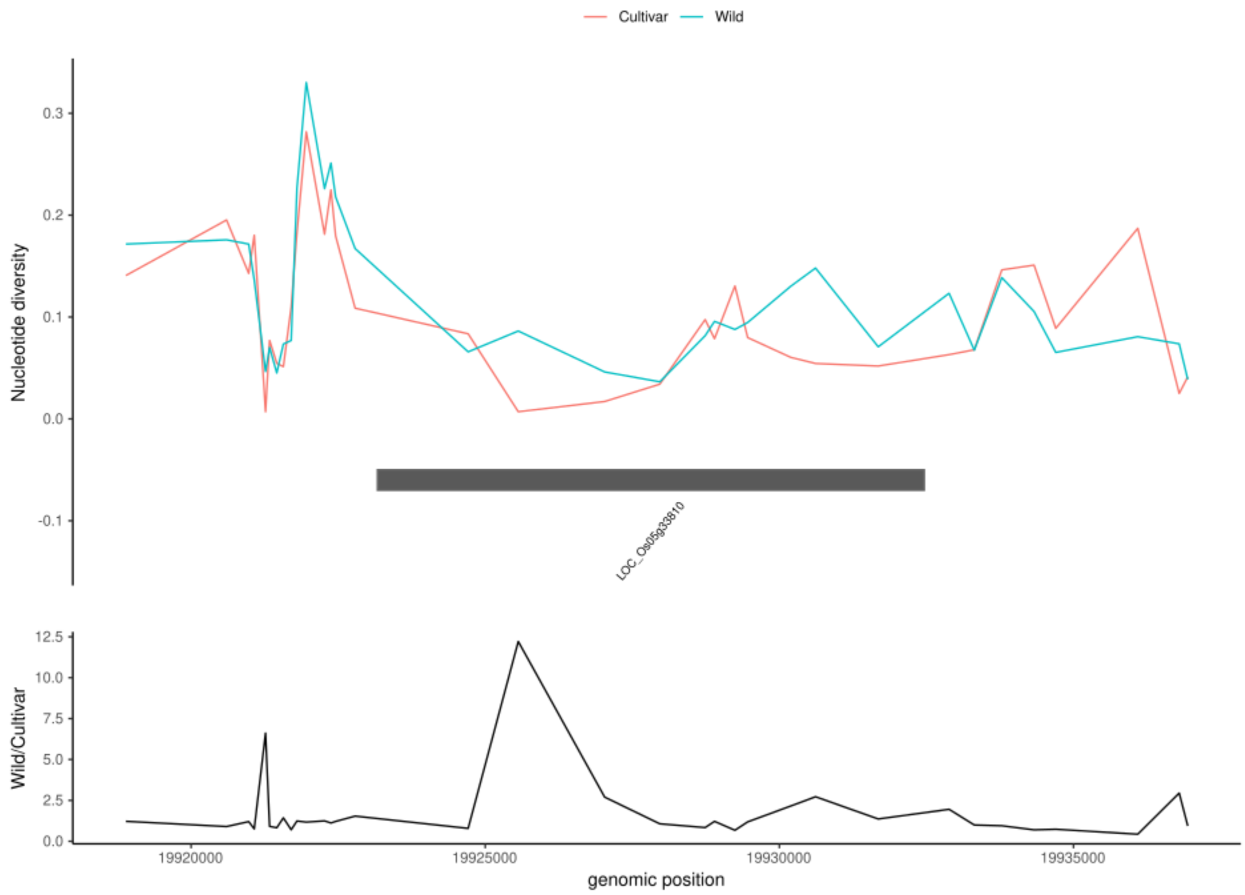

Os05g0408200

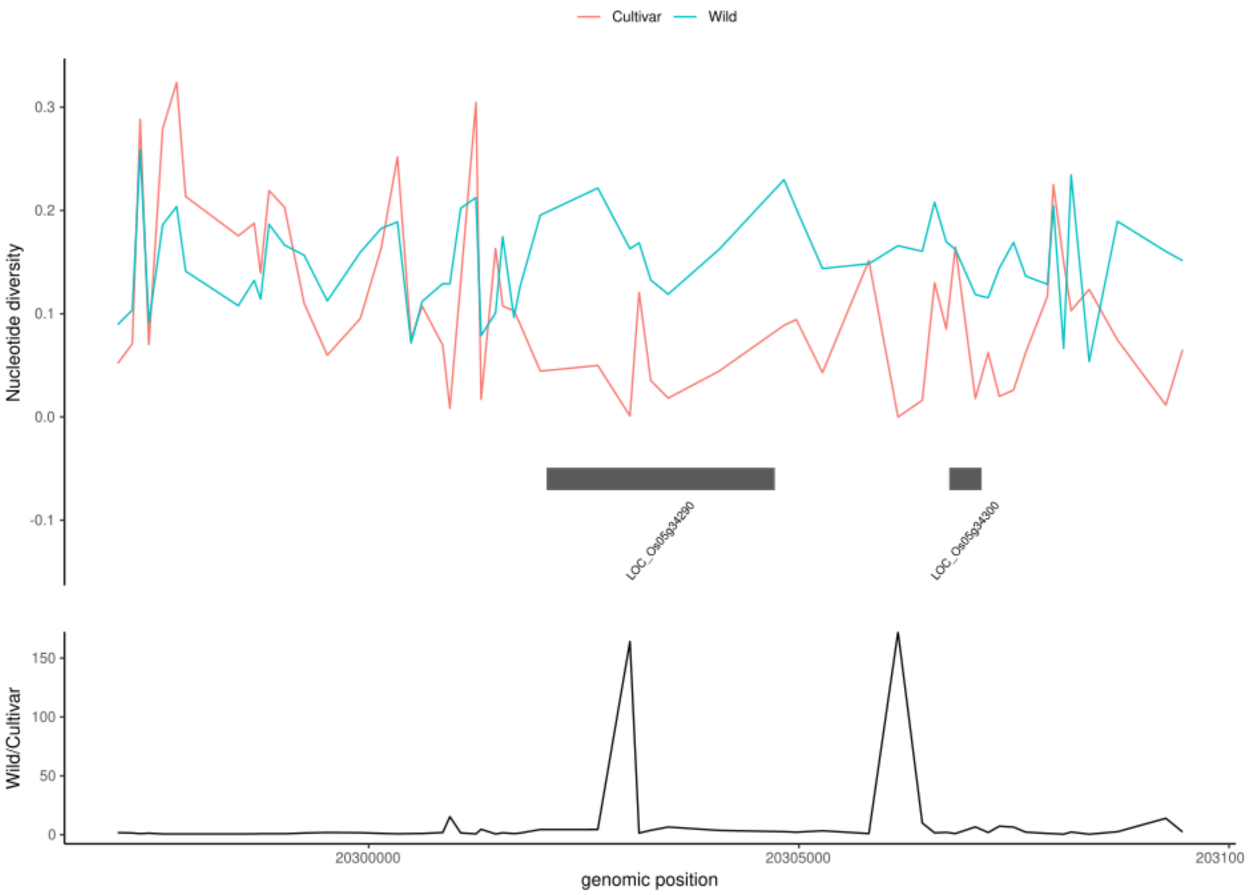

Os05g0415200

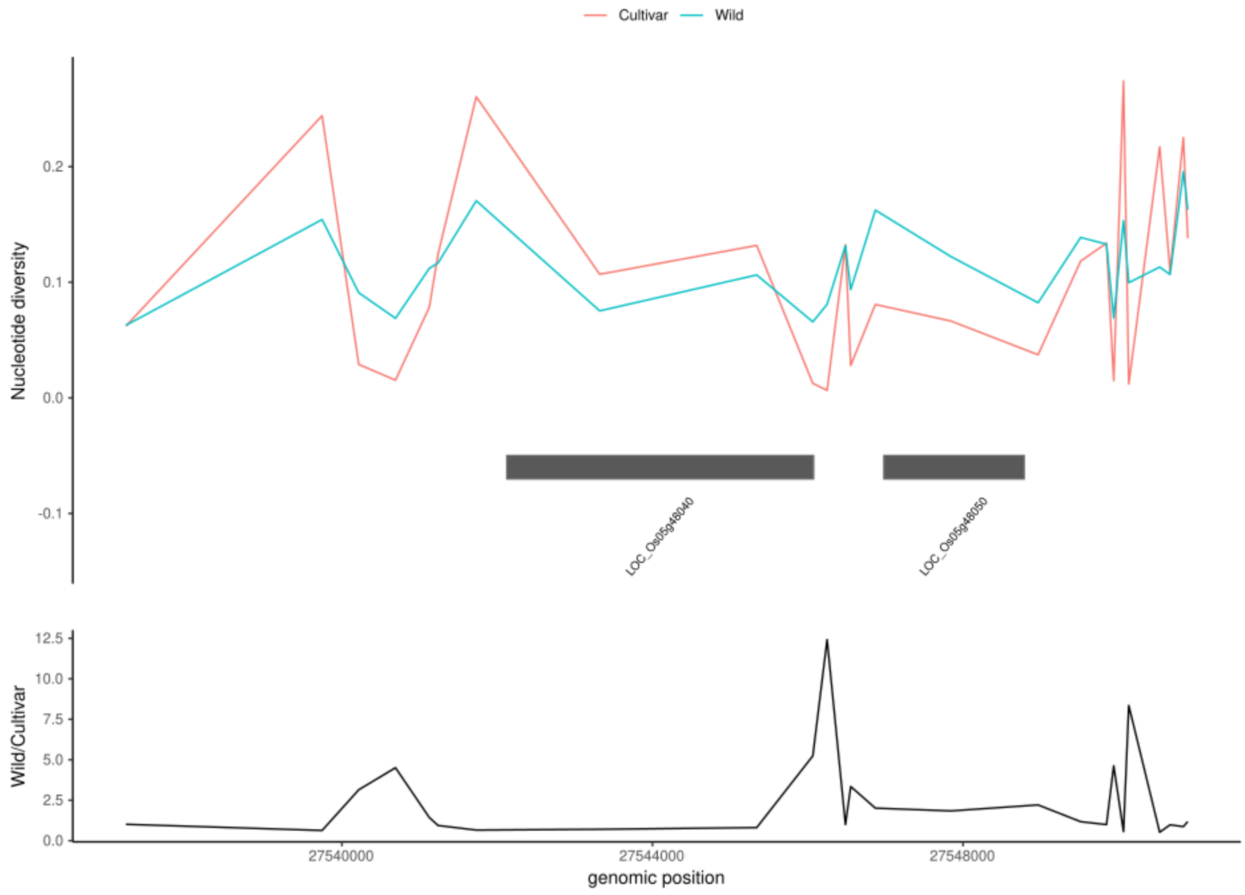

Os05g0554000

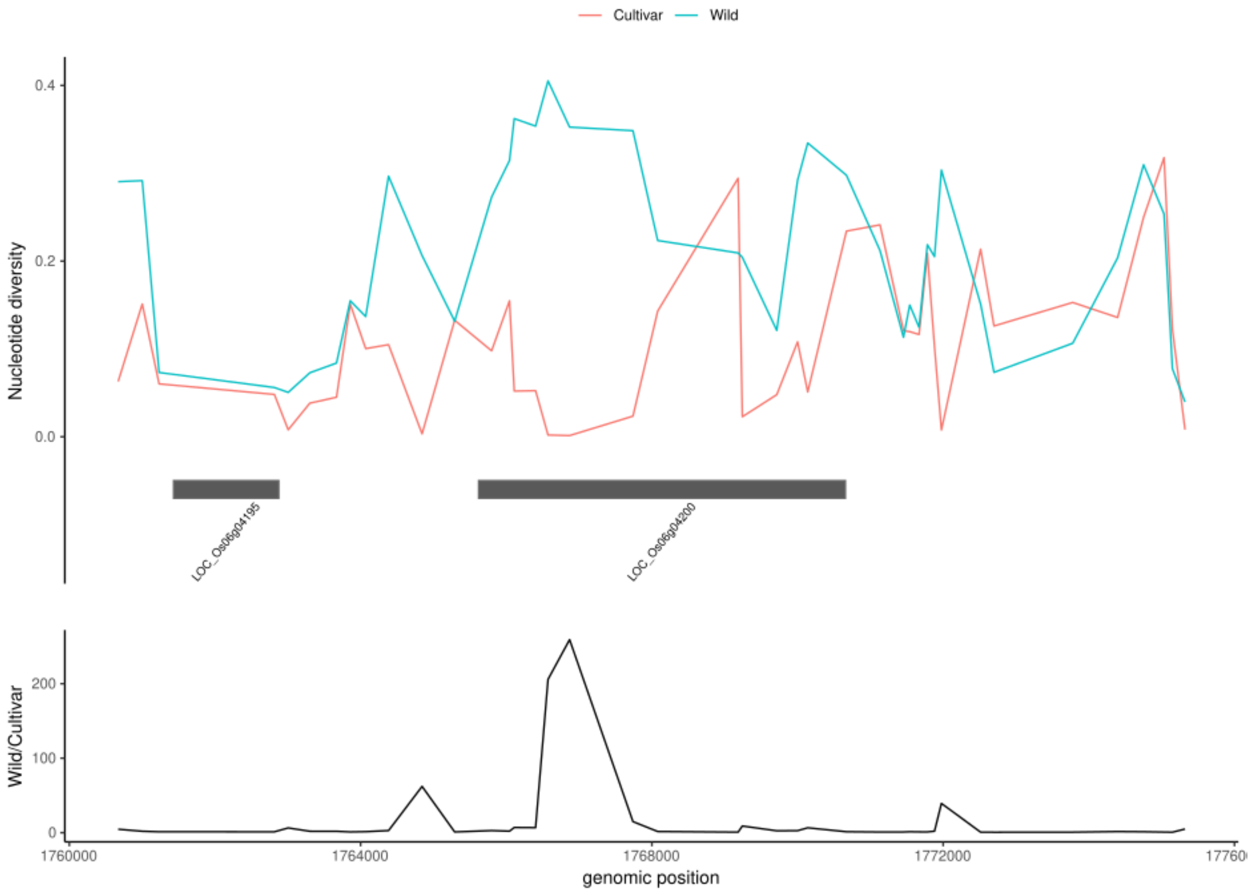

Os06g0133000

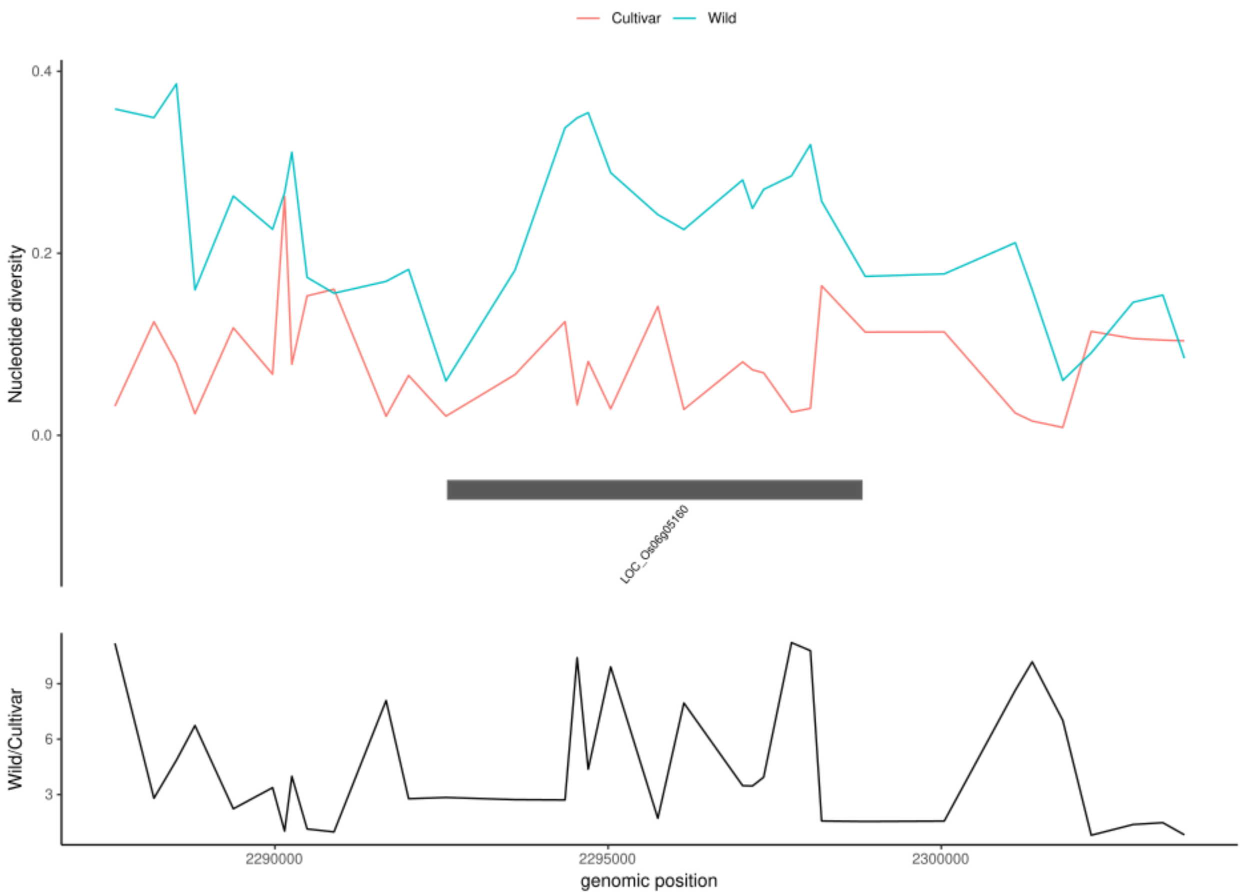

Os06g0143700

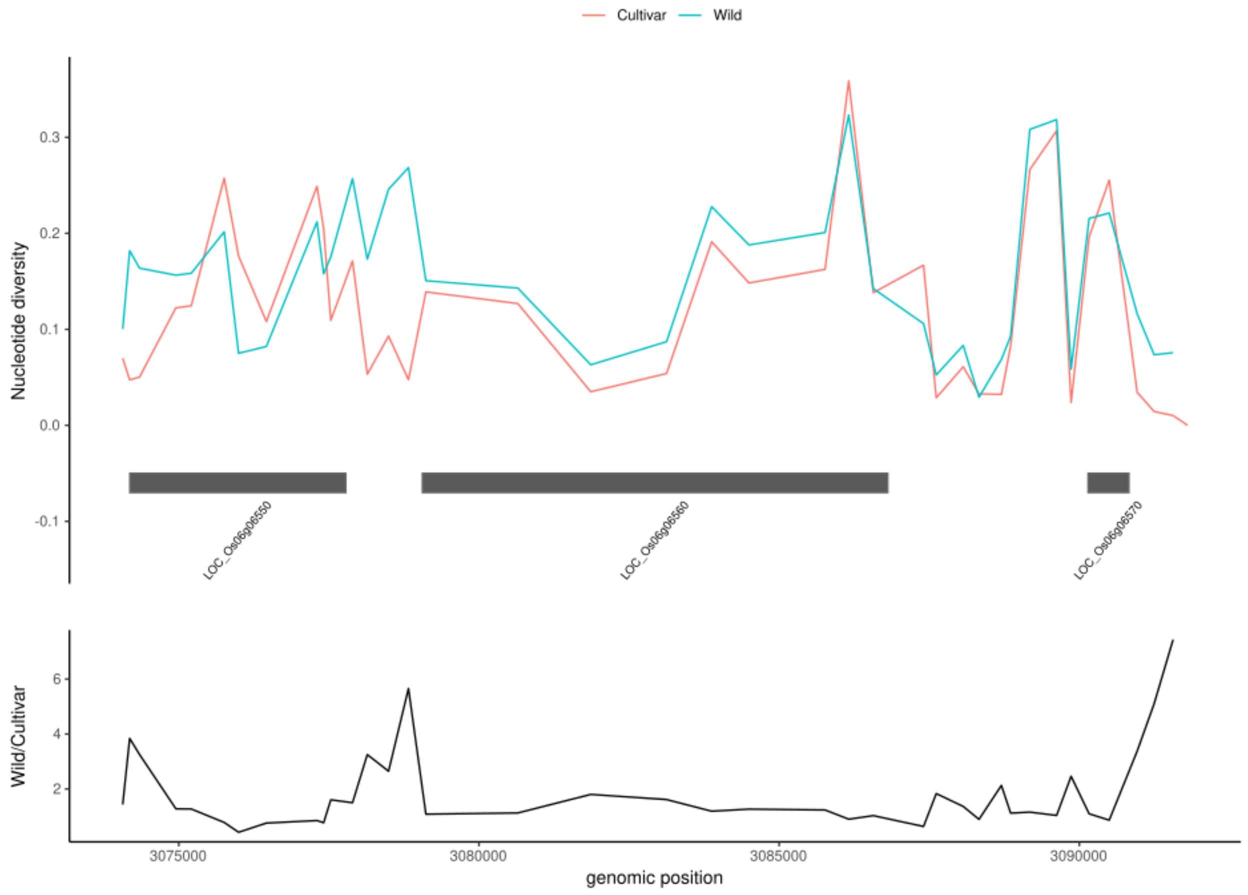

Os06g0160700

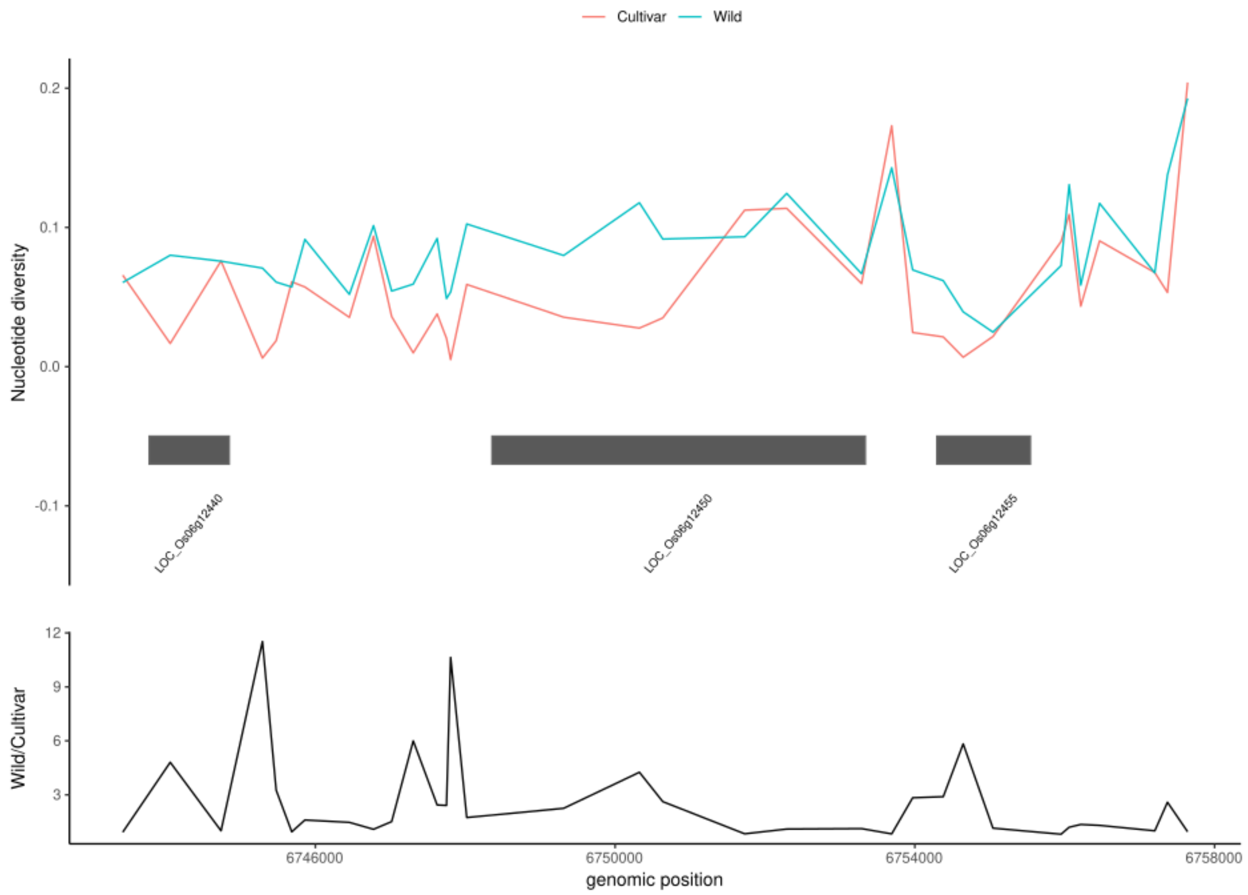

Os06g0229800

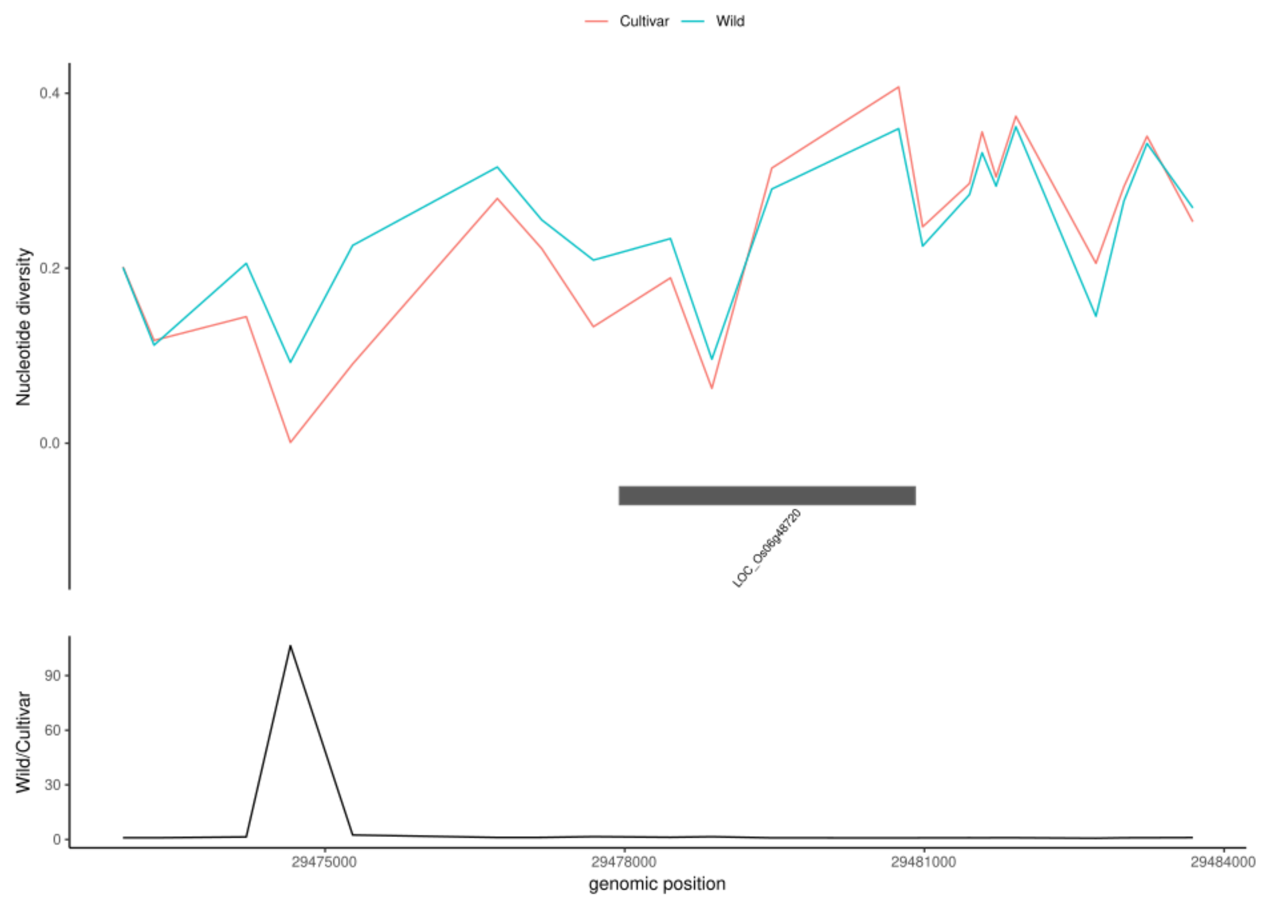

Os06g0700700

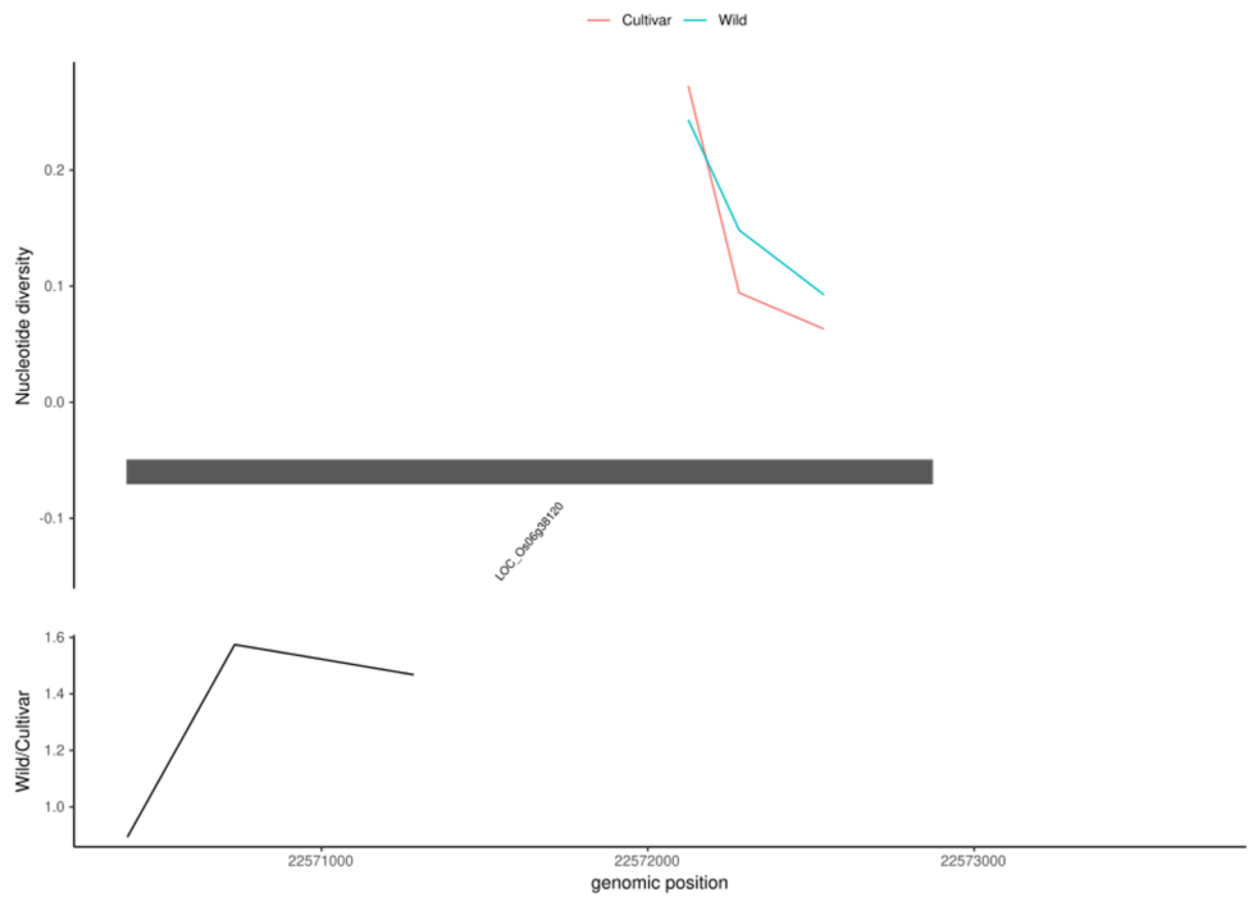

LOC_Os06g38120

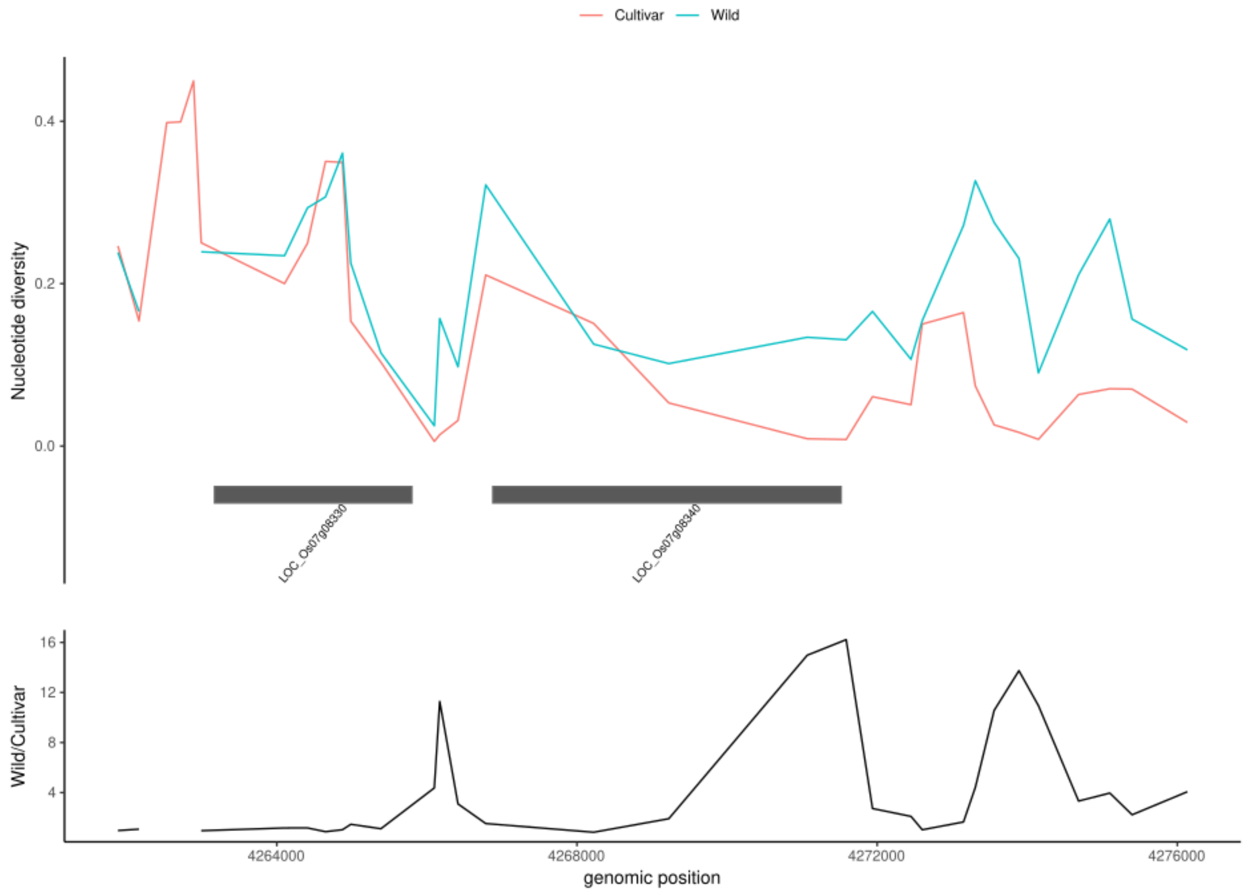

Os07g0181000

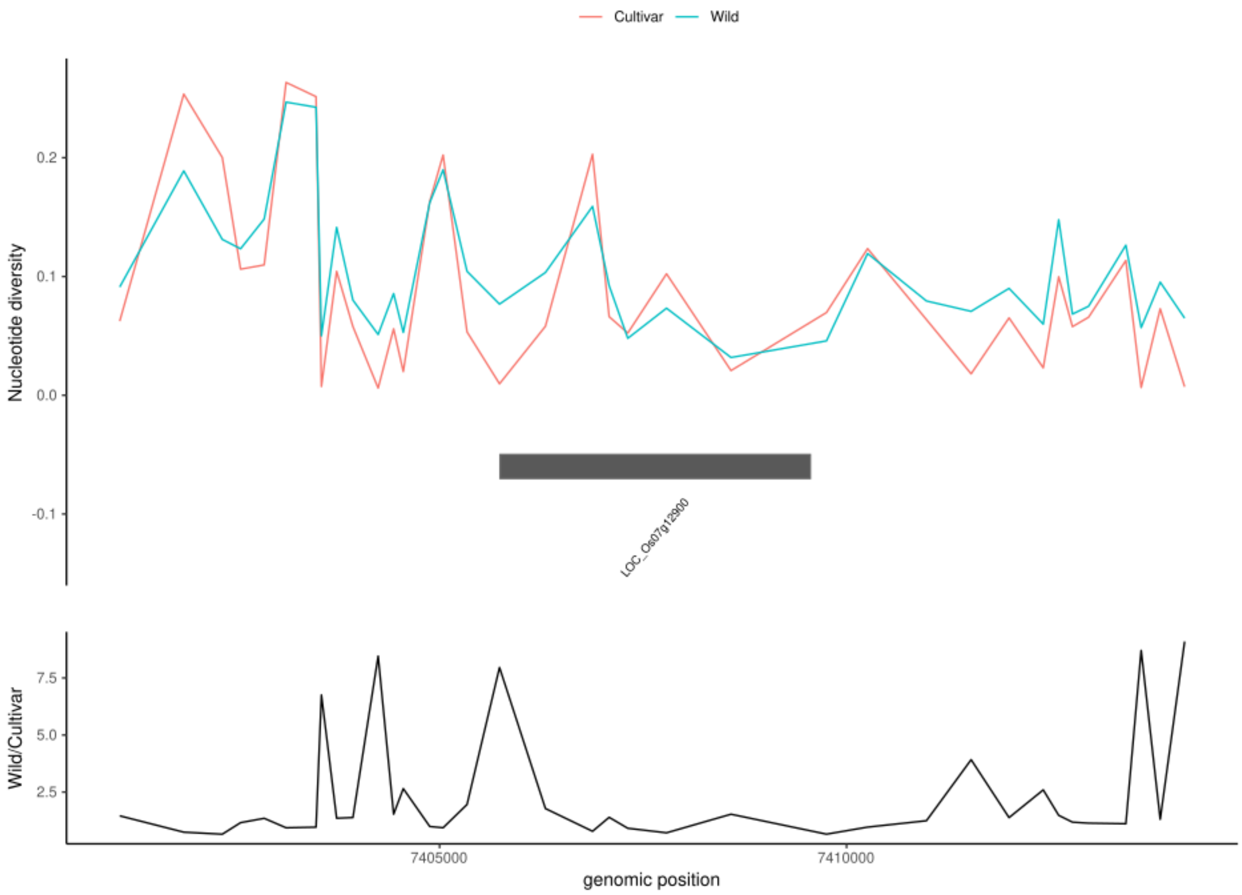

Os07g0232900

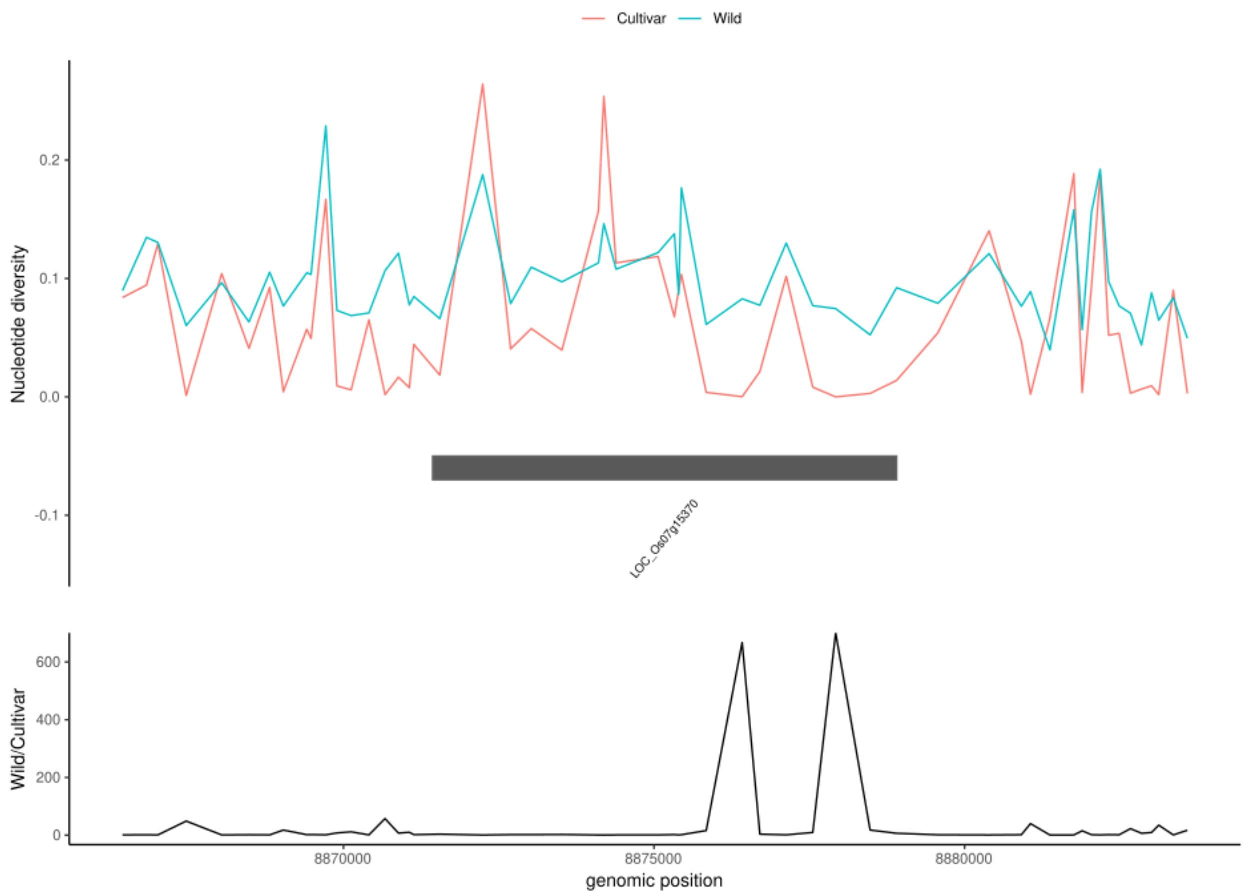

Os07g0257200

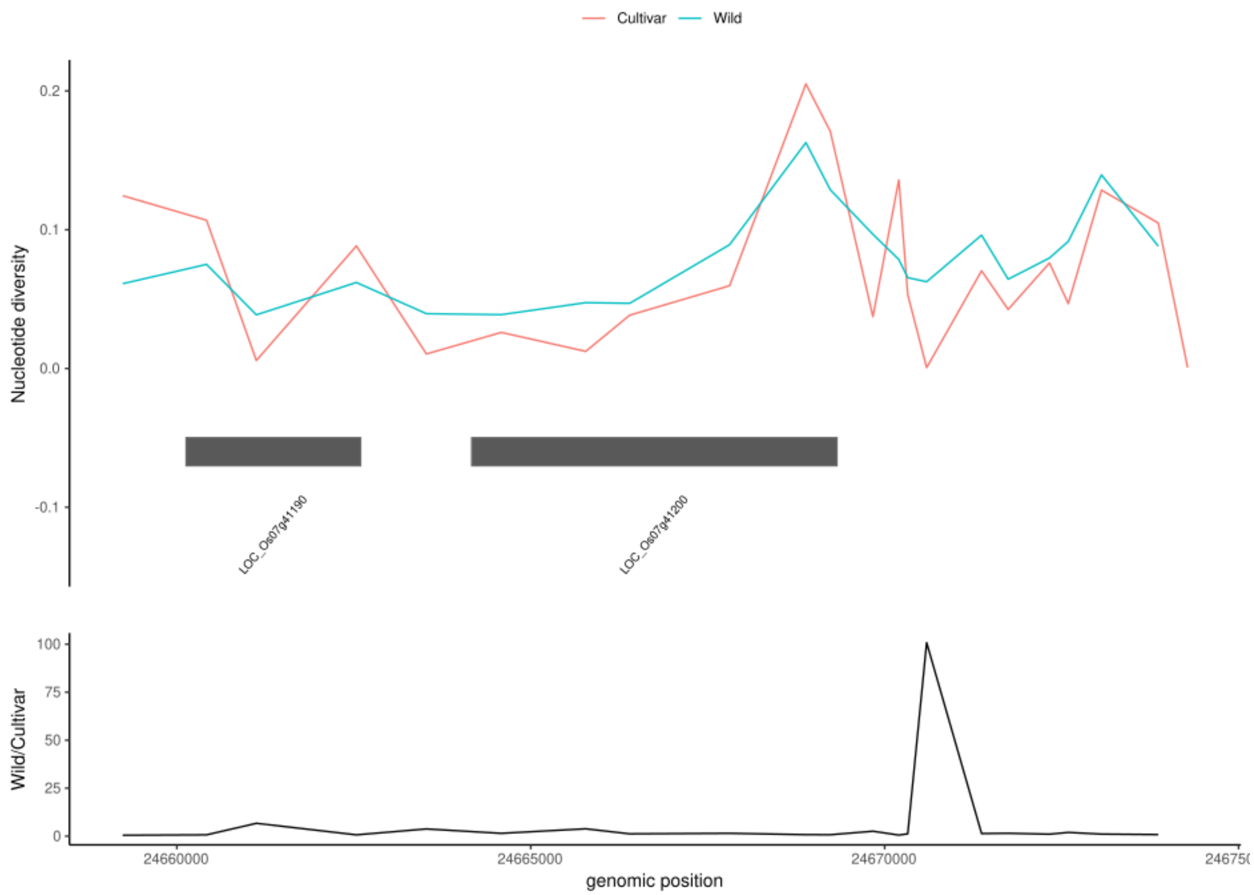

Os07g0603300

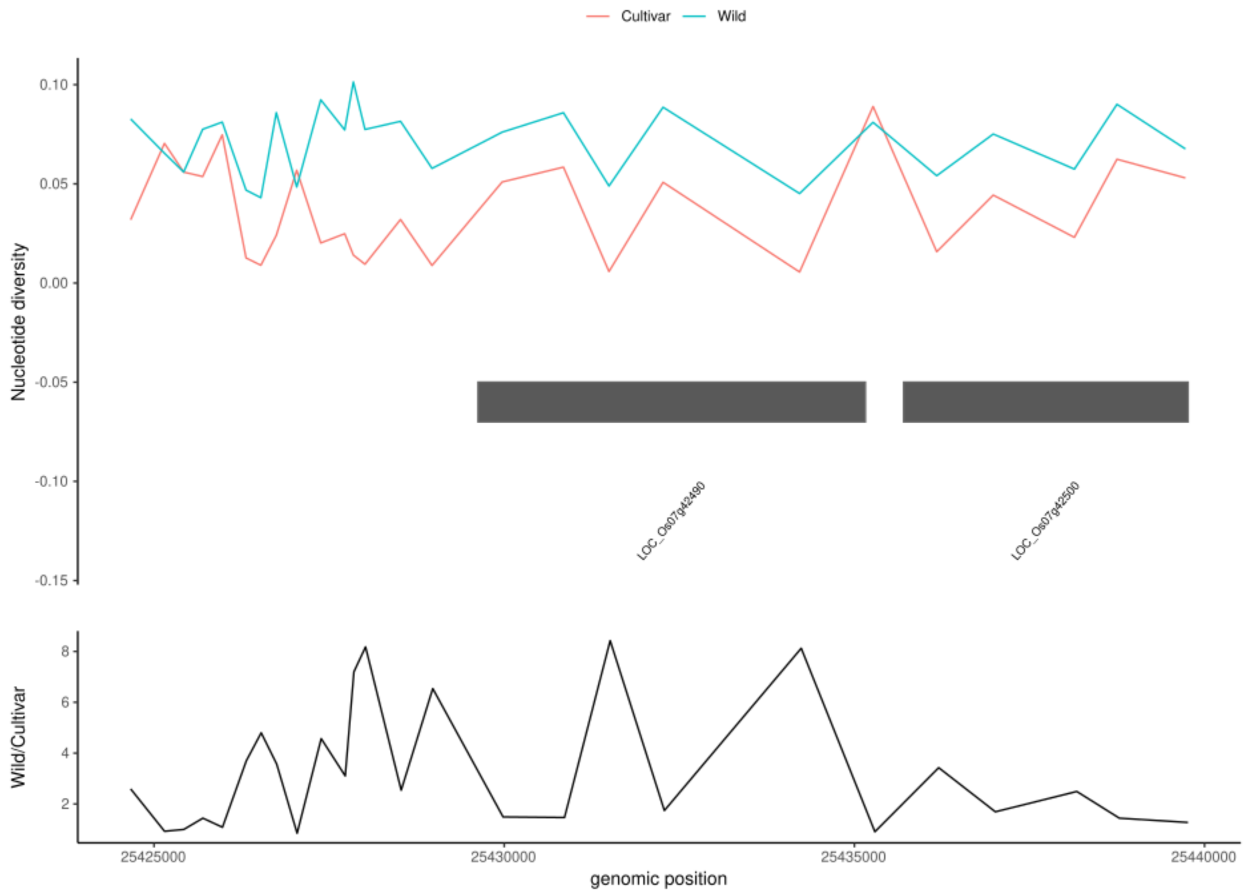

Os07g0616800

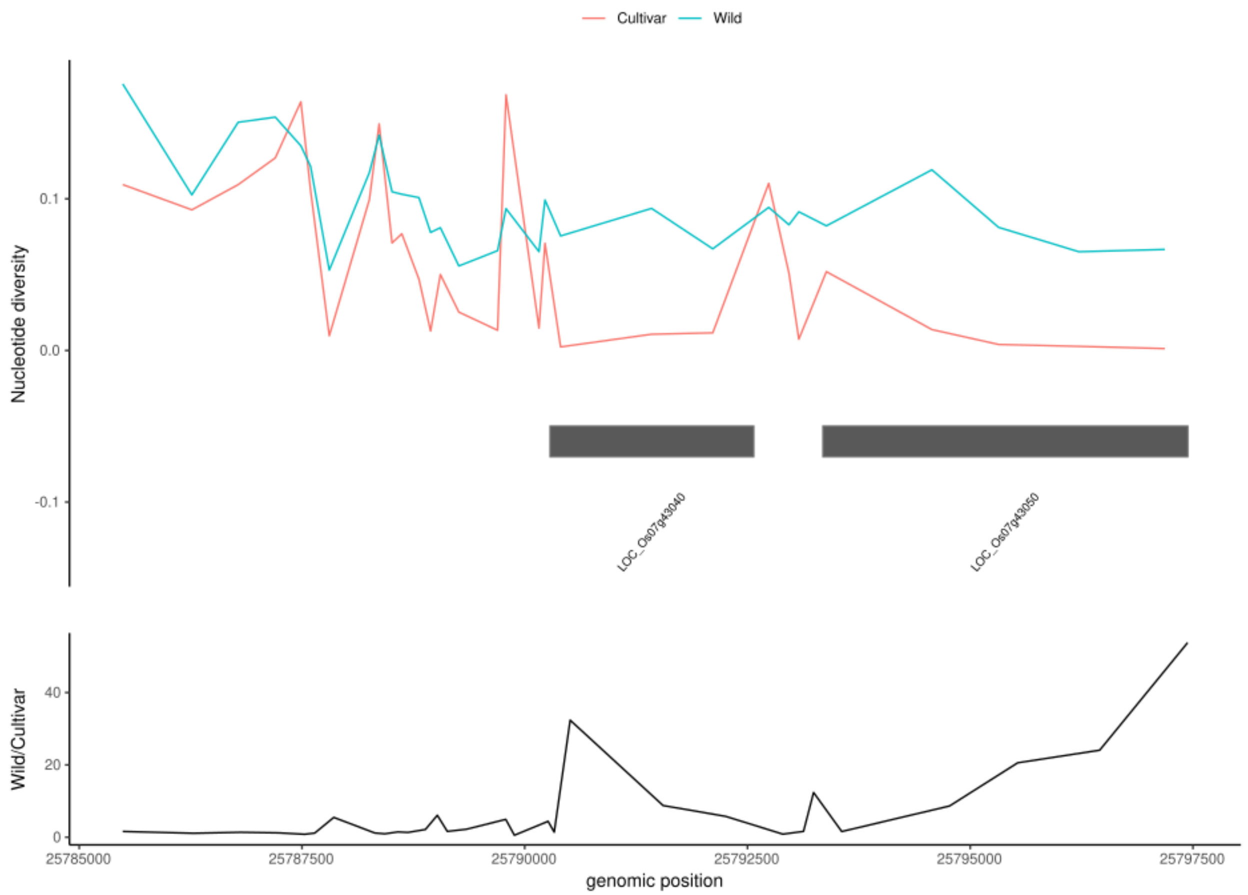

Os07g0623200

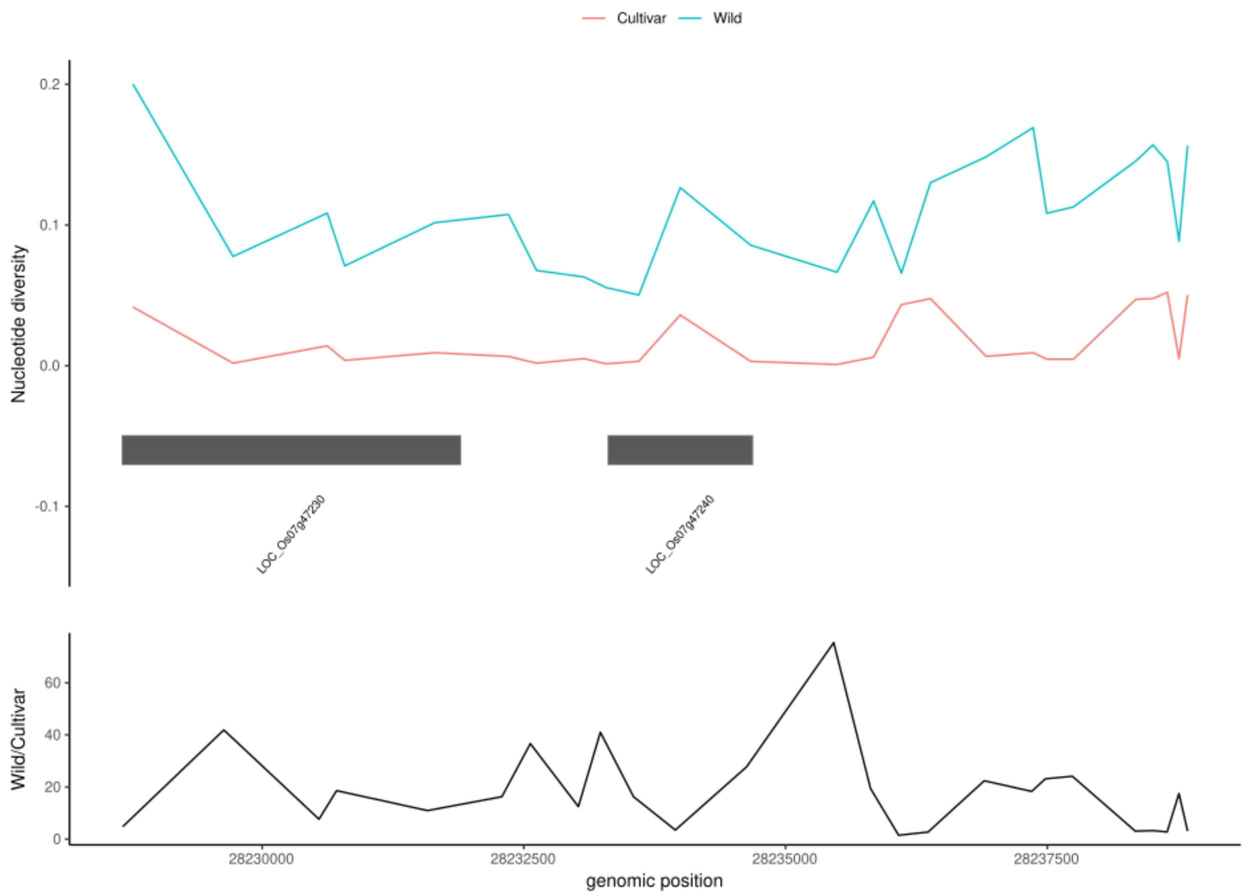

Os07g0668600

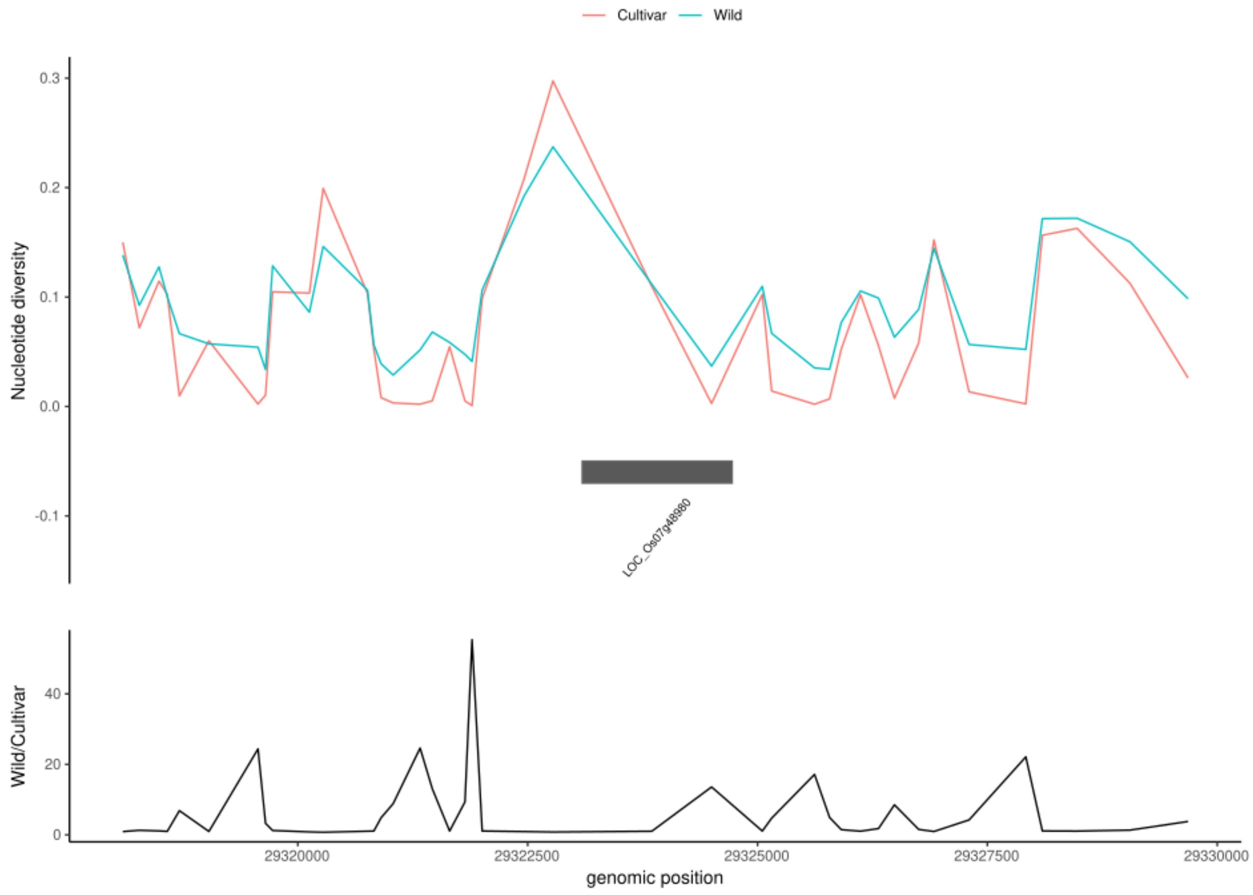

Os07g0689600

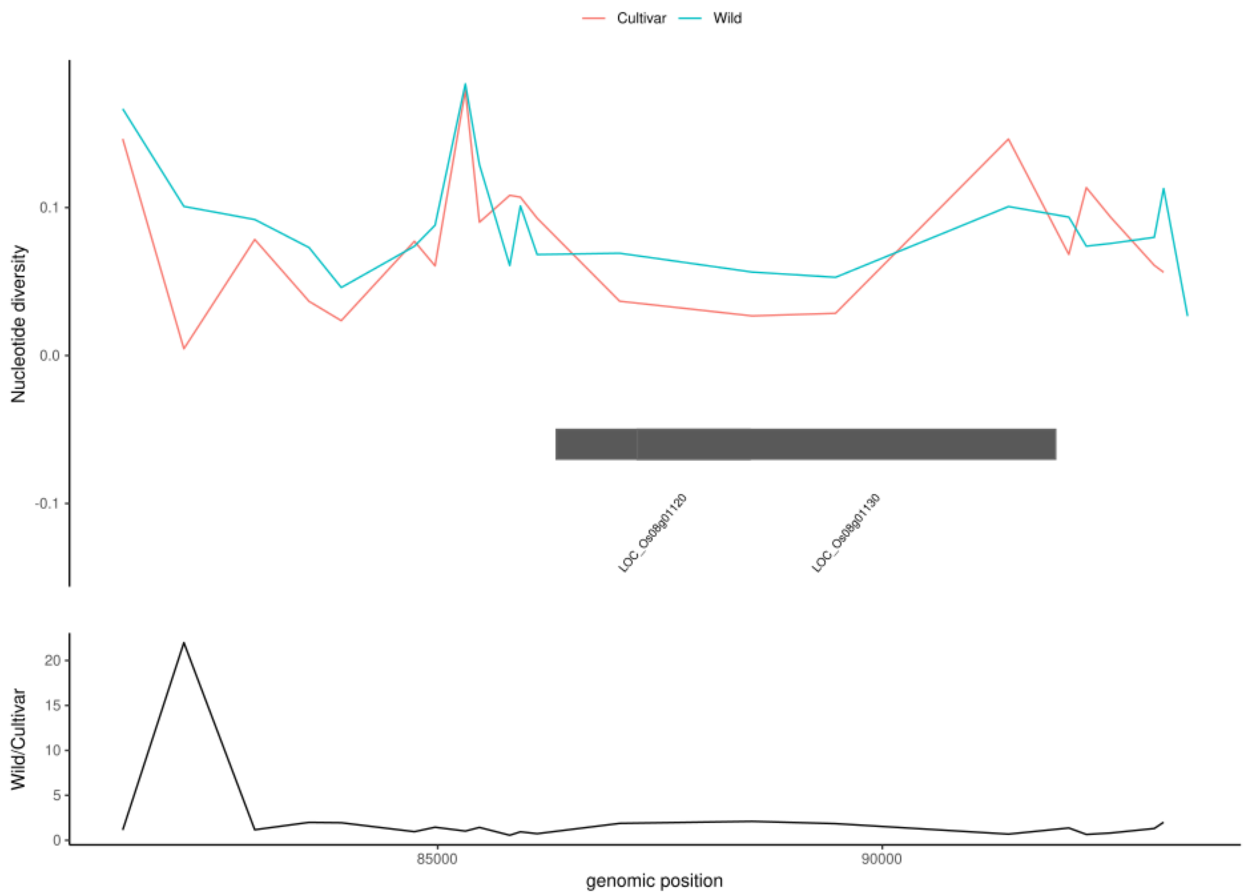

Os08g0101500

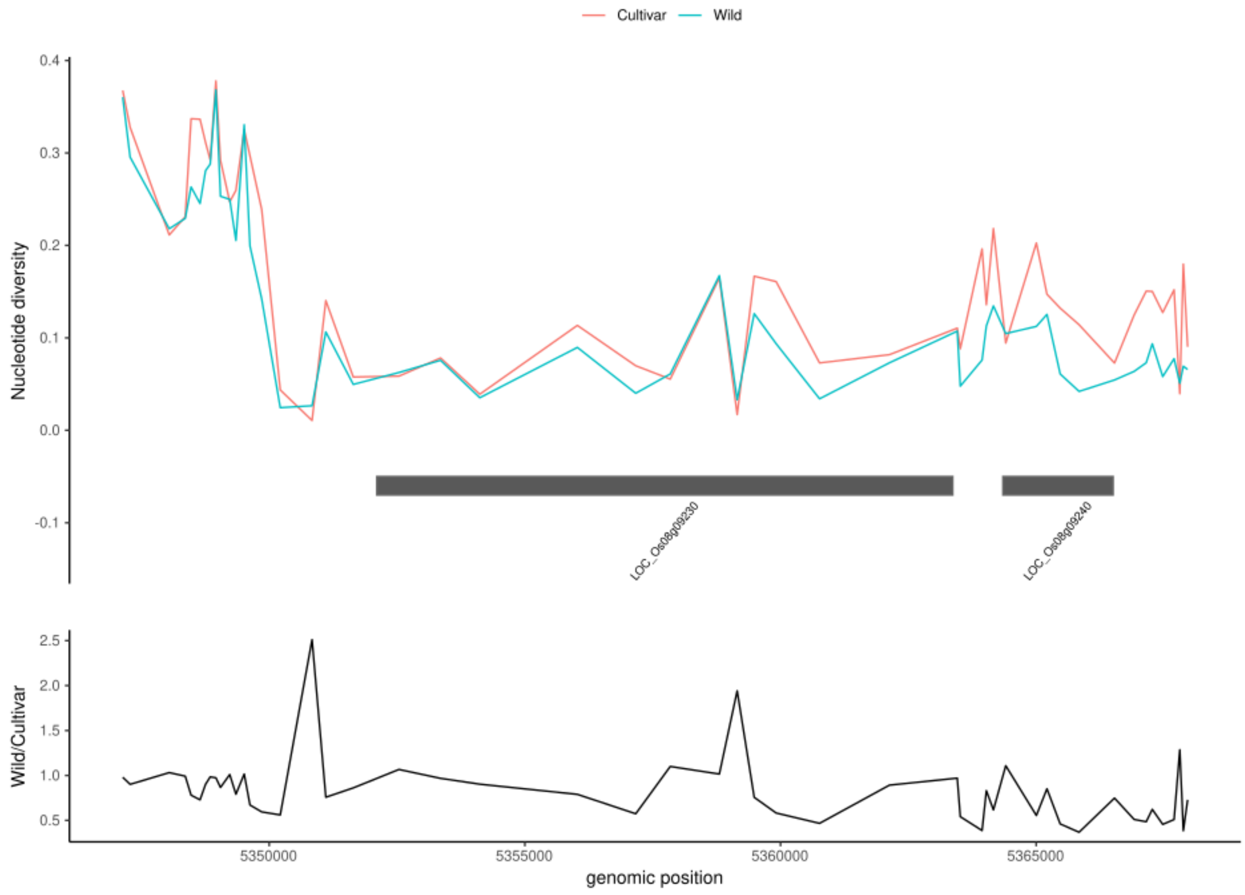

Os08g0191433

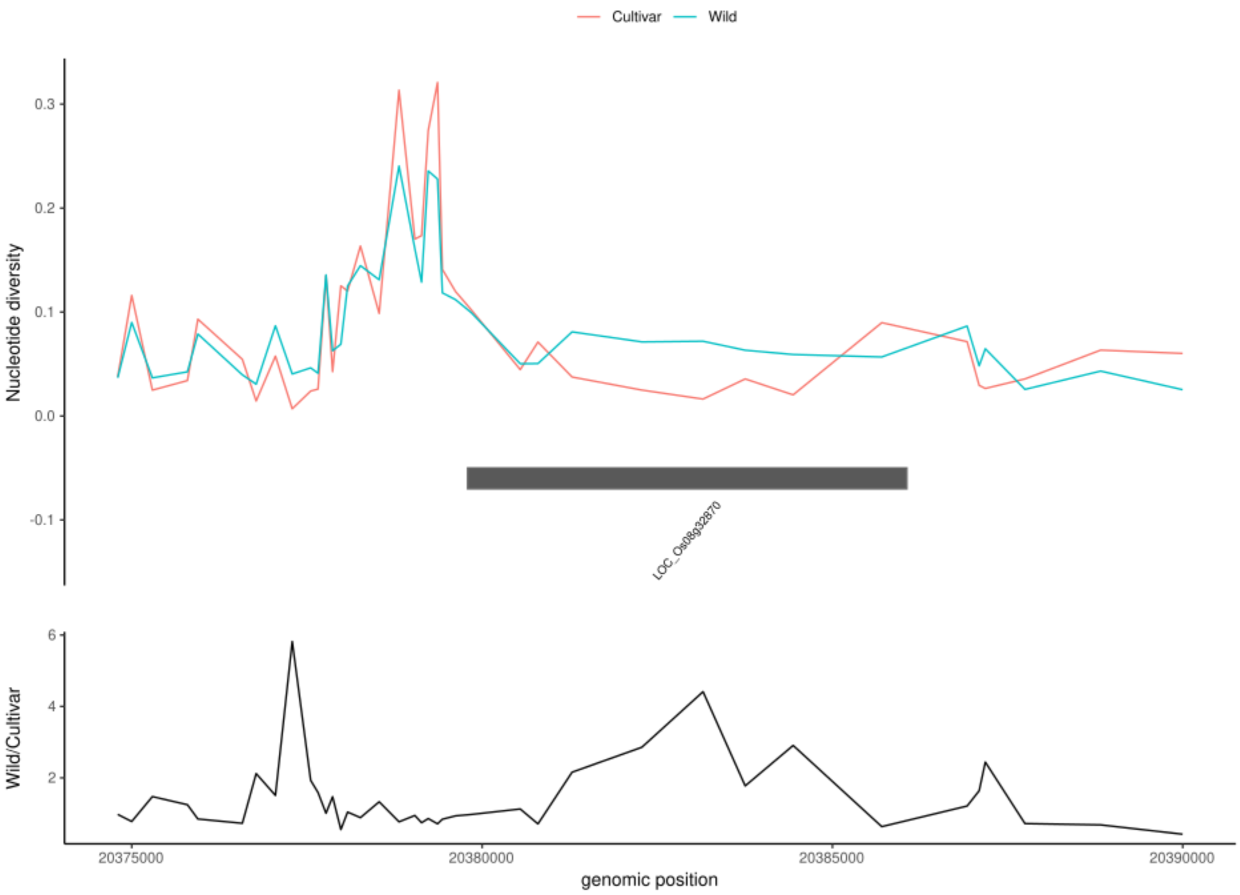

Os08g0424500

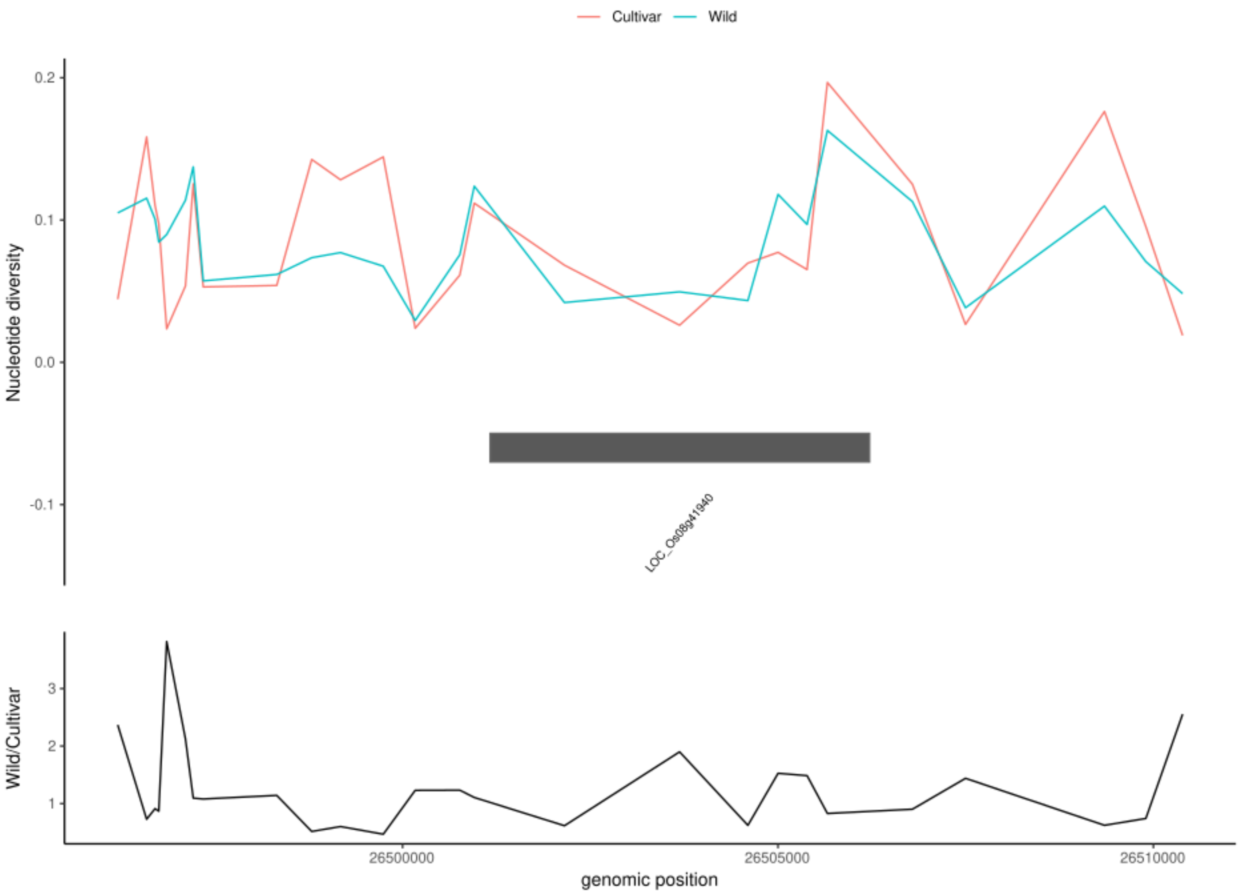

Os08g0531600

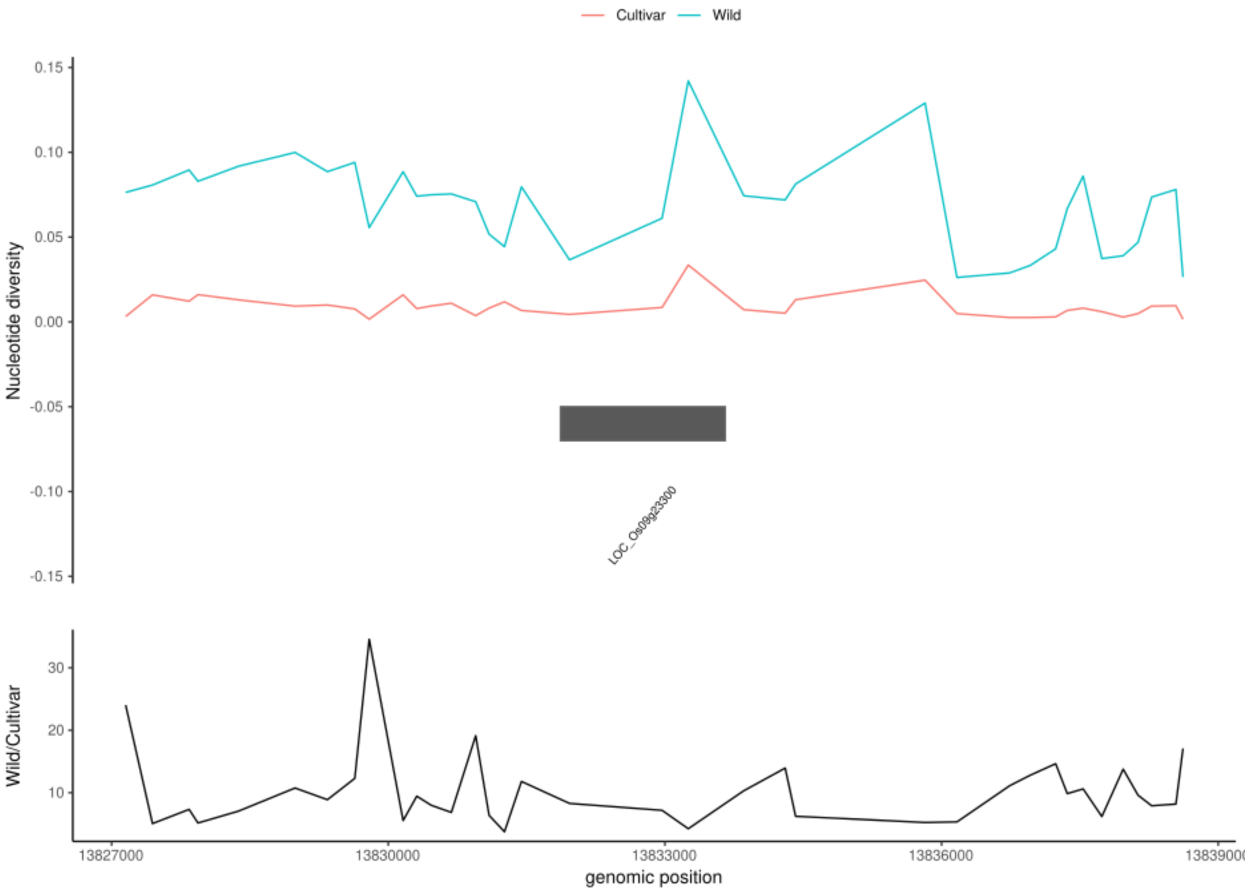

Os09g0396900

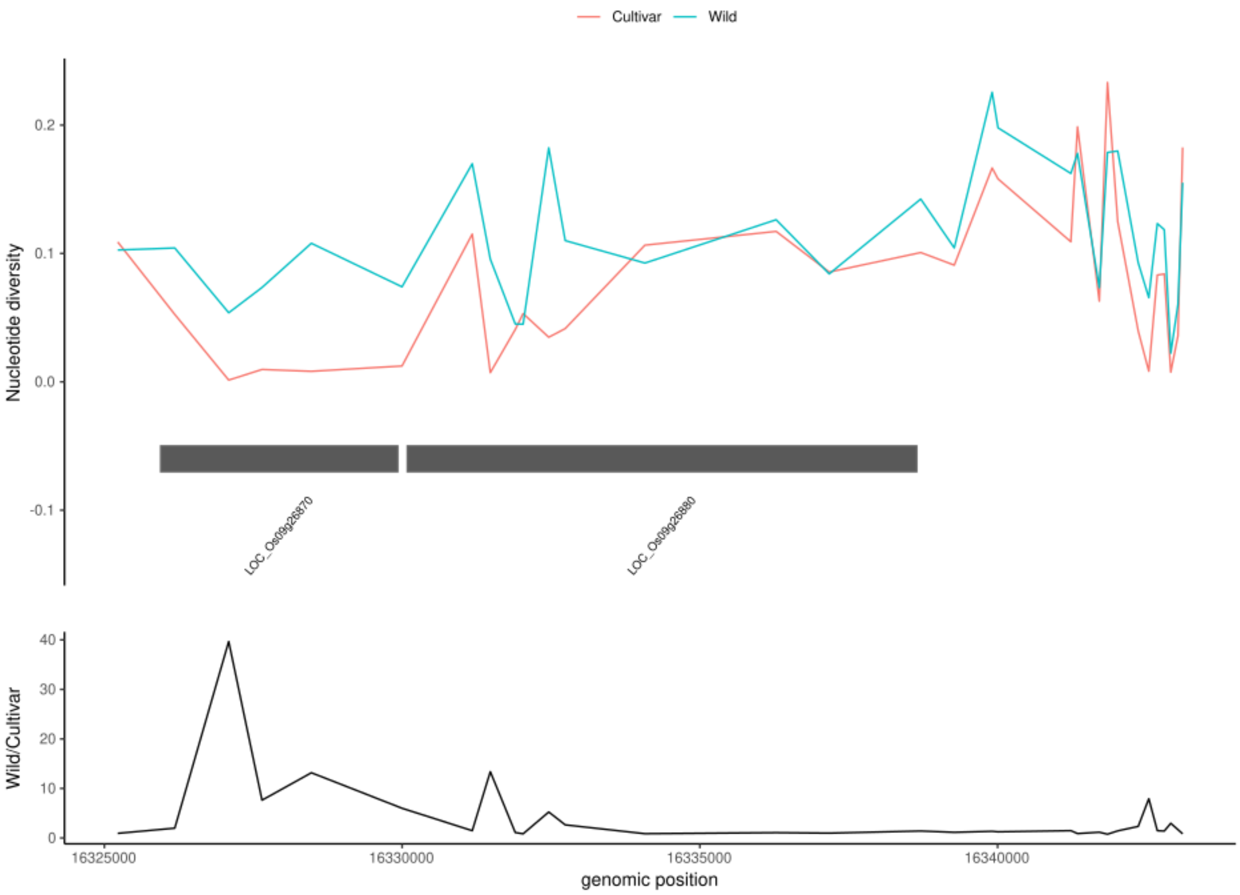

Os09g0440300

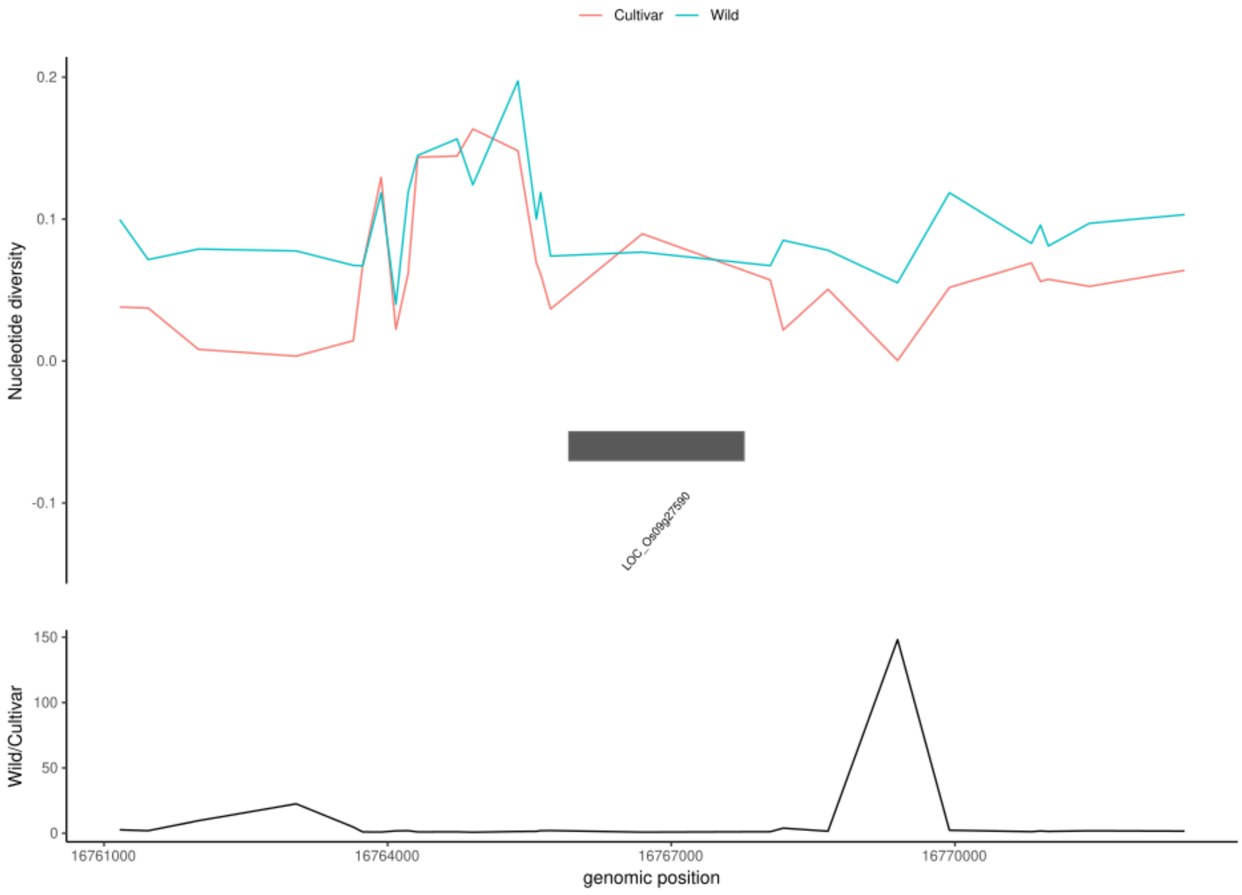

Os09g0448500

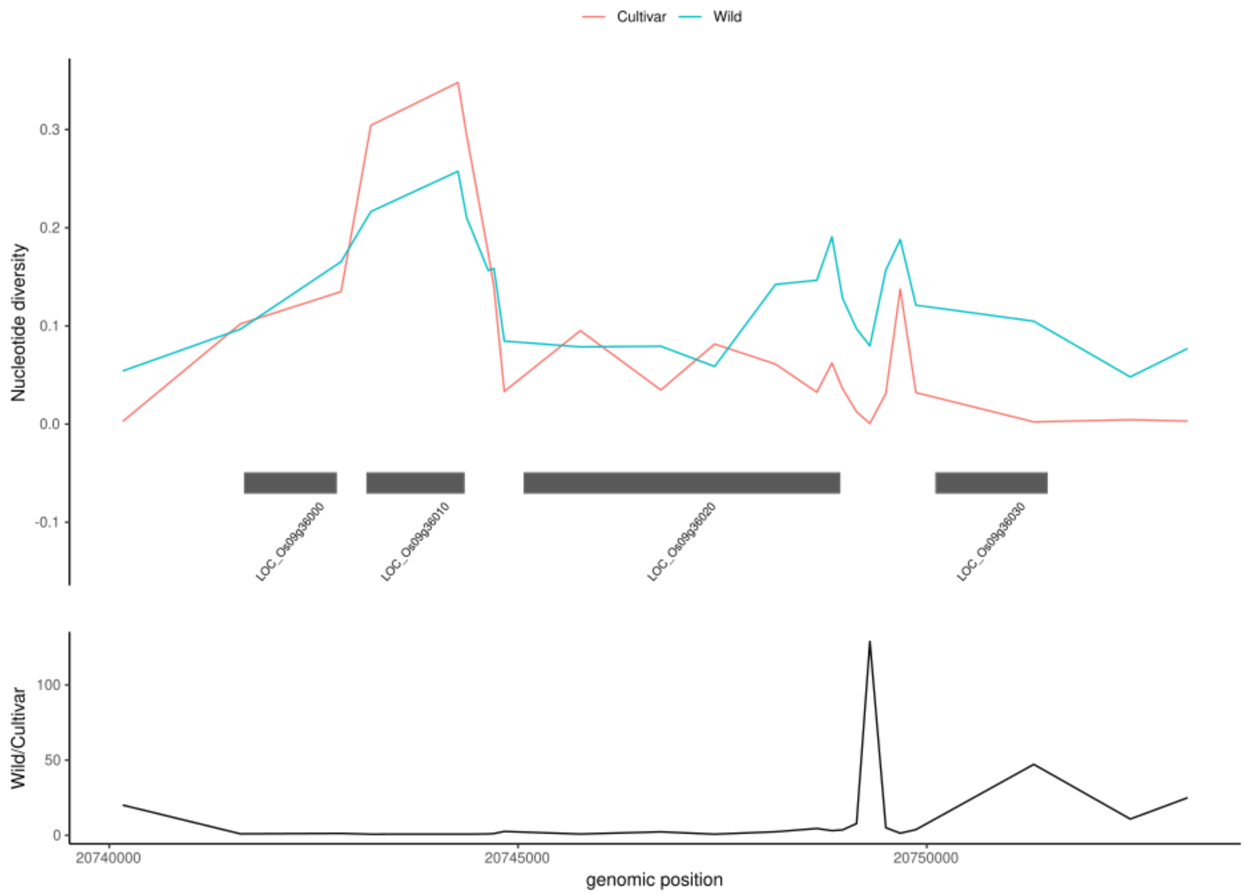

Os09g0529700

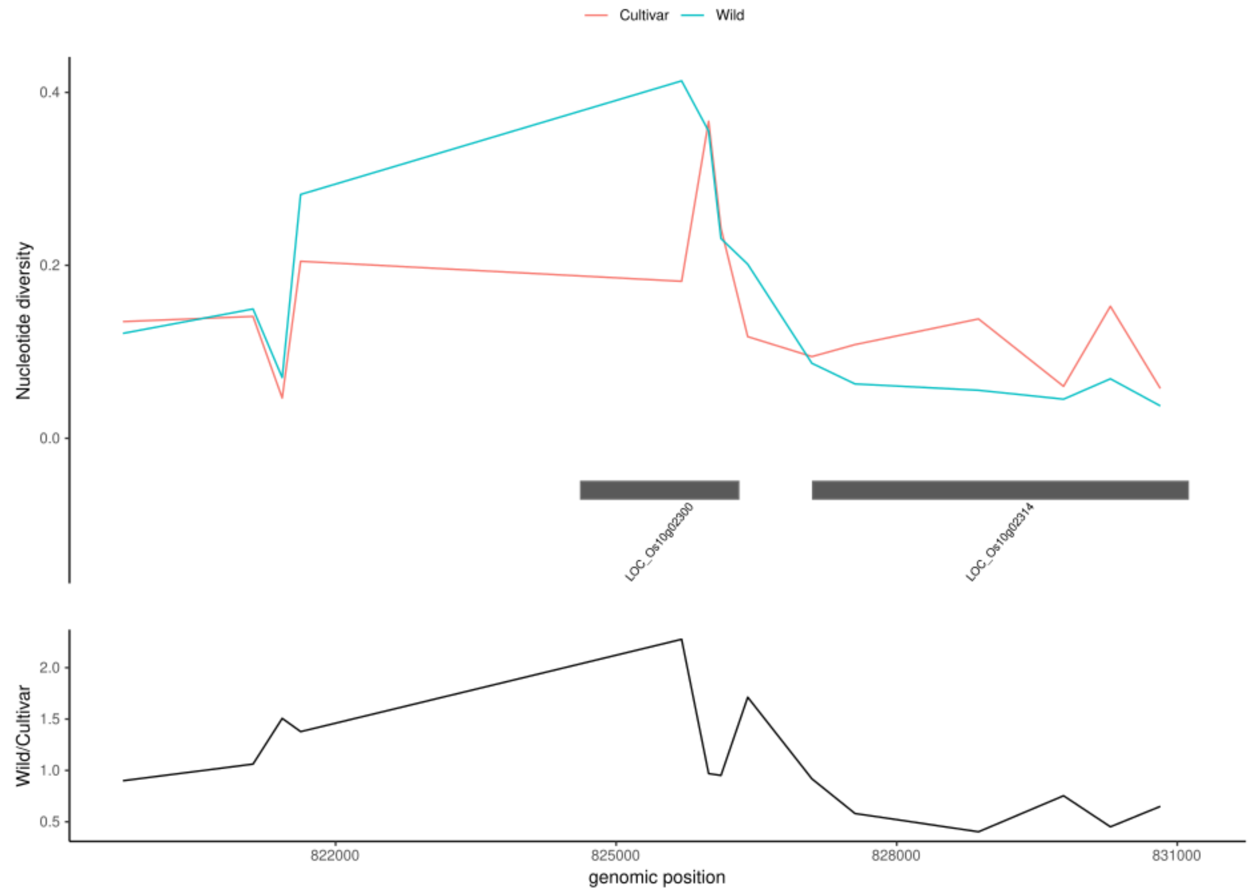

Os10g0112100

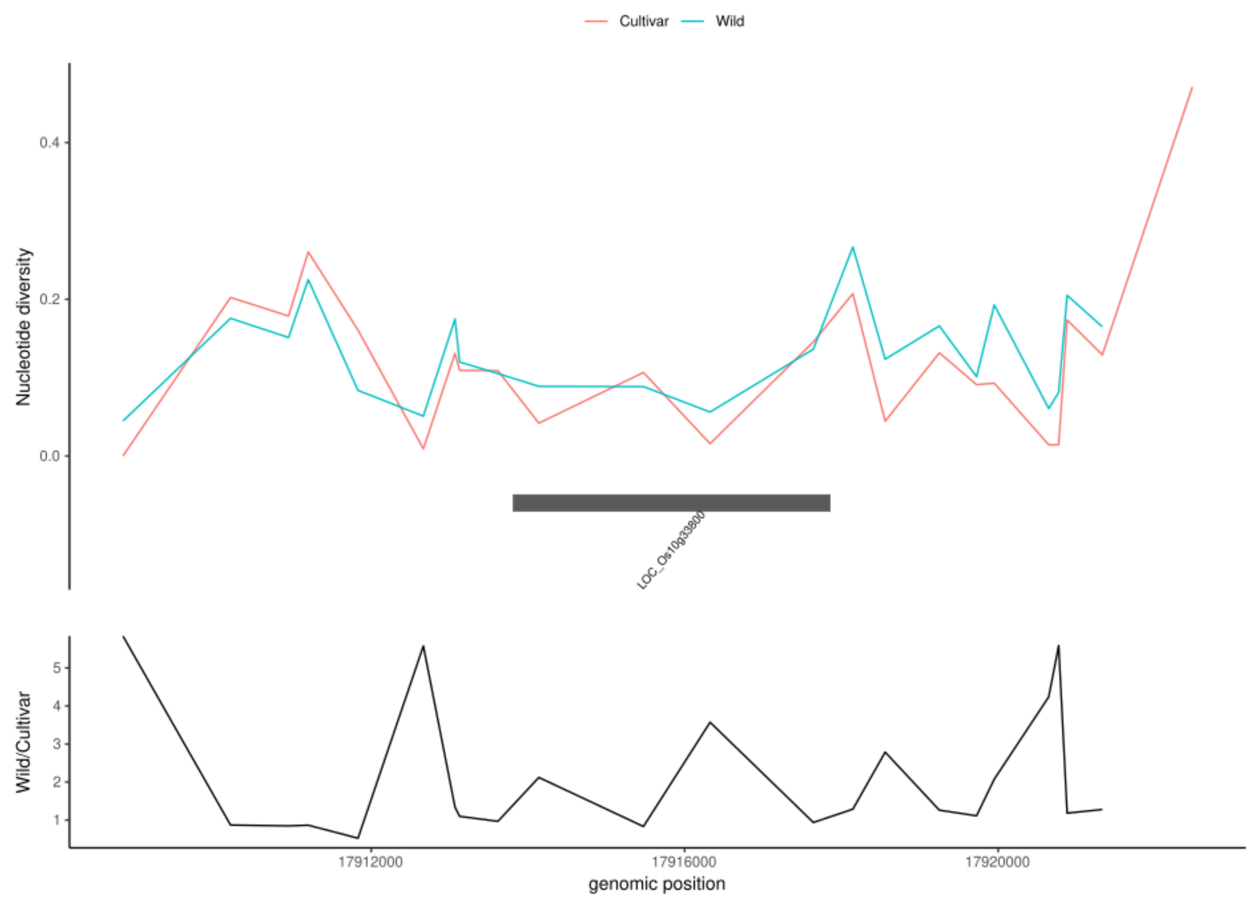

Os10g0478200

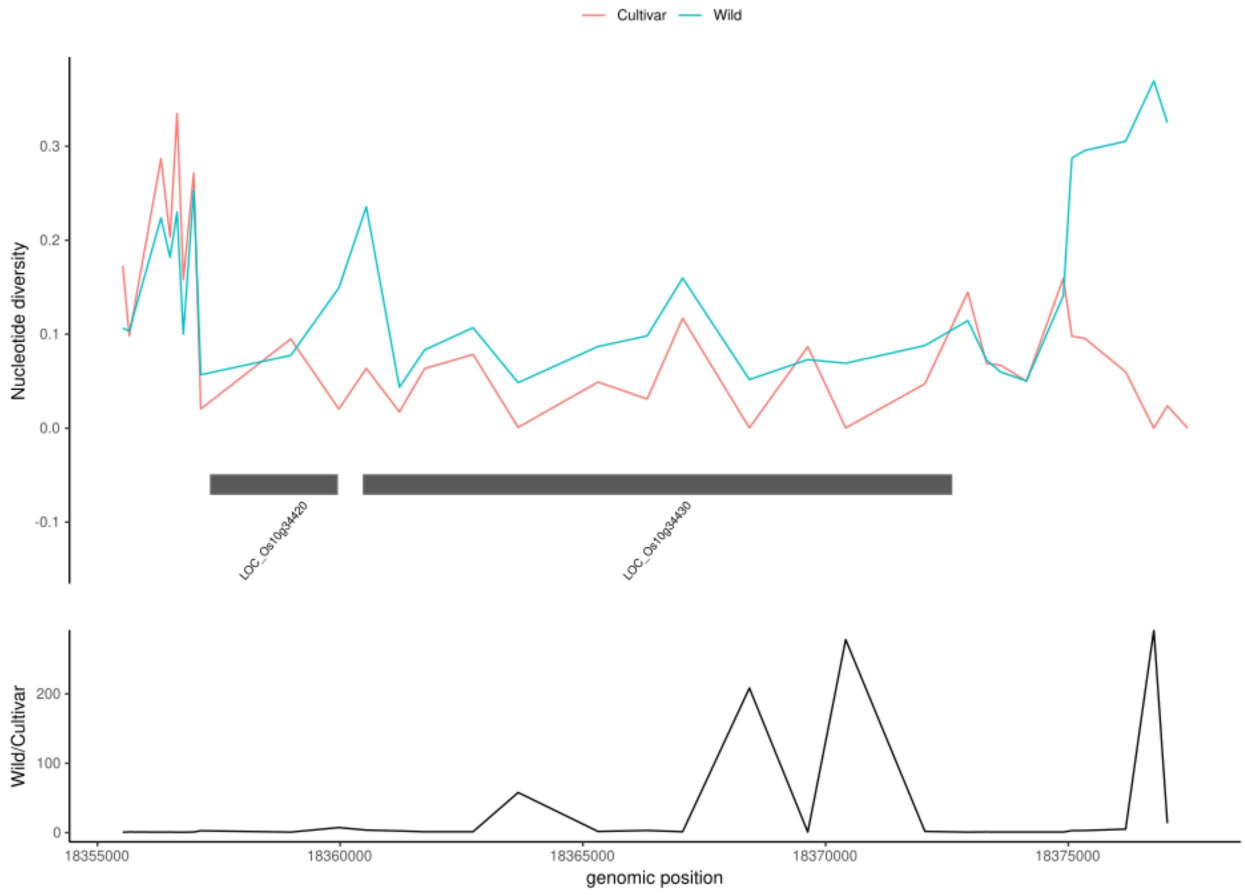

Os10g0485600

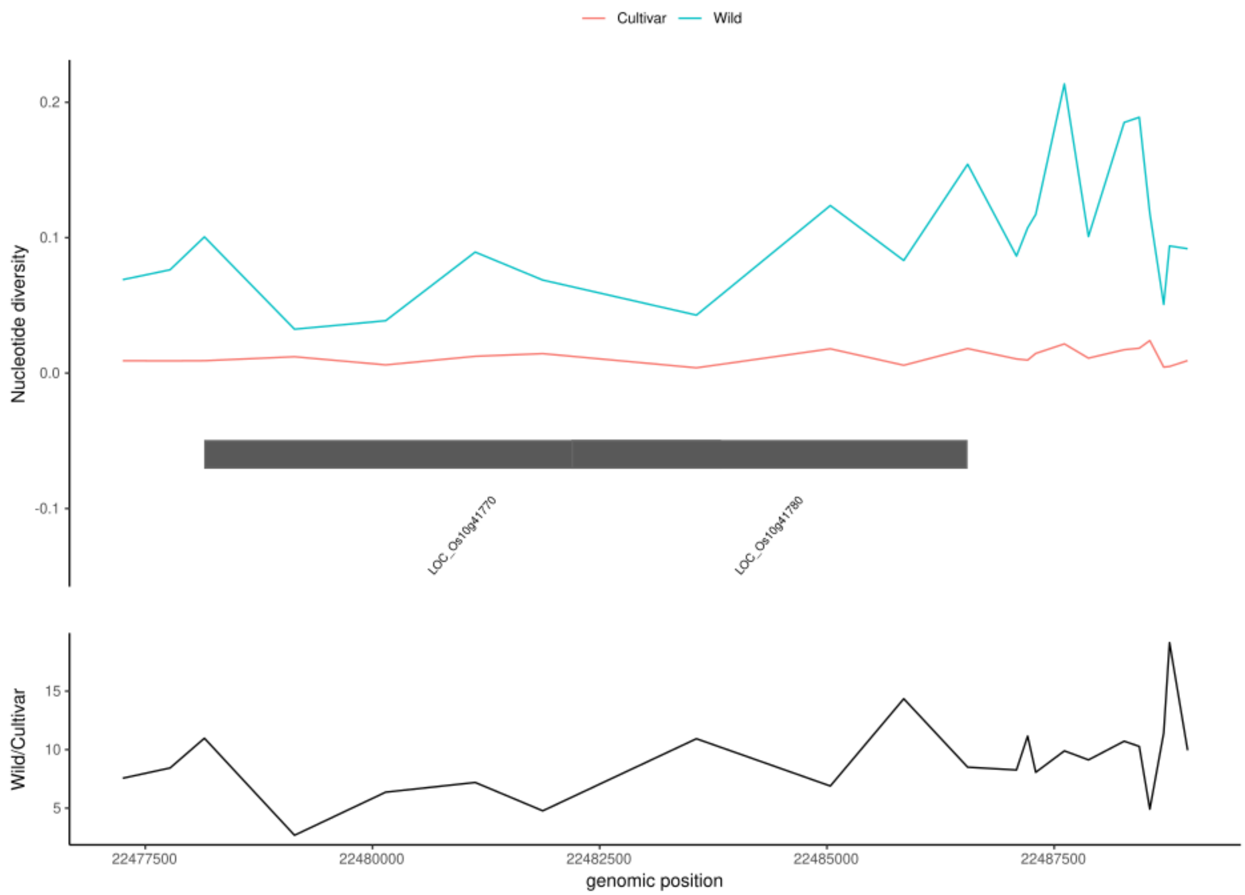

Os10g0567400

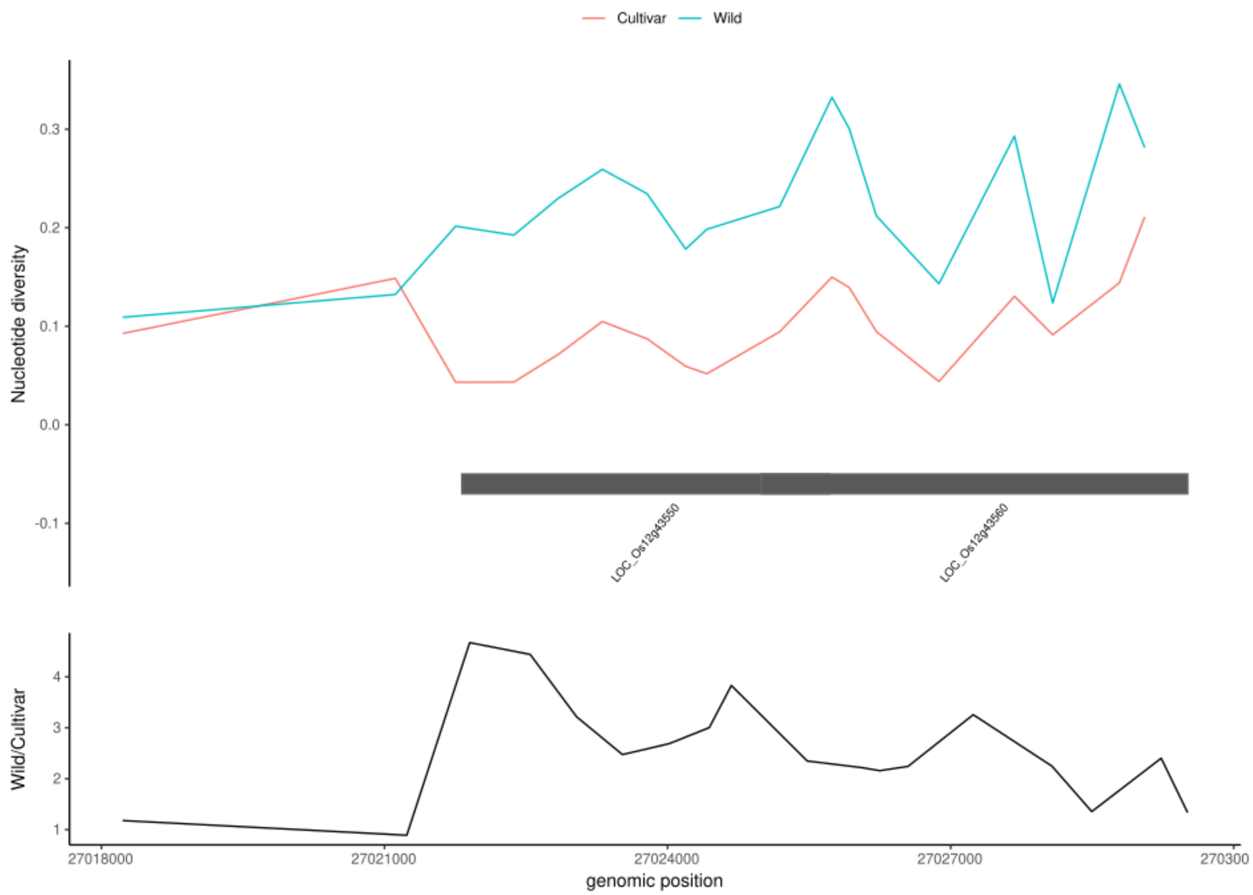

Os12g0631100

Supplementary Dataset 1. Haplotypic network of 80 nutritional and cooking quality related genes depicting number of haplotypes and their interactions with different rice types

(Os08g0424500)

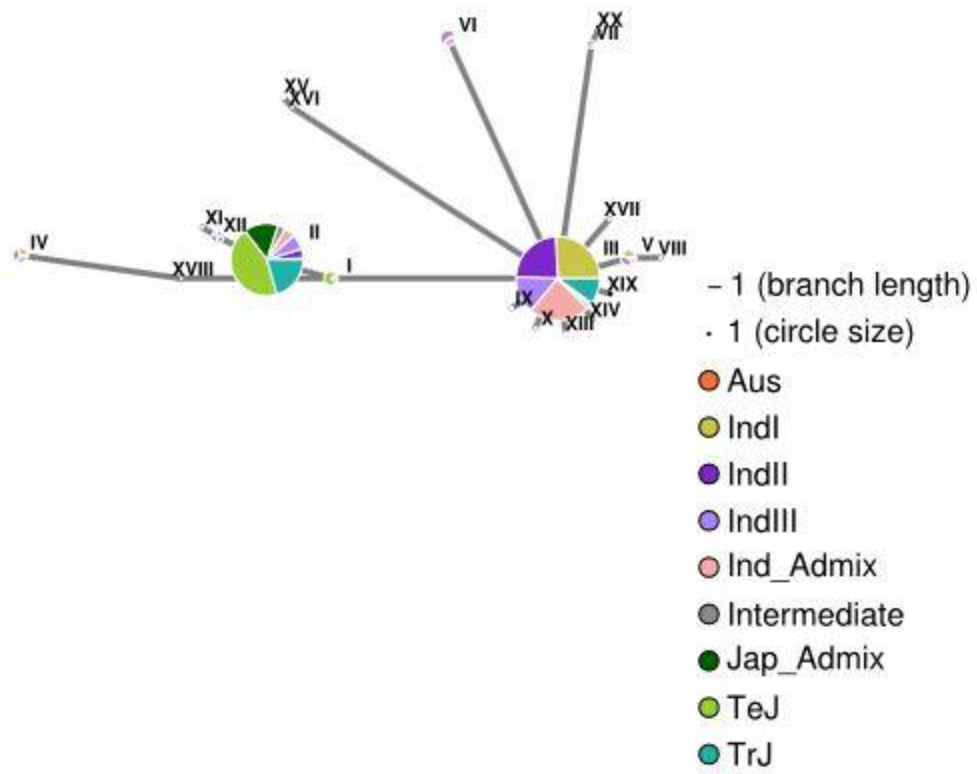

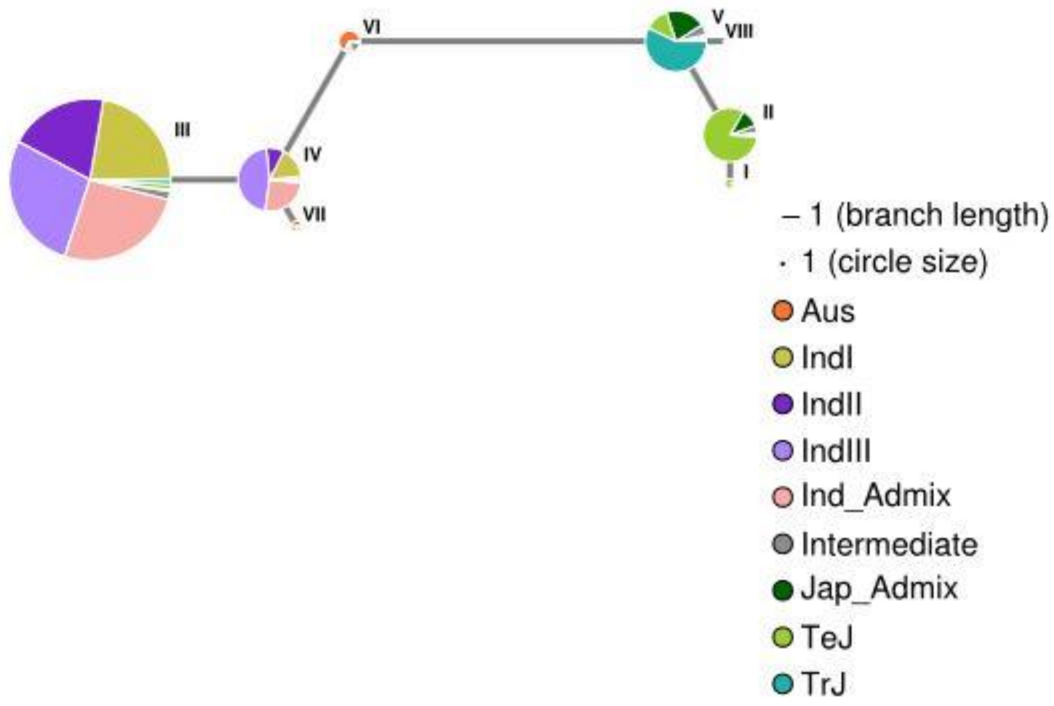

Os01g0179400

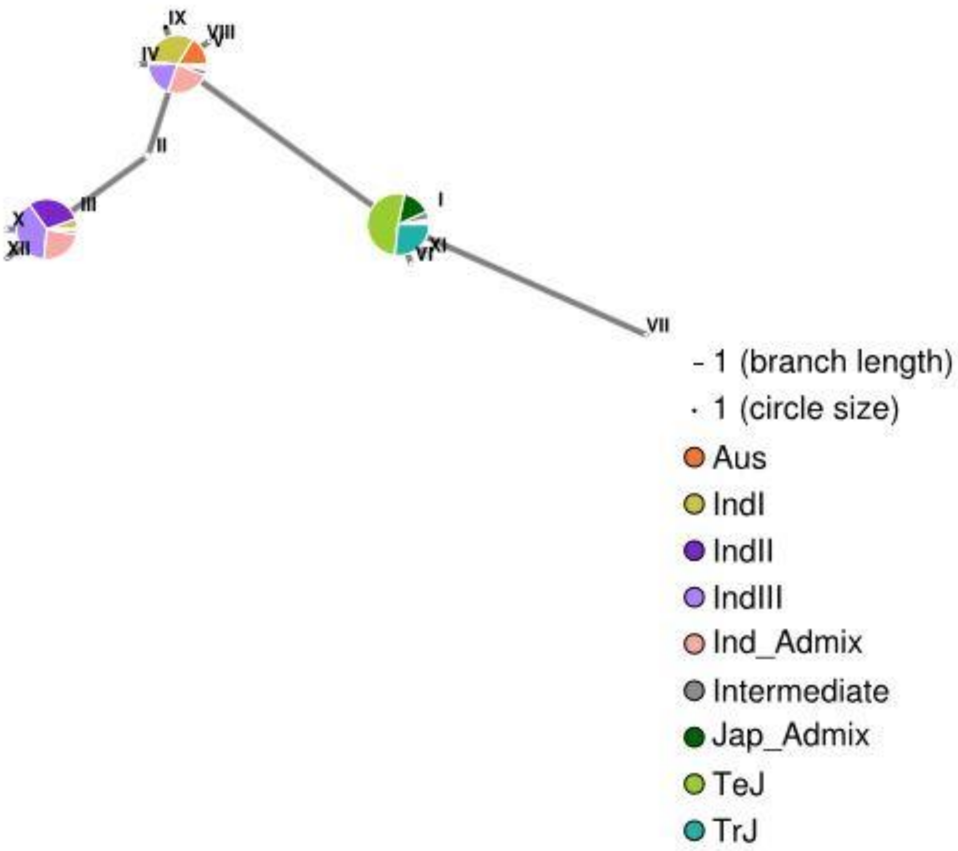

Os01g0190500

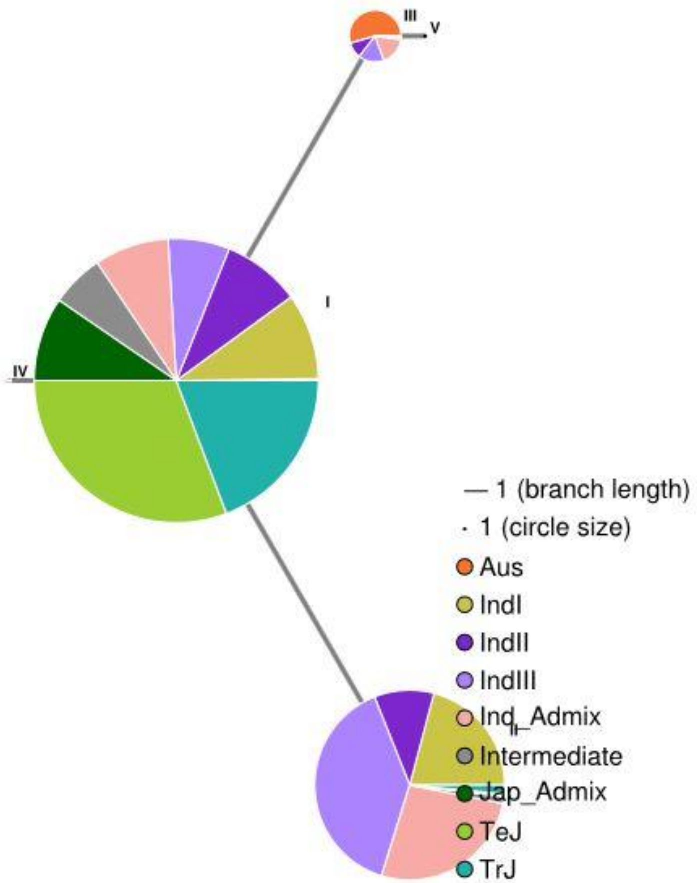

Os01g0265800

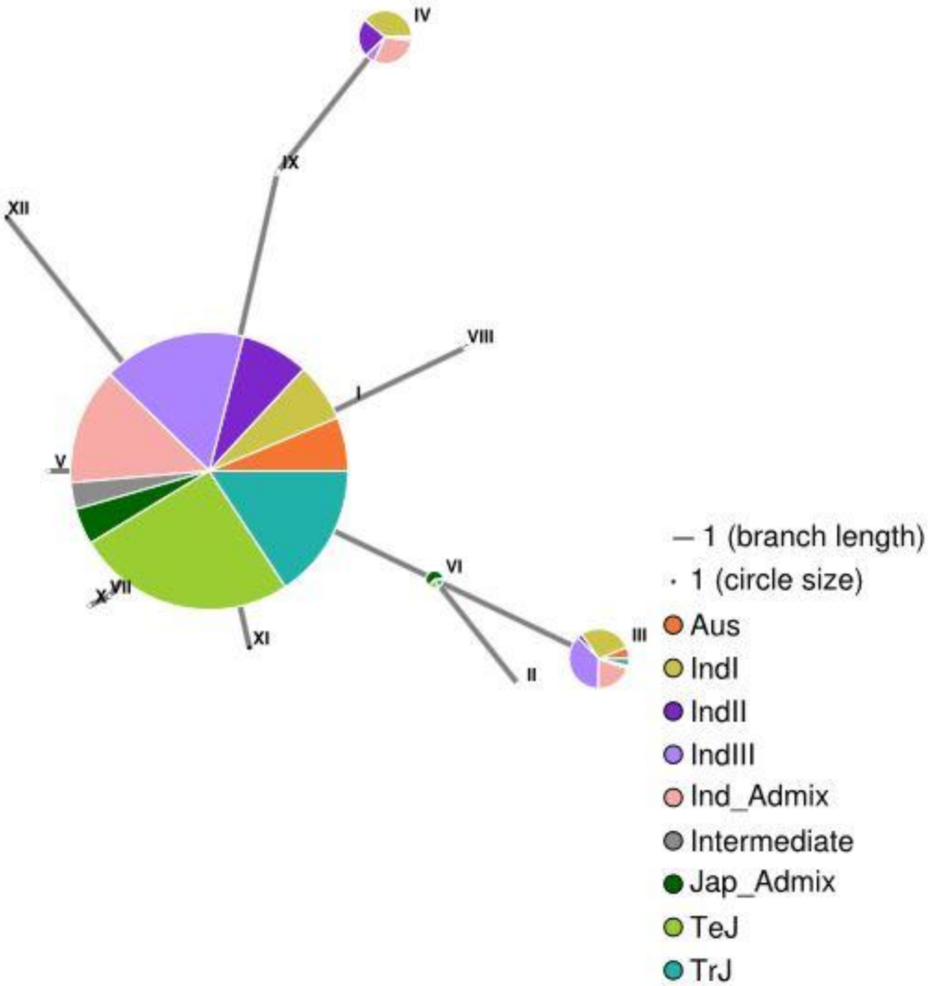

Os01g0367100

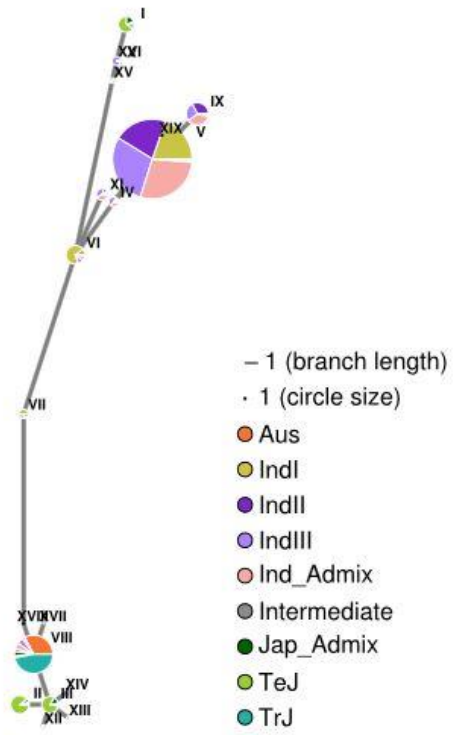

Os01g0633100

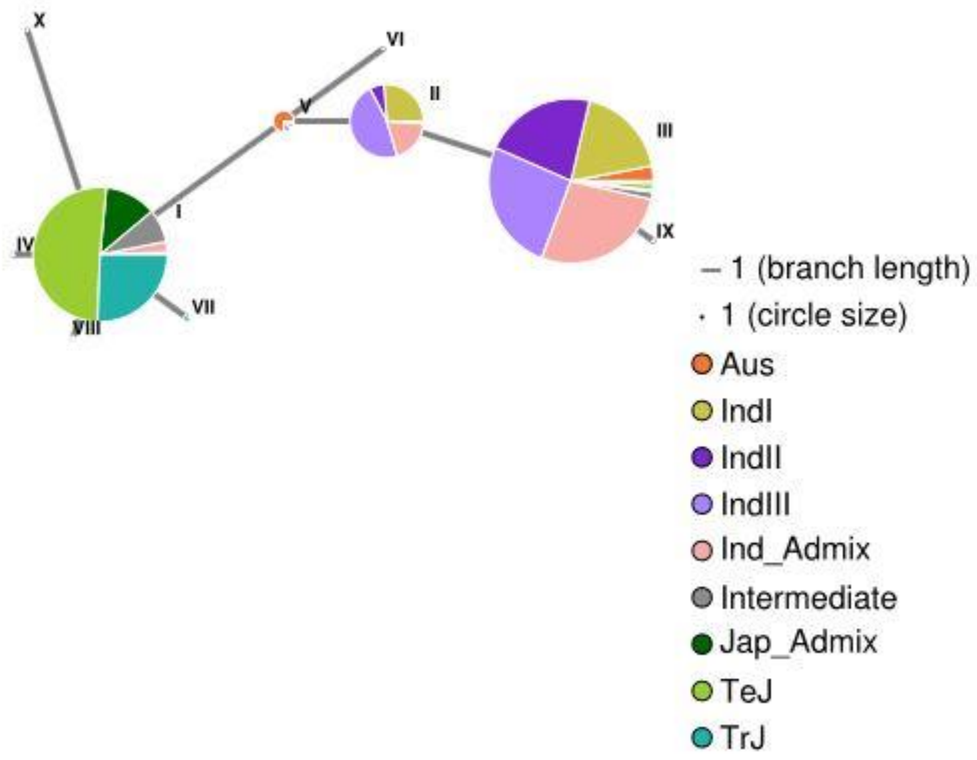

Os01g0878700

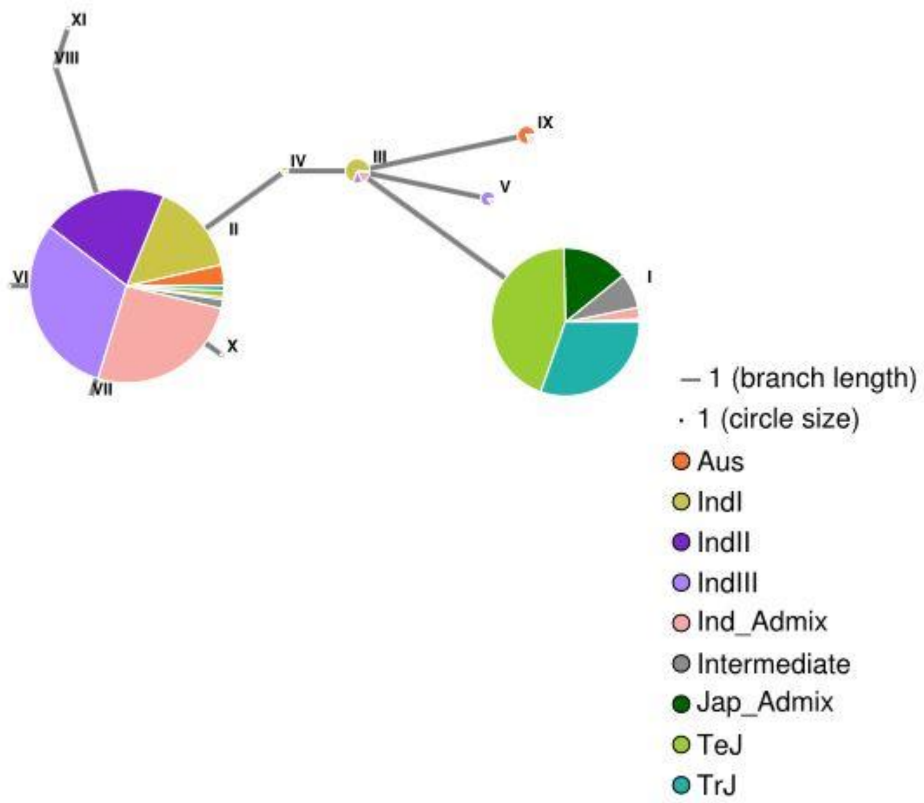

Os01g0883800

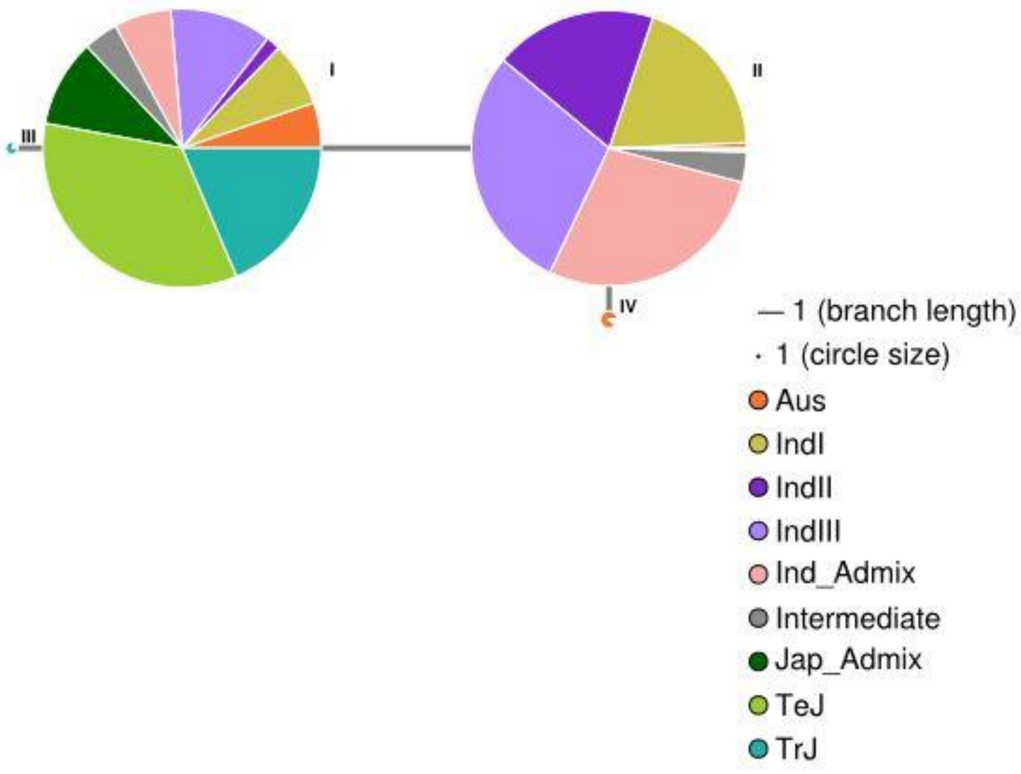

Os02g0102300

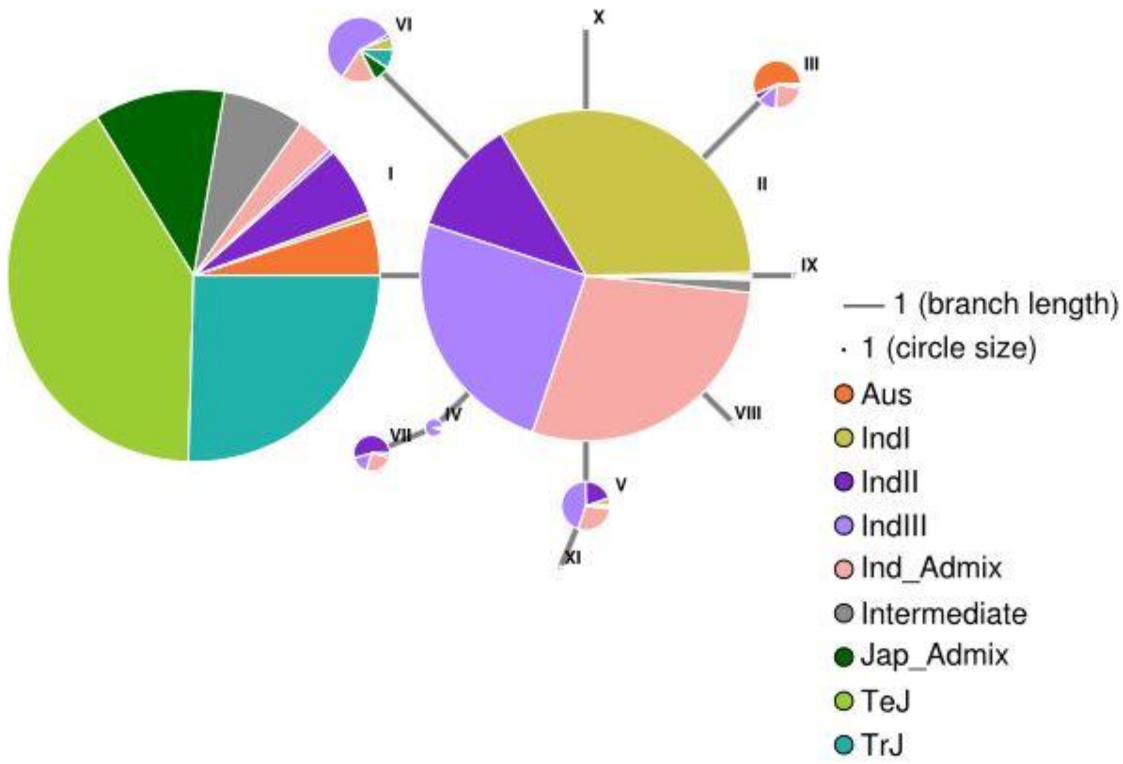

Os02g0117700

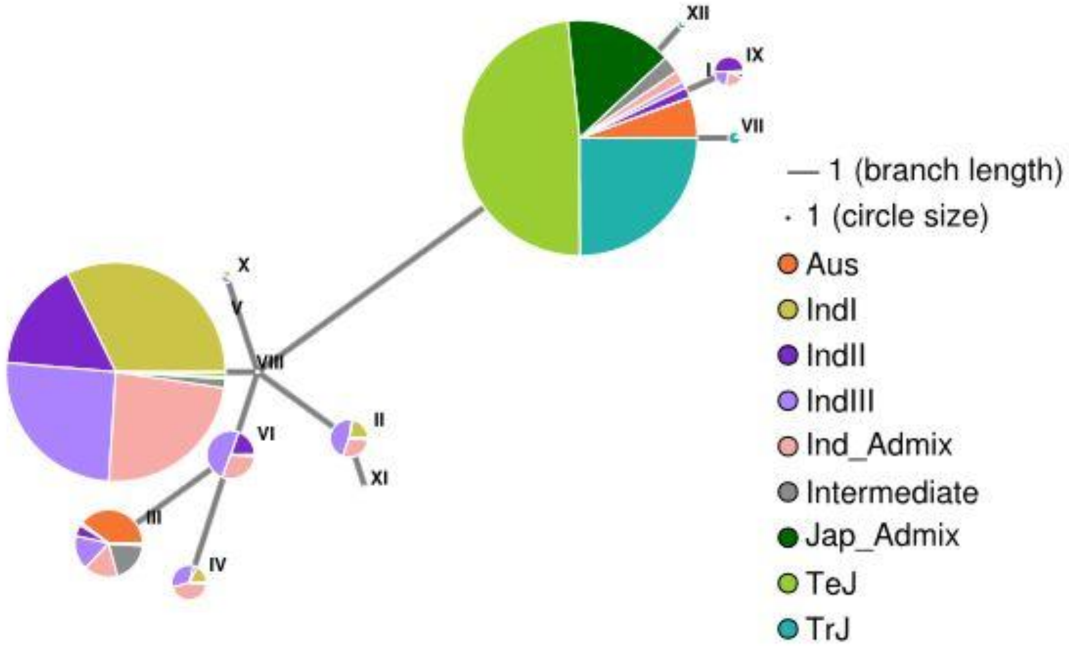

Os02g0196600

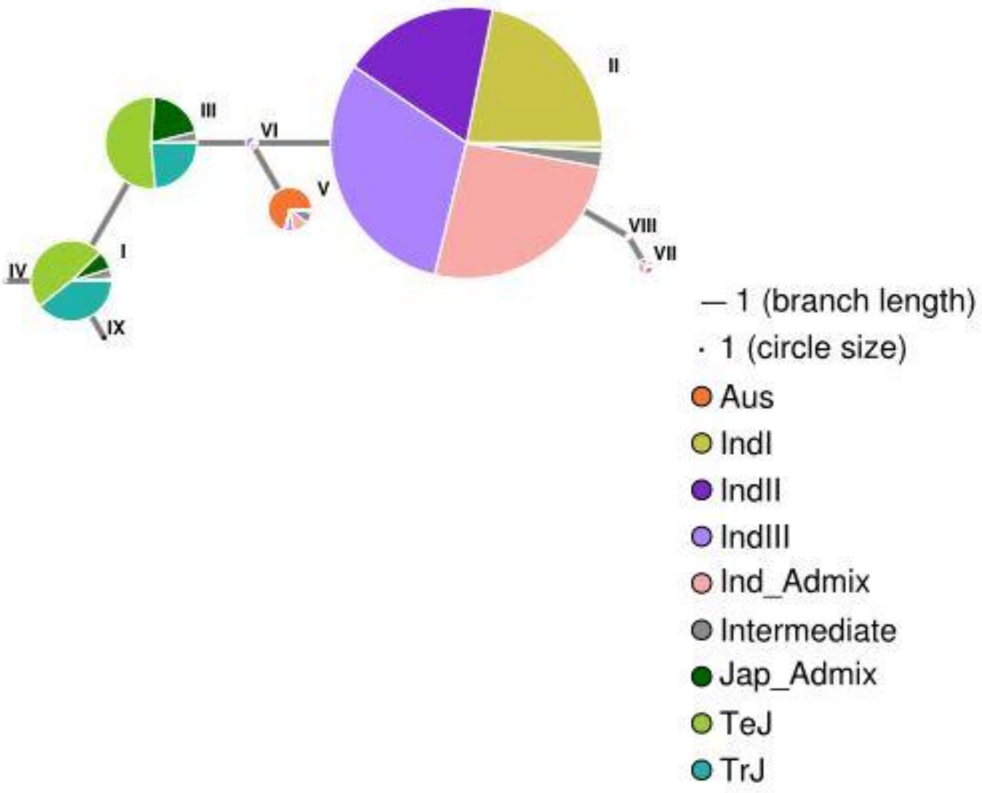

Os02g0202400

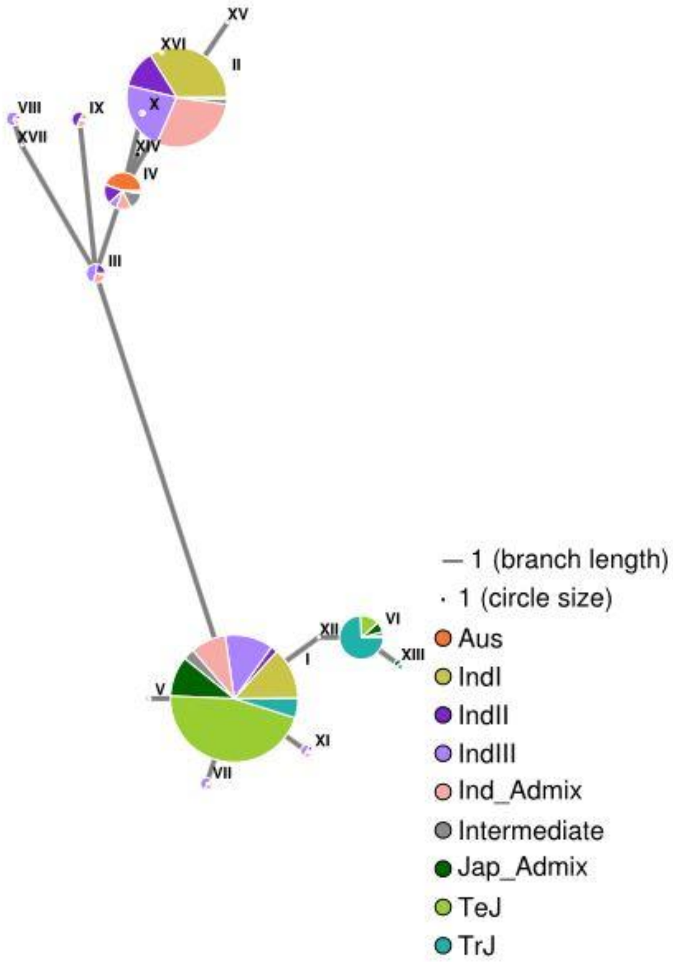

Os02g0244100

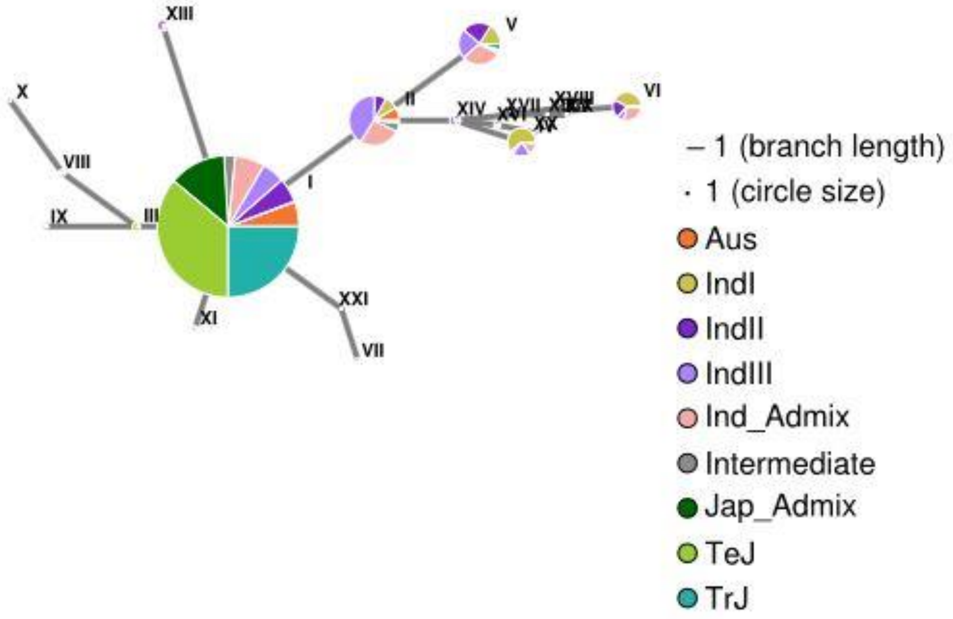

Os02g0612300

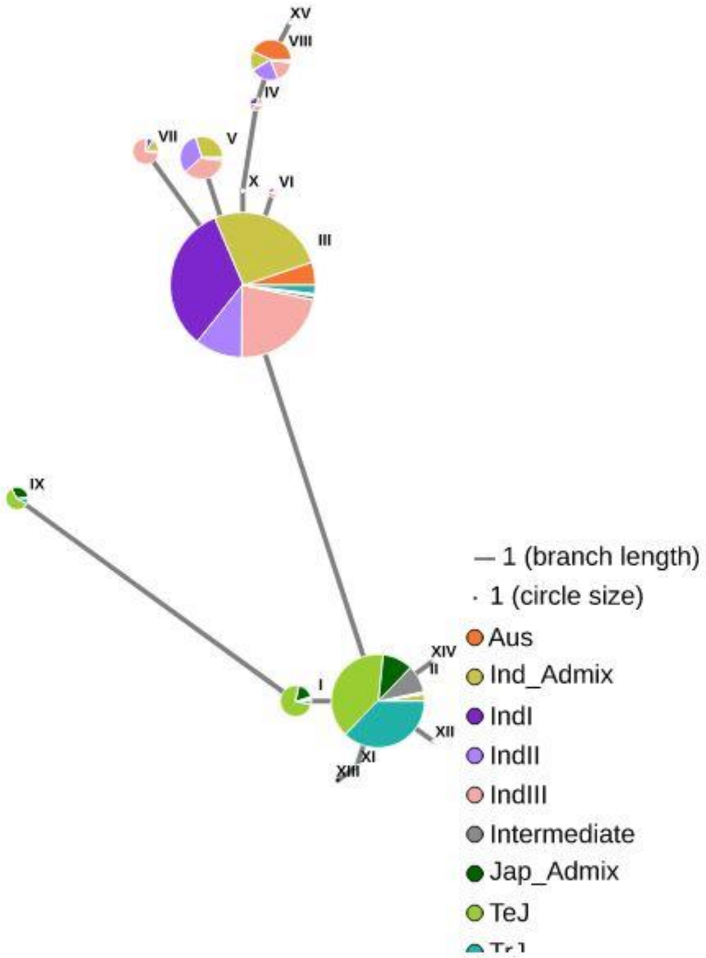

Os02g0649900

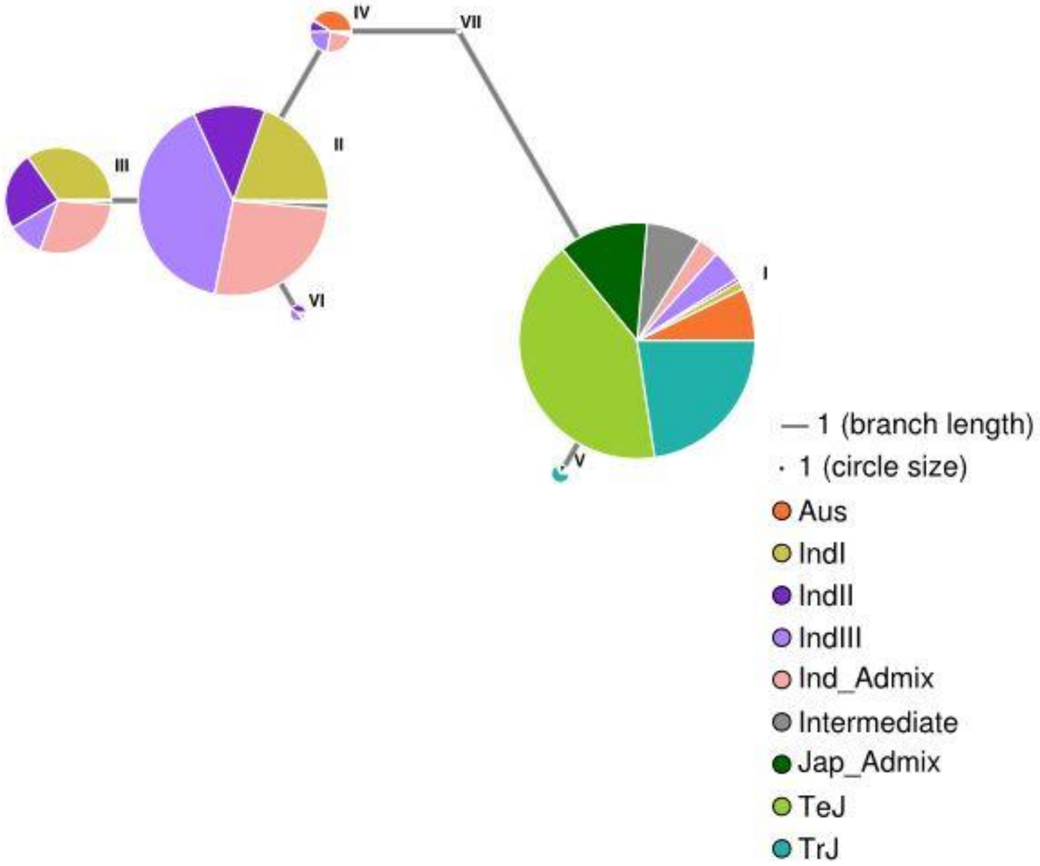

Os02g0682200

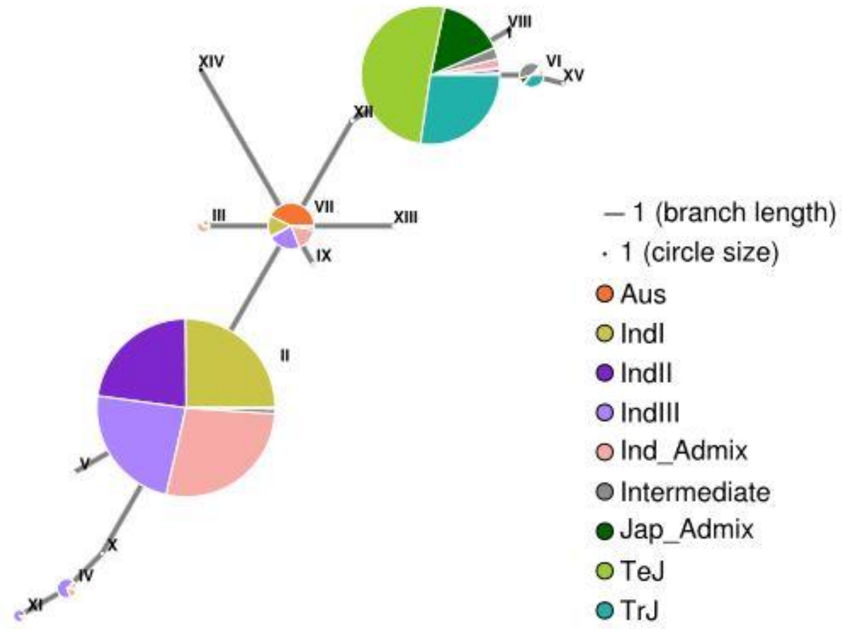

Os02g0716500

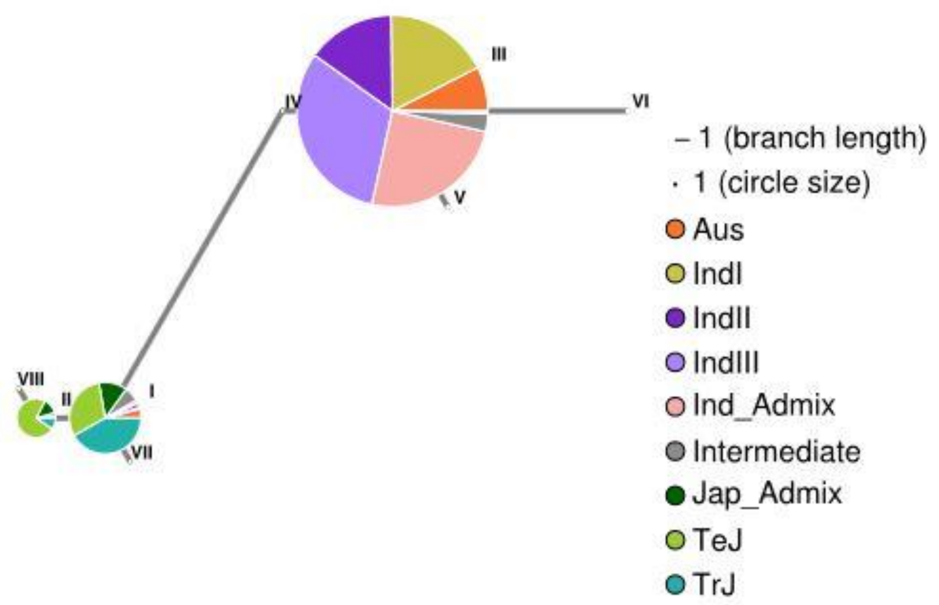

Os03g0128700

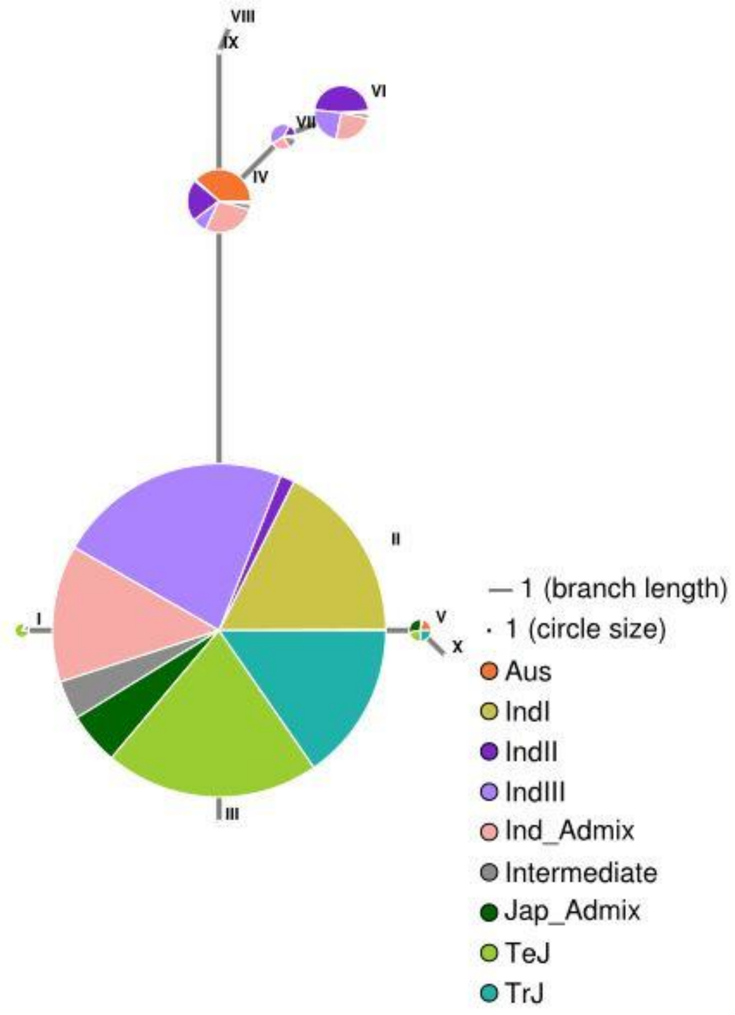

Os03g0129300

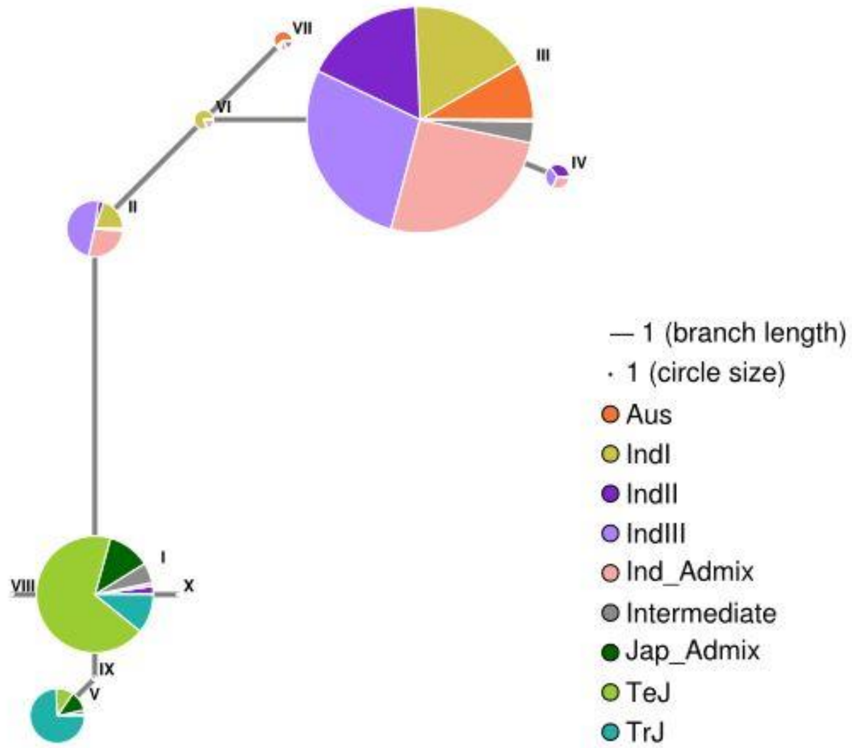

Os03g0142800

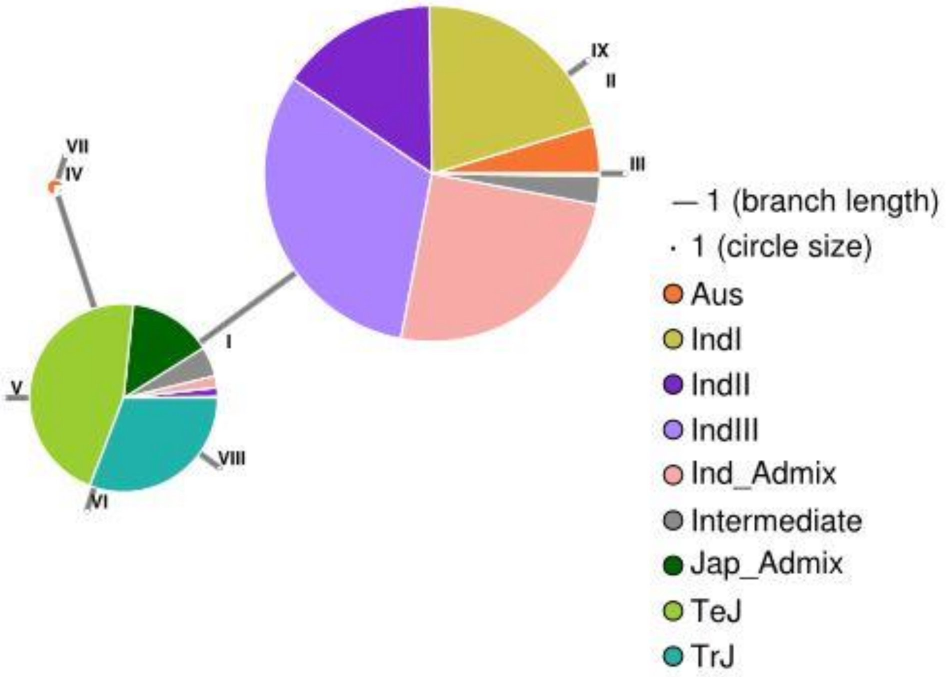

Os03g0150800

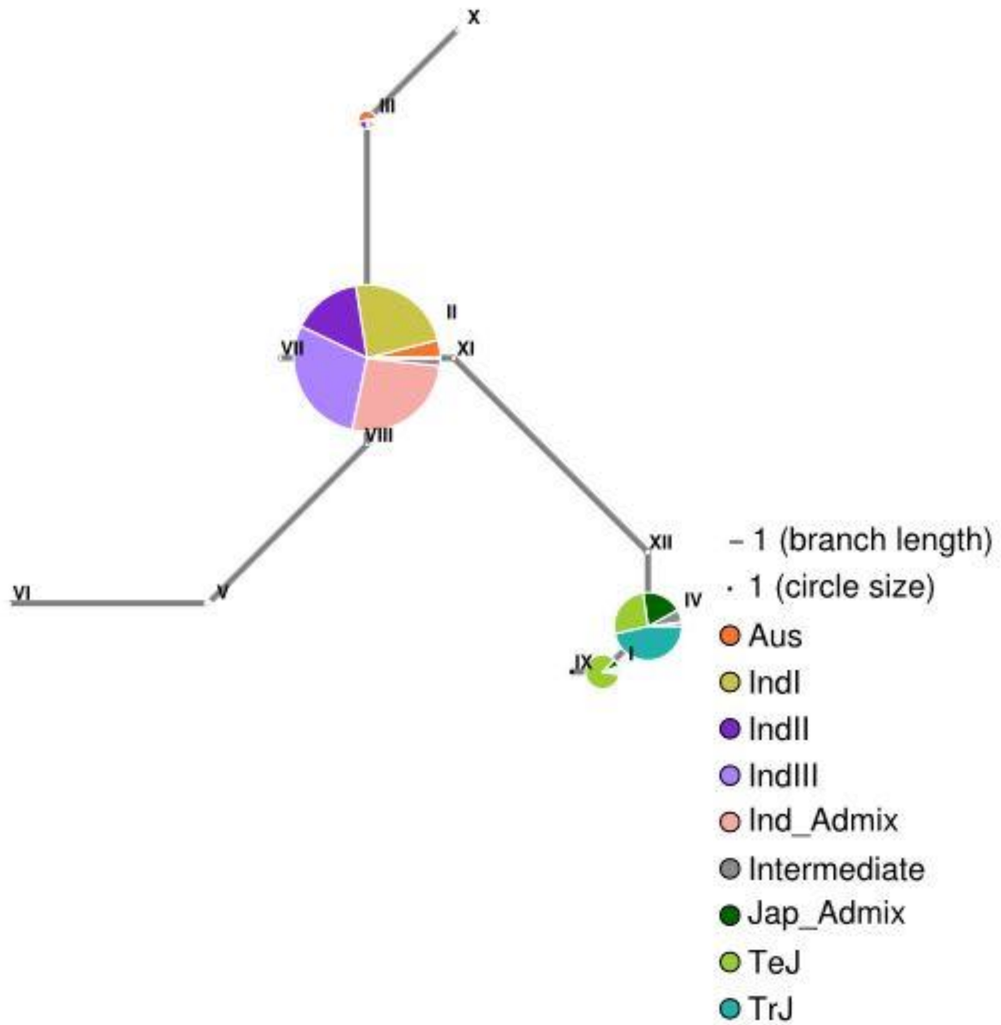

Os03g0192700

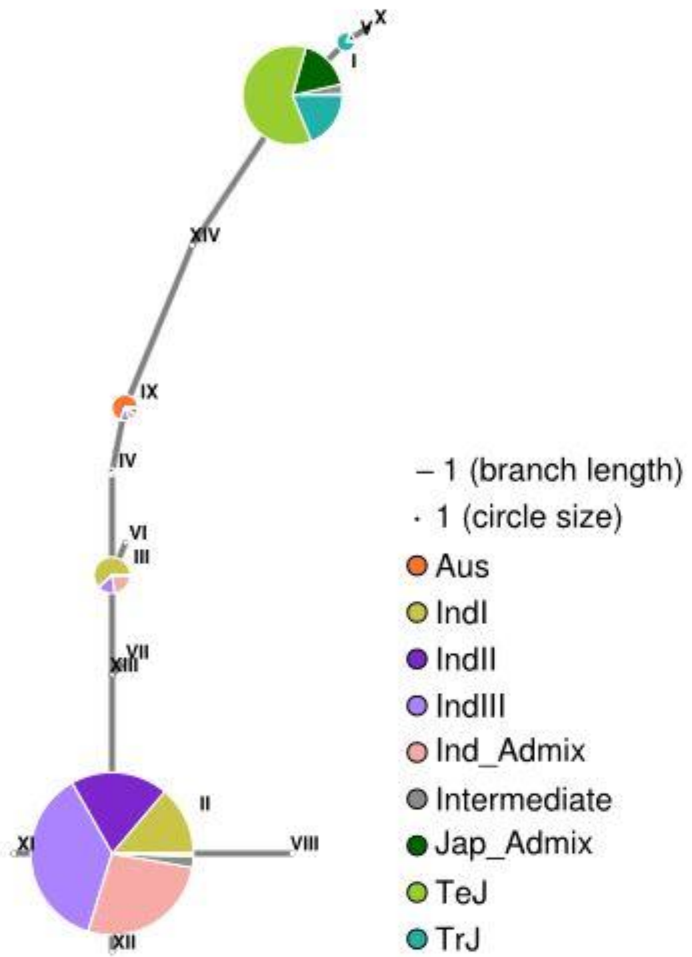

Os03g0216700

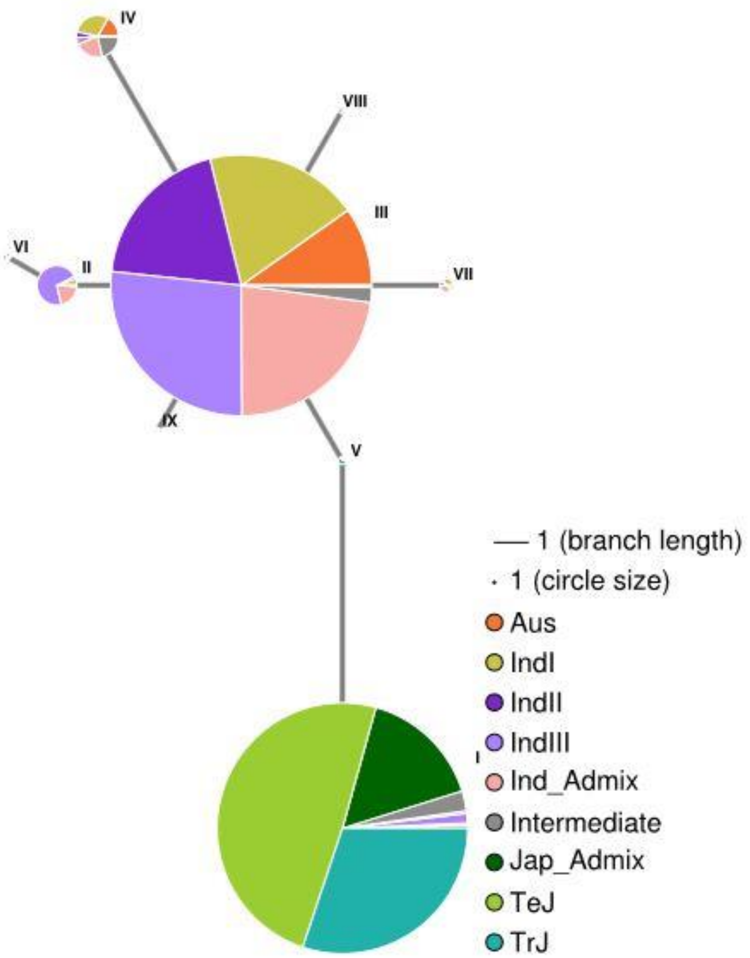

Os03g0237250

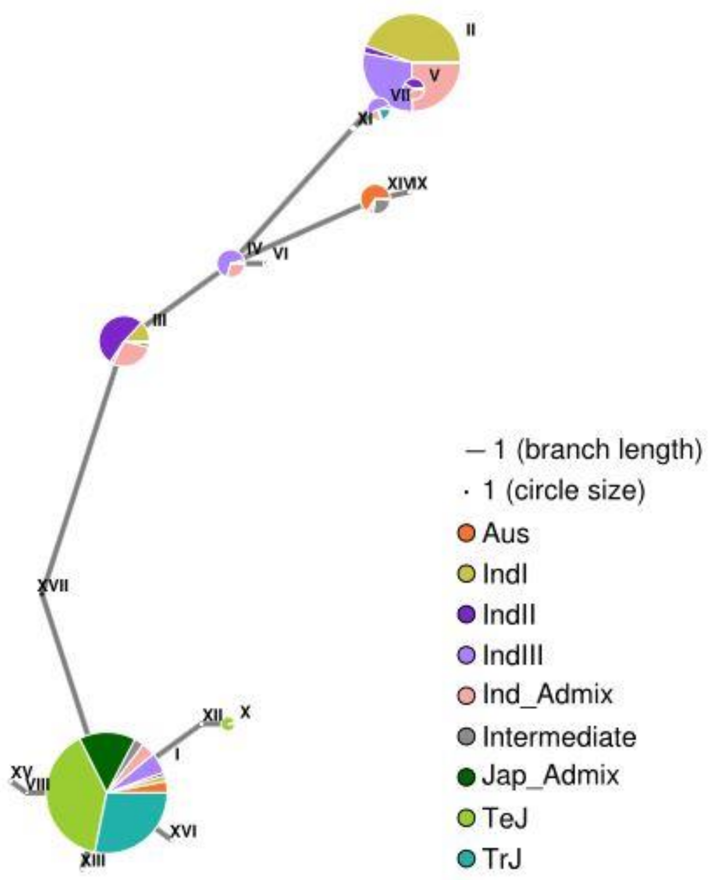

Os03g0264400

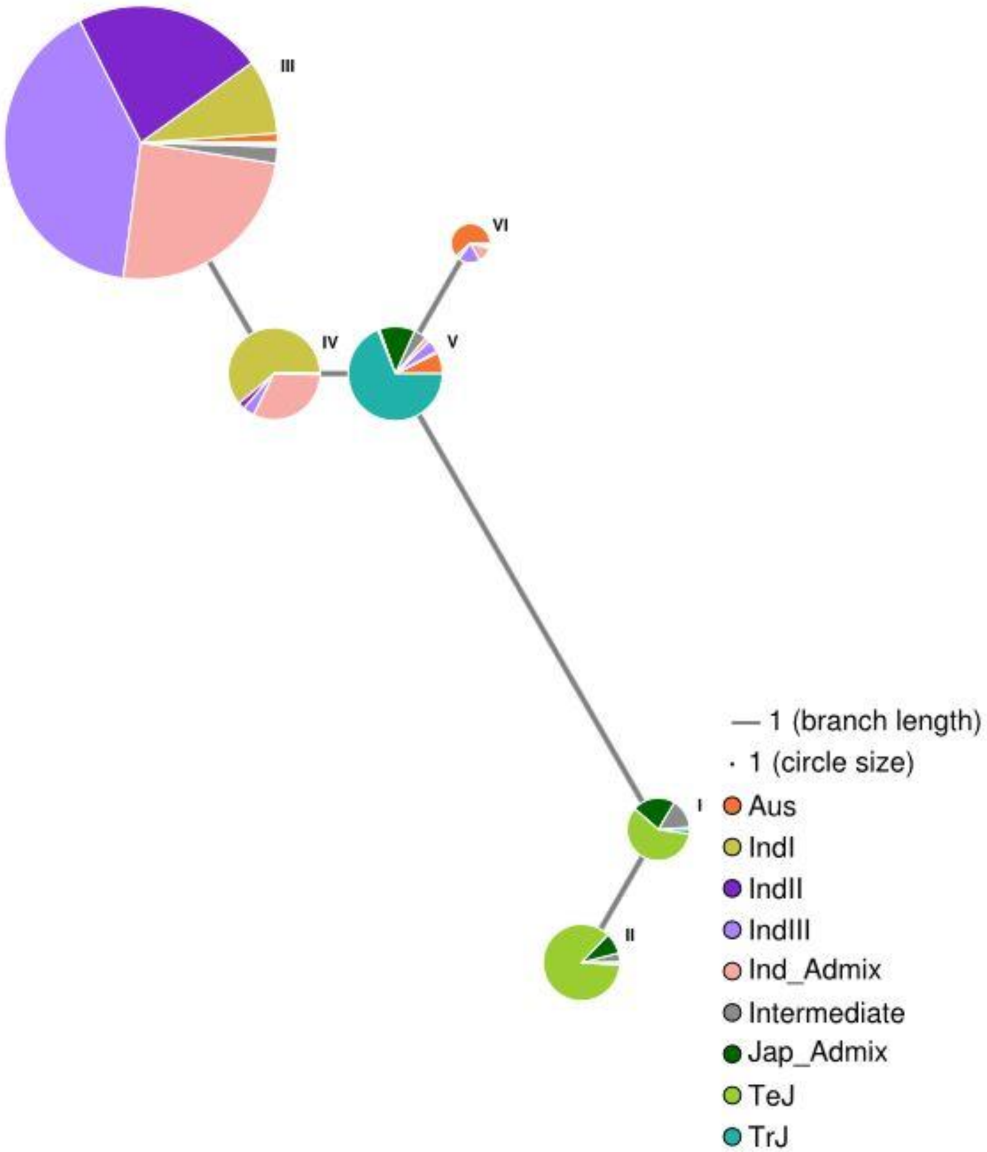

Os03g0307200

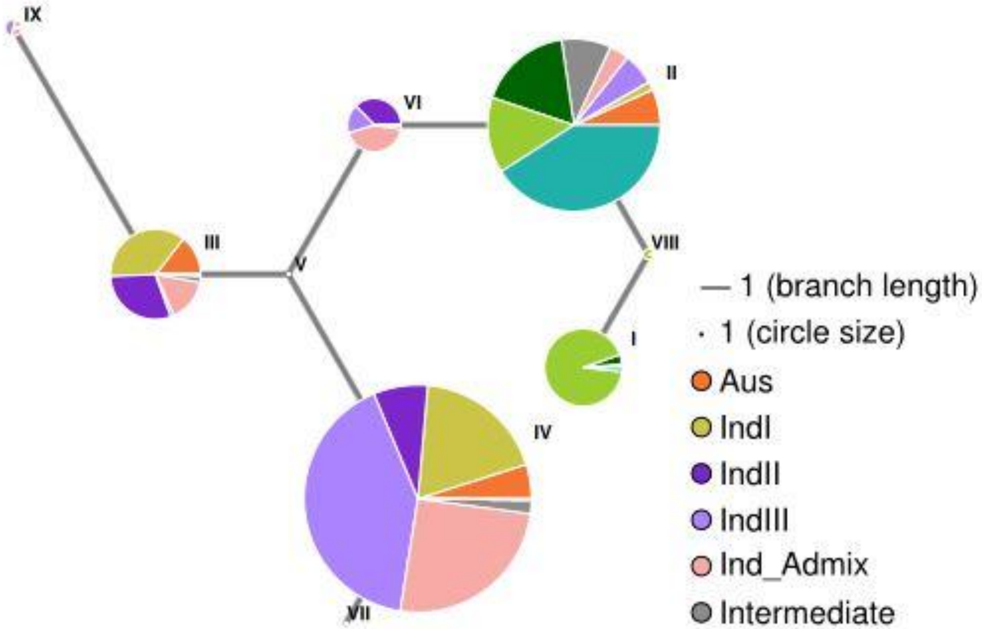

Os03g0369100

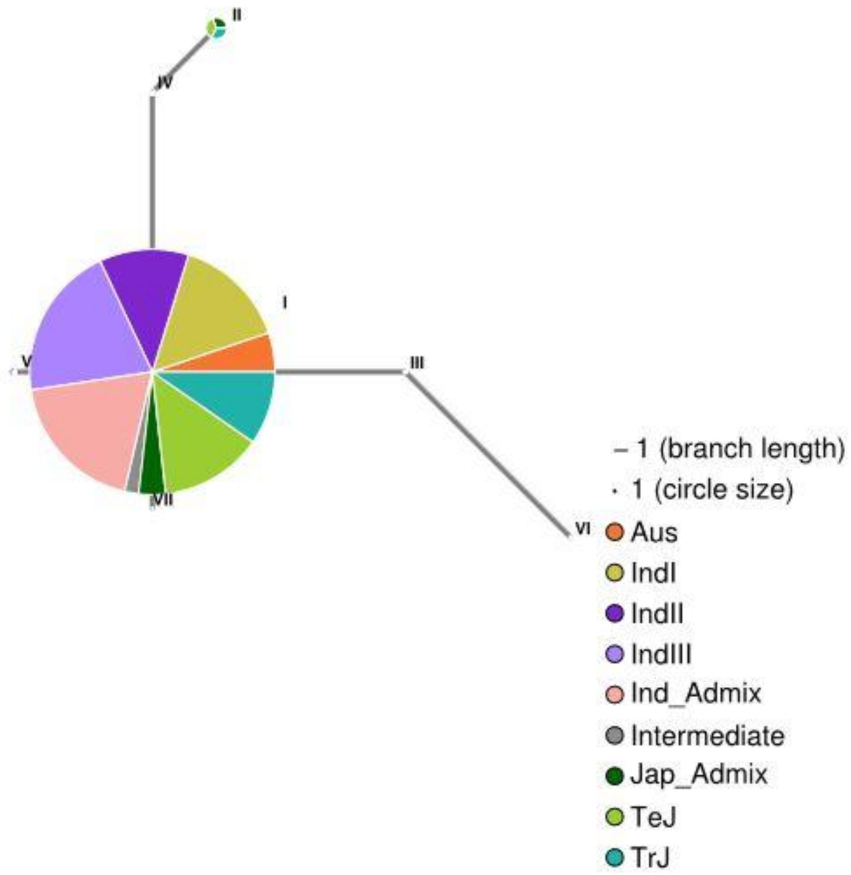

Os03g0656500

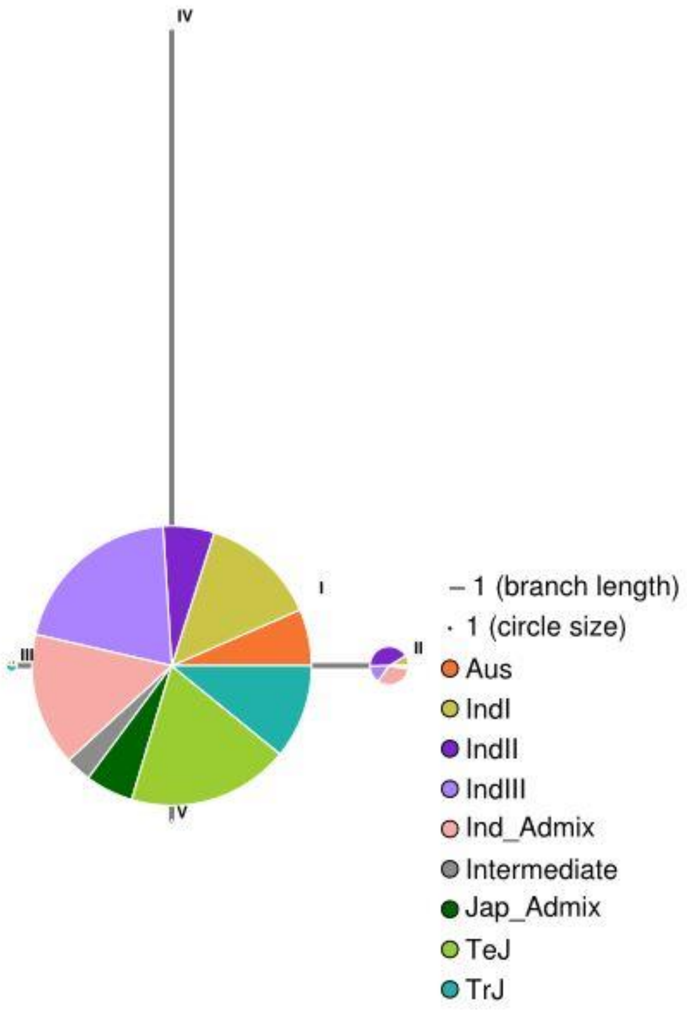

Os03g0667500

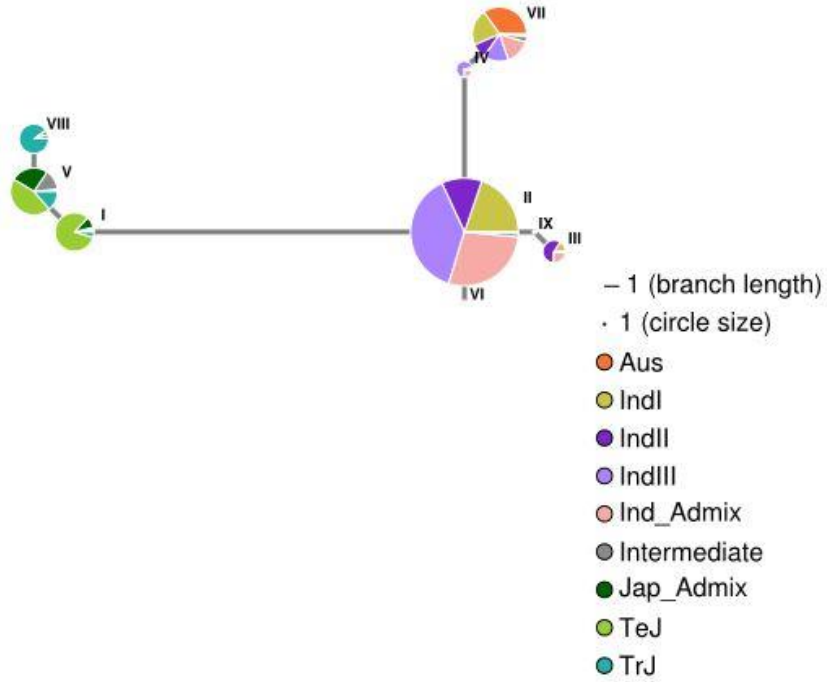

Os03g0737701

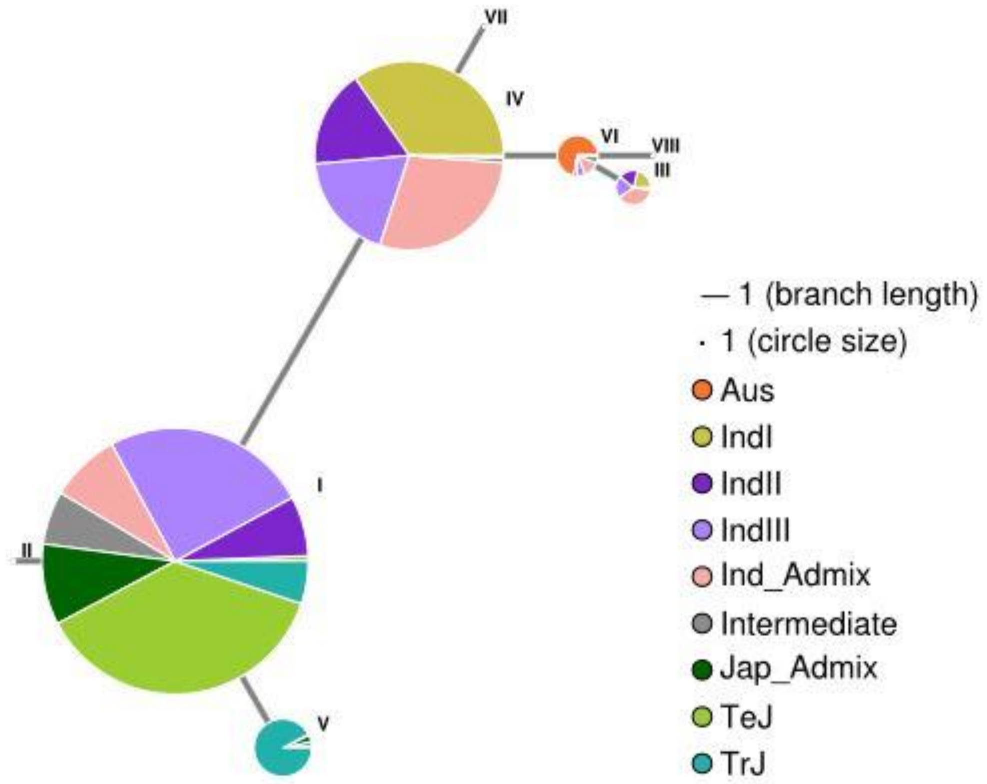

Os03g0753100

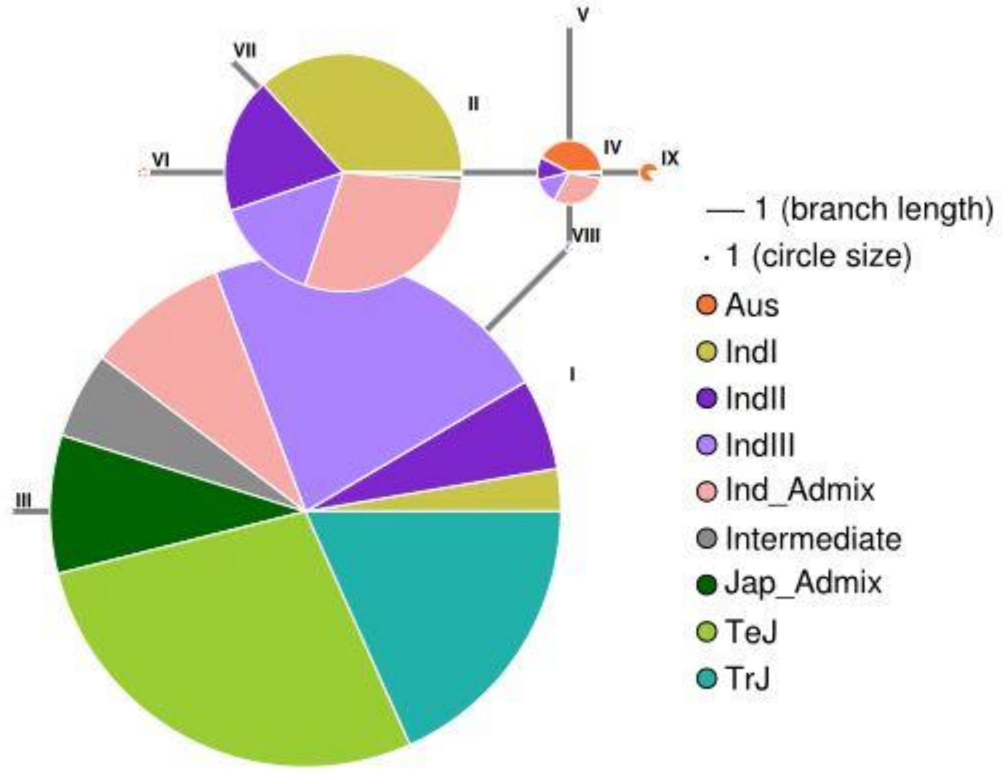

Os03g0758100

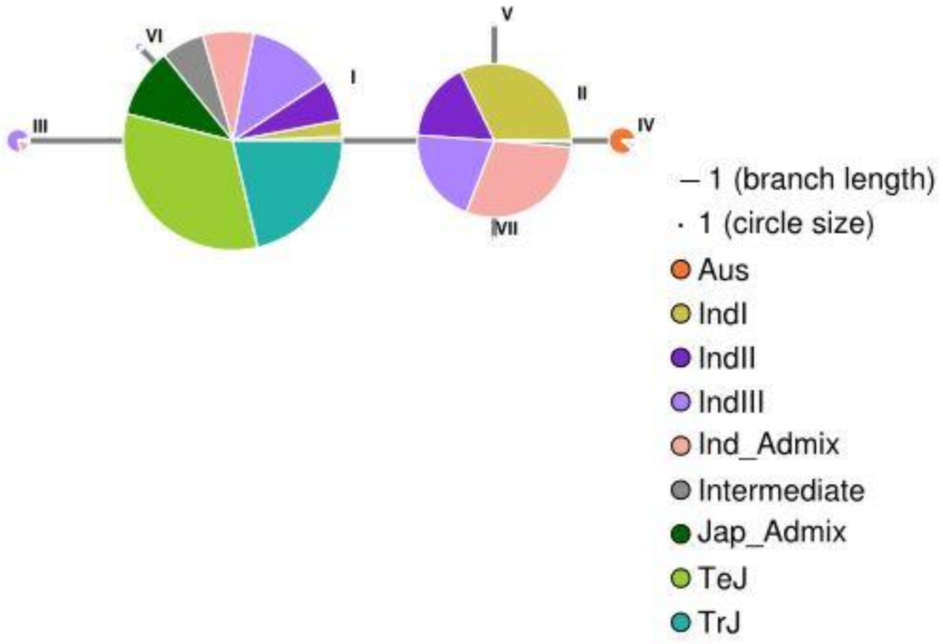

Os03g0766000

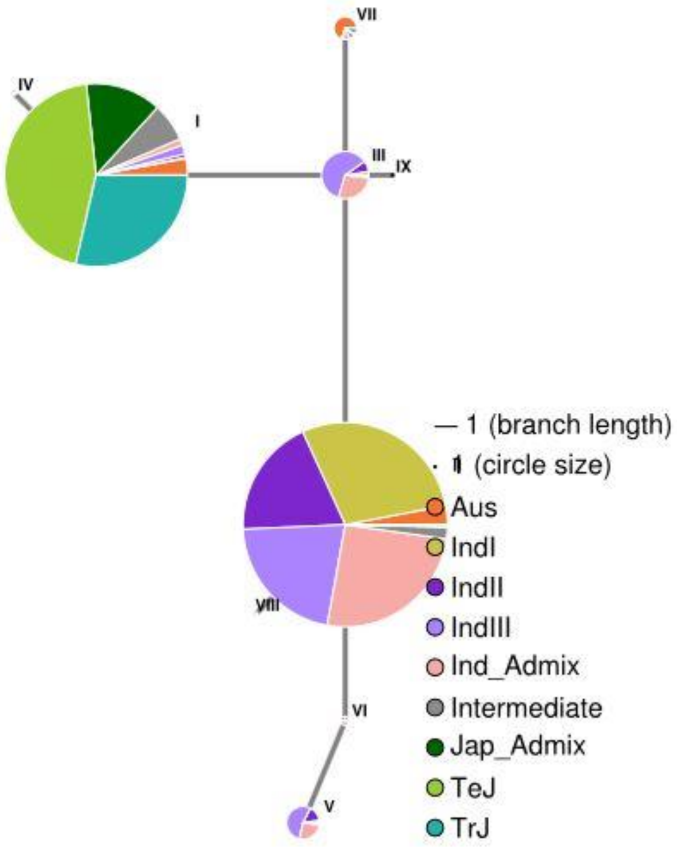

Os03g0826500

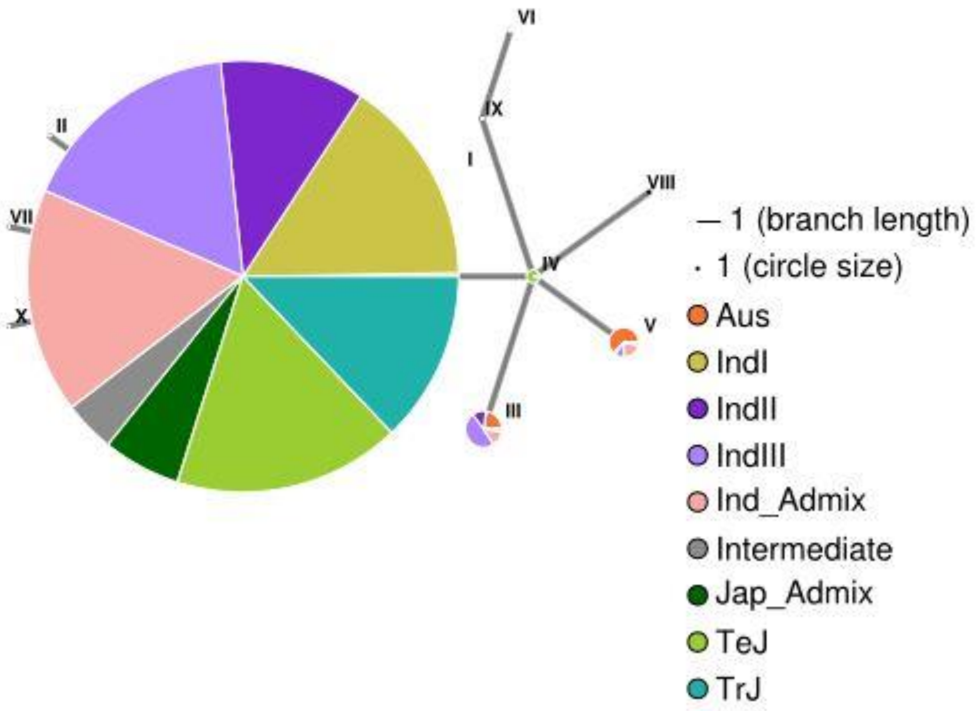

Os04g0463400

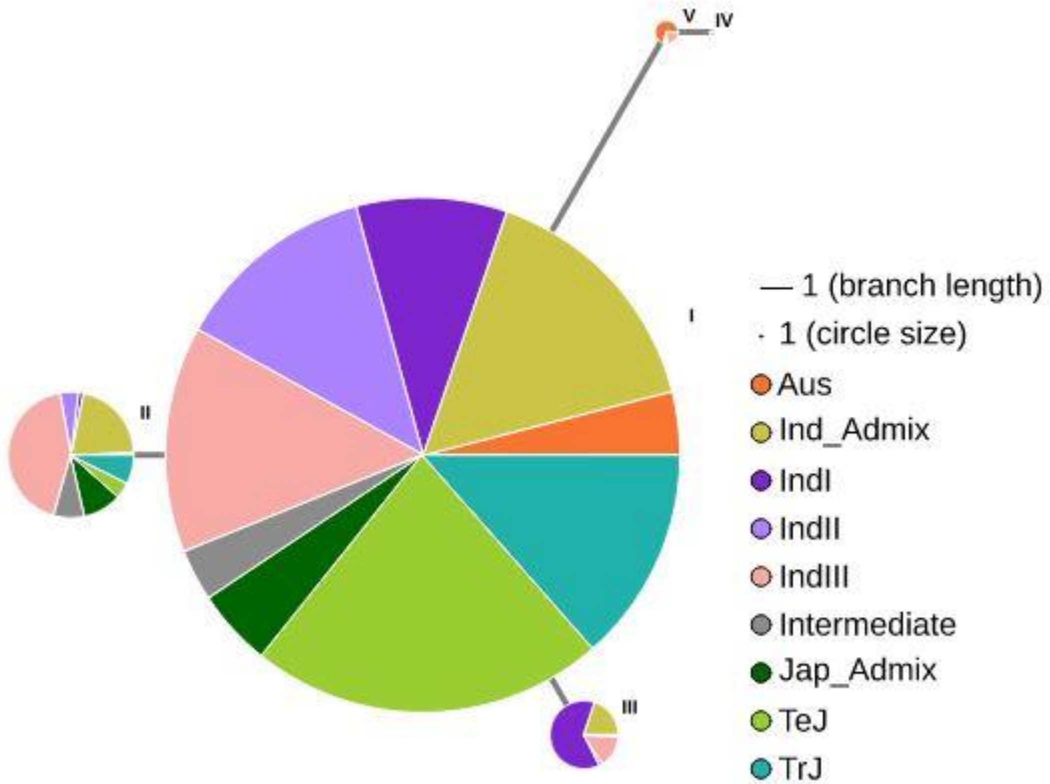

Os04g0537900

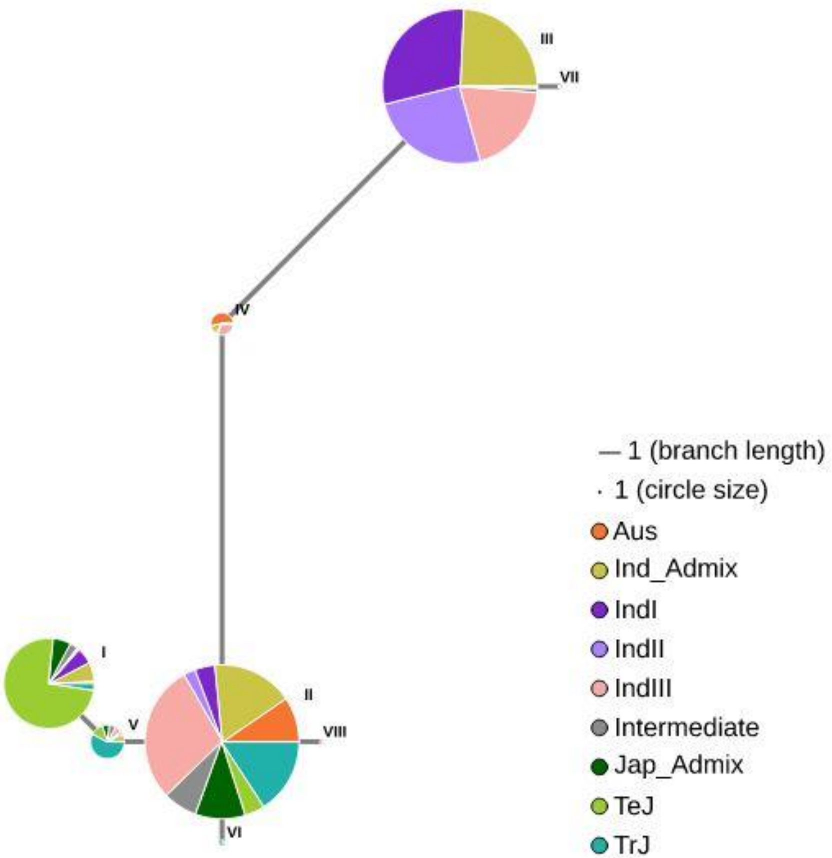

Os04g0542200

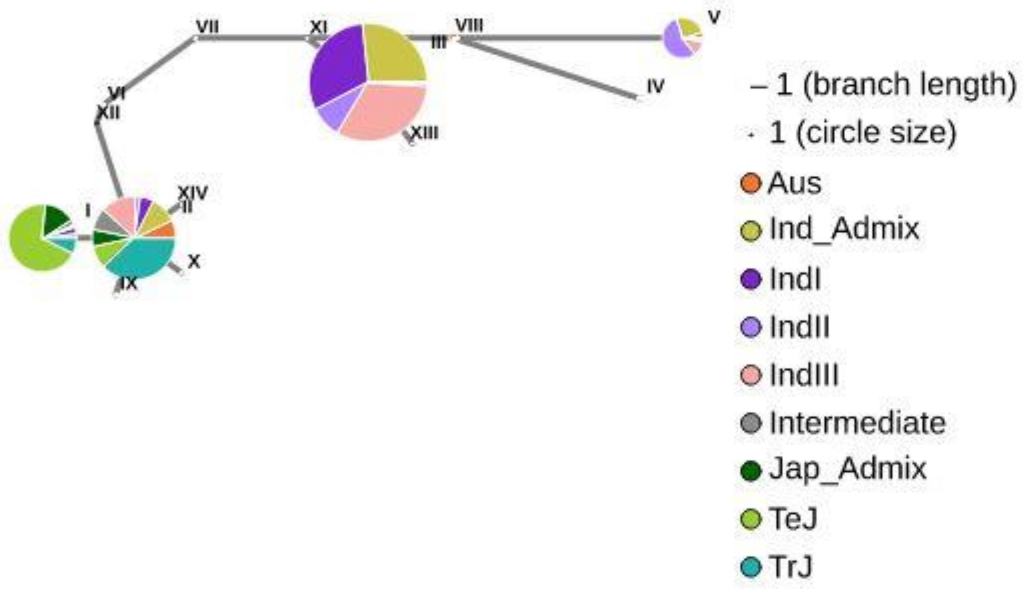

Os04g0557500

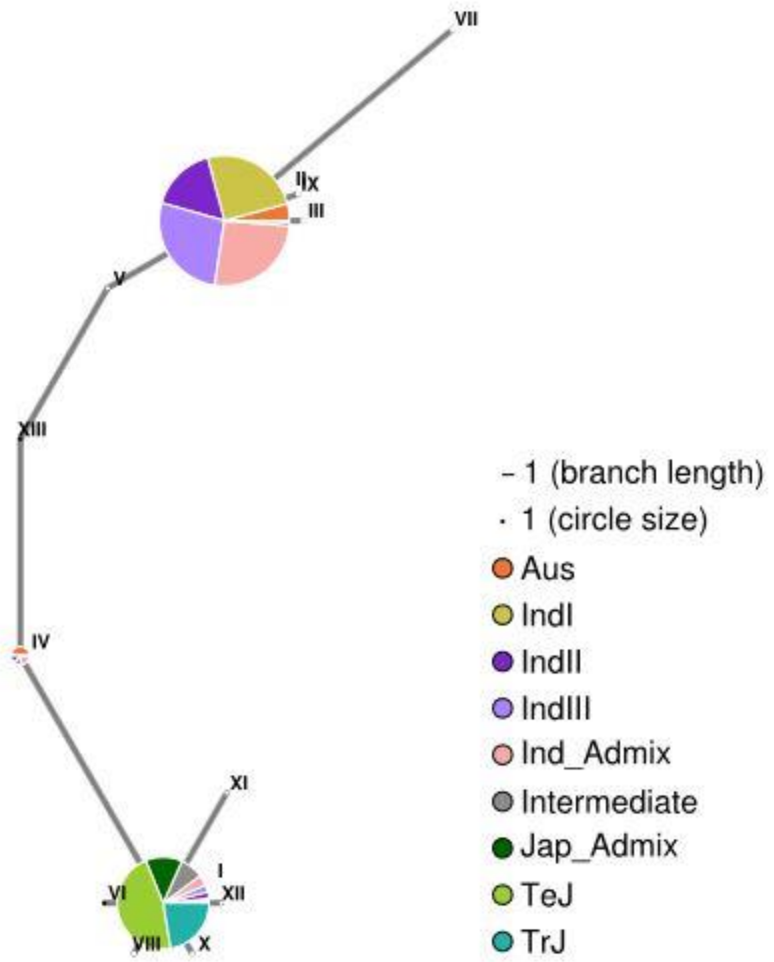

Os04g0597800

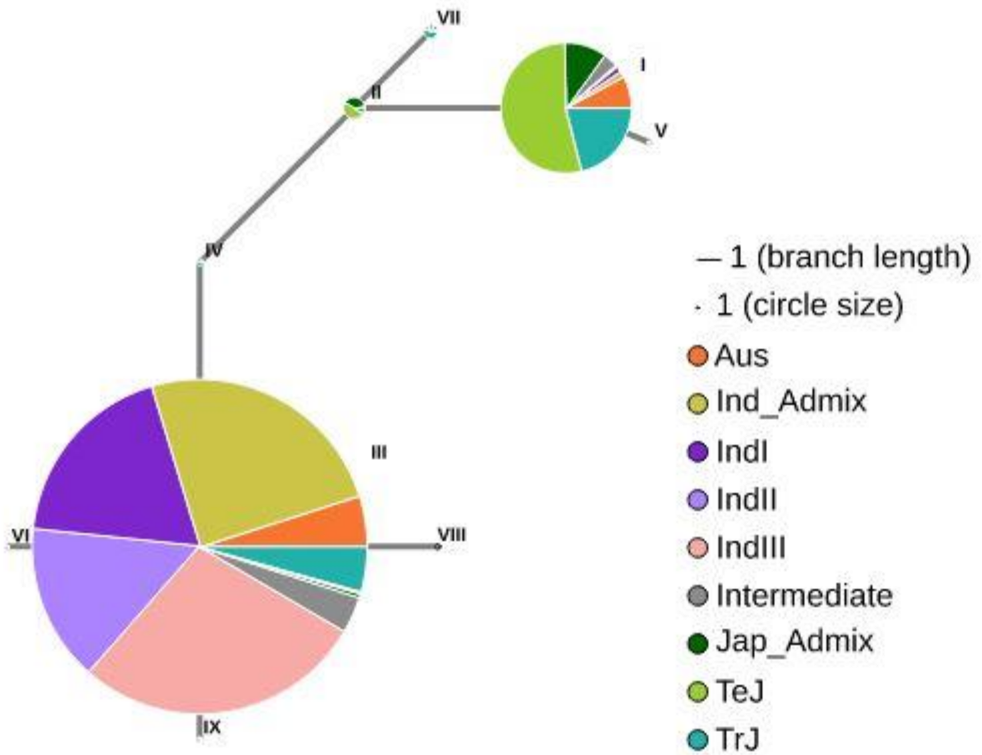

Os04g0645100

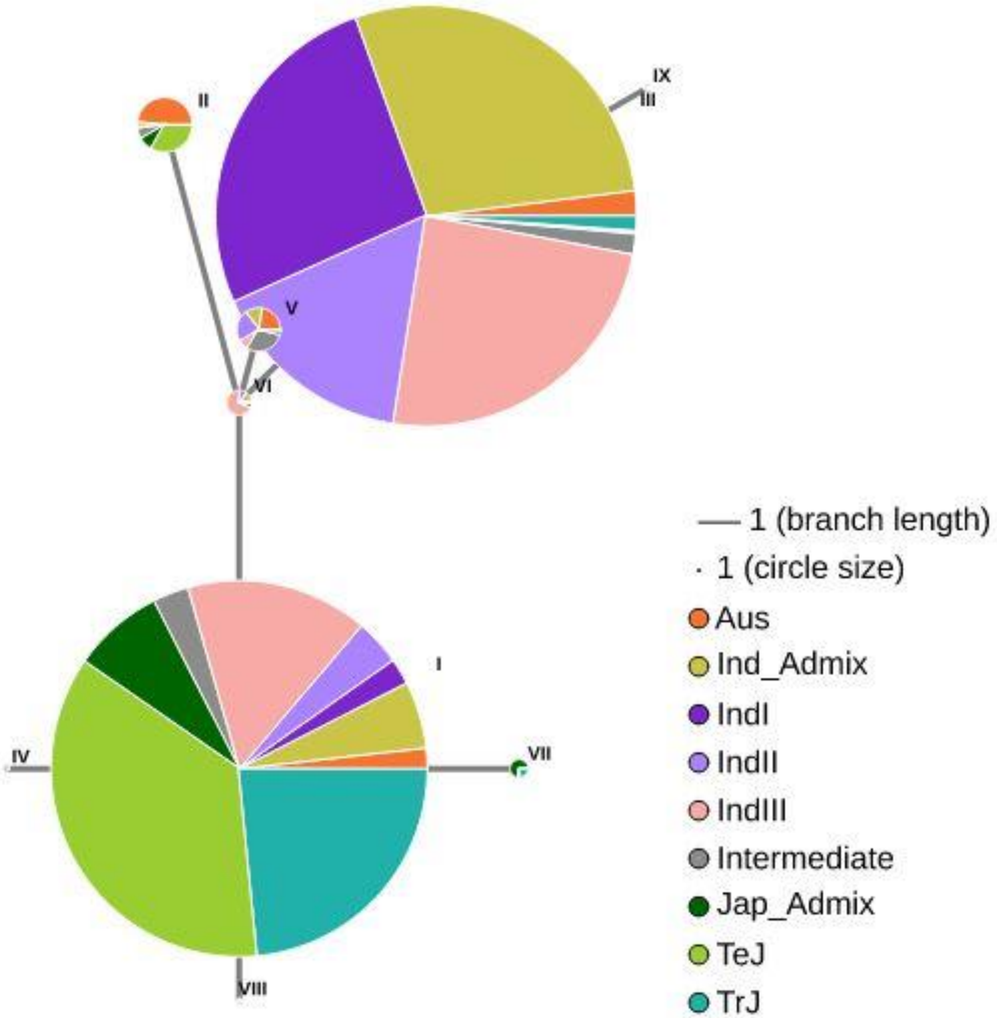

Os04g0652400

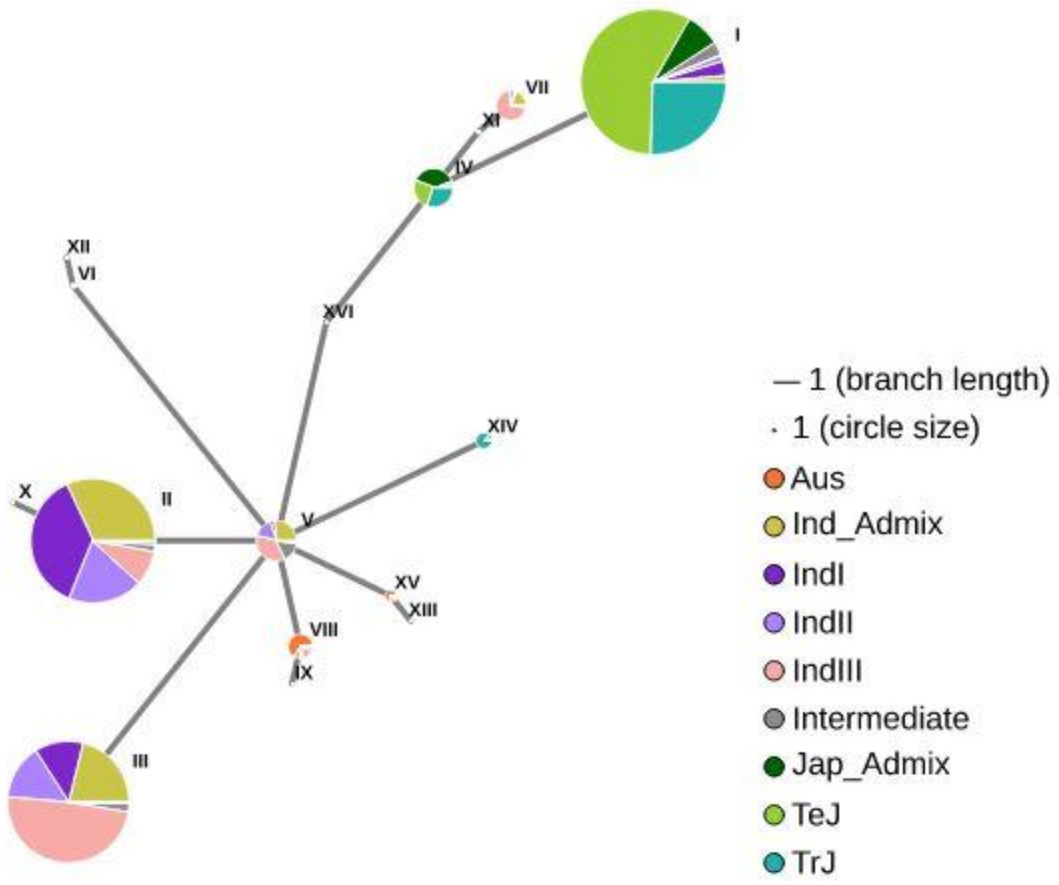

Os05g0115800

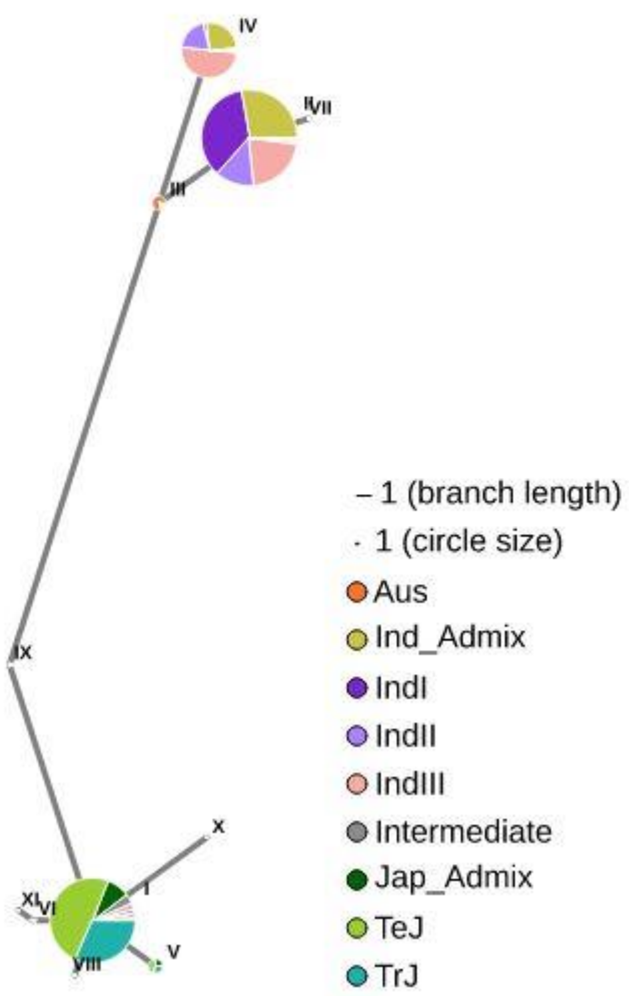

Os05g0156900

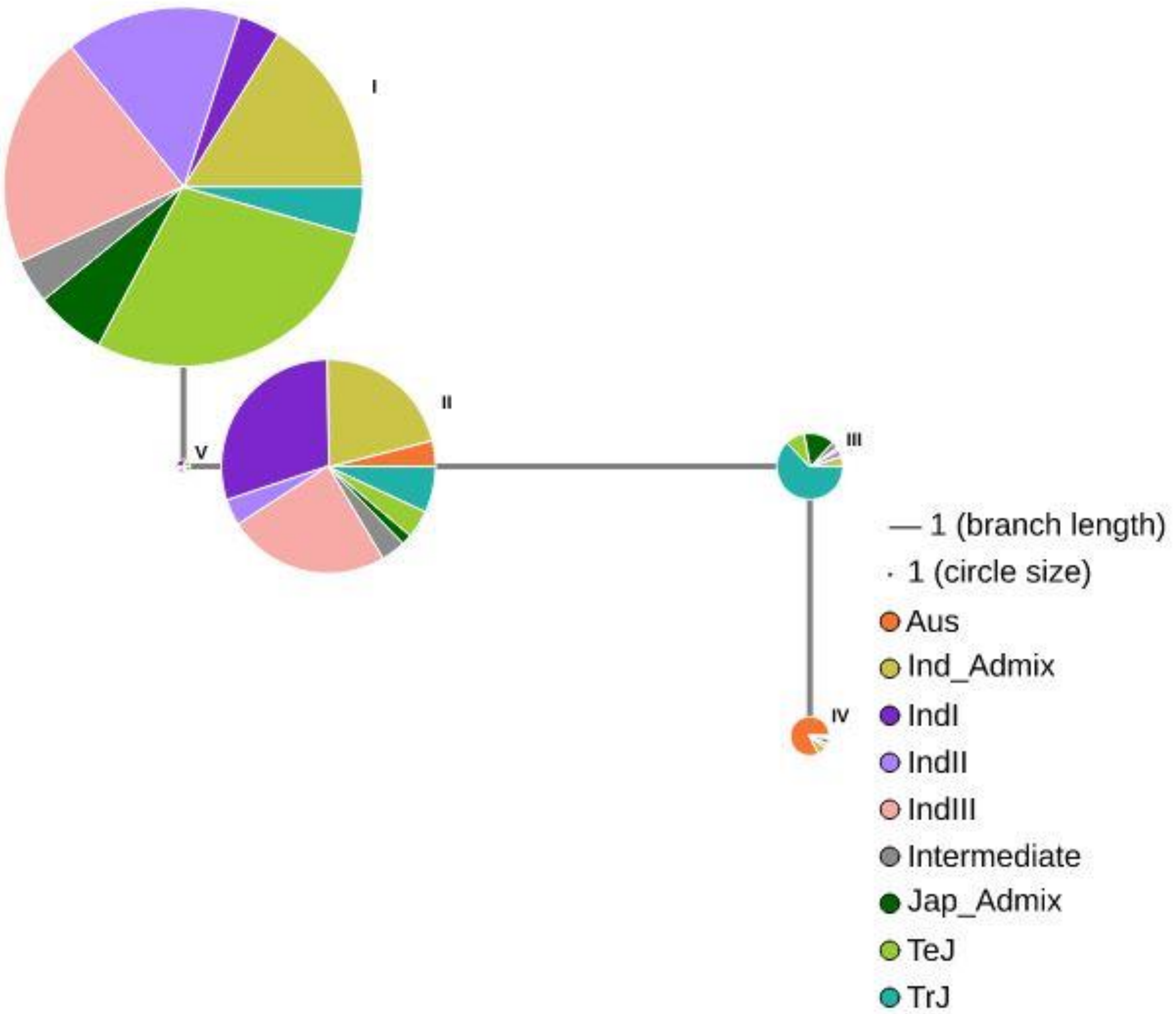

Os05g0187500

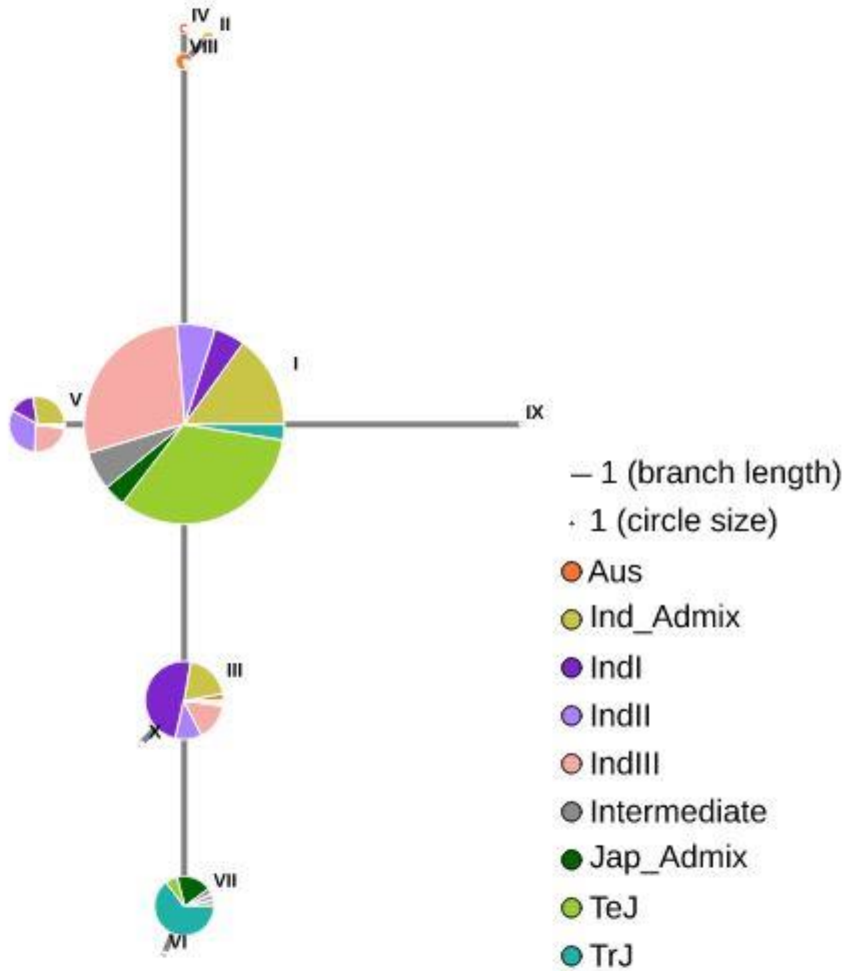

Os05g0198400

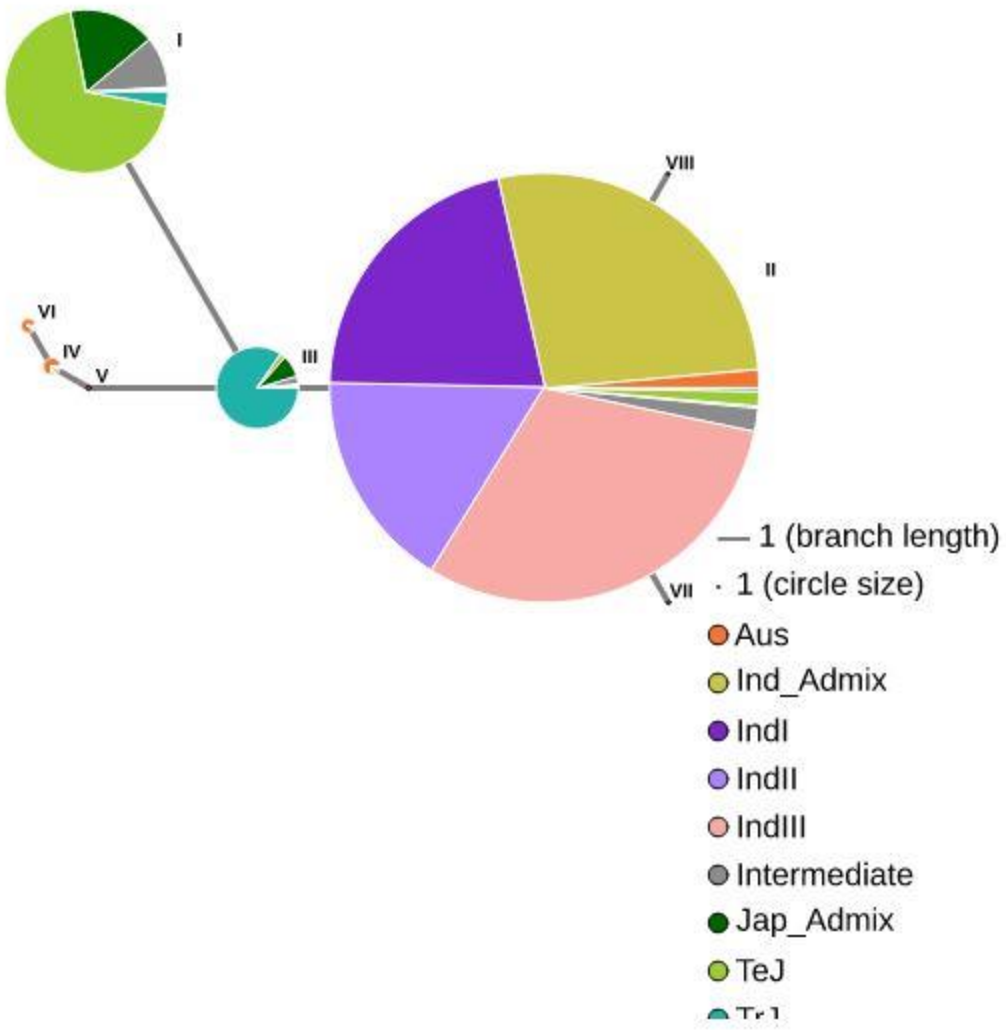

Os05g0408200

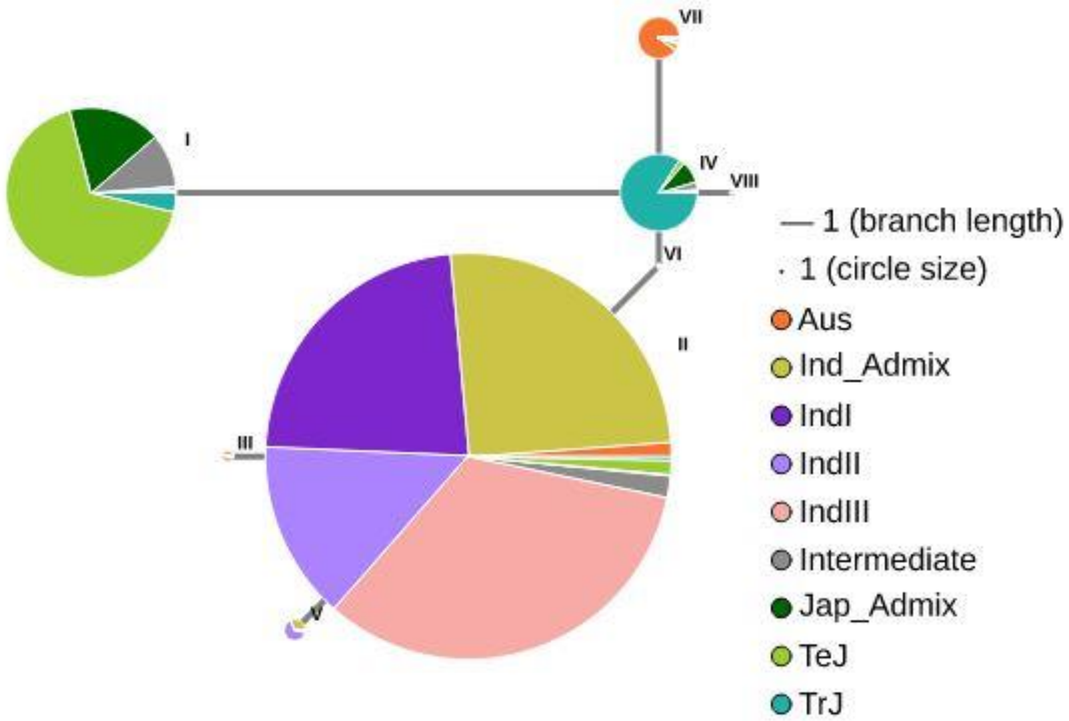

Os05g0415200

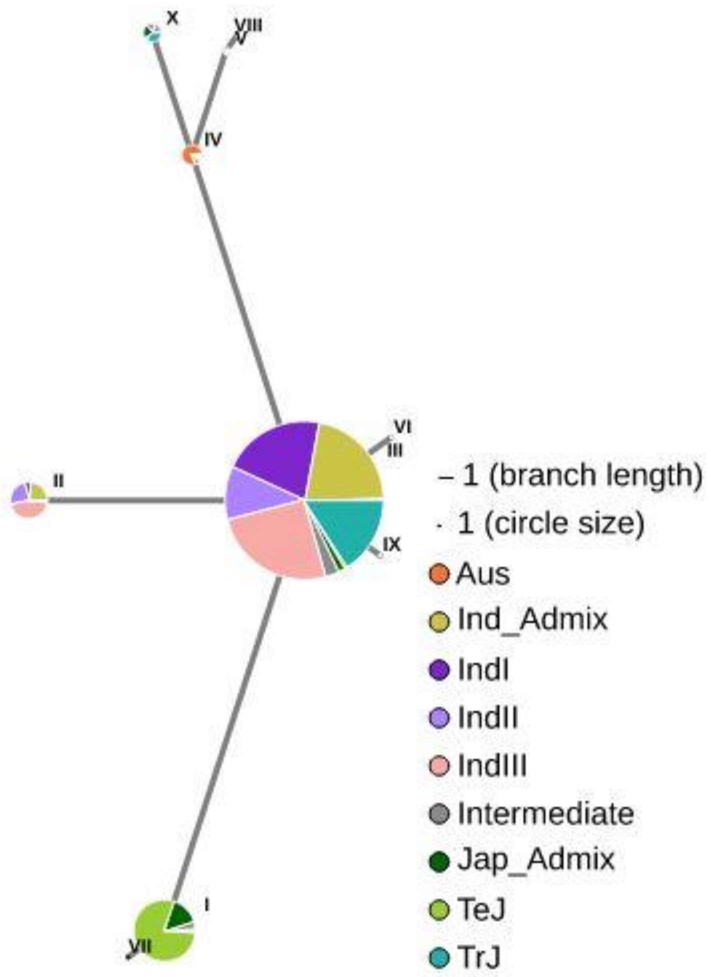

Os05g0554000

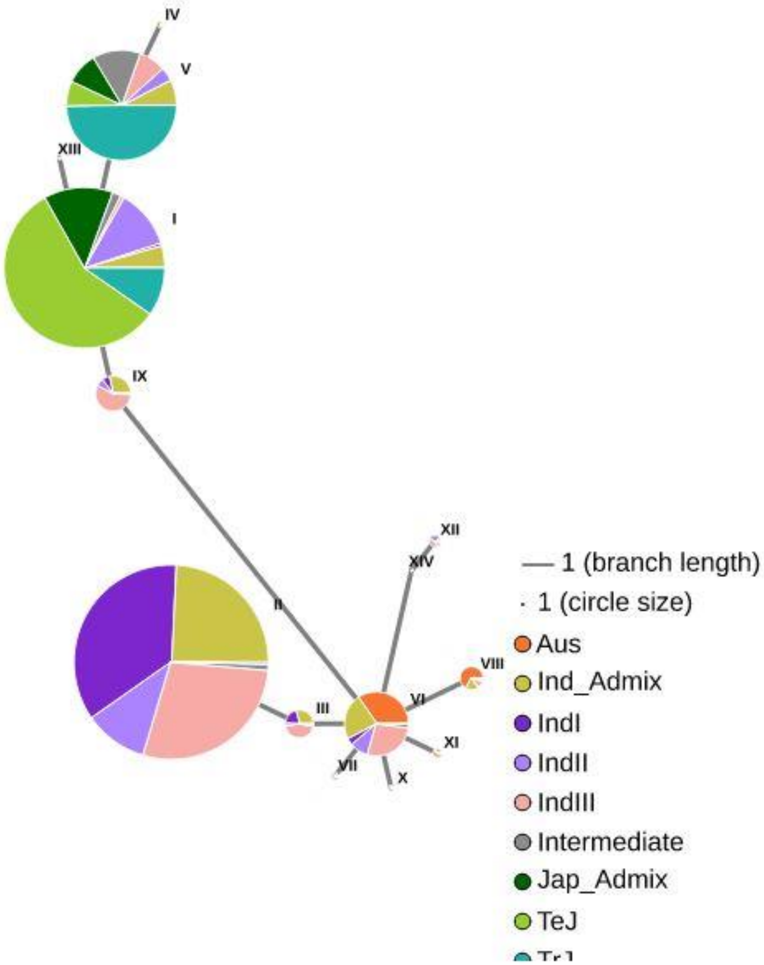

Os06g0133000

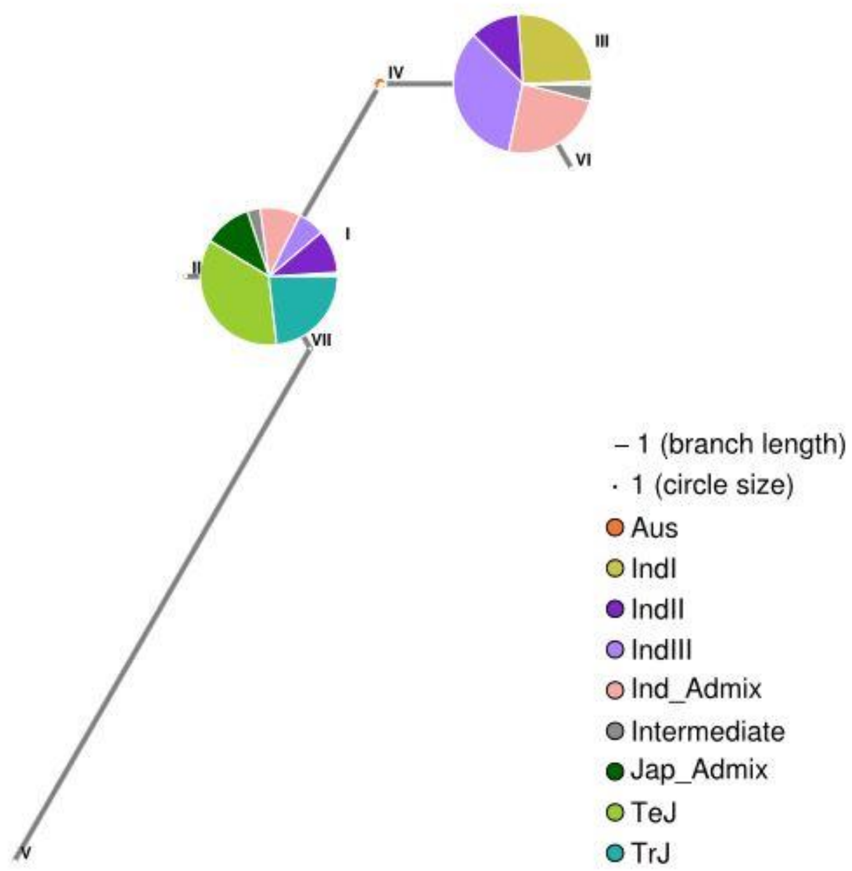

Os06g0143700

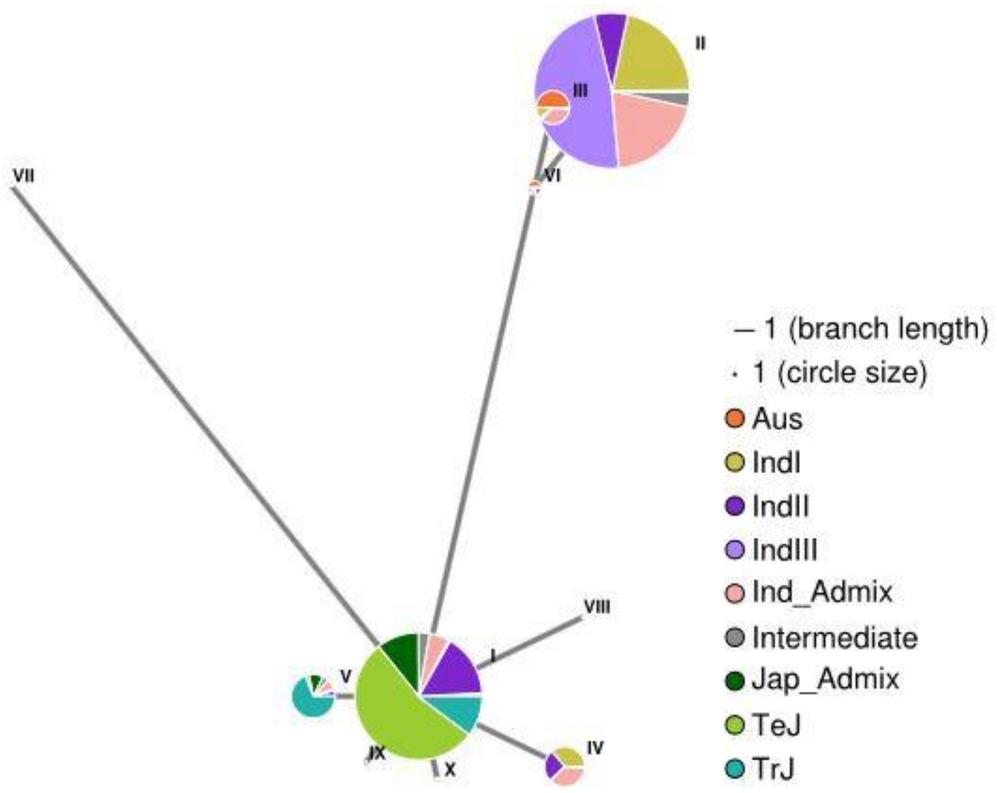

Os06g0160700

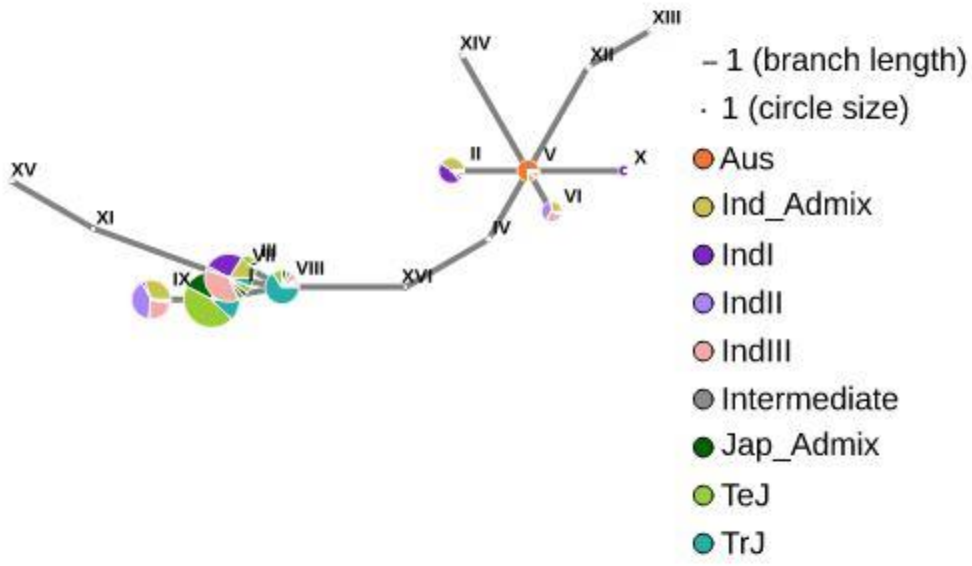

Os06g0229800

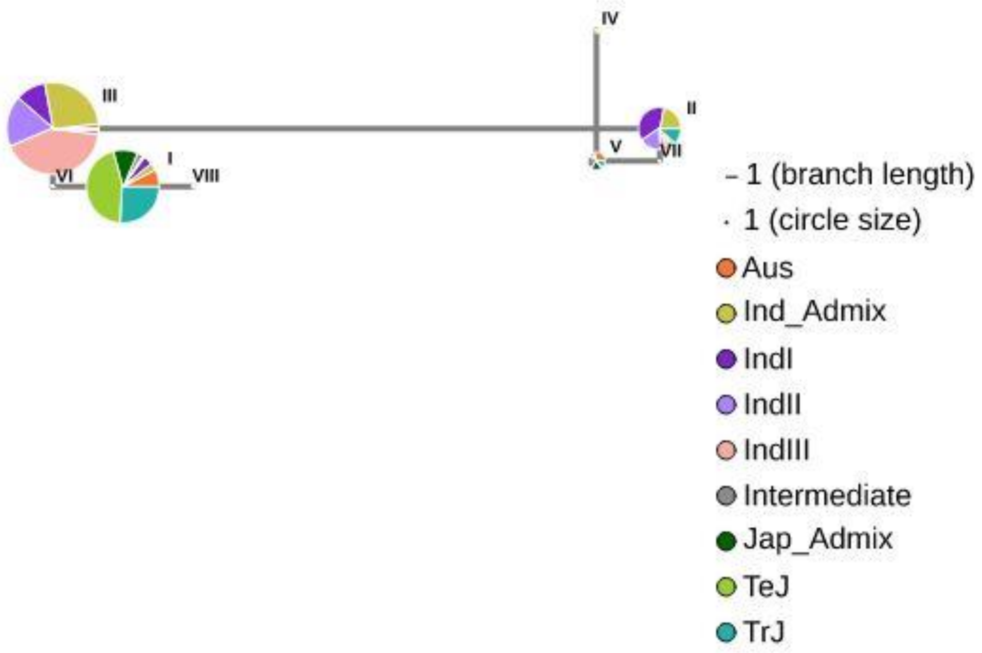

Os06g0700700

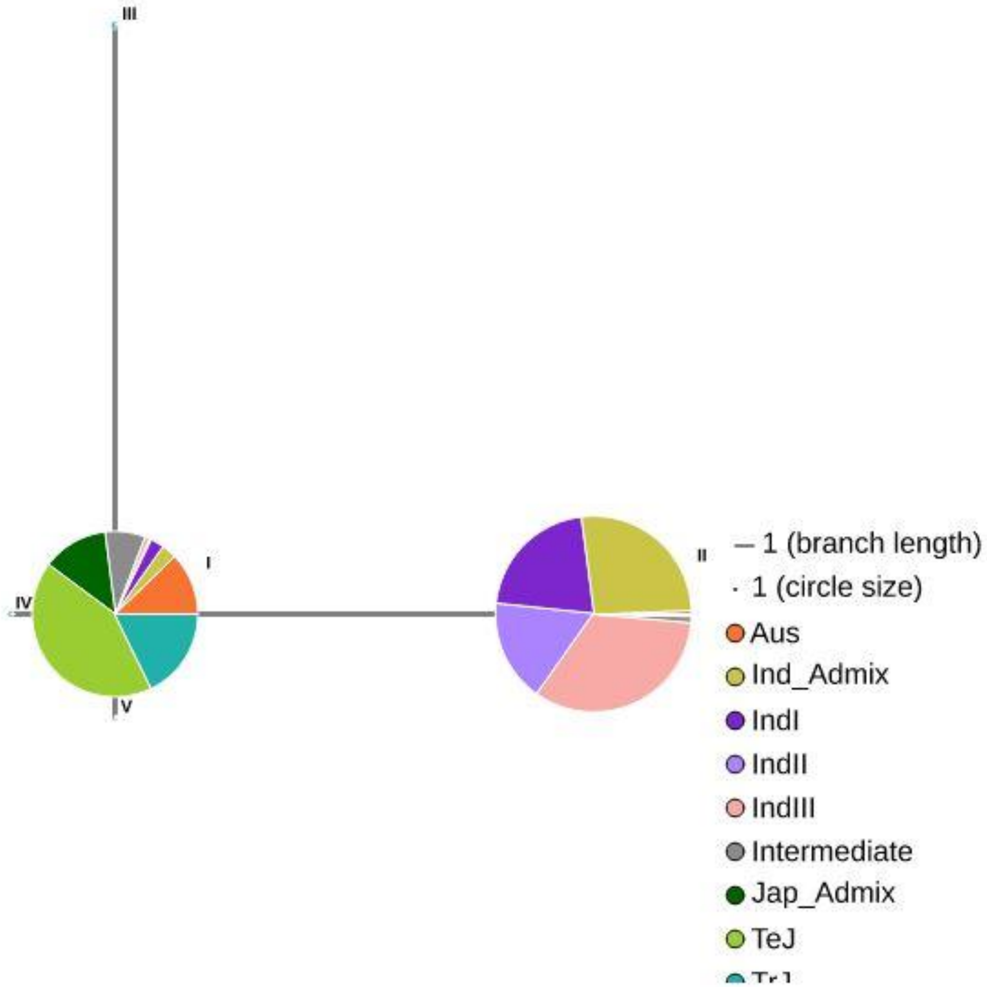

Os07g0181000

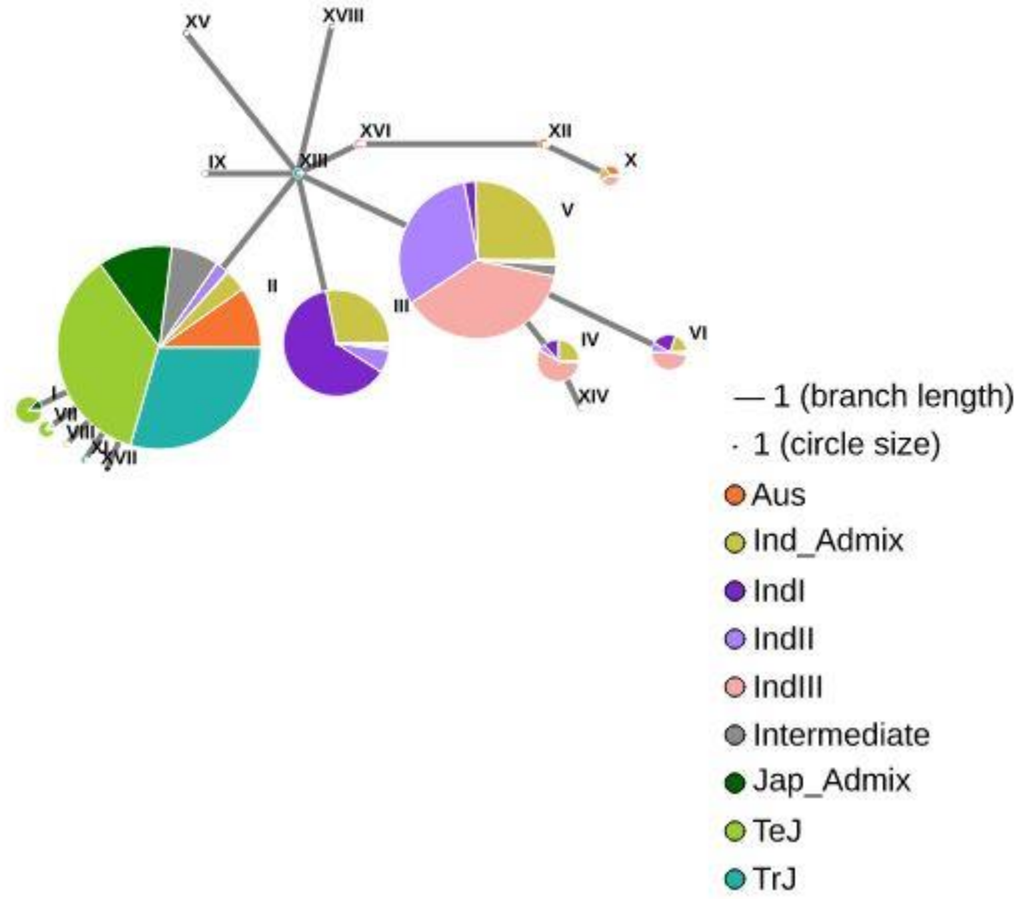

Os07g0257200

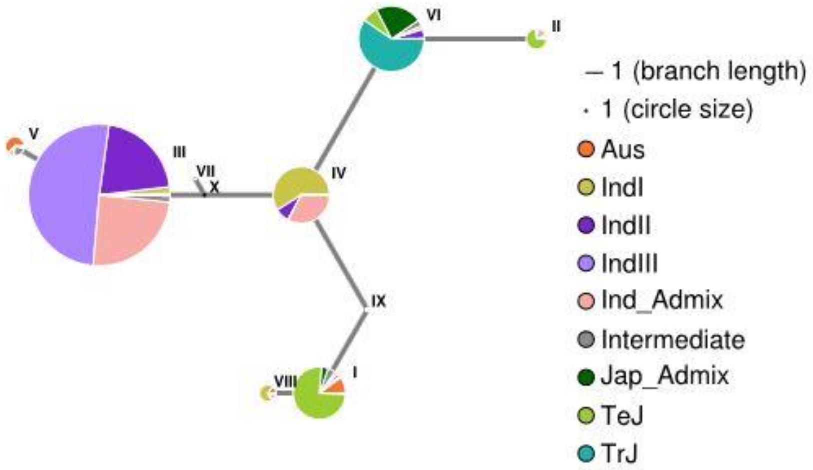

Os07g0603300

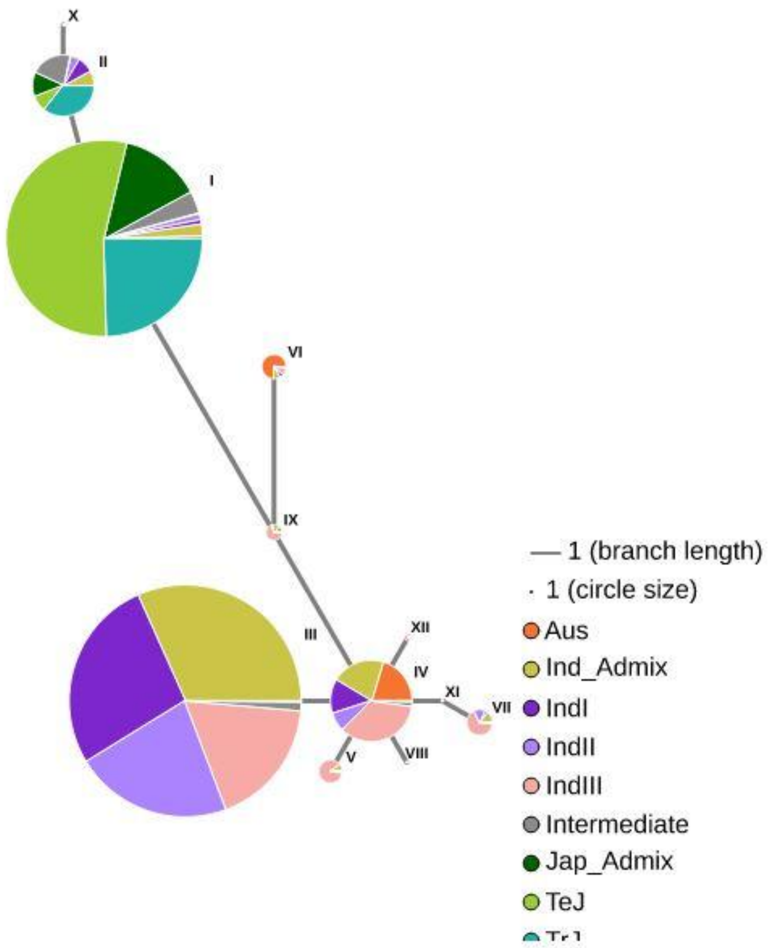

Os07g0616800

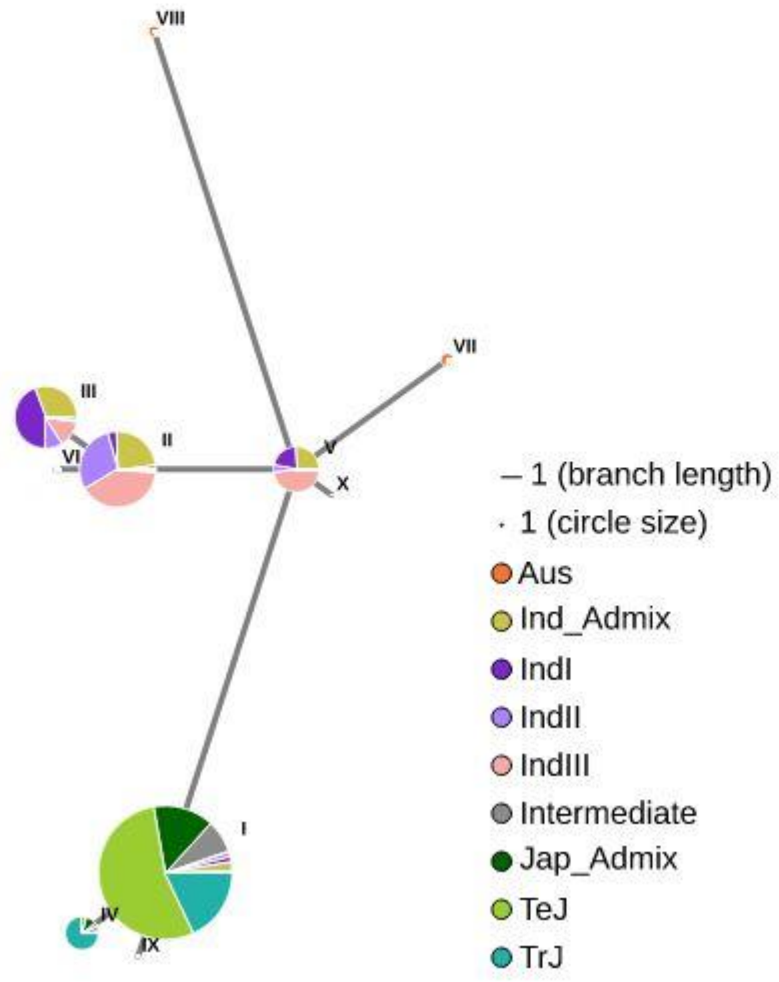

Os07g0623200

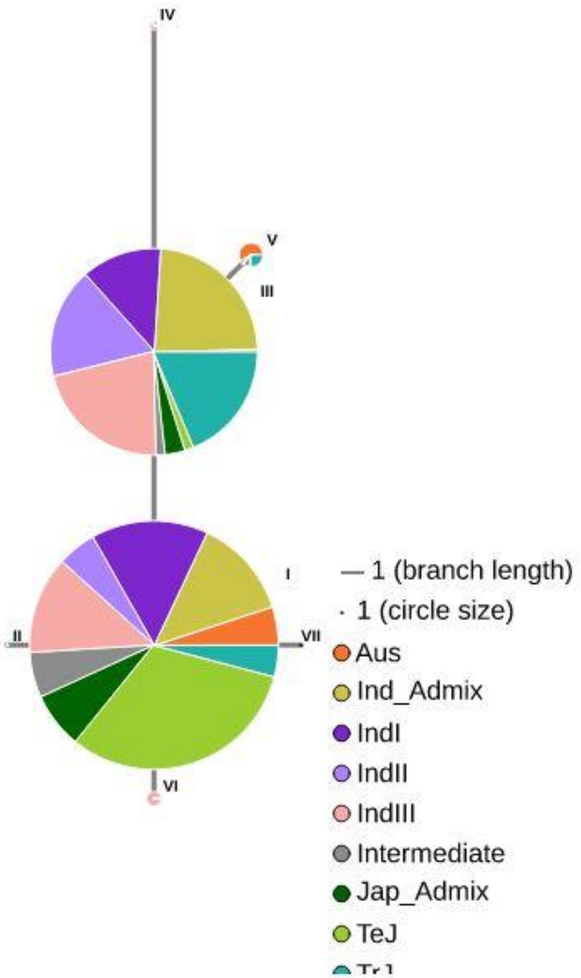

Os07g0668600

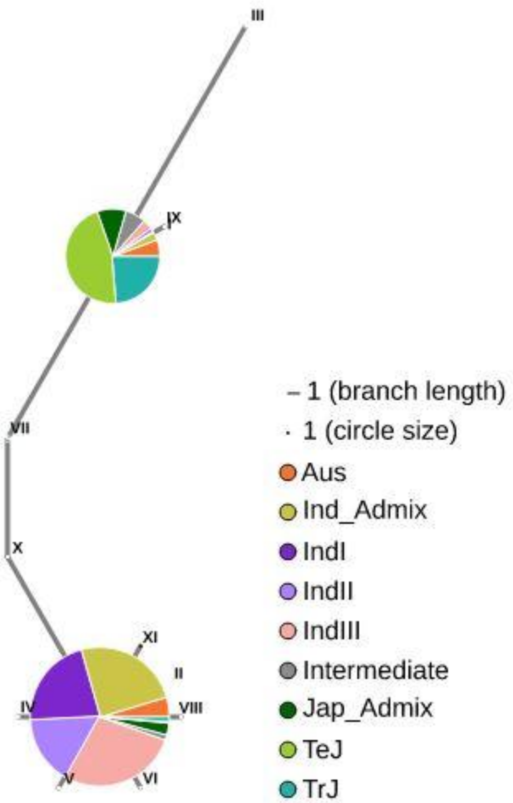

Os07g0689600

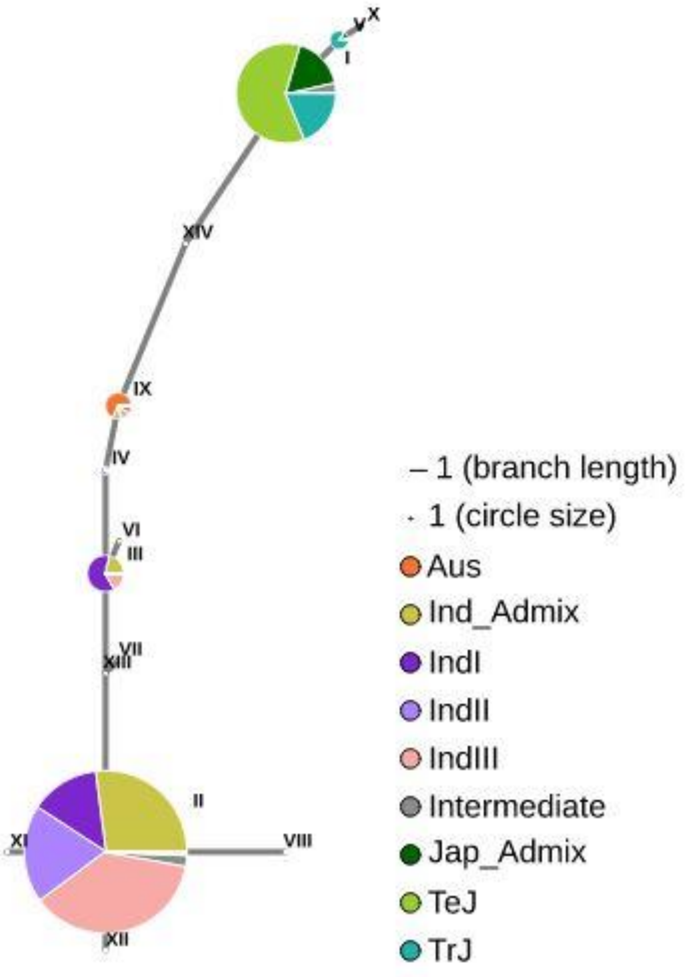

Os08g0101500

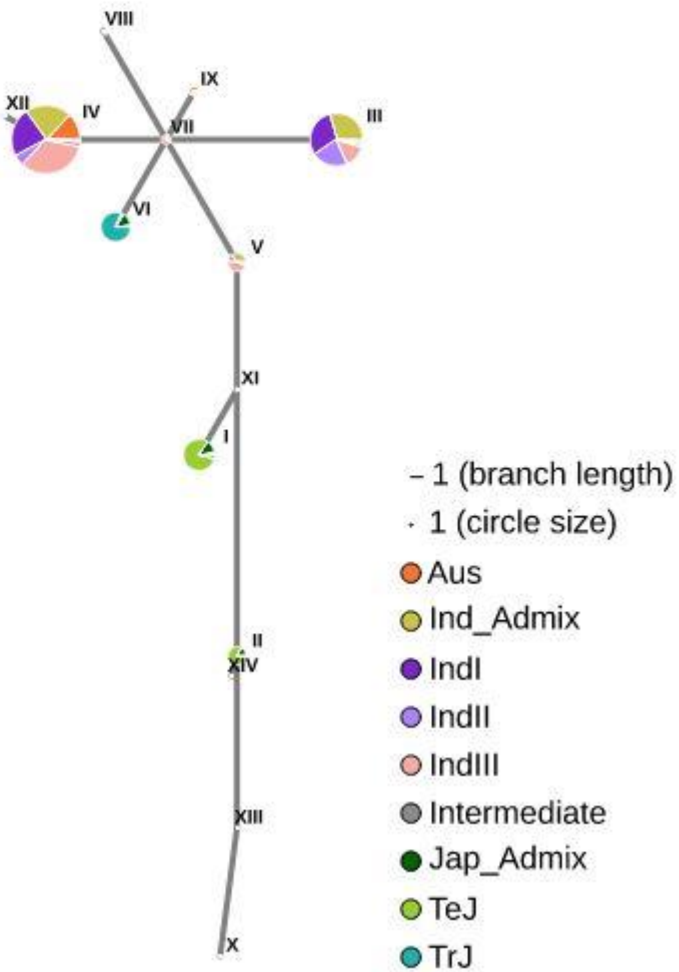

Os08g0191433

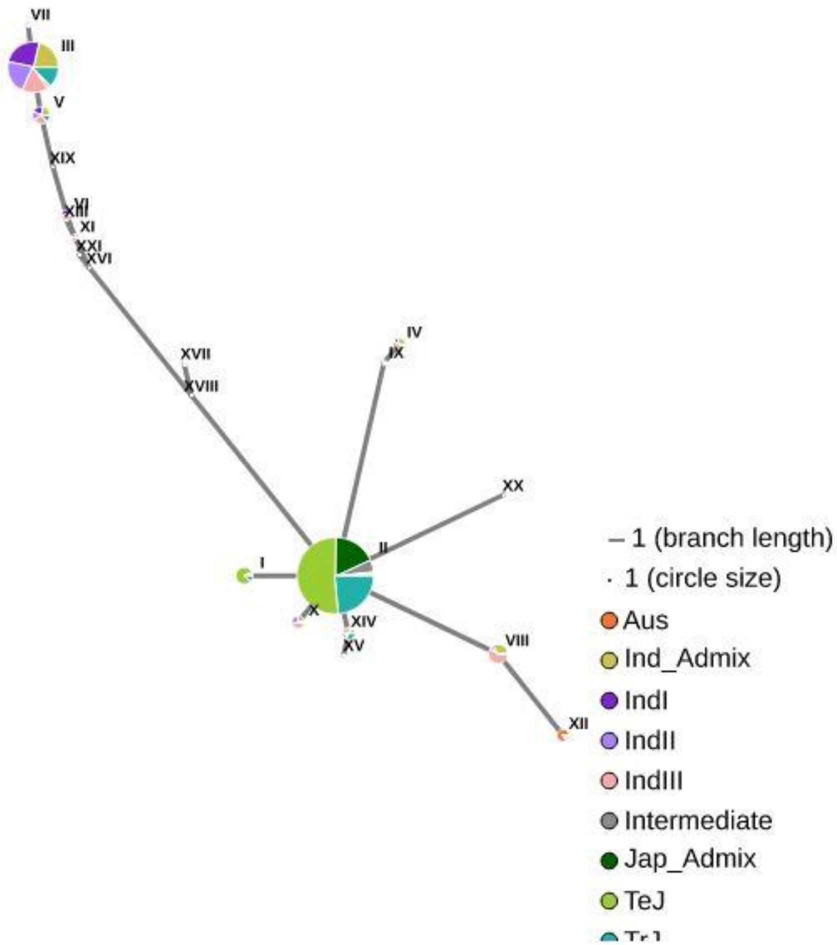

Os08g0424500

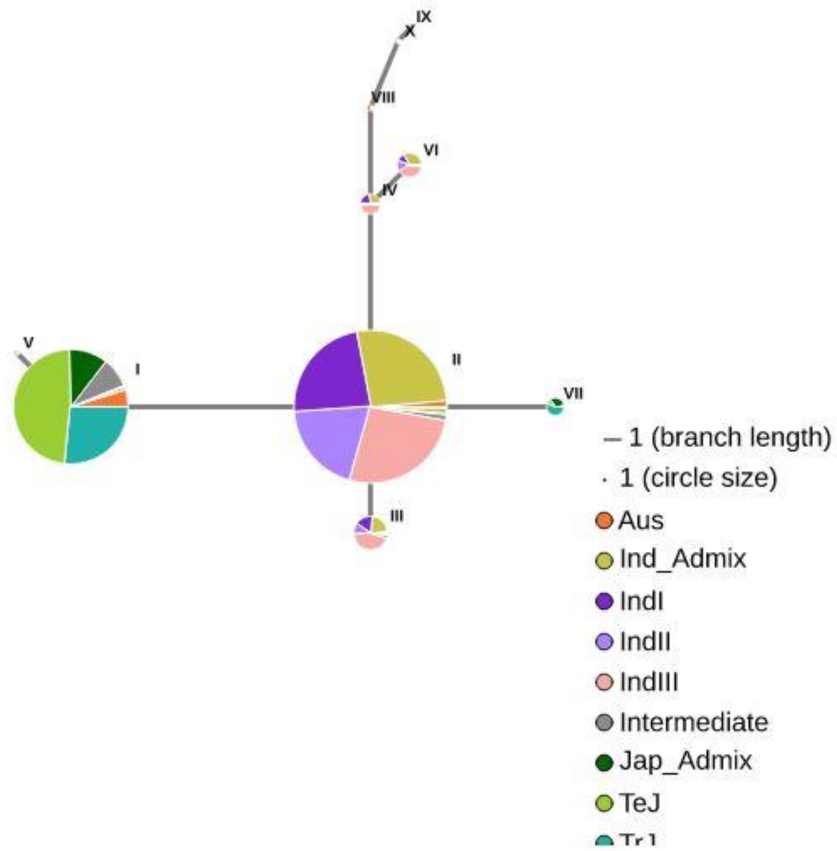

Os08g0531600

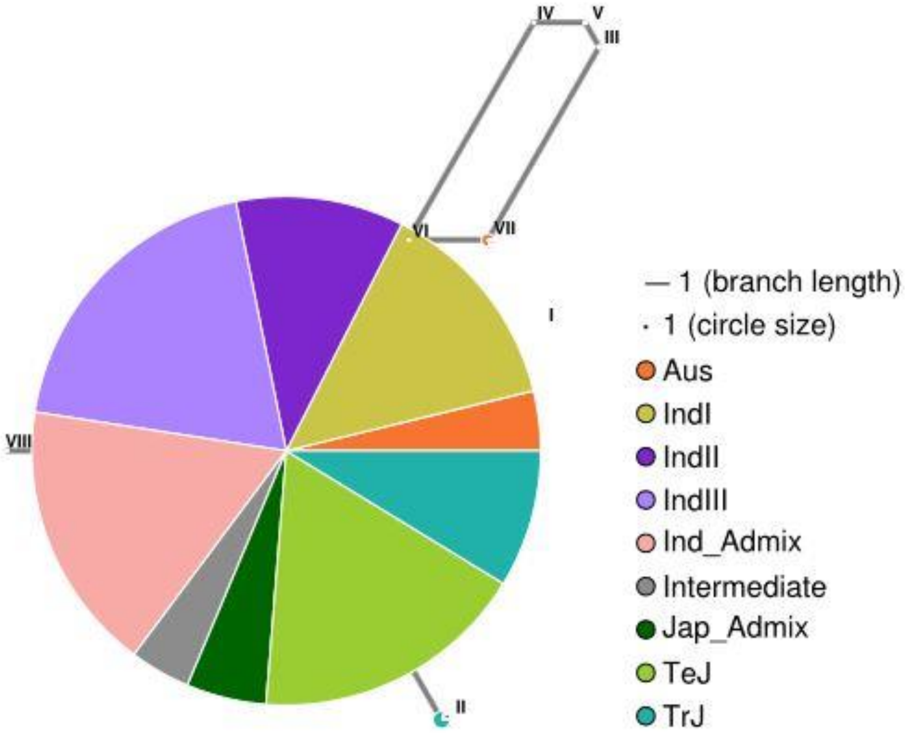

Os09g0396900

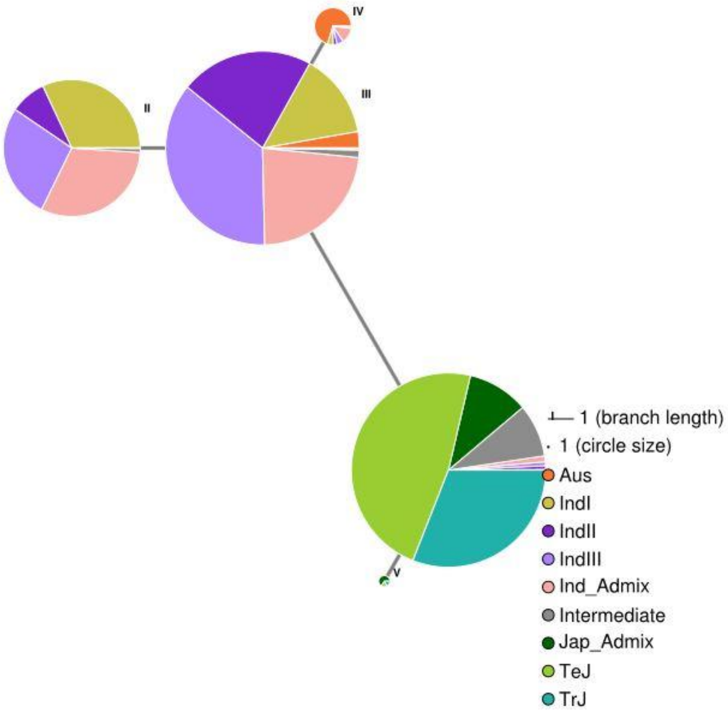

Os09g0440300

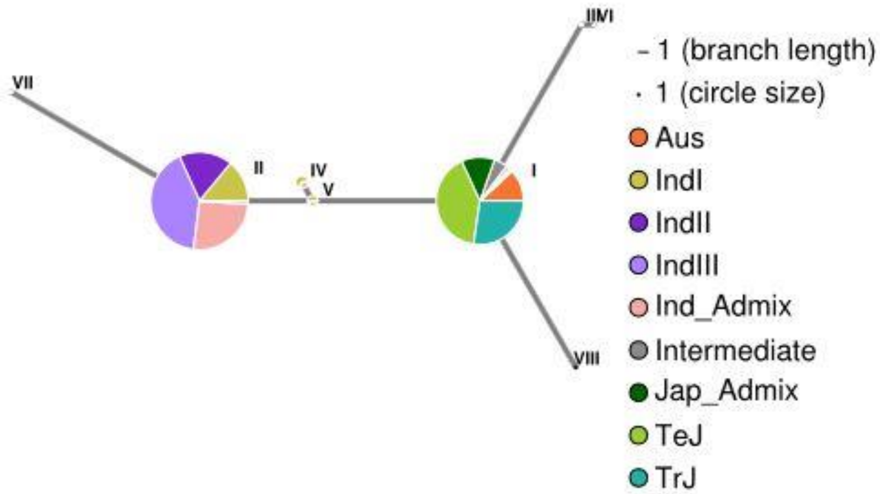

Os09g0448500

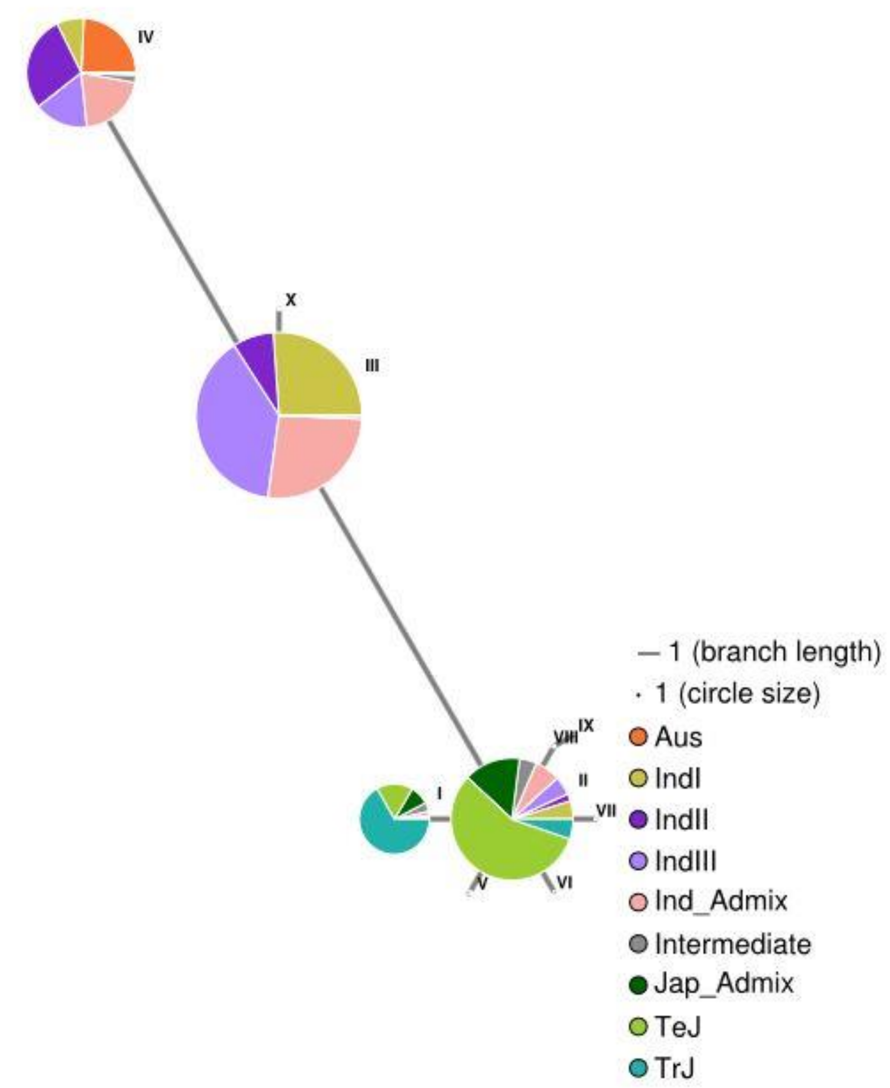

Os09g0529700

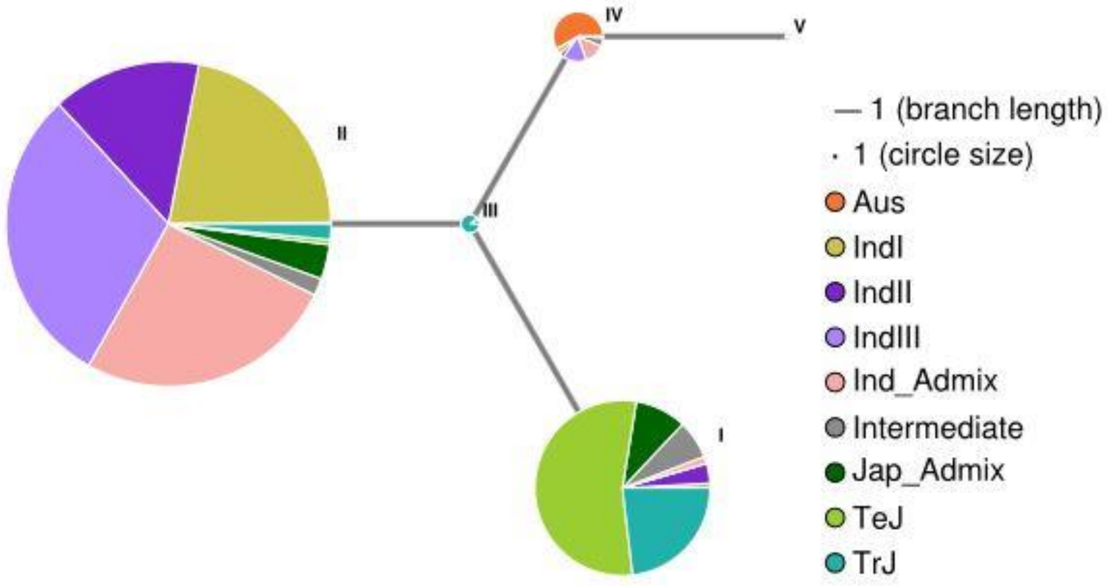

Os10g0478200

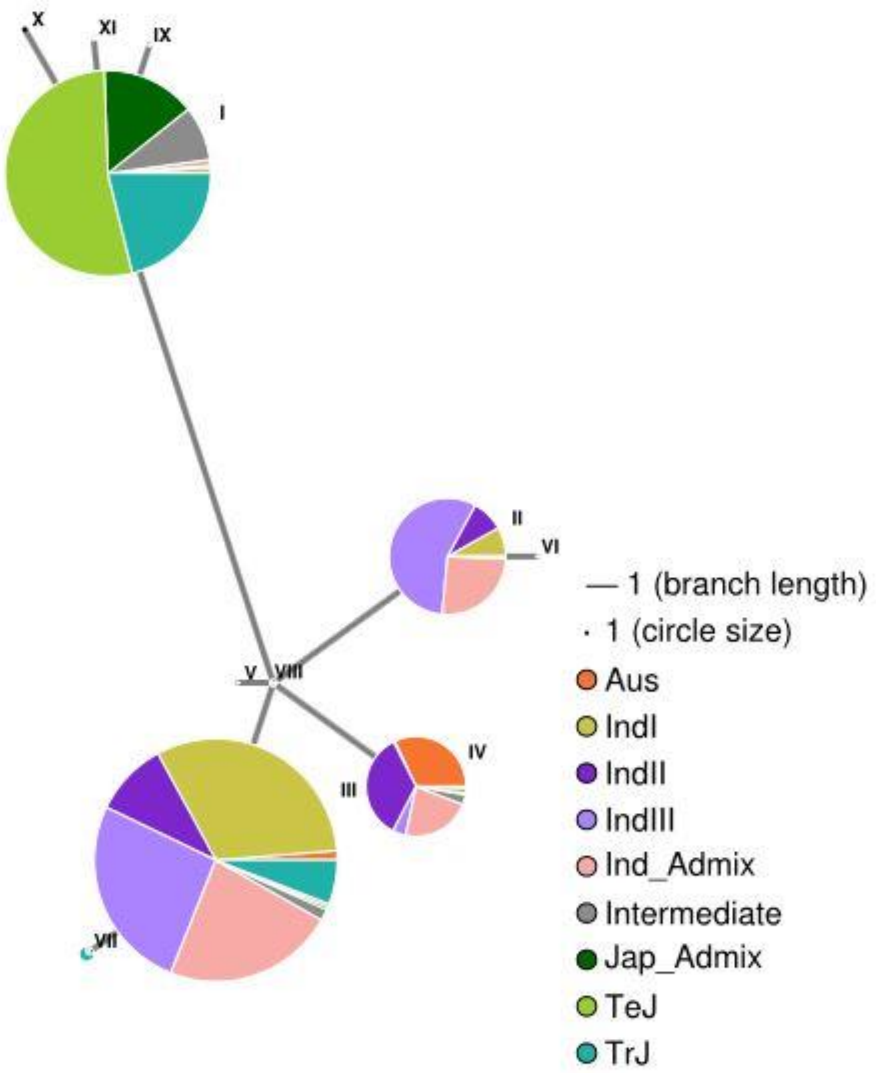

Os10g0485600

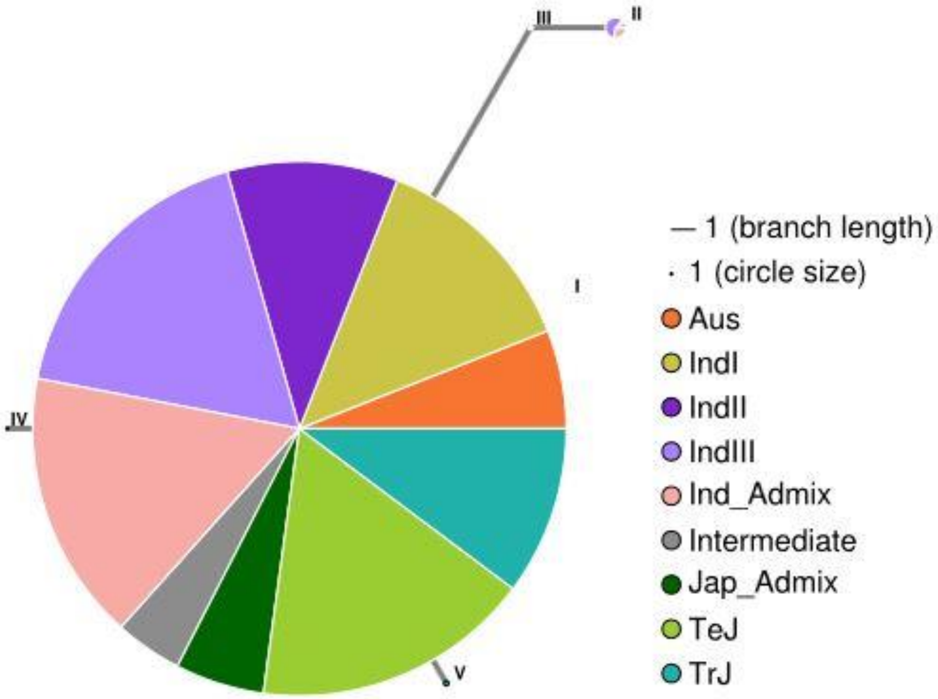

Os10g0567400

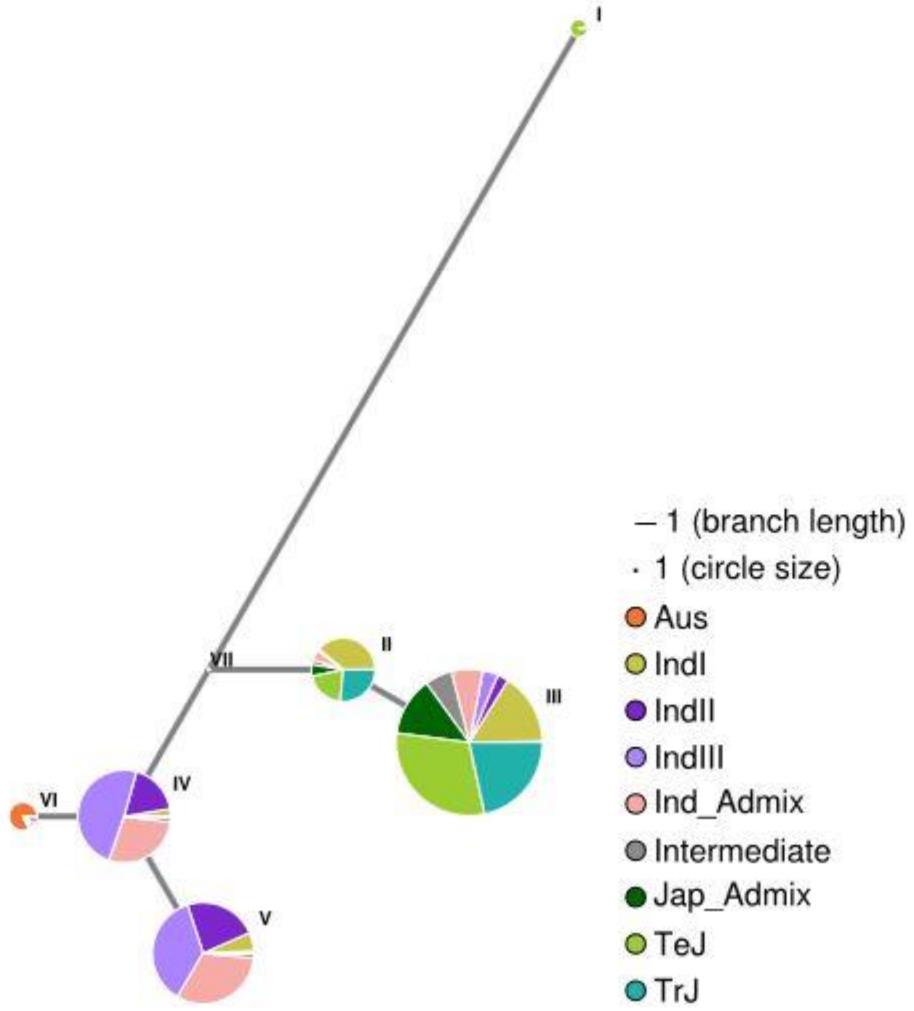

Os12g0631100

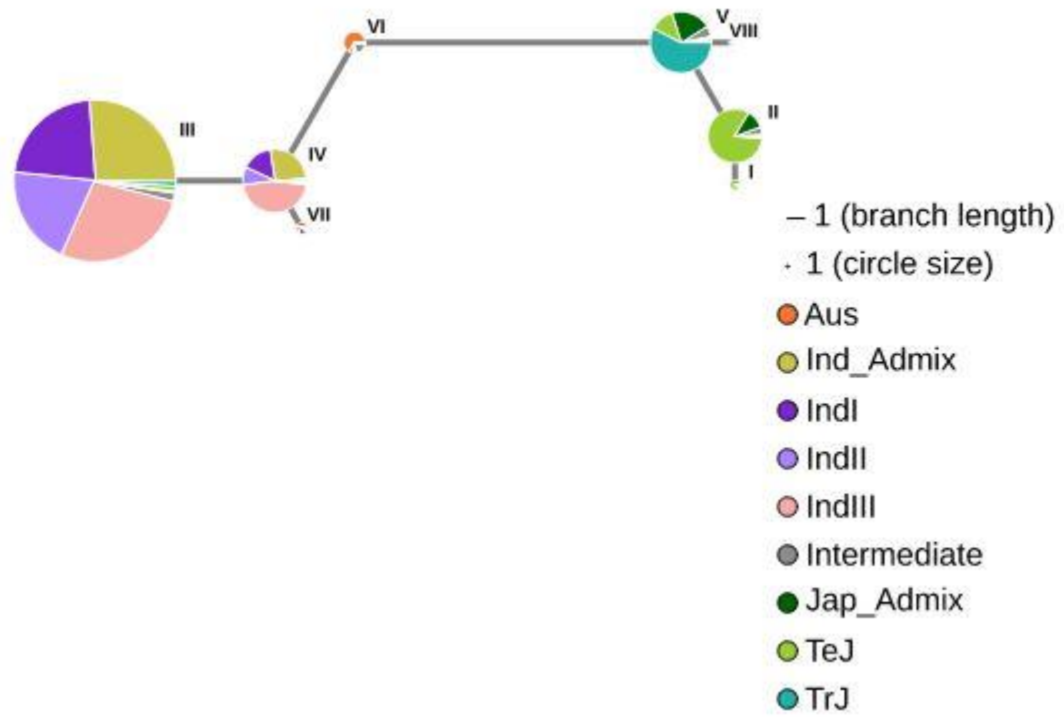

Os10g0112100

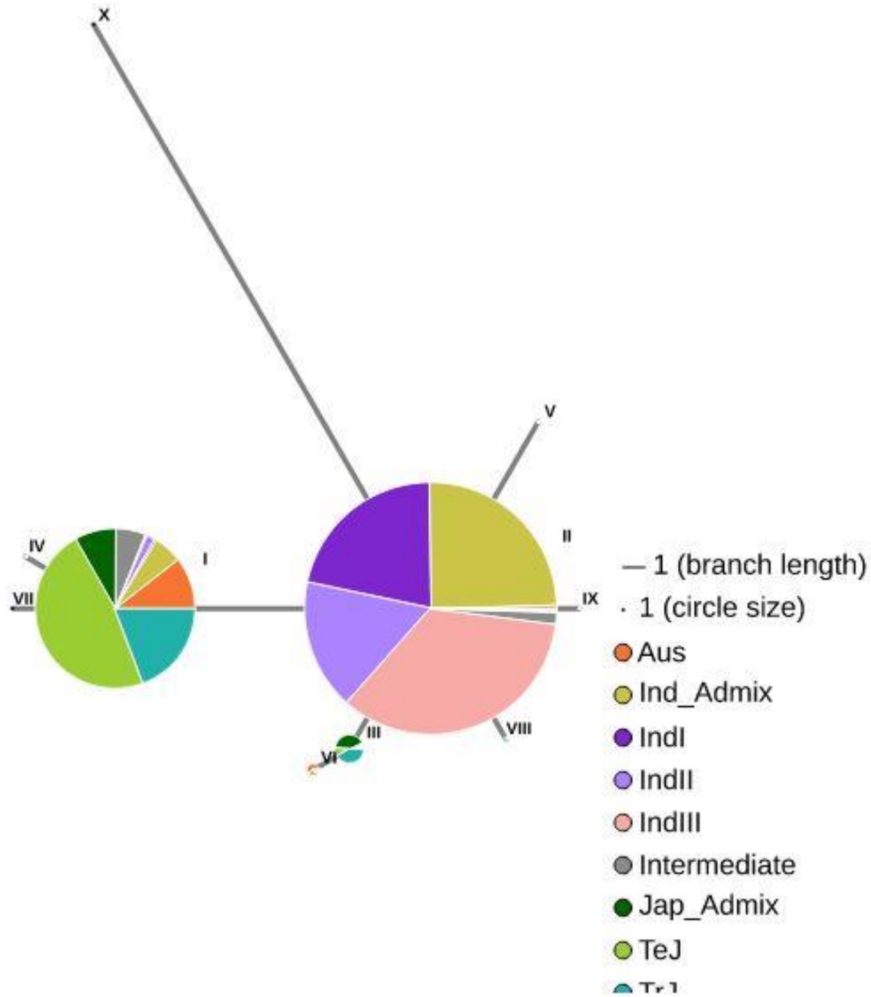

Os07g0232900

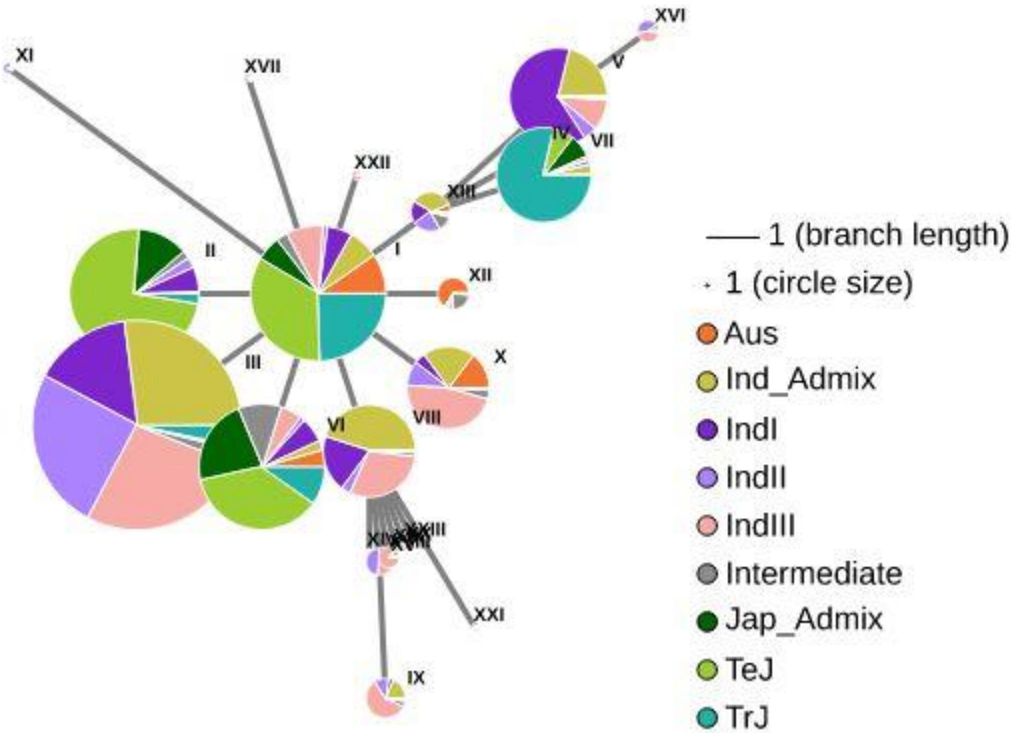

Os04g0620000

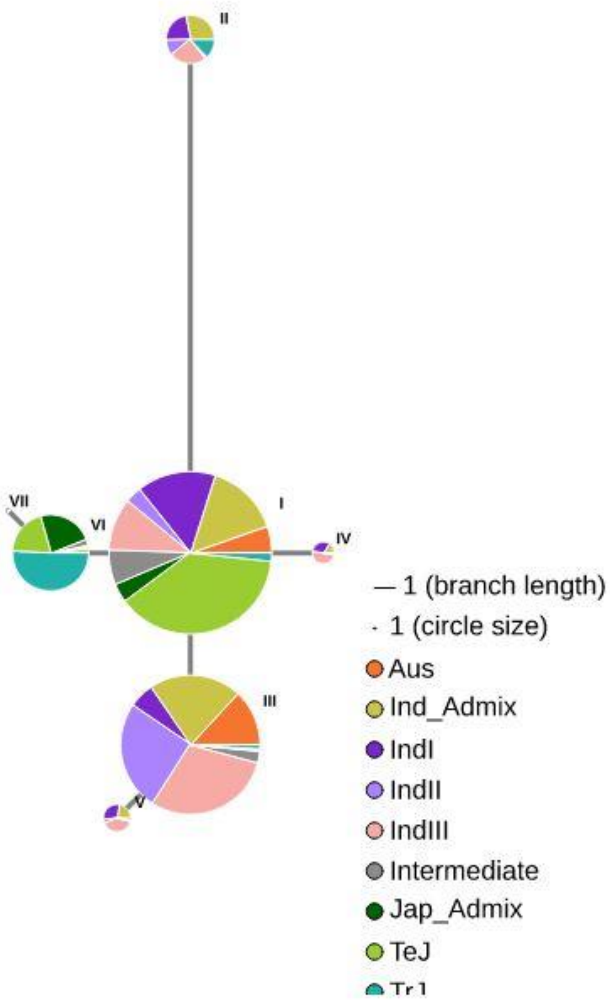

Os01g0952800

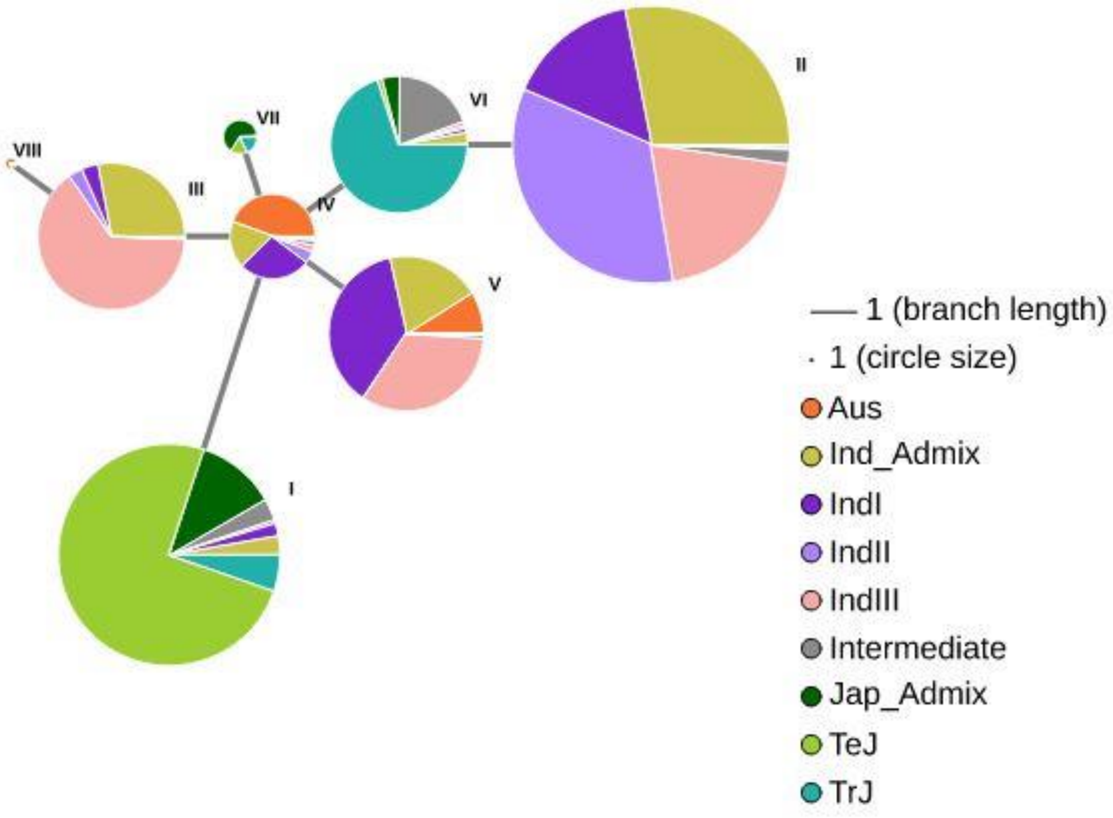

Os01g0762500

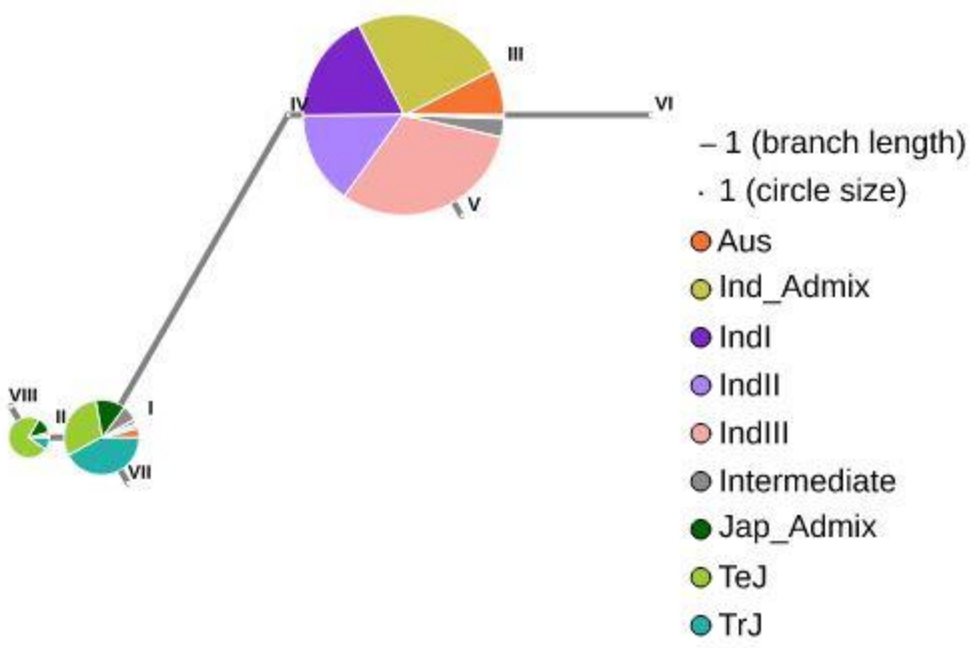

LOC_Os06g38120

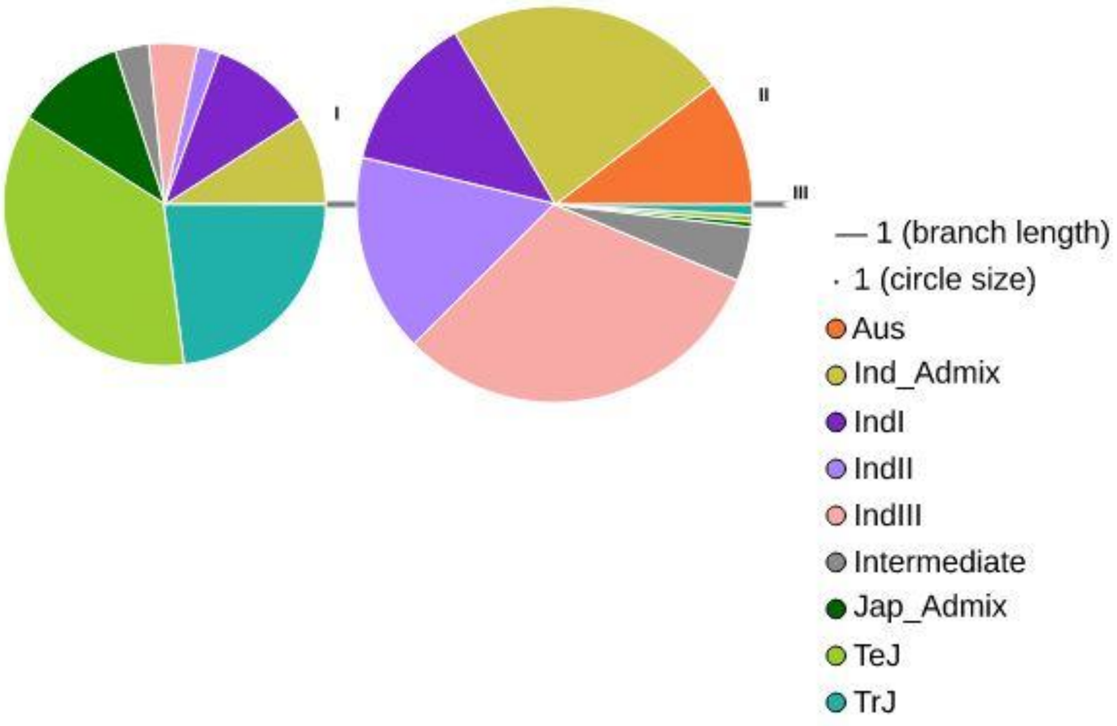

LOC_Os02g15169

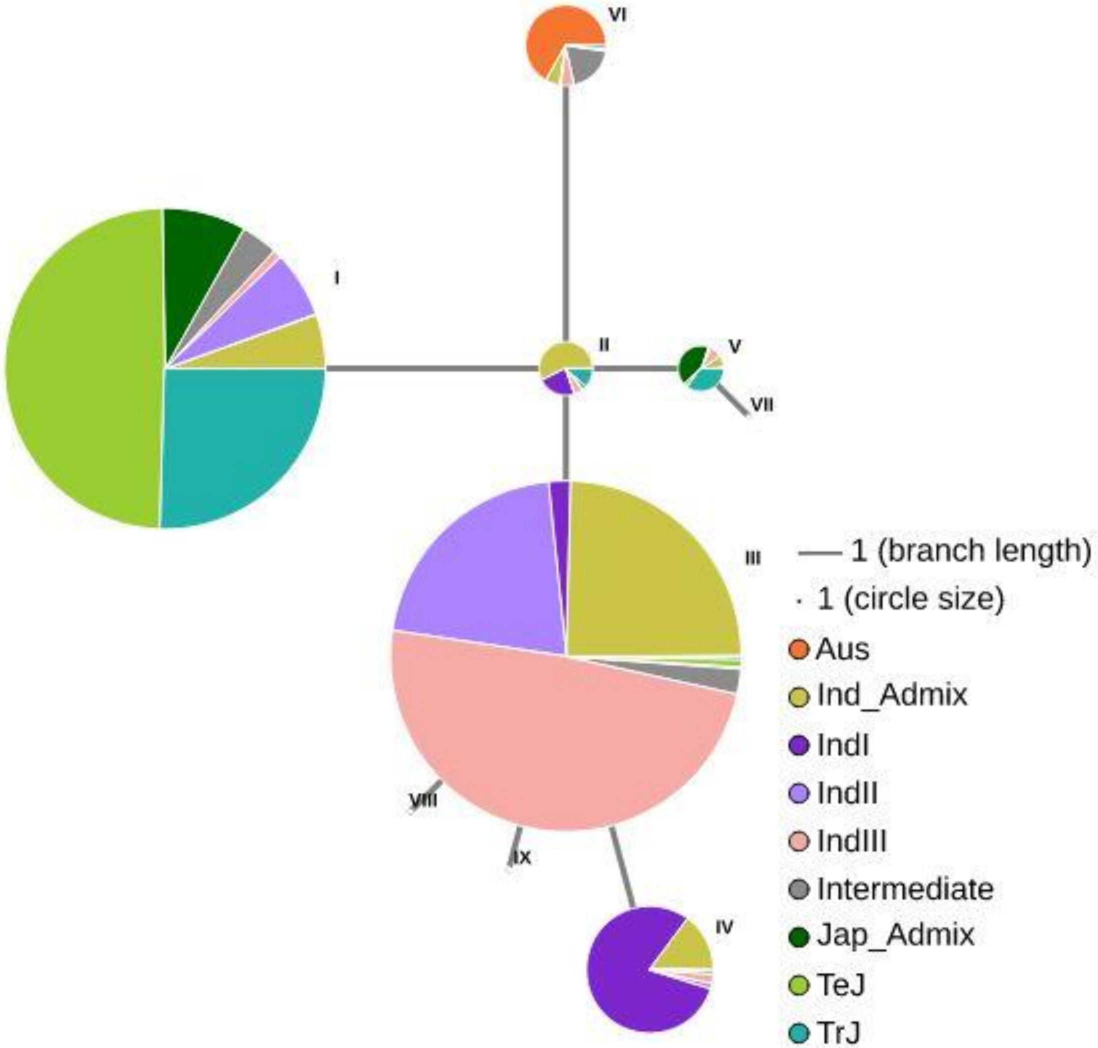

LOC_Os01g11900

Supplementary Dataset 3. Expression in different rice tissues among three cultivars for 80 nutritional and cooking quality related genes. The genes highlighted have specific expression in rice endosperm

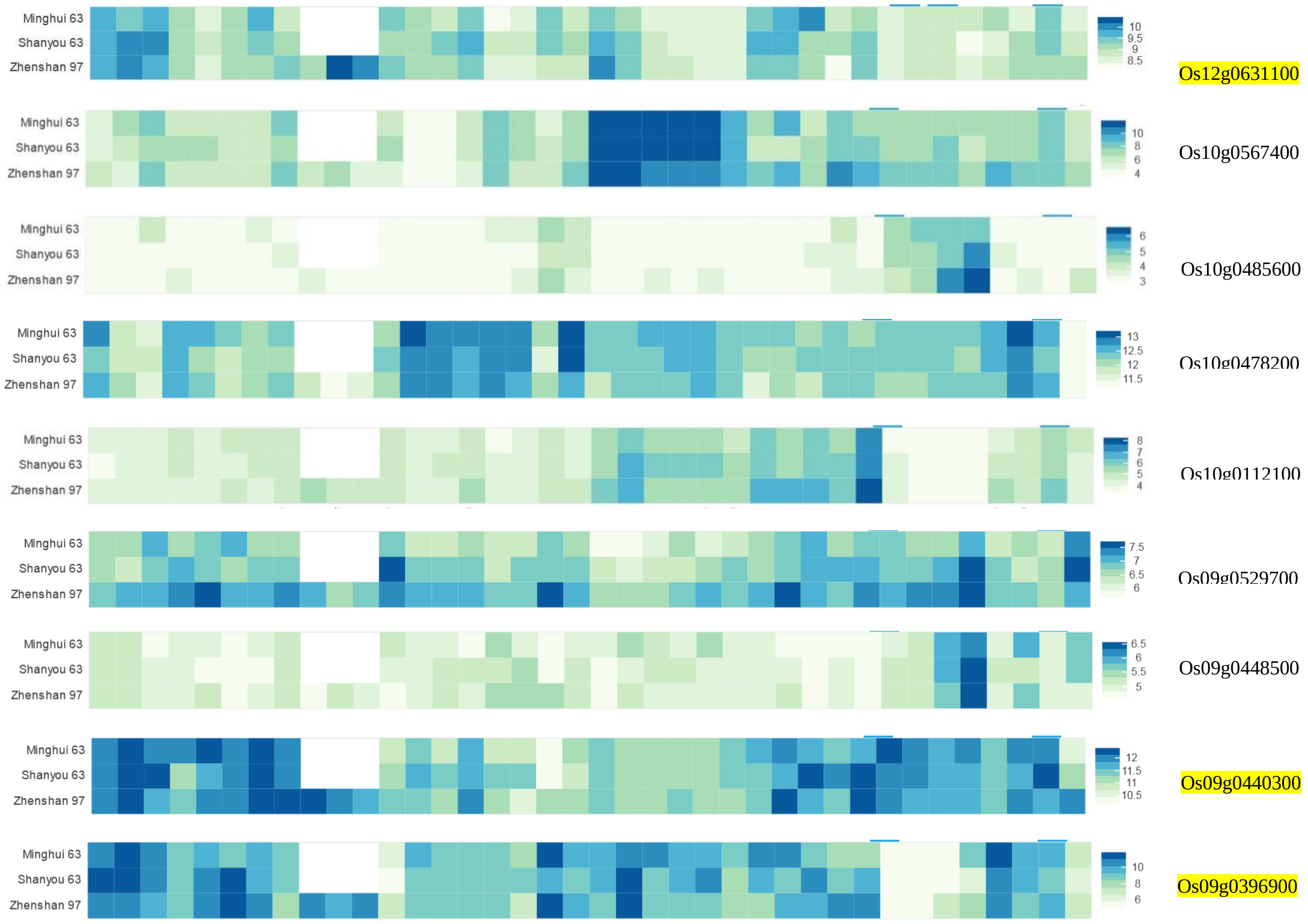

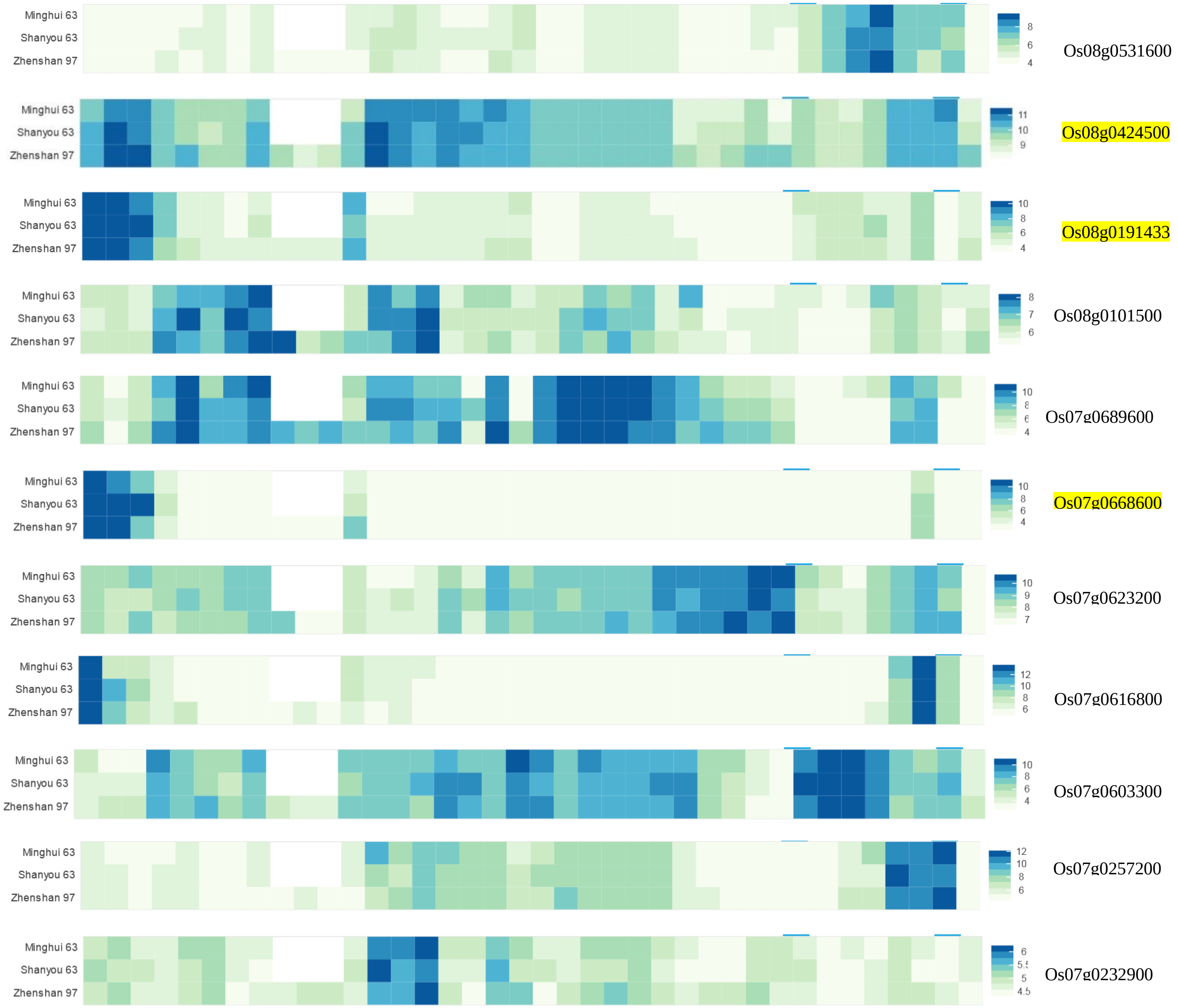
Minghui 63
Shanyou 63
Zhenshan 97
Os08g0531600
Os08g0424500
Os08g0191433
Os08g0101500
Os07g0689600
Os07g0668600
Os07g0623200
Os07g0616800
Os07g0603300
Os07g0257200
Os07g0232900

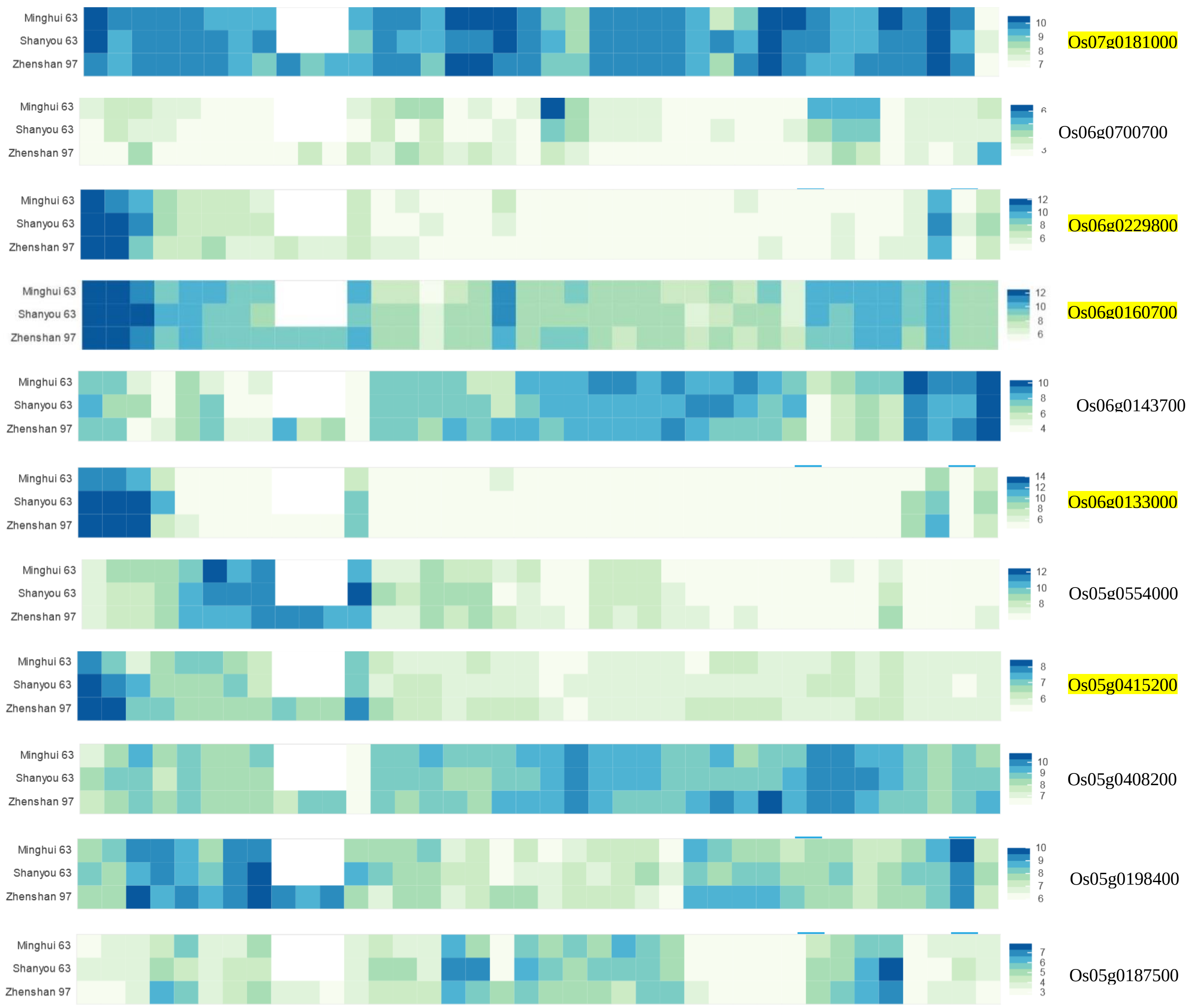
Minghui 63
Shanyou 63
Zhenshan 97
10
9
8
7
Os07g0181000
Minghui 63
Shanyou 63
Zhenshan 97
Os06g0700700
Minghui 63
Shanyou 63
Zhenshan 97
12
10
8
6
Os06g0229800
Minghui 63
Shanyou 63
Zhenshan 97
12
10
8
6
Os06g0160700
Minghui 63
Shanyou 63
Zhenshan 97
10
8
6
4
Os06g0143700
Minghui 63
Shanyou 63
Zhenshan 97
14
12
10
8
6
Os06g0133000
Minghui 63
Shanyou 63
Zhenshan 97
12
10
8
Os05g0554000
Minghui 63
Shanyou 63
Zhenshan 97
8
7
6
Os05g0415200
Minghui 63
Shanyou 63
Zhenshan 97
10
9
8
7
Os05g0408200
Minghui 63
Shanyou 63
Zhenshan 97
10
9
8
7
6
Os05g0198400
Minghui 63
Shanyou 63
Zhenshan 97
7
6
5
4
3
Os05g0187500

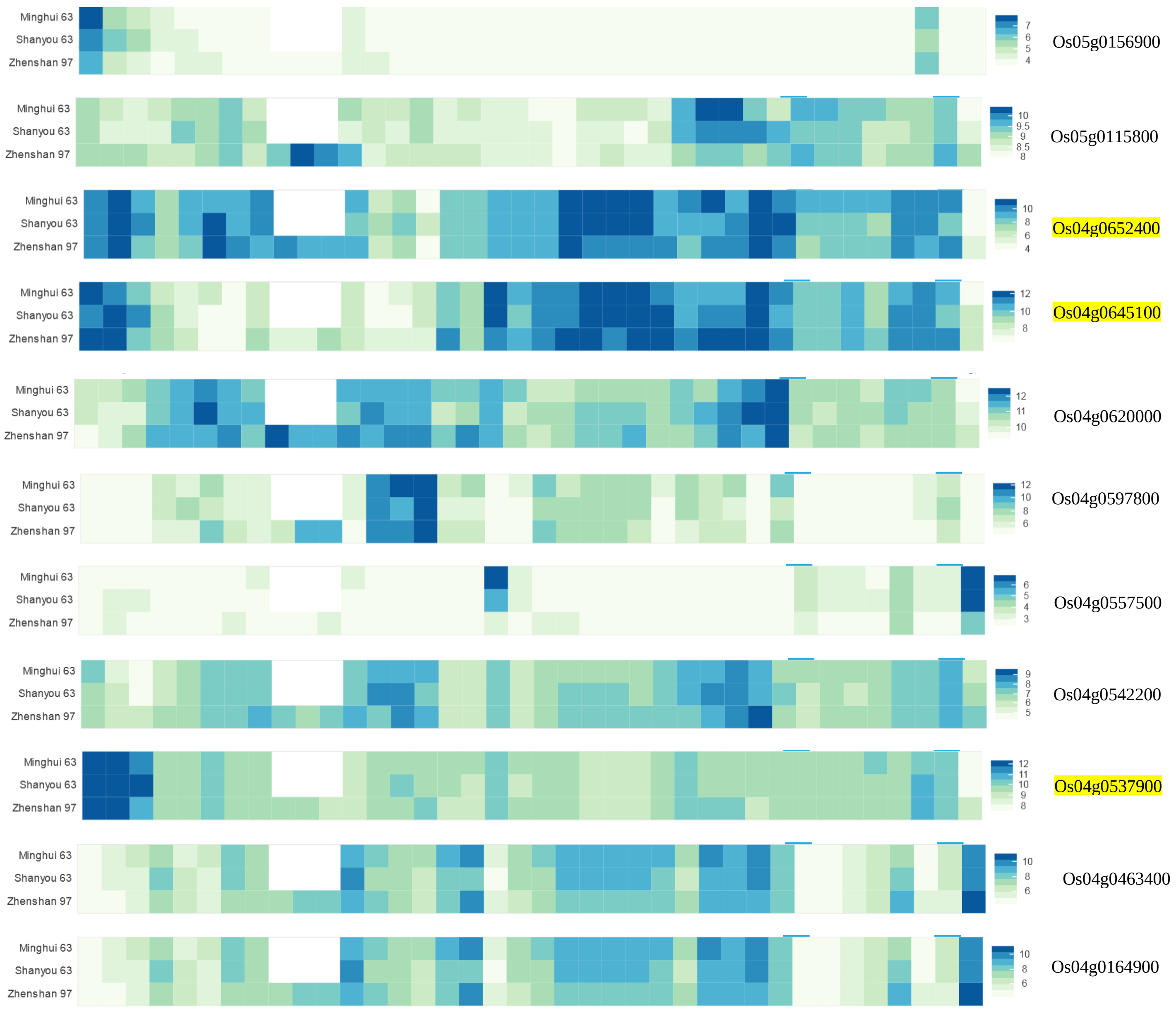
Minghui 63
Shanyou 63
Zhenshan 97
7
6
5
4
Os05g0156900
10
9.5
9
8.5
8
Os05g0115800
10
8
6
4
Os04g0652400
12
10
8
Os04g0645100
12
11
10
Os04g0620000
12
10
8
6
Os04g0597800
6
5
4
3
Os04g0557500
9
8
7
6
5
Os04g0542200
12
11
10
9
8
Os04g0537900
10
8
6
Os04g0463400
10
8
6
Os04g0164900

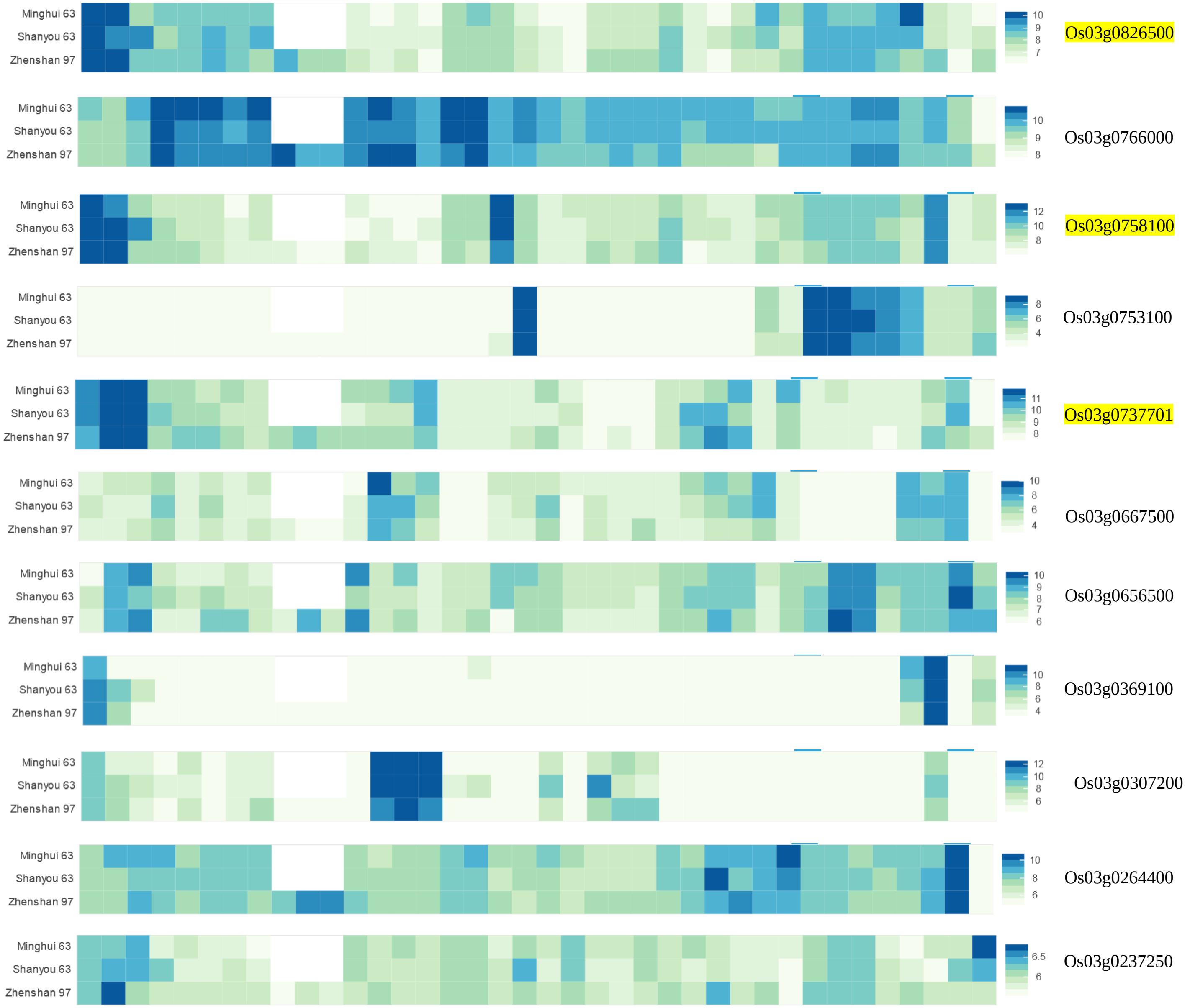
Minghui 63
Shanyou 63
Zhenshan 97
Os03g0826500
Os03g0766000
Os03g0758100
Os03g0753100
Os03g0737701
Os03g0667500
Os03g0656500
Os03g0369100
Os03g0307200
Os03g0264400
Os03g0237250

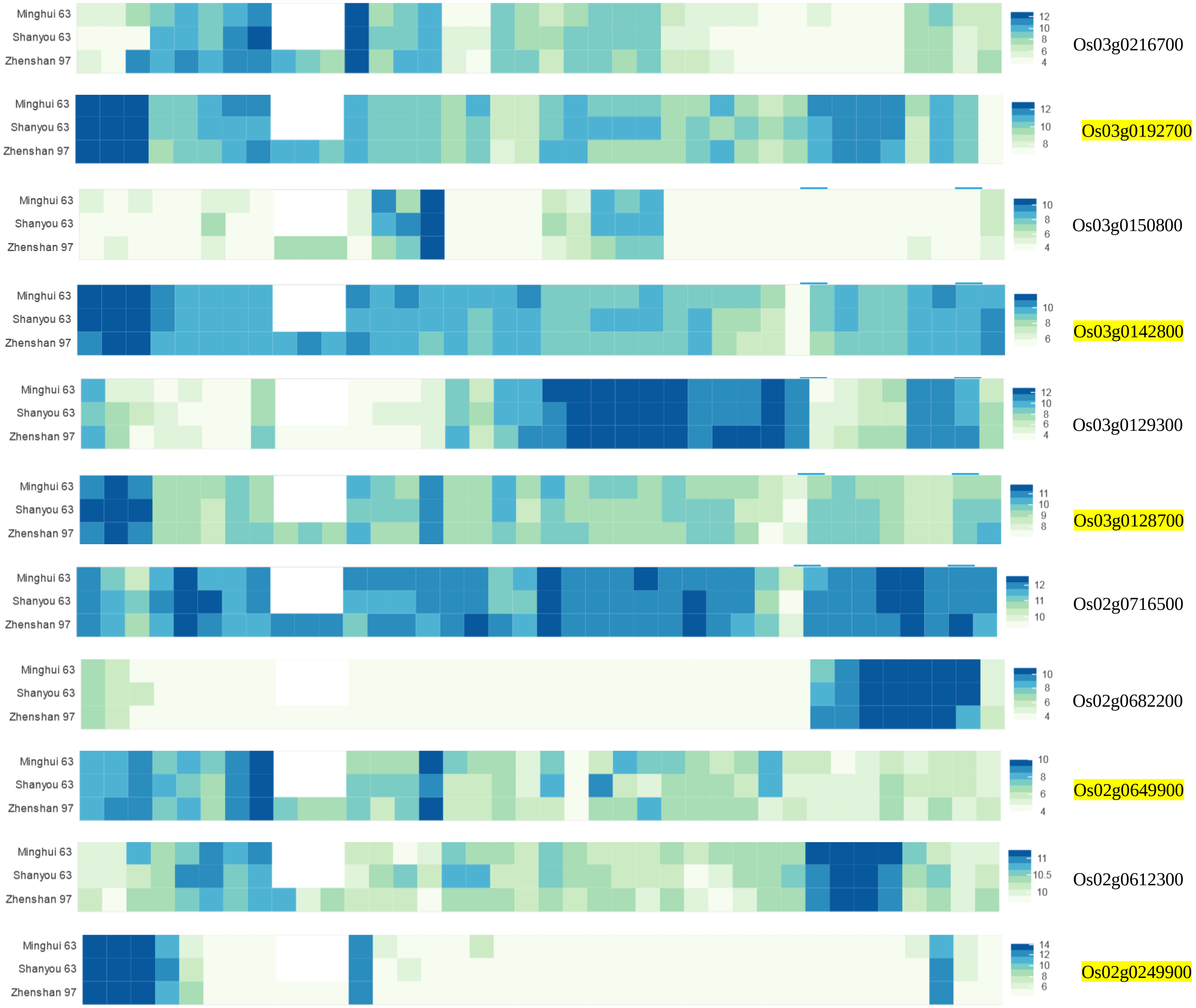
Minghui 63
Shanyou 63
Zhenshan 97
12
10
8
6
4
Os03g0216700
Minghui 63
Shanyou 63
Zhenshan 97
12
10
8
Os03g0192700
Minghui 63
Shanyou 63
Zhenshan 97
10
8
6
4
Os03g0150800
Minghui 63
Shanyou 63
Zhenshan 97
10
8
6
Os03g0142800
Minghui 63
Shanyou 63
Zhenshan 97
12
10
8
6
4
Os03g0129300
Minghui 63
Shanyou 63
Zhenshan 97
11
10
9
8
Os03g0128700
Minghui 63
Shanyou 63
Zhenshan 97
12
11
10
Os02g0716500
Minghui 63
Shanyou 63
Zhenshan 97
10
8
6
4
Os02g0682200
Minghui 63
Shanyou 63
Zhenshan 97
10
8
6
4
Os02g0649900
Minghui 63
Shanyou 63
Zhenshan 97
11
10.5
10
Os02g0612300
Minghui 63
Shanyou 63
Zhenshan 97
14
12
10
8
6
Os02g0249900

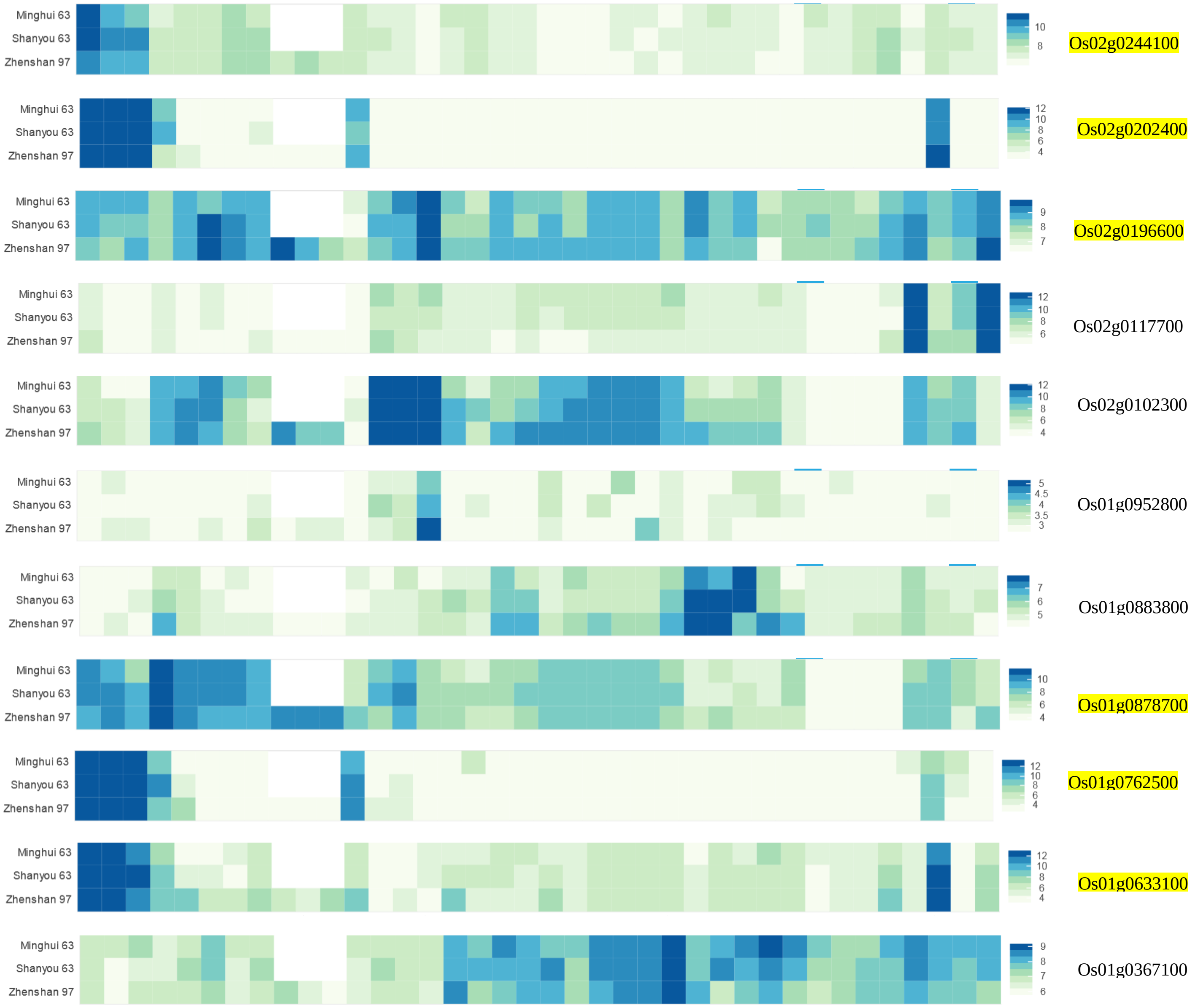
Minghui 63
Shanyou 63
Zhenshan 97
10
8
Os02g0244100
12
10
8
6
4
Os02g0202400
9
8
7
Os02g0196600
12
10
8
6
Os02g0117700
12
10
8
6
4
Os02g0102300
5
4.5
4
3.5
3
Os01g0952800
7
6
5
Os01g0883800
10
8
6
4
Os01g0878700
12
10
8
6
4
Os01g0762500
12
10
8
6
4
Os01g0633100
9
8
7
6
Os01g0367100

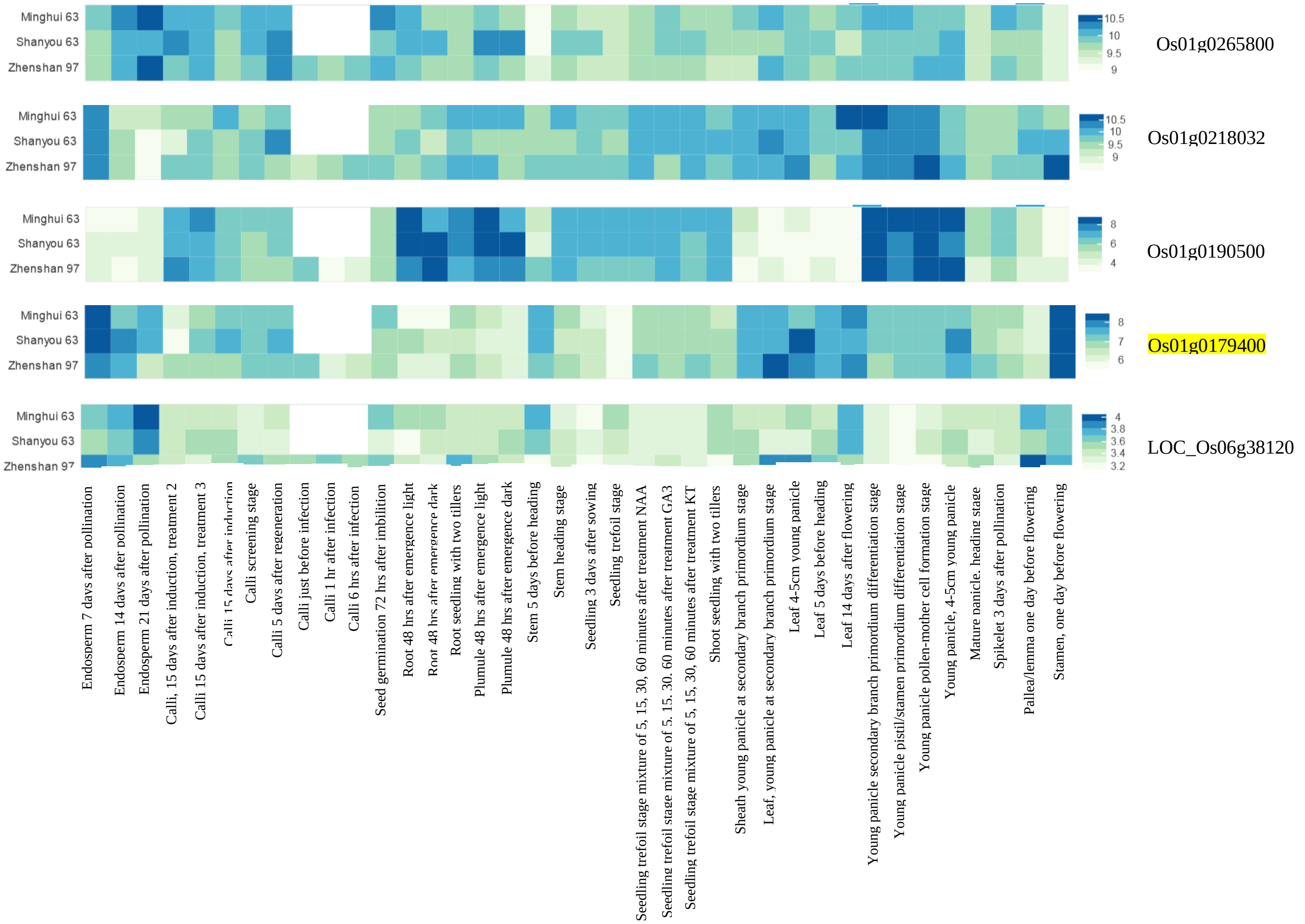

Minghui 63
Shanyou 63
Zhenshan 97
Os01g0265800
Os01g0218032
Os01g0190500
Os01g0179400
LOC_Os06g38120
10.5
10
9.5
9
8
6
4
7
3.8
3.6
3.4
3.2
Endosperm 7 days after pollination
Endosperm 14 days after pollination
Endosperm 21 days after pollination
Calli, 15 days after induction, treatment 2
Calli 15 days after induction, treatment 3
Calli screening stage
Calli 5 days after regeneration
Calli just before infection
Calli 1 hr after infection
Calli 6 hrs after infection
Seed germination 72 hrs after imbibition
Root 48 hrs after emergence light
Root seedling with two tillers
Plumule 48 hrs after emergence light
Plumule 48 hrs after emergence dark
Stem 5 days before heading
Stem heading stage
Seedling 3 days after sowing
Seedling trefoil stage
Seedling trefoil stage mixture of 5, 15, 30, 60 minutes after treatment NAA
Seedling trefoil stage mixture of 5, 15, 30, 60 minutes after treatment GA3
Seedling trefoil stage mixture of 5, 15, 30, 60 minutes after treatment KT
Shoot seedling with two tillers
Sheath young panicle at secondary branch primordium stage
Leaf, young panicle at secondary branch primordium stage
Leaf 4-5cm young panicle
Leaf 5 days before heading
Leaf 14 days after flowering
Young panicle secondary branch primordium differentiation stage
Young panicle pistil/stamen primordium differentiation stage
Young panicle pollen-mother cell formation stage
Young panicle, 4-5cm young panicle
Mature panicle, heading stage
Spikelet 3 days after pollination
Pallea/lemma one day before flowering
Stamen, one day before flowering
